# Synthesis of oxadiazole-2-oxide derivatives as potential drug candidates for schistosomiasis targeting S*j*TGR

**DOI:** 10.1186/s13071-021-04634-4

**Published:** 2021-04-26

**Authors:** Gongming Li, Qingqing Guo, Chao Feng, Huan Chen, Wenjiao Zhao, Shu Li, Yang Hong, Dequn Sun

**Affiliations:** 1 School of Life Science and Engineering, Southwest University of Science and Technology, Mianyang, 621010 China; 2 National Reference Laboratory of Animal Schistosomiasis, Key Laboratory of Animal Parasitology of Ministry of Agriculture, Shanghai Veterinary Research Institute, Chinese Academy of Agricultural Sciences, Shanghai, 200241 China; 3 Marine College, Shandong University (Weihai), Weihai, 264209 China

**Keywords:** Anti-schistosomiasis, *Schistosoma japonicum*, S*j*TGR, Oxadiazole-2-oxides, Furoxan

## Abstract

**Background:**

Schistosomiasis is a chronic parasitic disease that affects millions of people’s health worldwide. Because of the increasing drug resistance to praziquantel (PZQ), which is the primary drug for schistosomiasis, developing new drugs to treat schistosomiasis is crucial. Oxadiazole-2-oxides have been identified as potential anti-schistosomiasis reagents targeting thioredoxin glutathione reductase (TGR).

**Methods:**

In this work, one of the oxadiazole-2-oxides derivatives furoxan was used as the lead compound to exploit a series of novel furoxan derivatives for studying inhibitory activity against both recombinant *Schistosoma japonicum* TGR containing selenium (rS*j*TGR-Sec) and soluble worm antigen protein (SWAP) containing wild-type *Schistosoma japonicum* TGR (wtS*j*TGR), in order to develop a new leading compound for schistosomiasis. Thirty-nine novel derivatives were prepared to test their activity toward both enzymes. The docking method was used to detect the binding site between the active molecule and S*j*TGR. The structure–activity relationship (SAR) of these novel furoxan derivatives was preliminarily analyzed.

**Results:**

It was found that several new derivatives, including compounds **6a–6d**, **9ab**, **9bd** and **9be**, demonstrated greater activity toward rS*j*TGR-Sec or SWAP containing wtS*j*TGR than did furoxan. Interestingly, all intermediates bearing hydroxy (**6a–6d**) showed excellent inhibitory activity against both enzymes. In particular, compound **6d** with trifluoromethyl on a pyridine ring was found to have much higher inhibition toward both rS*j*TGR-Sec (half-maximal inhibitory concentration, IC_50_,7.5nM) and SWAP containing wtS*j*TGR (IC_50_ 55.8nM) than furoxan. Additionally, the docking method identified the possible matching sites between **6d** and *Schistosoma japonicum* TGR (S*j*TGR), which theoretically lends support to the inhibitory activity of **6d**.

**Conclusion:**

The data obtained herein showed that **6d** with trifluoromethyl on a pyridine ring could be a valuable leading compound for further study.
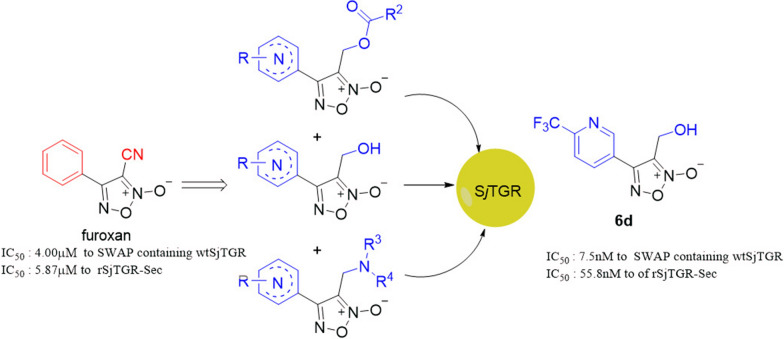

## Background

Schistosomiasis is one of the most widespread parasitic diseases in the world, and is one of the main tropical endemic diseases in Asia, Africa and South America [1]. It is estimated that approximately 779 million individuals around the world are at risk of schistosomes infection [2]. Schistosomiasis is caused by five species of the trematode flatworm: *Schistosoma japonicum* (*S. japonicum*), *Schistosoma mansoni* (*S. mansoni*), *Schistosoma haematobium*, *Schistosoma mekongi* and *Schistosoma intercalatum*. When the human body is infected, the worms will persist in the liver, hepatic portal system or the urinary tract system, leading to inflammation and obstructive disease [3]. In China, schistosomiasis is caused mainly by *Schistosoma japonicum*, and more than 77 thousand people are infected [4].

The most effective drug for the treatment of schistosomiasis is praziquantel (PZQ) (**1**), which has been used extensively since the 1980s [5] in the clinic without any backup drugs. The overuse of praziquantel has caused and accelerated the emergence of drug resistance. While PZQ is very effective against the adult forms of all schistosome species, it is weak for juvenile forms of all schistosomes, which might be the reason that PZQ cannot cure schistosomiasis completely. Although R-PZQ was approved by the Food and Drug Administration (FDA) as an orphan drug in 2018, it is also at risk of resistance, and there is still no alternative drug against the migratory juvenile and subadult worms [6–9]. Thus, there is an urgent need to look for potential reagents to discover novel alternatives to PZQ for the treatment of schistosomiasis.

**Fig. 1:**
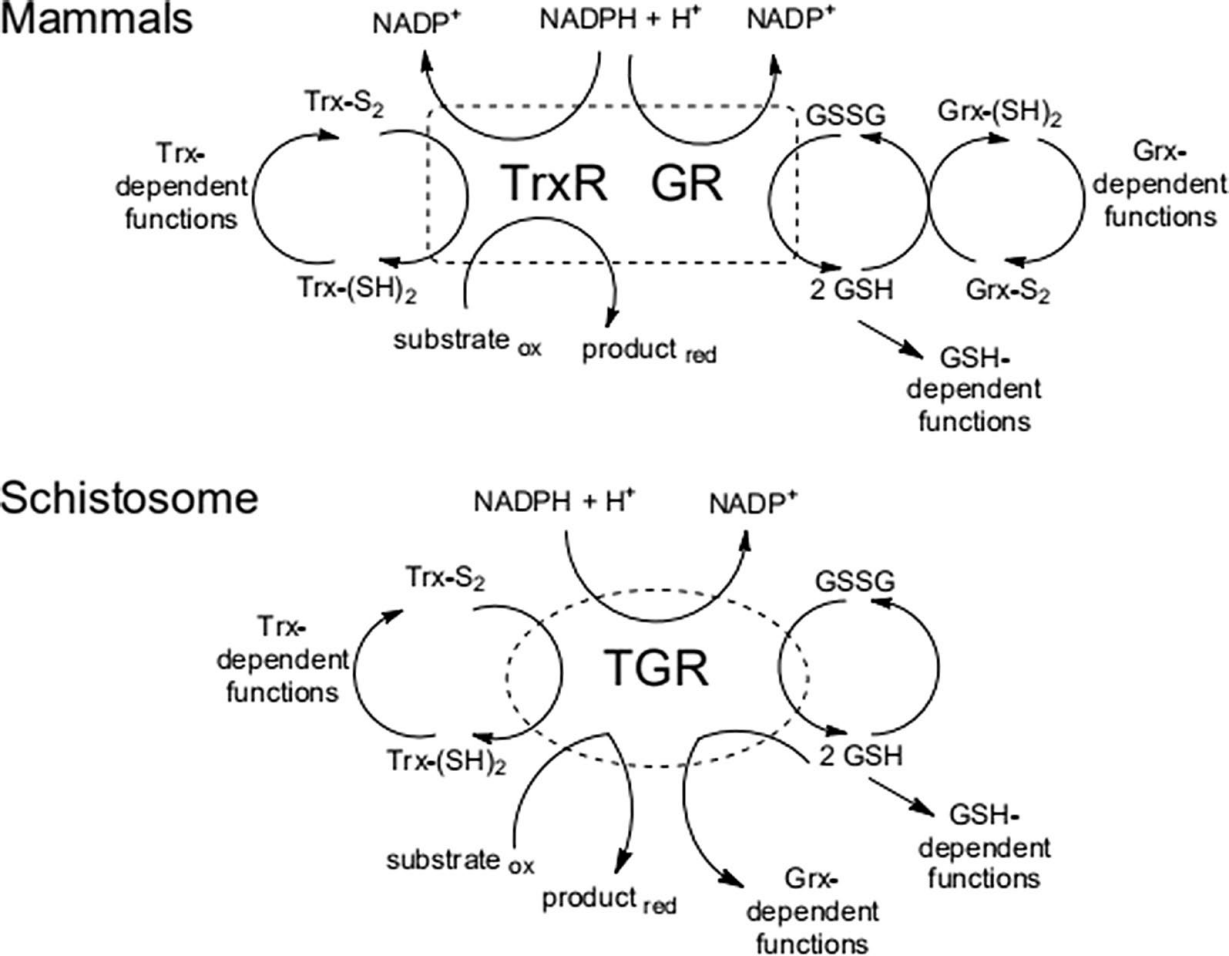
Redox pathways in mammals and *S. mansoni.*

It has been reported [1, 10–13] that instead of separate thioredoxin reductase (TrxR) and glutathione reductase (GR) enzymes of mammalian hosts, a single multifunctional selenocysteine-containing flavoenzyme, thioredoxin glutathione reductase (TGR), plays a critical role in maintaining proper redox balance for parasite survival in both *S. mansoni* and *S. japonicum* (Fig. 1). The cellular redox system is important in the physiological function of an organism. Given the biochemical differences between the redox metabolism of schistosomes and host (mammalian), TGR is inferred to be a potential target for new drug design.

In previous studies [1, 2, 10–13], some oxadiazole-2-oxide analogues have been shown to effectively inhibit S*m*TGR and S*j*TGR, even exhibiting prominent worm killing activity in vivo. Importantly, some oxadiazole-2-oxides showed good juvenile and adult *S. japonicum* killing activity in vitro [2]. Modifications around an oxadiazole-2-oxide skeleton have been demonstrated as a valuable strategy for discovering potential antischistosomal agents. The application of bioisosteres is an effective strategy in drug design. In the design of compounds, previous studies [1, 2, 10–13] have mainly substituted on the benzene ring and changed the CN moiety, but did not pay attention to modification of the benzene ring and other moieties in the furoxan structure with corresponding bioisosteres. Therefore, in this work, the strategy of applying bioisosteres was exploited to design novel oxadiazole-2-oxide derivatives.

On the other hand, the prokaryotic expression system has been used extensively for protein expression due to its rapid growth rate, capacity for continuous fermentation, and relatively low cost. Due to the lack of the post-translational modifications of this expression system, the bioactivity, function, structure, solubility and so on will be affected to the expressed functional products. Although eukaryotic expression systems can resolve this problem, just like a two-edged sword, some disadvantages such as low yield, demanding culture conditions and higher cost cannot be totally avoided [14]. Thus, the special soluble worm antigen protein (SWAP) containing wtS*j*TGR extracted from worms might be a better alternative target for studying the bioactivity. It would reflect the actual situation in advance when *Schistosoma japonicum* is treated with chemical compounds. Therefore, the target compounds herein were also applied to test the inhibition activity on SWAP containing wtS*j*TGR in addition to rS*j*TGR-Sec.

**Fig. 2:**
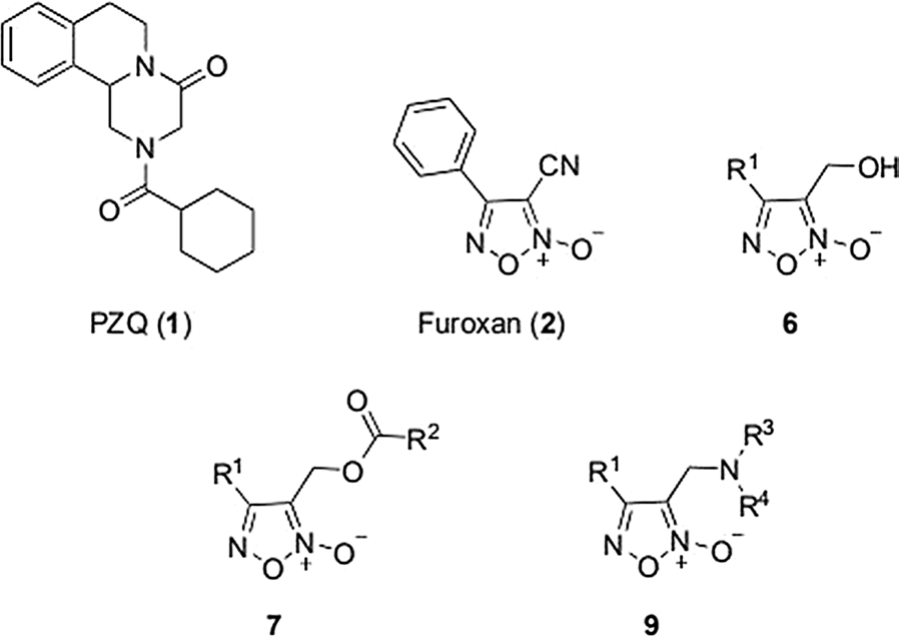
The structure of praziquantel (PZQ) and furoxan and the general structure of compounds **6**, **7** and **9**..

In this work, two series of novel furoxan derivatives (**7** and **9**) (Fig. 2) were obtained through two methods: either by replacing the phenyl moiety of furoxan with its bioisosteres pyridine or substituted pyridine, or by modifying the cyano moiety into an ester or amine group. Because the fluorine atom possesses a strong electron-withdrawing effect and high lipophilicity, appropriate introduction of fluorine atoms into molecules can improve their biofunction [15]. Therefore, several fluorine-containing derivatives were also intentionally synthesized. The target compounds (**7** and **9**) and their corresponding intermediates (**6**) (Fig. 2) were tested for their inhibitory activity on TrxR from rS*j*TGR-Sec and SWAP containing wtS*j*TGR.. Based on the result obtained, the SAR of these novel furoxan derivatives was preliminarily analyzed.

## Methods

### Reagent

All chemical reagents involved in synthetic procedures were purchased commercially from J&K Chemicals. The chemical reagents used in activity measurement, including dimethyl sulfoxide (DMSO), ethylene diamine tetraacetic acid (EDTA), dithionitrobenzoic acid (DTNB), and MTT, were purchased from Sigma-Aldrich Inc.

### Synthesis of oxadiazole-2-oxide derivatives

**Scheme 1:**
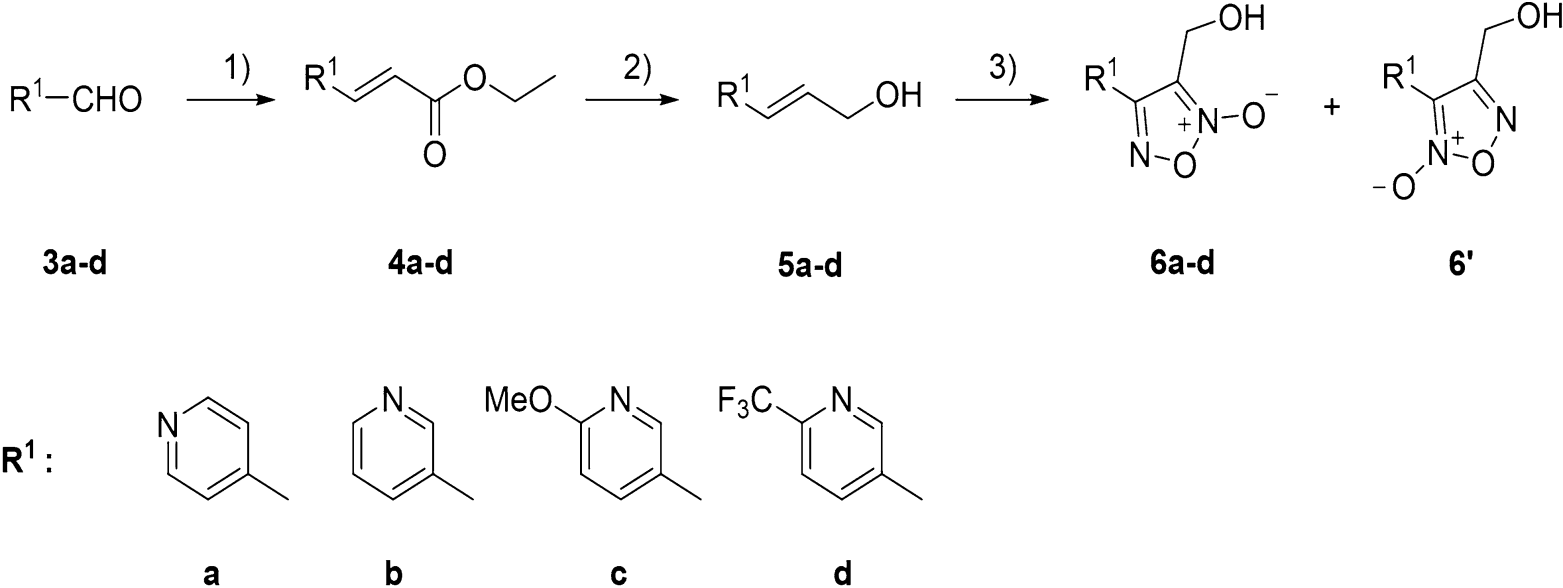
Synthesis of compounds **6a–6d**. Reagents and conditions: (1) NaH, THF, triethyl-phosphonoacetate, r.t; (2) DIBAL-H, DCM, −78 °C; (3) NaNO_2_, AcOH, 60 °C.THF, tetrahydrofuran; DIBAL-H, diisobutylaluminum hydride; DCM, dichloromethane; r.t., room temperature.

The synthesis of compounds **6a–d** commenced with the synthesis of α,β-unsaturated esters (**4a–d**) from substituted nicotinaldehydes (**3a–d**) by Wittig reaction [16]. Compounds **4a–d** were then reduced using diisobutylaluminum hydride (DIBAL-H) [17] to produce alcohols **5a–d**. Subsequent sodium nitrate-mediated cyclization [17] gave oxadiazole-2-oxides (**6a–d**) (Scheme 1).

**Scheme 2:**
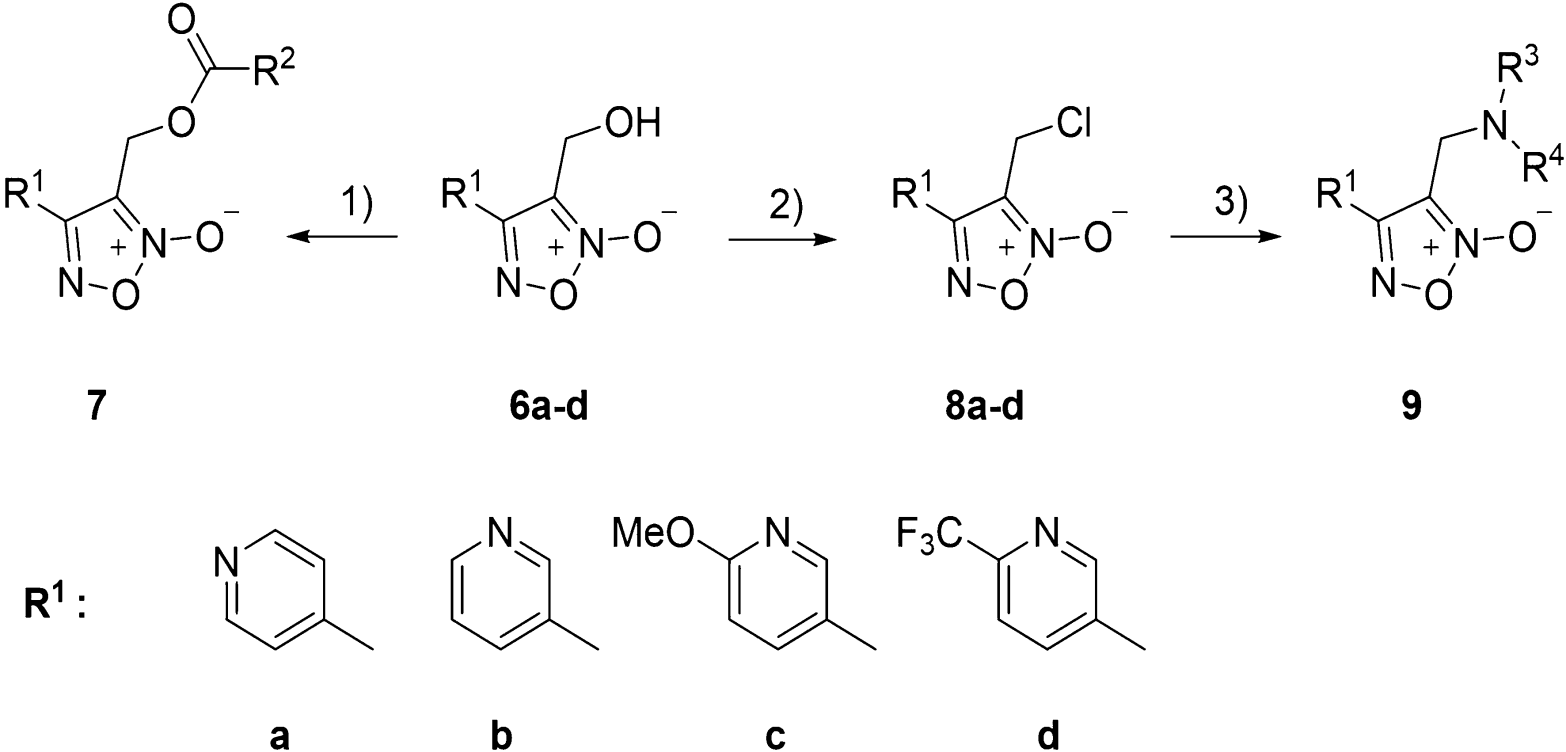
Synthesis of compound **7** and **9**. Reagents and conditions: (1) Acyl chloride, Et_3_N, DCM, DMAP, r.t; (2) benzene sulfonyl chloride, Et_3_N, DCM, DMAP, r.t; (3) amine, K_2_CO_3_, NaI, MeCN, r.t. DCM, dichloromethane; DMAP, dimethylaminopyridine; r.t., room temperature.

In Scheme 2, compound **7** was readily prepared by esterification of alcohol **6a–d** with various acyl chlorides using triethylamine and dimethylaminopyridine (DMAP) as base and catalyst, respectively. The synthesis of target compounds **9** began with the treatment of compounds **6a–d** with benzenesulfonyl chloride to generate unstable intermediates **8a–d,** which were aminated in situ to afford various target amines **9**. As a result, a total of 39 novel oxadiazole-2-oxide derivatives were synthesized and their structures were determined by nuclear magnetic resonance (NMR) and high-resolution mass spectrometry (HRMS). The preparation process and spectrum of the compounds are detailed in Additional file 1.

### Crystallography

Normally, two isomers **6** and **6'** were obtained (Scheme 1) and one of them was the main product after cyclization. In order to confirm the conformation of main product, its crystal was selected and was measured on a SuperNova diffractometer (dual-source, Cu at zero, EosS2). The crystal was kept at 150 K during data collection. Using Olex2 [18], the structure was solved with the Superflip structure solution program using charge-flipping and refined with the ShelXL refinement package using least squares minimization.

### Preparation of the rS*j*TGR-Sec and the SWAP containing wtS*j*TGR

The rS*j*TGR-Sec was expressed and harvested as described previously [13, 19]. Most of rS*j*TGR-Sec was expressed in *E. coli* as a soluble His-tagged fusion protein when bacterial growth occurred at 24 °C. The rS*j*TGR-Sec was purified by His Mag™ Agarose beads according to manufacturer’s instructions. The concentration of purified rS*j*TGR-Sec was determined using a Bradford assay kit (Sangon, China), and the protein was identified by sodium dodecyl sulfate polyacrylamide gel electrophoresis (SDS-PAGE) and stored at 4 °C [20].

The SWAP containing wtS*j*TGR was prepared from adult schistosomes. Rabbits were infected with cercariae of *S. japonicum* by exposing their abdominal skin and were sacrificed at 42 days after infection to collect the adult schistosomes by perfusion. The worms were washed three times with phosphate-buffered saline (PBS). After sufficient grinding of the adult schistosomes in PBS, a suitable amount of benzoyl sulfonyl fluoride (PMSF) solution was added to obtain a final concentration of 1 mM. The homogenate was centrifuged at 4 °C, 12,000×*g* for 20 min [1, 10]. The concentration of supernatant containing wtS*j*TGR was determined by Bradford assay kit (Sangon, China).

### Compound inhibition of rS*j*TGR-Sec and SWAP containing wtS*j*TGR

The novel furoxan derivatives in this work were dissolved in DMSO with a storage solution concentration of 64 millimoles per liter. The analysis of TrxR activity of SWAP containing wtS*j*TGR and rS*j*TGR-Sec was performed based on methods described previously [21]. Analysis of the inhibitory activity of the compounds against the TrxR activity of SWAP containing wtS*j*TGR and rS*j*TGR-Sec described in this paper was also based on a previously described method [2]. The reaction was performed in 0.1 M potassium phosphate buffer (pH 7.0) containing 10 mM EDTA, 100 µM nicotinamide adenine dinucleotide phosphate (NADPH), 0.2 µg supernatant of SWAP containing wtS*j*TGR or 0.6 µg rS*j*TGR-Sec, and the studied compounds with different concentrations at 25 °C in a final volume of 200 μL, and was initiated by adding 3 mM DTNB. The increase of the absorbance at 412 nm was monitored, and the IC_50_ values of the compounds were calculated by GraphPad Prism v6.0c software; The results are reported in Table 1. All assays were done in triplicate.

**Table 1:**
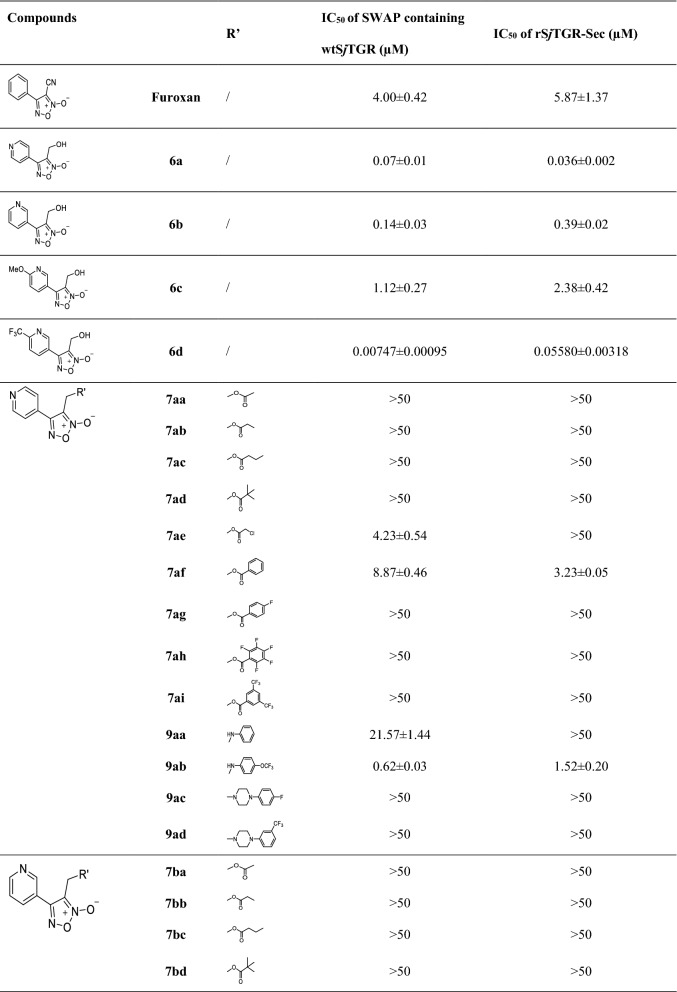

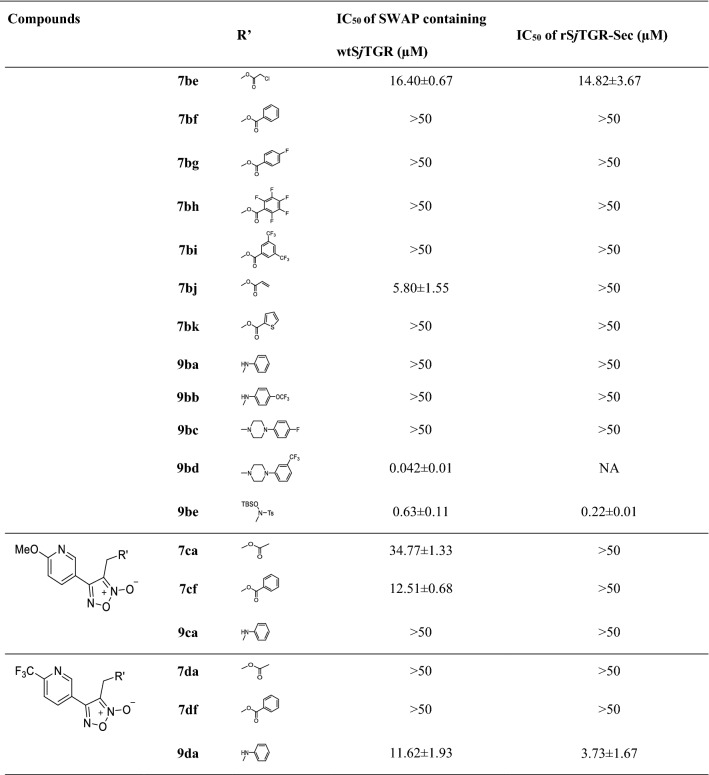
Structures of oxadiazole-2-oxide derivatives and their inhibitory activity against the TrxR activity of SWAP containing wtS*j*TGR and rS*j*TGR-Sec

IC50 values > 50 μM signify lack of fitted curve through the dose-response data—that is, flat response within the range tested

*NA* not applicable

### Docking study

The crystal structure of S*j*TGR is a homodimer, and we retained chain B for docking studies [22]. The docking was conducted by AutoDock 4, and the results are displayed in Figs. 4 and 5. The results were further processed and displayed by PyMOL2.3.2. The hydrophobic effects between inhibitors and protein were analyzed by LigPlot^+^v.2.1, and the result is shown in Fig. 5.

## Results and discussion

### Synthesis and crystallography

**Scheme 3:**
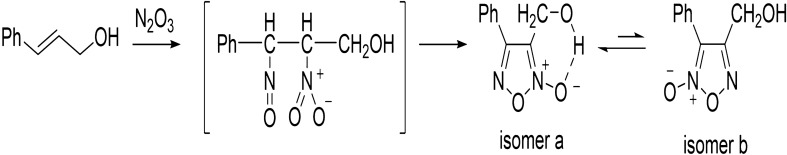
The mechanism of forming an oxadiazole-N-oxide ring reported by Gasco and co-workers.

In total, 39 novel oxadiazole-2-oxide derivatives were synthesized following Schemes 1 and 2. The structures of these compounds are detailed in Table 1. During synthesis of the important intermediate **6**, two isomers were inevitably formed when the oxadiazole-N-oxide ring was constructed by sodium nitrate-mediated cyclization. Gasco and co-workers reported [23] that intramolecular hydrogen bond played a crucial role on the formation of the main isomer during cyclization and could stabilize the structure of the isomer a (Scheme 3).

**Fig. 3:**
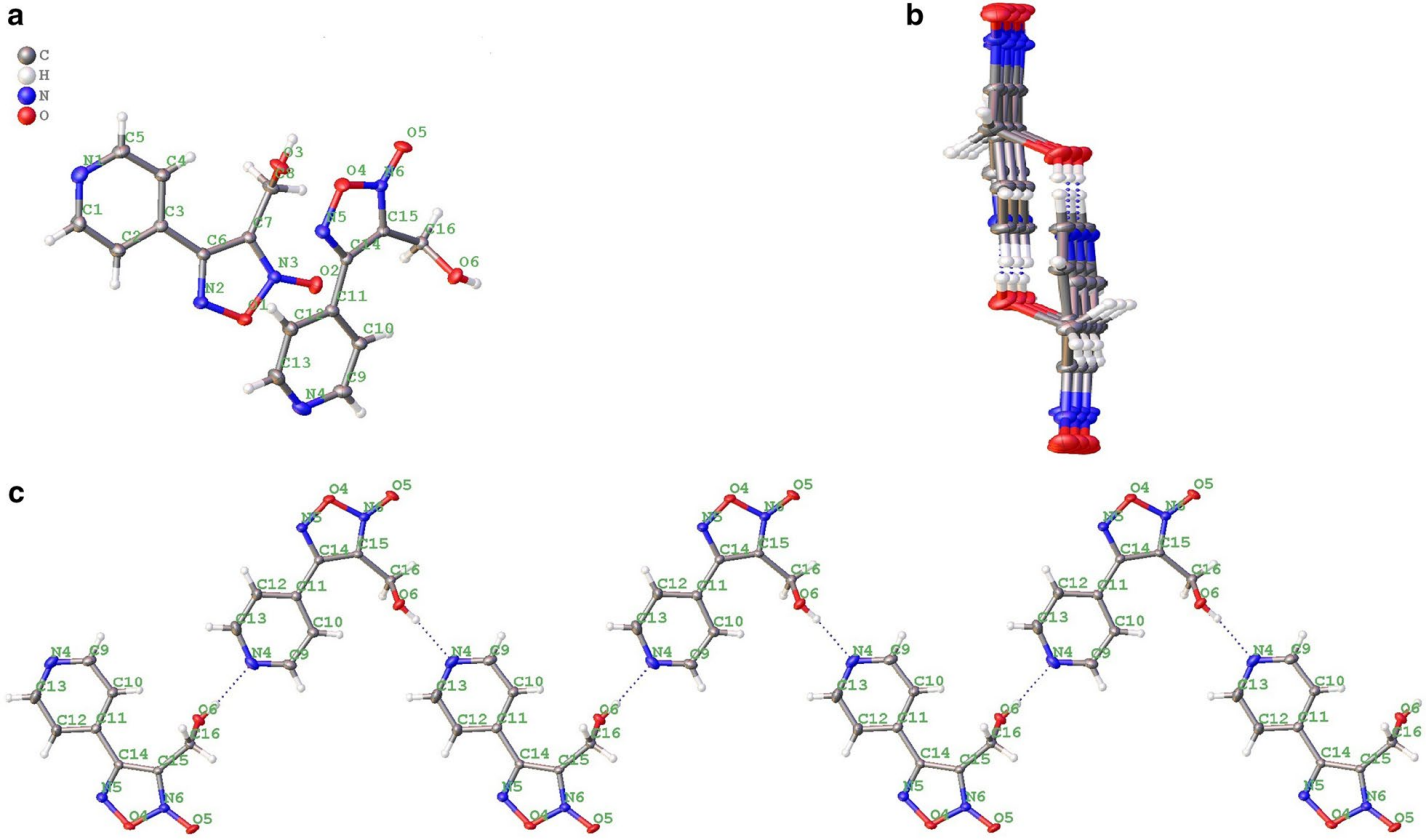
**a** Crystal structure of **6a**; **b** hydrogen bond helix chain formed by molecule’s nitrogen atoms labeled N1, N2 and N3; **c** hydrogen bond zigzag chain formed by the molecule’s nitrogen atoms labeled N4, N5 and N6.

For our case, two isomers, **6** and **6'**, were obtained (Scheme 1), and one of them was the main product after cyclization. To confirm the position of the N-oxide moiety, and whether the main product was **6** or **6**', the crystal of the main product was obtained. It was interesting that the X-ray confirmed that the main product had a similar conformation with isomer a in Scheme 3, and specifically, the main product was **6a** (Scheme 1). The crystal structure showed that there were two independent molecules in an asymmetric unit (Fig. 3a). One molecule’s nitrogen atoms were labeled N1, N2 and N3, and the other molecule’s nitrogen atoms were labeled N4, N5 and N6. However, intermolecular hydrogen bonds, instead of intramolecular hydrogen bonds, were found in the crystal of **6a**. The molecule’s nitrogen atoms labeled N1, N2 and N3 formed a hydrogen bond helix chain (Fig. 3b), while the nitrogen atoms labeled N4, N5 and N6 formed a hydrogen bond zigzag chain (Fig. 3c). This result also indicated that after the pyridine replaced the benzene, the intramolecular hydrogen bond shown in Scheme 3 might not be necessary for the formation of main isomer **6a–d**.

### TGR activity assessment

With furoxan as the positive control, oxadiazole-2-oxides (**6, 7, 9**) were evaluated for their inhibitory activity against both rS*j*TGR-Sec and SWAP containing wtS*j*TGR under different concentrations (Table 1). As shown in Table 1, all intermediates bearing hydroxy (**6a–6d**) showed better inhibitory activity against both enzymes than furoxan. The pyridine ring in the structure of compound **6a** had a stronger electron-withdrawing effect on the oxadiazole-2-oxide ring than compound **6b**, and **6a** (IC_50_ 70nM of SWAP containing wtS*j*TGR, IC_50_ 36nM of rS*j*TGR-Sec) had better inhibitory activity on both enzymes than **6b** (IC_50_ 0.14µM of SWAP containing wtS*j*TGR, IC_50_ 0.39µM of rS*j*TGR-Sec). Compared with **6b**, the electron-donating methoxy group at the ortho position of pyridine (like compound **6c**) decreased the inhibitory activity (**6c**, IC_50_ 1.1µM of SWAP containing wtS*j*TGR, IC_50_ 2.4µM of rS*j*TGR-Sec). Notably, electron-withdrawing group trifluoromethyl made **6d** exhibited excellent inhibitory effect (IC_50_ 7.5nM of SWAP containing wtS*j*TGR, IC_50_ 55.8nM of rS*j*TGR-Sec). These data indicated that the electron-withdrawing substituents very possibly provided enhanced potency.

To further investigate the relationship between the structure and bioactivity, modification of hydroxyl group into the ester group and amine, respectively, afforded compounds **7** and **9**. It was found that the ester group could not effectively improve the inhibitory effect, although the ethyl ester (**7ca**), chloracetyl ester (**7ae, 7be**), benzoyl ester (**7af, 7cf**) and acryloyl ester (**7bj**) exhibited inhibitory effects similar to furoxan. Only **7ca** (IC_50_ 34.77µM of SWAP containing wtS*j*TGR) showed inhibitory effects against SWAP containing wtS*j*TGR, but not as good as furoxan. Among chloracetyl esters, perhaps because the pyridine ring of **7ae** had a stronger electron-withdrawing effect, **7ae** (IC_50_ 4.23µM of SWAP containing wtS*j*TGR) showed a slight advantage over **7be** (IC_50_ 16.40µM of SWAP containing wtS*j*TGR, IC_50_ 14.82µM of rS*j*TGR-Sec). However, **7ae** did not show an effective inhibitory effect on rS*j*TGR-Sec. As for phenyl derivatives, **7af** (IC_50_ 8.87µM of SWAP containing wtS*j*TGR, IC_50_ 3.23µM of rS*j*TGR-Sec) and **7cf** (IC_50_ 12.51µM of SWAP containing wtS*j*TGR) did not differ significantly in inhibiting SWAP containing wtS*j*TGR, but **7cf** did not show an inhibitory effect on rS*j*TGR-Sec. Among acrylate compounds, only **7bj** showed inhibitory activity against SWAP containing wtS*j*TGR (IC_50_ 5.80µM).

Some amine derivatives (**9aa**, **9ab**, **9bd**, **9be**, **9da**) showed inhibitory activity, among which **9bd** had an inhibitory effect against SWAP containing wtS*j*TGR at the nanomolar level (IC_50_ = 42nM). Compared with phenyl amino derivative **9aa**, **9ba**, **9ca** and **9da**, the pyridine with strong electron-withdrawing ability improved the activity, such as compounds **9aa** (IC_50_ 21.57µM to SWAP containing wtS*j*TGR) and **9da** (IC_50_ 11.62µM to SWAP containing wtS*j*TGR, IC_50_ 3.73µM to rS*j*TGR-Sec) showed better inhibition effects than **9ba** (IC_50_ > 50µM) and **9ca** (IC_50_ > 50µM). Similar to phenyl amino derivatives, 4-trifluoromethoxyphenyl amino derivative **9ab** (IC_50_ 0.62µM to SWAP containing wtS*j*TGR, IC_50_ 1.52µM to rS*j*TGR-Sec) showed better inhibitory effects than **9bb** (IC_50_ > 50µM both to SWAP containing wtS*j*TGR and rS*j*TGR-Sec). However, among piperazine substituted derivatives (**9ac**, **9ad** and **9bd**), **9bd** exhibited good inhibitory activity against SWAP containing wtS*j*TGR (IC_50_ 0.042µM). Interestingly, compound **9be** is an intermediate of a synthetic target molecule, but it displayed much better inhibitory effects (IC_50_ 0.63µM to SWAP containing wtS*j*TGR, IC_50_ 0.22µM to rS*j*TGR-Sec) than furoxan. Perhaps the sulfamine or Si-O-N moieties in molecule **9be** has some influence on enzyme inhibition ability; this result might provide a new direction for the design of additional compounds.

### Docking studies

**Fig. 4:**
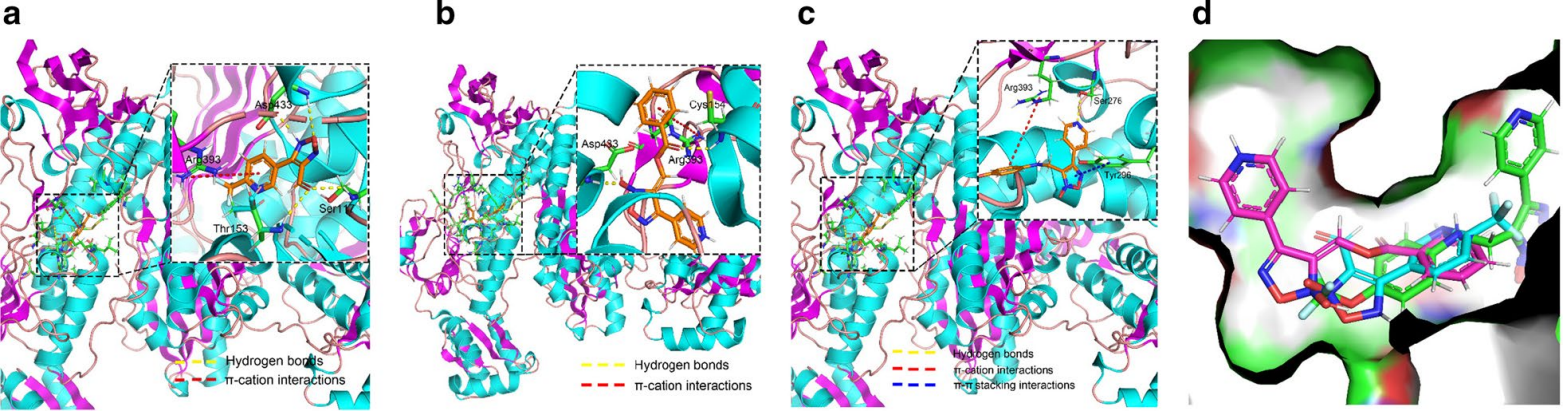
In **a**–**c**, the protein (S*j*TGR) is shown as ribbons, the synthesized inhibitors are shown as orange sticks, and the residues that can interact with inhibitors are shown as green sticks. **a** Compound **6d** forms a *π*-cation interaction (red dashed line) with Arg393 and hydrogen bonds (yellow dashed lines) with Ser117, Thr153 and Asp433. **b** Compound **7af** forms a *π*-cation interaction with Arg393 and hydrogen bonds with Cys154, and Asp433. **c** Compound **9ab** forms a *π*-cation interaction with Arg393, a *π*–*π* stacking interaction (blue dashed line) with Tyr296, and a hydrogen bond with Ser276. **d** Superposition of docking poses of compounds **6d** (blue), **7af** (purple), **9ab** (green) in the binding pocket of S*j*TGR. S*j*TGR, *Schistosoma japonicum* thioredoxin glutathione reductase.

**Fig. 5:**
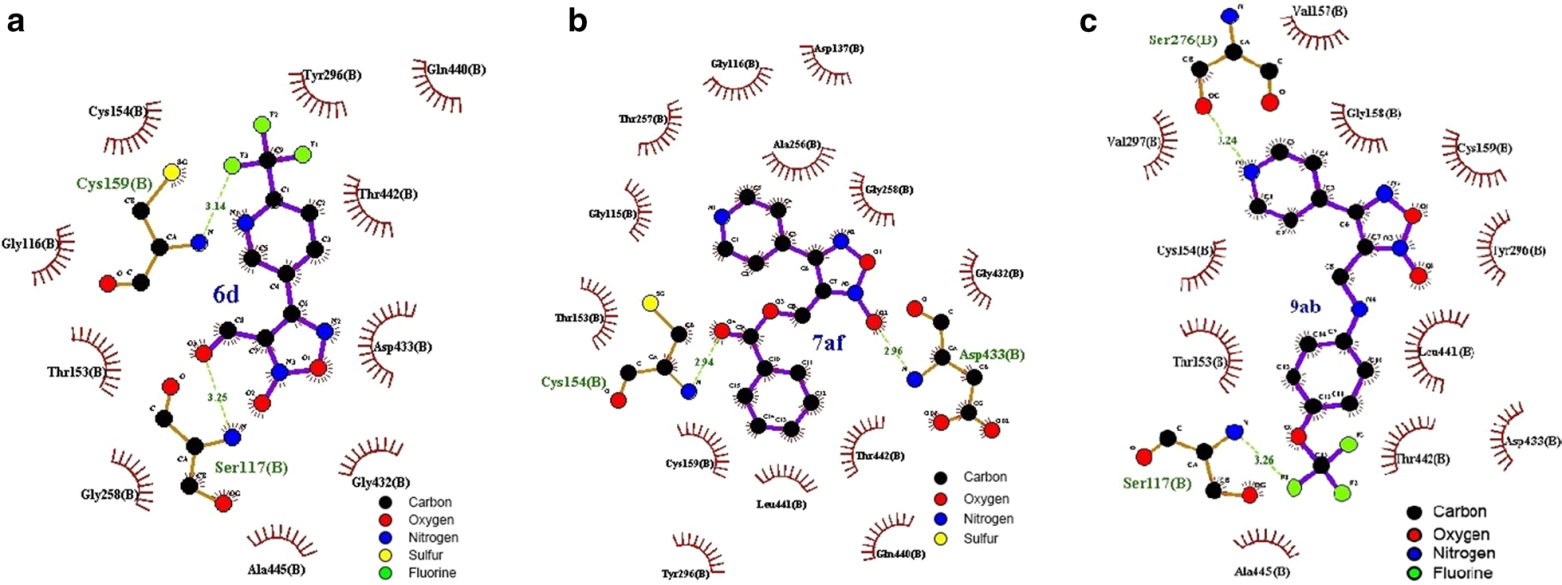
LigPlot^+^ generated two-dimensional schematic overview of molecular interactions between S*j*TGR and compounds **6d**, **7af** and **9ab**. Hydrogen bonds are indicated by green dashed lines with corresponding distances between the atoms given in Å. Hydrophobic contacts are shown by red arcs with spokes radiating toward the atoms involved. S*j*TGR, *Schistosoma japonicum* thioredoxin glutathione reductase.

To rationalize the obtained bioactivity data and to understand how the synthesized inhibitors interact with schistosomal proteins, the selected compounds with high activity **6d**, **7af** and **9ab** were docked to the available crystal structure of S*j*TGR (thioredoxin glutathione reductase from *Schistosoma japonicum*, PDB ID:4LA1). The crystal structure of S*j*TGR is a homodimer, and we retained chain B for docking studies. The results are displayed in Figs. 4 and 5.

Compound **6d** formed four hydrogen bonds with Asp433, Ser117 and Thr153, and a π-cation interaction with Arg393 (Fig. 4a), and two strong hydrogen bonds existed between the hydroxyl moiety of **6d** and S*j*TGR. These results could explain the highest activity of **6d**. Compound **7af** formed two hydrogen bonds with Cys154 and Asp433, and a π-cation interaction with Arg393 (Fig. 4b**)**, while the pyridine ring and oxadiazole ring did not form any type of interactions with the residues of Arg393. Compound **9ab** formed one hydrogen bond with Ser276, a π-cation interaction with Arg393 and a π-π stacking interaction with Tyr296. It was observed that the oxadiazole ring participated in the formation of chemical bonds, to constitute more interaction bonds. The distance between the oxadiazole ring and other ring-shaped conjugated structures should be taken into consideration. After superimposing three compounds (**6d**, **7af** and **9ab**) together (Fig. 4d), each of them formed one π-cation interaction with Arg393; therefore, it was speculated that Arg393 played an anchor role during the binding process.

On the other hand, a hydrophobic effect was shown by red arcs with spokes radiating toward the atoms involved (Fig. 5). The numbers of the residues that had hydrophobic contacts with the inhibitors were negatively associated with enzyme inhibitory activity. Among three compounds (**6d**, **7af** and **9ab**), compound **7af** had the most hydrophobic contacting residues and it had the lowest activity, while compound **6d** had the least hydrophobic contacting residues and it exhibited the highest activity.

## Conclusions

Using furoxan as the leading compound, a series of novel esters and amine derivatives have been synthesized by modifying the cyano moiety into an ester or amine group and replacing phenyl moiety with its bioisosteres pyridine or substituted pyridine. The structure of the key intermediate for target compounds was confirmed by crystallography. The inhibitory activity of all title compounds and key intermediates was evaluated against the TrxR activity of both rS*j*TGR-Sec and SWAP containing wtS*j*TGR for the first time. Several new derivatives, **6a–d**, **9ab**, **9bd** and **9be**, showed better activity toward rS*j*TGR-Sec or SWAP containing wtS*j*TGR than furoxan. The SAR of these novel furoxan derivatives was preliminarily analyzed. A pyridine ring with strong electron-withdrawing ability might be beneficial for enhancing the inhibitory effect. Docking studies showed that hydroxyl moiety can form two strong hydrogen bonds with S*j*TGR, which is of great help in enhancing the inhibitory activity of the compounds. Compound **6d** with trifluoromethyl on the pyridine ring has the highest activity and is a good S*j*TGR inhibitor and could be a potent antischistosomal agent.

## Supplementary Information

**Additional file 1.** The preparation process and spectrum of compounds.

## Data Availability

Data supporting the conclusions of this article are included within the article and its additional file.

## Abbreviations

PZQ: Praziquantel
TGR: Thioredoxin glutathione reductase
S*j*TGR: *Schistosoma japonicum* TGR
S*m*TGR: *Schistosoma mansoni* TGR
rS*j*TGR-Sec: Recombinant *Schistosoma japonicum* TGR containing selenium
SWAP: Soluble worm antigen
wtS*j*TGR: Wild-type S*j*TGR
SAR: Structure–activity relationship
TrxR: Thioredoxin reductase
GR: Glutathione reductase
NADPH: Nicotinamide adenine dinucleotide phosphate
NMR: Nuclear magnetic resonance
HRMS: High-resolution mass spectrometry
SDS-PAGE: Sodium dodecyl sulfate polyacrylamide gel electrophoresis
PBS: Phosphate-buffered saline
DMSO: Dimethyl sulfoxide
EDTA: Ethylene diamine tetraacetic acid
DTNB: Dithionitrobenzoic acid
DIBAL-H: Diisobutylaluminum hydride
DMAP: Dimethylaminopyridine
PMSF: Benzoyl sulfonyl fluoride
