# Supplementary material for: Synthesis of oxadiazole-2-oxide derivatives as potential drug candidates for schistosomiasis targeting SjTGR
Source: Parasit Vectors. 2021 Apr 26;14:225. doi: 10.1186/s13071-021-04634-4 (PMC8074465; doi:10.1186/s13071-021-04634-4)
Supplement: Supplementary file 1 — Additional file 1. The preparation process and spectrum of compounds. [file 13071_2021_4634_MOESM1_ESM.docx]

**Additional file 1: The preparation process and spectrum of compounds**

All chemical reagents involved in synthetic procedure were purchased. All synthesized compounds were verified by ^1^H-NMR, ^13^C-NMR or HRMS.

**Synthesis of Oxadiazole-2-oxide derivatives**

**1. Synthesis of furoxan 2**

Compound **2** was prepared as previously report [1].

***2.* General procedure for the synthesis of ethyl (E)-3-(pyridin-3-yl)acrylates 4a-d.**

To a stirred solution of triethyl phosphonoacetate (28 mmol) in THF (100 ml) was added NaH (28 mmol) at 0℃. The mixture was stirred at the same temperature for 1.5 h. and then it was stirred at room temperature for 1 h. Nicotinaldehyde (18.67 mmol) was added slowly to reaction mixture and the mixture was stirred at room temperature overnight. The reaction was quenched with 30ml brine and the THF was removed under vacuum. The water phase was extracted with ethyl acetate and dried with anhydrous Na_2_SO_4._ Then the mixture was filtered and ethyl acetate was removed under vacuum. The concentrate was purified by chromatography on a silica gel column to give **4a-d.**

*2.1. Characterization data of ethyl (E)-3-(pyridin-4-yl)acrylate* ***4a*** [2].

Yield 81%; white solid; ^1^H-NMR (400 MHz, Chloroform-*d*) δ 8.66 (dd, *J* = 4.6, 1.5 Hz, 2H, pyridine-H), 7.60 (d, *J* = 16.1 Hz, 1H, CH), 7.37 (dd, *J* = 4.6, 1.4 Hz, 2H, pyridine-H), 6.60 (d, *J* = 16.1 Hz, 1H, CH), 4.29 (q, *J* = 7.1 Hz, 2H, CH2), 1.35 (d, *J* =14.3 Hz, 3H, CH3).

*2.2. Characterization data of ethyl (E)-3-(pyridin-3-yl)acrylate* ***4b*** [3].

Yield 94%; yellowish oil; ^1^H NMR (400 MHz, Chloroform-*d*) δ 8.75 (s, 1H, pyridine-H), 8.63~8.58 (m, 1H, pyridine-H), 7.8~7.81 (m, 1H, pyridine-H), 7.67 (d, *J*= 16.1 Hz, 1H, CH), 7.33 (dd, *J* = 7.7, 4.8 Hz, 1H, pyridine-H), 6.52 (d, *J* = 16.1 Hz, 1H, CH), 4.28 (q, *J* = 7.1 Hz, 2H, CH2), 1.35 (t, *J* = 7.1 Hz, 3H, CH3).

*2.3. Characterization data of ethyl (E)-3-(6-methoxypyridin-3-yl)acrylate* ***4c*** [4].

Yield 99%; white solid; ^1^H-NMR (400 MHz, Chloroform-*d*) δ 8.27 (d, *J* = 2.5 Hz, 1H, pyridine-H), 7.77 (dd, *J* = 8.7, 2.5 Hz, 1H, pyridine-H), 7.63 (d, *J* = 16.0 Hz, 1H, pyridine-H), 6.77 (d, *J* = 8.7 Hz, 1H, CH), 6.33 (d, *J* = 16.0 Hz, 1H, CH), 4.26 (q, *J* = 7.1 Hz, 2H, CH2), 3.97 (s, 3H, CH3), 1.34 (t, *J* = 7.1 Hz, 3H, CH3).

*2.4. Characterization data of ethyl (E)-3-(6-(trifluoromethyl)pyridin-3-yl)acrylate* ***4d*** [5].

Yield 82%; white solid; ^1^H NMR (400 MHz, Chloroform-*d*) δ 8.85 (s, 1H, pyridine-H), 7.99 (d, *J* = 8.1 Hz, 1H, pyridine-H), 7.72 (d, *J* = 2.5 Hz, 1H, pyridine-H), 7.69 (d, *J* = 10.6 Hz, 1H, CH), 6.59 (d, *J* = 16.1 Hz, 1H, CH), 4.30 (q, *J* = 7.1 Hz, 2H, CH2), 1.36 (t, *J* = 7.1 Hz, 3H, CH3).

**3. General procedure for the synthesis of (E)-3-(pyridin-3-yl)prop-2-en-1-ols 5a-d.**

To a solution of **4a-d** (14 mmol) in CH_2_Cl_2_ (30 ml) was added dropwise DIBAL-H (1.5M in toluene, 42 mmol) at -78℃ over 50 min. The reaction mixture was stirred at same temperature for 4 h. The reaction was quenched with MeOH and the mixture was stirred for 0.5 additional hour. Then the flask was move to air atmosphere and excess sodium potassium tartrate aqueous solution was added. The mixture was stirred at room temperature for 4 h. The organic layer was separated and the aqueous layer was extracted with ethyl acetate. The combined organic layer was washed with brine and water, dried with anhydrous Na_2_SO_4_, filtered with celite and concentrated under vacuum. The crude product was purified by chromatography on a silica gel column to give **5a-d**.

*3.1. Characterization data of (E)-3-(pyridin-4-yl)prop-2-en-1-ol* ***5a*** [2].

Yield 73%; white solid; ^1^H-NMR (400 MHz, Chloroform-*d*) δ 8.49 (dd, *J* = 4.6, 1.4Hz, 2H, pyridine-H), 7.24 (dd, *J* = 4.7, 1.4 Hz, 2H, pyridine-H), 6.59~6.55 (m, 2H, CH=CH), 4.39 (d, *J* = 2.0 Hz, 2H, CH2).

*3.2. Characterization data of (E)-3-(pyridin-3-yl)prop-2-en-1-ol* ***5b*** [3].

Yield 84%; yellowish oil; ^1^H NMR (400 MHz, Chloroform-d) δ 8.51 (s, 1H, pyridine-H), 8.42~8.35 (m, 1H, pyridine-H), 7.70~7.64 (m, 1H, pyridine-H), 7.22 (dd, *J* = 7.8, 4.9 Hz, 1H, pyridine-H), 6.60 (d, *J* = 16.1 Hz, 1H, CH), 6.43 (dt, *J* = 16.0, 5.0Hz, 1H, CH), 5.24 (s, 1H, OH), 4.35 (d, *J* = 4.9 Hz, 2H, CH2).

*3.3. Characterization data of (E)-3-(6-methoxypyridin-3-yl)prop-2-en-1-ol* ***5c*** [6].

Yield 84%; white soli; ^1^H-NMR (400 MHz, Chloroform-*d*) δ 8.08 (d, *J* = 2.5 Hz, 1H, pyridine-H), 7.63 (dd, *J* = 8.6, 2.5 Hz, 1H, pyridine-H), 6.70 (d, *J* = 8.6 Hz, 1H, pyridine-H), 6.52 (d, *J* = 16.0 Hz, 1H, CH), 6.24 (dt, *J* = 15.9, 5.6 Hz, 1H, CH), 4.30 (t, *J* = 4.6 Hz, 2H, CH2), 3.93 (s, 3H, CH3), 2.51 (s, 1H, OH).

*3.4. Characterization data of (E)-3-(6-(trifluoromethyl)pyridin-3-yl)prop-2-en-1-ol* ***5d*** [5].

Yield 78%;white solid; ^1^H-NMR (400 MHz, Chloroform-*d*) δ 8.67 (s, 1H, pyridine-H), 7.84 (d, *J* = 7.8 Hz, 1H, pyridine-H), 7.62 (d, *J* = 8.2 Hz, 1H, pyridine-H), 6.69 (d, *J* = 16.1 Hz, 1H, CH), 6.55 (dt, *J* = 16.0, 4.8 Hz, 1H, CH), 4.40 (d, *J* = 4.2 Hz, 2H, CH2), 2.77 (s, 1H, OH).

**4. General procedure for synthesis of 3-(hydroxymethyl)-4-pyridin-1,2,5-oxadiazole 2-oxides 6a-d.**

To a solution of (E)-3-pyridinprop-2-en-1-ol (**5a-d,** 1.85 mmol) in AcOH (5 ml) was added sodium nitrite(4.63 mmol) portion wise over 45 min. The reaction was stirred at 60℃ for 10.5 h. The solvent was removed under vacuum and the residue was dissolved in ethyl acetate. The organic layer was washed with brine and water, dried with anhydrous Na_2_SO_4_, filtered and concentrated under vacuum to afford crude product which was purified by chromatography on a silica gel column to give **6a-d**.

*4.1. Characterization data of 3-(hydroxymethyl)-4-(pyridin-4-yl)-1,2,5-oxadiazole 2-oxide* ***6a***

Yield 30%; White solid; m.p: 142-146℃; ^1^H-NMR (400 MHz, Chloroform-*d*) δ 8.84 (d, *J* = 5.9 Hz, 2H, pyridine-H), 7.81 (d, *J* = 6.0 Hz, 2H, pyridine-H), 4.78 (s, 2H, CH2), 3.04 (s, 1H, OH); ^13^C NMR(100MHz, Chloroform-d) δ 53.464, 114.133, 121.840, 134.204, 151.129, 155.045; HRMS: (m/z)[M+H]^+^ calcd for C_8_H_7_N_3_O_3_: 194.0560. Found: 194.0766.

*4.2. Characterization data of* *3-(hydroxymethyl)-4-(pyridin-3-yl)-1,2,5-oxadiazole 2-oxide* ***6b****.*

Yield 31%; white solid; m.p: 113-119℃; ^1^H-NMR (400 MHz, Methanol-*d*4) δ 9.08 (s, 1H, pyridine-H), 8.78~8.70 (m, 1H, pyridine-H), 8.42~8.32 (m, 1H, pyridine-H), 7.64 (dd, *J* = 7.7, 5.1 Hz, 1H, pyridine-H), 4.66 (s, 2H, CH2); ^13^C-NMR (101 MHz, Methanol-*d*4) δ 154.70, 151.02, 147.67, 135.92, 124.20, 123.75, 114.25, 52.17; HRMS: (m/z)[M+H]^+^ calcd for C_8_H_7_N_3_O_3_: 194.0560. Found: 194.0753.

*4.3. Characterization data of 3-(hydroxymethyl)-4-(6-methoxypyridin-3-yl)-1,2,5-oxadi- azole 2-oxide* ***6c****.*

Yield 47%;white solid; m.p: 130-142℃; ^1^H-NMR (400 MHz, Methanol-*d*4) δ 8.69 (d, *J* = 2.3 Hz, 1H, pyridine-H), 8.17 (dd, *J* = 8.7, 2.4 Hz, 1H, pyridine-H), 6.96 (d, *J* = 8.7 Hz, 1H, pyridine-H), 4.63 (s, 2H, CH2), 3.99 (s, 3H, CH3); ^13^C-NMR (101MHz, Methanol-*d*4) δ 165.79, 154.84, 146.38, 137.74, 116.56, 114.26, 111.03, 53.04, 52.15; HRMS: (m/z)[M+H]^+^ calcd for C_9_H_9_N_3_O_4_: 224.0671. Found: 224.0663.

*4.4. Characterization data of 3-(hydroxymethyl)-4-(6-(trifluoromethyl)pyridine-3-yl)-1,2,5-oxadiazole 2-oxide* ***6d****.*

Yield 39%; white solid; m.p: 90-96℃; ^1^H-NMR (400 MHz, Chloroform-*d*) δ 9.25 (s, 1H, pyridine-H), 8.45 (d, *J* = 7.9 Hz, 1H, pyridine-H), 7.89 (d, *J* = 8.1 Hz, 1H, pyridine-H), 4.79 (d, *J* = 3.2 Hz, 2H, CH2), 3.0~2.89 (m, 1H, OH); ^13^C-NMR (101 MHz, Chloroform-*d*) δ 150.15 (q, *J* = 35.5 Hz), 148.69, 137.01, 125.64, 120.99 (q, *J* = 275.6Hz ), 120.97 (m), 113.97, 53.16; HRMS: (m/z)[M+H]^+^ calcd for C_9_H_6_F_3_N_3_O_3_: 262.0440. Found: 262.0429.

**5. General procedure for synthesis of compounds 7.**

To a solution of **6a-d** (0.16 mmol) and DMAP (0.032 mmol) in anhydrous CH_2_Cl_2_ (5 ml) was added triethylamine (dissolved in 1 ml CH_2_Cl_2_, 0.24 mmol) at 0℃. The reaction mixture was stirred for 0.5 h at same temperature. Then acyl chloride (dissolved in 1 ml CH_2_Cl_2_, 0.24 mmol) was added and the reaction mixture was stirred at room temperature. When the reaction was completed, the reaction mixture was washed with brine, water and dried with anhydrous Na_2_SO_4_. The organic phase was filtered and concentrated, the concentrate was purified by chromatography on a silica gel column to give **7**.

*5.1. Characterization data of* *3-(acetoxymethyl)-4-(pyridin-4-yl)-1,2,5-oxadiazole 2-oxide* ***7aa****.*

Yield 67%; yellowish solid; m.p : 62-64℃; ^1^H-NMR (400 MHz, Chloroform-*d*) δ 2.12 (s, 3H, CH3), 5.24 (s, 2H, CH2), 7.72 (m, 2H, pyridine–H), 9.00 (m, 2H,pyridine–H); ^13^C-NMR (101 MHz, Chloroform-*d*) δ 20.532, 54.047, 110.877, 121.704, 133.848, 151.133, 154.799, 169.979; HRMS:(m/z)[M+H]^+^ calcd for C_10_H_9_N_3_O_3_: 236.0666. Found: 236.0765.

*5.2. Characterization data of* *3-((propionyloxy)methyl)-4-(pyridin-4-yl)-1,2,5-oxadiazole 2-oxide* ***7ab****.*

Yield 86%; yellowish solid; m.p : 46-53℃; ^1^H-NMR (400 MHz, Chloroform-*d*) δ 8.85 (d, *J* = 5.4 Hz, 2H, pyridine-H), 7.67 (d, *J* = 5.7 Hz, 2H, pyridine-H), 5.18 (s, 2H, CH2), 2.39 (q, *J* = 7.5 Hz, 2H, CH2), 1.14 (t, *J* = 7.5 Hz, 3H, CH3); ^13^C-NMR (101 MHz, Chloroform-*d*) δ 173.36, 154.55, 151.03, 133.64, 121.37, 110.80, 53.76, 27.07, 8.84; HRMS: (m/z)[M+H]^+^ calcd for C_11_H_11_N_3_O_4_: 250.0822. Found: 250.0823.

*5.3. Characterization data of 3-((butyryloxy)methyl)-4-(pyridin-4-yl)-1,2,5-oxadiazole 2-oxide* ***7ac****.*

Yield 91%; yellowish oil; ^1^H-NMR (400 MHz, Chloroform-*d*) δ 8.85 (s, 2H, pyridine-H), 7.68 (d, *J* = 4.7 Hz, 2H, pyridine-H), 5.18 (s, 2H, CH2), 2.34 (t, *J* = 7.4 Hz, 2H, CH2), 1.65 (h, *J* = 7.2 Hz, 2H, CH2), 0.94 (t, *J* = 7.4 Hz, 3H, CH3); ^13^C-NMR (101 MHz, Chloroform-*d*) δ 172.57, 154.53, 151.00, 133.67, 121.40, 110.81, 53.67, 35.52, 18.20, 13.55; HRMS: (m/z)[M+H]^+^ calcd for C_12_H_13_N_3_O_4_: 264.0979. Found: 264.0975.

*5.4. Characterization data of 3-((pivaloyloxy)methyl)-4-(pyridin-4-yl)-1,2,5-oxadiazole 2-oxide* ***7ad****.*

Yield 89%; yellowish oil; ^1^H-NMR (400 MHz, Chloroform-*d*) δ 8.83 (d, *J* = 4.4 Hz, 2H, pyridine-H), 7.68 (d, *J* = 5.3 Hz, 2H, pyridine-H), 5.17 (s, 2H, CH2), 1.16 (s, 9H, CH3); ^13^C-NMR (101 MHz, Chloroform-*d*) δ 177.42, 154.40, 150.88, 133.80, 121.35, 110.75, 54.10, 38.98, 26.95; HRMS: (m/z)[M+H]^+^ calcd for C_13_H_15_N_3_O_4_: 278.1135. Found: 278.1307.

*5.5. Characterization data of* *3-((2-chloroacetoxy)methyl)-4-(pyridin-4-yl)-1,2,5- oxadiazole 2-oxide* ***7ae****.*

Yield 71%; yellowish solid; m.p : 84-88℃; ^1^H-NMR (400 MHz, Chloroform-*d*) δ 4.11 (s, 2H, CH2-Cl), 5.28 (s, 2H, CH2-O), 7.66 (m, *J* = 4.8Hz, 2H, pyridine–H), 8.86 (m, *J* = 5.2Hz, 2H, pyridine–H); ^13^C-NMR (400 MHz, Chloroform-*d*) δ 40.101, 55.147, 110.116, 121.365, 133.354, 151.116, 154.533, 166.460; HRMS:(m/z)[M+H]^+^ calcd for C_10_H_8_ClN_3_O_4_: 270.0276. Found: 270.0484.

*5.6. Characterization data of 3-((benzoyloxy)methyl)-4-(pyridin-4-yl)-1,2,5-oxadiazole 2-oxide* ***7af****.*

Yield 62%; white solid; m.p : 86-90℃; ^1^H-NMR (400 MHz, Chloroform-*d*) δ 5.42 (s, 2H, CH2), 7.43 (m, *J* = 8Hz, 2H, 3,5-ph), 7.59 (t, *J* = 7.2Hz, 1H, 4-ph), 7.71 (dd, *J* = 1.6Hz, *J* = 4.8Hz, 2H, 3,5-py), 7.96 (dd, *J* = 1.2Hz, *J* = 8Hz, 2H, 2,6-ph), 8.82 (dd, *J* = 1.6Hz, *J* = 4.8Hz, 2H, 2,6-py); ^13^C-NMR(400MHz, Chloroform-*d*) δ 57.241, 110.788, 121.368, 128.203, 128.666, 129.801, 133.616, 133.966, 151.044, 151.829, 165.499; HRMS:(m/z)[M+H]^+^ calcd for C_15_H_11_N_3_O_4_: 298.0822. Found: 298.0958.

*5.7. Characterization data of 3-(((4-fluorobenzoyl)oxy)methyl)-4-(pyridin-4-yl)-1,2,5- oxadiazole 2-oxide* ***7ag****.*

Yield 76%; white solid; m.p : 108-110℃; ^1^H-NMR (400 MHz, Chloroform-*d*) δ 5.42 (s, 2H, CH2), 7.10 (m, *J* = 2.4Hz，*J* = 8.4Hz, 2H, 3,5-ph), 7.70 (m, *J* = 1.6 Hz, *J* = 1.6Hz, 2H, 3,5-py), 7.98 (m, 2H, 2,6-ph), 8.82 (m, *J* = 1.6Hz, *J* = 4.8Hz, 2H, 2,6-py); ^13^C-NMR (400 MHz, Chloroform-*d*) δ 110.788, 121.368, 128.203, 128.666, 129.801, 133.616, 133.966, 151.044, 151.829, 165.499; HRMS:(m/z)[M+H]^+^ calcd for C_15_H_11_N_3_O_4_: 316.0728. Found: 316.0756.

*5.8. Characterization data of 3-(((perfluorobenzoyl)oxy)methyl)-4-(pyridin-4-yl)- 1,2,5-oxadiazole 2-oxide* ***7ah****.*

Yield 83%; yellow solid; m.p : 96-102℃; ^1^H-NMR (400 MHz, Chloroform-*d*) δ 8.84 (s, 2H, pyridine-H), 7.67 (d, *J* = 4.4 Hz, 2H, pyridine-H), 5.45 (s, 2H, CH2); ^13^C-NMR (101 MHz, Chloroform-*d*) δ 158.13, 154.53, 151.08, 147.03 (m), 145.30 (m), 144.44 (m), 142.68 (m), 139.09 (m), 136.50 (m), 133.36, 121.31, 109.86, 106.20 (m), 55.51; HRMS: (m/z)[M+H]^+^ calcd for C_15_H_6_F_5_N_3_O_4_: 388.0351. Found: 388.0483.

*5.9. Characterization data of 3-(((3,5-bis(trifluoromethyl)benzoyl)oxy)methyl)-4-(pyridin-4-yl)-1,2,5-oxadiazole 2-oxide* ***7ai****.*

Yield 83%; white solid; m.p : 134-139℃; ^1^H-NMR (400 MHz, Chloroform-*d*) δ 8.83 (d, *J* = 5.1 Hz, 2H, pyridine-H), 8.39 (s, 2H, benzene-H), 8.09 (s, 1H, benzene-H), 7.68 (d, *J* = 5.7 Hz, 2H, pyridine-H), 5.50 (s, 2H, CH2); ^13^C-NMR (101 MHz, Chloroform-*d*) δ 163.10, 154.45, 151.07, 133.50, 132.56 (q, *J* = 34.5 Hz), 130.48, 129.88 (d, *J* = 3.0 Hz), 127.27 (m), 122.57 (q, *J* = 274.2 Hz), 121.34, 110.31, 55.25; HRMS: (m/z)[M+H]^+^ calcd for C_17_H_9_F_6_N_3_O_4_: 434.0570. Found: 434.0685.

*5.10. Characterization data of* *3-(acetoxymethyl)-4-(pyridin-3-yl)-1,2,5-oxadiazole 2-oxide* ***7ba****.*

Yield 98%; colorless oil; ^1^H-NMR (400 MHz, Chloroform-*d*) δ 8.97 (dd, *J* = 2.3, 0.9 Hz, 1H, pyridine-H), 8.83 (dd, *J* = 4.9, 1.7 Hz, 1H, pyridine-H), 8.10 (ddd, *J* = 8.0, 2.3, 1.7 Hz, 1H, pyridine-H), 7.52 (ddd, *J* = 8.0, 4.9, 0.9 Hz, 1H, pyridine-H), 5.15 (s, 2H, CH2), 2.12 (s, 3H, CH3); ^13^C-NMR (101 MHz, Chloroform-*d*) δ 169.81, 154.29, 152.25, 148.28, 135.06, 124.06, 122.67, 110.92, 53.85, 20.36; HRMS: (m/z)[M+H]^+^ calcd for C_10_H_9_N_3_O_4_: 236.0666. Found: 236.0838.

*5.11. Characterization data of 3-((propionyloxy)methyl)-4-(pyridin-3-yl)-1,2,5-oxadiazole 2-oxide* ***7bb****.*

Yield 90%; white solid; m.p: 42-48℃; ^1^H-NMR (400 MHz, Chloroform-*d*) δ 8.97 (dd, *J* = 2.3, 0.8 Hz, 1H, pyridine-H), 8.82 (dd, *J* = 4.9, 1.6 Hz, 1H, pyridine-H), 8.11 (ddd, *J* = 8.0, 2.3, 1.7 Hz, 1H, pyridine-H), 7.52 (ddd, *J* = 8.0, 4.9, 0.9 Hz, 1H, pyridine-H), 5.16 (s, 2H, CH_2_), 2.38 (q, *J* = 7.5 Hz, 2H, CH_2_), 1.13 (t, *J* = 7.5 Hz, 3H, CH_3_); ^13^C-NMR (101 MHz, Chloroform-*d*) δ 173.28, 154.28, 152.27, 148.32, 134.99, 123.98, 122.62, 110.98, 53.80, 27.03, 8.78; HRMS: (m/z)[M+H]^+^ calcd for C_11_H_11_N_3_O_4_: 250.0822. Found: 250.0961.

*5.12. Characterization data of 3-((butyryloxy)methyl)-4-(pyridin-3-yl)-1,2,5-oxadiazole 2-oxide* ***7bc****.*

Yield 79%; colorless oil; ^1^H-NMR (400 MHz, Chloroform-*d*) δ 8.98 (s, 1H, pyridine-H), 8.82 (d, *J* = 5.0 Hz, 1H, pyridine-H), 8.10 (d, *J* = 7.9 Hz, 1H, pyridine-H), 7.51 (dd, *J* = 8.0, 4.9 Hz, 1H, pyridine-H), 5.16 (s, 2H, CH2), 2.33 (t, *J* = 7.3 Hz, 2H, CH2), 1.64 (h, *J* = 7.0 Hz, 2H, CH2), 0.93 (t, *J* = 7.3 Hz, 3H, CH3); ^13^C-NMR (101 MHz, Chloroform-*d*) δ 172.48, 154.26, 152.29, 148.34, 134.97, 123.98, 122.65, 110.96, 53.71, 35.52, 18.18, 13.50; HRMS: (m/z)[M+H]^+^ calcd for C_12_H_13_N_3_O_4_: 264.0979. Found: 264.0975.

*5.13. Characterization data of 3-((pivaloyloxy)methyl)-4-(pyridin-3-yl)-1,2,5-oxadiazole 2-oxide* ***7bd****.*

Yield 98%; white solid; m.p: 54-61℃; ^1^H-NMR (400 MHz, Chloroform-*d*) δ 8.98 (s, 1H, pyridine-H), 8.82 (d, *J* = 4.8 Hz, 1H, pyridine-H), 8.15~8.10 (m, 1H, pyridine-H), 7.52 (dd, *J* = 8.0, 4.9 Hz, 1H, pyridine-H), 5.16 (s, 2H, CH2), 1.16 (s, 9H, CH3); ^13^C-NMR (101 MHz, Chloroform-*d*) δ 177.47, 154.14, 152.18, 148.15, 135.03, 124.08, 122.74, 110.97, 54.16, 38.96, 26.96; HRMS:(m/z)[M+H]^+^ calcd for C_13_H_15_N_3_O_4_: 278.1135. Found: 278.1178.

*5.14. Characterization data of 3-((2-chloroacetoxy)methyl)-4-(pyridin-3-yl)-1,2,5- oxadiazole 2-oxide* ***7be****.*

Yield 82%; white solid; m.p: 87-101℃; ^1^H-NMR (400 MHz, Chloroform-*d*) δ 8.97 (d, *J* = 2.3 Hz, 1H, pyridine-H), 8.84 (dd, *J* = 5.0, 1.6 Hz, 1H, pyridine-H), 8.12~8.08 (m, 1H, pyridine-H), 7.56~7.50 (m, 1H, pyridine-H), 5.26 (s, 2H, CH2), 4.12 (s, 2H, CH2); ^13^C-NMR (101 MHz, Chloroform-*d*) δ 166.47, 154.25, 152.46, 148.27, 135.06, 124.12, 122.40, 110.33, 55.22, 40.12; HRMS: (m/z)[M+H]^+^ calcd forC_10_H_8_ClN_3_O_4_: 270.0276. Found: 270.0543.

*5.15. Characterization data of* *3-((benzoyloxy)methyl)-4-(pyridin-3-yl)-1,2,5-oxadiazole 2-oxide* ***7bf****.*

Yield 80%; white solid; m.p: 69-76℃; ^1^H-NMR (400 MHz, Chloroform-*d*) δ 9.04 (d, *J* = 2.3 Hz, 1H, pyridine-H), 8.80 (dd, *J* = 4.9, 1.7 Hz, 1H, pyridine-H), 8.14 (dt, *J*= 8.1, 2.0 Hz, 1H, pyridine-H), 8.00~7.95 (m, 2H, benzene-H), 7.60 (t, *J* = 7.4 Hz, 1H, benzene-H), 7.50 (dd, *J* = 8.0, 4.8 Hz, 1H, pyridine-H), 7.44 (t, *J* = 7.8 Hz, 2H, benzene-H), 5.40 (s, 2H, CH2); ^13^C-NMR (101 MHz, Chloroform-*d*) δ 165.51, 154.37, 152.33, 148.35, 135.03, 133.90, 129.83, 128.64, 128.29, 124.05, 122.63, 111.01, 54.42; HRMS: (m/z)[M+H]^+^ calcd for C_15_H_11_N_3_O_4_: 298.0822. Found: 298.1056.

*5.16. Characterization data of 3-(((4-fluorobenzoyl)oxy)methyl)-4-(pyridine-3-yl)-1,2,5 -oxadiazole 2-oxide* ***7bg****.*

Yield 79%; yellowish oil; ^1^H-NMR (400 MHz, Chloroform-*d*) δ 9.02 (s, 1H, pyridine-H), 8.80 (d, *J* = 4.8 Hz, 1H, pyridine-H), 8.13 (dt, *J* = 8.0, 1.9 Hz, 1H, pyridine-H), 7.98 (dd, *J* = 8.7, 5.4 Hz, 2H, benzene-H), 7.50 (dd, *J* = 8.0, 4.8 Hz, 1H, pyridine-H), 7.10 (t, *J* = 8.6 Hz, 2H, benzene-H), 5.39 (s, 2H, CH2); ^13^C-NMR (101 MHz, Chloroform-d) δ 166.23 (d, J = 255.9 Hz), 164.54, 154.26, 152.26, 148.26, 135.08, 132.50 (d, J = 9.5 Hz), 124.57 (d, J = 3.0 Hz), 124.09, 122.68, 115.91 (d, J = 22.2 Hz), 110.91 , 54.51; HRMS: (m/z)[M+H]^+^ calcd for C_15_H_10_FN_3_O_4_: 316.0728. Found: 316.0760.

*5.17. Characterization data of 3-(((perfluorobenzoyl)oxy)methyl)-4-(pyridine-3-yl)-1,2,5- oxadiazole 2-oxide* ***7bh****.*

Yield 88%; white solid; m.p: 75-80℃; ^1^H-NMR (400 MHz, Chloroform-*d*) δ 8.98 (s, 1H, pyridine-H), 8.82 (d, *J* = 4.8 Hz, 1H, pyridine-H), 8.11 (dt, *J* = 8.1, 1.7 Hz, 1H, pyridine-H), 7.53 (dd, *J* = 8.0, 4.7 Hz, 1H, pyridine-H), 5.43 (s, 2H, CH2); ^13^C-NMR (101 MHz, Chloroform-*d*) δ 158.12, 154.24, 152.47, 148.19, 147.05 (m), 145.31 (m), 144.45 (m), 142.65 (m), 139.01 (m), 136.50 (m), 134.99, 124.13, 122.37, 110.05, 106.20 (m), 55.57; HRMS: (m/z)[M+H]^+^ calcd for C_15_H_6_F_5_N_3_O_4_: 388.0351. Found: 388.0538.

*5.18. Characterization data of* *3-(((3,5-bis(trifluoromethyl)benzoyl)oxy)methyl)-4-(pyridin-3-yl)-1,2,5-oxadiazole 2-oxide* ***7bi****.*

Yield 94%; yellowish oil; ^1^H-NMR (400 MHz, Chloroform-*d*) δ 8.99 (s, 1H, pyridine-H), 8.83 (d, *J* = 4.4 Hz, 1H, pyridine-H), 8.39 (s, 2H, benzene-H), 8.17~8.12 (m, 1H, pyridine-H), 8.11 (s, 1H, benzene-H), 7.54 (dd, *J* = 7.7, 4.9 Hz, 1H, pyridine-H), 5.50 (s, 2H, CH2); ^13^C-NMR (101 MHz, Chloroform-*d*) δ 163.10, 154.17, 152.50, 148.21, 135.02, 132.49 (q, *J* = 34.4 Hz), 130.49, 129.92 (d, *J* = 2.9 Hz), 127.24 (m), 124.14, 122.58 (q, *J* = 274.2 Hz), 122.46, 110.51, 55.37; HRMS: (m/z)[M+H]^+^ calcd for C_17_H_9_F_6_N_3_O_4_: 434.0570. Found: 434.0686.

*5.19. Characterization data of* *3-((acryloyloxy)methyl)-4-(pyridin-3-yl)-1,2,5-oxadiazole 2-oxide* ***7bj****.*

Yield 22%; yellowish oil; ^1^H-NMR (400 MHz, Chloroform-*d*) δ 8.99 (d, *J* = 2.3 Hz, 1H, pyridine-H), 8.82 (dd, *J* = 4.9, 1.6 Hz, 1H, pyridine-H), 8.14~8.08 (m, 1H, pyridine-H), 7.51 (dd, *J* = 8.0, 4.9 Hz, 1H, pyridine-H), 6.46 (d, *J* = 17.3 Hz, 1H, CH2-H), 6.12 (dd, *J* = 17.3, 10.5 Hz, 1H, CH), 5.94 (d, *J* = 10.5 Hz, 1H, CH2-H), 5.24 (s, 2H, CH2); ^13^C-NMR (101 MHz, Chloroform-*d*) δ 164.93, 154.32, 152.32, 148.32, 135.05, 133.30, 126.58, 124.07, 122.60, 110.87, 53.98; HRMS:(m/z)[M+H]^+^ calcd for C_11_H_9_N_3_O_4_: 248.0666. Found: 248.0769.

*5.20. Characterization data of* *4-(pyridin-3-yl)-3-(((thiophene-2-carbonyl)oxy)methyl) -1,2,5-oxadiazole 2-oxide* ***7bk****.*

Yield 91%; colorless oil; ^1^H-NMR (400 MHz, Chloroform-*d*) δ 9.04 (d, *J* = 2.3 Hz, 1H, pyridine-H), 8.80 (dd, *J* = 4.9, 1.7 Hz, 1H, pyridine-H), 8.14 (dt, *J* = 8.1, 2.0 Hz, 1H, pyridine-H), 7.79 (d, *J* = 3.6 Hz, 1H, thiophene-H), 7.62 (d, *J* = 4.8 Hz, 1H, thiophene-H), 7.50 (dd, *J* = 8.0, 4.8 Hz, 1H, pyridine-H), 7.11 (t, *J* = 4.3 Hz, 1H, thiophene-H), 5.37 (s, 2H, CH2); ^13^C-NMR (101 MHz, Chloroform-d) δ 160.92, 154.38, 152.29, 148.39, 135.07, 134.78, 133.88, 131.35, 128.11, 124.01, 122.61, 110.86, 54.43; HRMS: (m/z)[M+H]^+^ calcd for C_13_H_9_N_3_O_4_S: 304.0387. Found: 304.0391.

*5.21. Characterization data of 3-(acetoxymethyl)-4-(6-methoxypyridin-3-yl)-1,2,5- oxadiazole 2-oxide* ***7ca****.*

Yield 89%; white solid; m.p : 67-75℃; ^1^H-NMR (400 MHz, Chloroform-*d*) δ 8.51 (d, *J* = 2.3 Hz, 1H, pyridine-H), 7.97 (dd, *J* = 8.7, 2.5 Hz, 1H, pyridine-H), 6.92 (d, *J* =8.7 Hz, 1H, pyridine-H), 5.13 (s, 2H, CH2), 4.02 (s, 3H, CH3), 2.13 (s, 3H, CH3); ^13^C-NMR (101 MHz, Chloroform-d) δ 169.86, 165.88, 154.36, 146.38, 137.34, 115.73, 111.98, 110.91, 53.99, 20.42； HRMS: (m/z)[M+H]^+^ calcd for C_11_H_11_N_3_O_5_: 266.0771. Found: 266.0772.

*5.22. Characterization data of 3-((benzoyloxy)methyl)-4-(6-methoxypyridin-3-yl)-1,2,5- oxadiazole 2-oxide* ***7cf****.*

Yield 94%; white solid; m.p : 62-69℃; ^1^H-NMR (400 MHz, Chloroform-*d*) δ 8.59~8.56 (m, 1H, pyridine-H), 8.03~7.96 (m, 3H, pyridine-H, benzene-H), 7.60 (t, *J* =7.4 Hz, 1H, benzene-H), 7.44 (t, *J* = 7.7 Hz, 2H, benzene-H), 6.89 (d, *J* = 8.7 Hz, 1H, pyridine-H), 5.38 (s, 2H, CH2), 3.99 (s, 3H, CH3); ^13^C-NMR (101 MHz, Chloroform-*d*) δ 165.89, 165.55, 154.41, 146.45, 137.34, 133.78, 129.86, 128.58, 128.49, 115.78, 111.97, 110.96, 54.58, 53.97; HRMS: (m/z)[M+H]^+^ calcd for C_16_H_13_N_3_O_5_: 328.0928. Found: 328.0931.

*5.23. Characterization data of 3-(acetoxymethyl)-4-(6-(trifluoromethyl)pyridin-3-yl)- 1,2,5-oxadiazole 2-oxide* ***7da****.*

Yield 96%; yellowish solid; m.p: 73-78℃; ^1^H-NMR (400 MHz, Chloroform-*d*) δ 9.12 (s, 1H, pyridine-H), 8.36 (d, *J* = 7.7 Hz, 1H, pyridine-H), 7.92 (d, *J* = 8.0 Hz, 1H, pyridine-H), 5.16 (s, 2H, CH2), 2.13 (s, 3H, CH3); ^13^C-NMR (101 MHz, Chloroform-d) δ 169.79, 153.18, 150.34 (q, *J* = 35.6 Hz), 148.47, 136.71, 125.41, 120.97 (q, *J* =275.8 Hz), 120.92 (m), 110.71, 53.70, 20.29; HRMS: (m/z)[M+H]^+^ calcd for C_11_H_8_F_3_N_3_O_4_: 304.0540. Found: 304.0545.

*5.24. Characterization data of* *3-((benzoyloxy)methyl)-4-(6-(trifluoromethyl)pyridin-3 -yl)-1,2,5-oxadiazole 2-oxide* ***7df****.*

Yield 98%; yellowish solid; m.p :102-107℃; ^1^H-NMR (400 MHz, Chloroform-*d*) δ 9.17 (s, 1H, pyridine-H), 8.37 (d, *J* = 8.2 Hz, 1H, pyridine-H), 8.05~7.75 (m, 3H, pyridine-H, benzene-H), 7.66~7.54 (m, 1H, benzene-H), 7.52~7.35 (m, 2H, benzene-H), 5.41 (s, 2H, CH2); ^13^C-NMR (101 MHz, Chloroform-*d*) δ 165.48, 153.17, 150.38 (q, *J* = 35.7 Hz), 148.55, 136.73, 134.03, 129.80, 128.68, 128.09, 125.45, 120.96 (q, *J*= 275.6 Hz), 120.90 (m), 110.80, 54.31; HRMS: (m/z)[M+H]^+^ calcd for C_16_H_10_F_3_N_3_O_4_: 366.0696. Found: 366.0693.

**6. General procedure for synthesis of compounds 8a-d.**

To a solution of **6a-d** (0.16 mmol) and DMAP (0.032 mmol) in anhydrous CH_2_Cl_2_ (5 ml) was added triethylamine (dissolved in CH_2_Cl_2_, 0.24 mmol) at 0℃. The reaction mixture was stirred for 0.5 h at same temperature. Then Benzene sulfonyl chloride (dissolved in CH_2_Cl_2_, 0.24 mmol) was added and the reaction mixture was stirred at room temperature. When the reaction was completed, the reaction mixture was washed with brine, water and dried with anhydrous Na_2_SO_4_. The organic phase was filtered and concentrated, the concentrate was purified by chromatography on a silica gel column gave **8a-d**.

**7. General procedure for synthesis of compounds 9.**

A mixture of fresh **8a-d** (0.15 mmol), amine (0.225 mmol), sodium iodide (0.03 mmol) and potassium carbonate (0.225 mmol) in acetonitrile (5 ml) was stirred at room temperature overnight. The solvent was removed under vacuum and the residue was dissolve in ethyl acetate. The organic phase was washed with brine, water and dried with anhydrous Na_2_SO_4_, filtered and concentrated, the concentrate was purified by chromatography on a silica gel column to give **9.**

*7.1 Characterization data of 3-((phenylamino)methyl)-4-(pyridin-4-yl)-1,2,5-oxad-iazole 2-oxide* ***9aa****.*

Yield 65%; white solid; m.p : 93-104℃; ^1^H-NMR (400 MHz, Chloroform-*d*) δ 8.82 (d, *J* = 5.0 Hz, 2H, pyridine-H), 7.63 (d, *J* = 5.0 Hz, 2H, pyridine-H), 7.18 (t, *J* = 7.7 Hz, 2H, benzene-H), 6.82 (t, *J* = 7.4 Hz, 1H, benzene-H), 6.57 (d, *J* = 7.9 Hz, 2H, benzene-H), 4.46 (s, 2H, CH2), 4.12 (s, 1H, NH); ^13^C-NMR (101 MHz, Chloroform-d) δ 154.83, 150.97, 145.51, 134.09, 129.58, 121.67, 119.84, 113.72, 113.12, 37.44; HRMS: (m/z)[M+H]^+^ calcd for C_14_H_12_N_4_O_2_: 269.1033. Found: 269.1032.

*7.2. Characterization data of 4-(pyridin-4-yl)-3-(((4-(trifluoromethoxy)phenyl)amino) methyl)-1,2,5-oxadiazole 2-oxide* ***9ab****.*

Yield 47%; white solid; m.p : 99-108℃; ^1^H-NMR (400 MHz, Chloroform-*d*) δ 8.83 (d, *J* = 5.6 Hz, 2H, pyridine-H), 7.61 (d, *J* = 5.8 Hz, 2H, pyridine-H), 7.03 (d, *J* = 8.5 Hz, 2H, benzene-H), 6.54 (d, *J* = 8.9 Hz, 2H, benzene-H), 4.45 (d, *J* = 6.4 Hz, 2H, CH2), 4.25 (t, *J* = 5.8 Hz, 1H, NH); ^13^C-NMR (101 MHz, Chloroform-*d*) δ 154.70, 150.99, 144.30, 142.10, 133.95, 122.65, 121.58, 120.52 (q, *J* = 257.1 Hz), 114.17, 37.56; HRMS: (m/z)[M+H]^+^ calcd for C_15_H_11_F_3_N_4_O_3_: 353.0856. Found: 353.0858.

*7.3. Characterization data of 3-((4-(4-fluorophenyl)piperazin-1-yl)methyl)-4-(pyridin-4-yl)-1,2,5-oxadiazole 2-oxide* ***9ac****.*

Yield 79%; white solid; m.p : 128-137℃; ^1^H-NMR (400 MHz, Chloroform-*d*) δ 8.82 (d, *J* = 4.3 Hz, 2H, pyridine-H), 7.96 (d, *J* = 5.1 Hz, 2H, pyridine-H), 6.97 (t, *J* = 8.6 Hz, 2H, benzene-H), 6.92~6.83 (m, 2H, benzene-H), 3.63 (s, 2H, CH2), 3.14 (t, *J* = 4.7 Hz 4H, CH2), 2.74 (t, *J* = 4.8 Hz, 4H, CH2); ^13^C-NMR (101 MHz, Chloroform-*d*) δ 158.52, 156.14, 155.58, 150.83, 147.51, 134.36, 121.88, 118.05 (d, *J* = 7.7 Hz), 115.59 (d, *J* = 22.1 Hz), 112.03, 52.88, 50.05; HRMS: (m/z)[M+H]^+^ calcd for C_18_H_18_FN_5_O_2_: 356.1517. Found: 356.1514.

*7.4. Characterization data of 4-(pyridin-4-yl)-3-((4-(3-(trifluoromethyl)phenyl)piperazin-1-yl)methyl)-1,2,5-oxadiazole 2-oxide* ***9ad****.*

Yield 72%; white solid; m.p : 89-103℃; ^1^H-NMR (400 MHz, Chloroform-*d*) δ 8.83 (d, *J* = 5.1 Hz, 2H, pyridine-H), 7.96 (d, *J* = 5.0 Hz, 2H, pyridine-H), 7.36 (t, *J* = 8.1 Hz, 1H, benzene-H), 7.16~7.03 (m, 3H, benzene-H), 3.64 (s, 2H, CH2), 3.27 (t, *J* = 4.8 Hz, 4H, CH2), 2.75 (t, *J* = 4.8 Hz, 4H, CH2); ^13^C-NMR (101 MHz, Chloroform-*d*) δ 155.56, 150.97, 150.84, 134.31, 131.39 (q, *J* = 31.7 Hz), 129.62, 124.20 (q, *J* = 273.7 Hz), 121.84, 119.07, 116.48-116.24 (m), 112.46-112.23 (m), 111.98, 52.65, 50.02, 48.60; HRMS: (m/z)[M+H]^+^ calcd for C_19_H_18_F_3_N_5_O_2_: 406.1485. Found: 406.1487.

*7.5. Characterization data of 3-((phenylamino)methyl)-4-(pyridin-3-yl)-1,2,5-oxadiazole 2-oxide* ***9ba****.*

Yield 67%;white solid; m.p : 142-147℃; ^1^H-NMR (400 MHz, Chloroform-*d*) δ 8.97 (s, 1H, pyridine-H), 8.80 (d, *J* = 4.7 Hz, 1H, pyridine-H), 8.03 (dt, *J* = 8.1, 1.8 Hz, 1H, pyridine-H), 7.47 (dd, *J* = 7.9, 4.8 Hz, 1H, pyridine-H), 7.15 (t, *J* = 7.8 Hz, 2H, benzene-H), 6.79 (t, *J* = 7.3 Hz, 1H, benzene-H), 6.54 (d, *J* = 8.2 Hz, 2H, benzene-H), 4.45 (s, 2H, CH2); ^13^C-NMR (101 MHz, Chloroform-*d*) δ 154.47, 152.00, 148.33, 145.58, 135.36, 129.51, 124.04, 123.07, 119.65, 113.66, 113.37, 37.50; HRMS: (m/z)[M+H]^+^ calcd for C_14_H_12_N_4_O_2_: 269.1033. Found: 269.1040.

*7.6. Characterization data of 4-(pyridin-3-yl)-3-(((4-(trifluoromethoxy)phenyl)amino)methyl)-1,2,5-oxadiazole 2-oxide* ***9bb****.*

Yield 25%; white solid; m.p : 121-128℃; ^1^H-NMR (400 MHz, DMSO-*d*6) δ 8.98 (d, *J* = 2.3 Hz, 1H, pyridine-H), 8.79 (dd, *J* = 4.9, 1.6 Hz, 1H, pyridine-H), 8.21 (dt, *J* = 8.0, 2.0 Hz, 1H, pyridine-H), 7.60 (dd, *J* = 8.0, 4.8 Hz, 1H, pyridine-H), 7.08 (d, *J* = 8.5 Hz, 2H, benzene-H), 6.58 (d, *J* = 8.9 Hz, 2H, benzene-H), 6.53 (t, *J* = 5.5 Hz, 1H, NH), 4.44 (d, *J* = 4.5 Hz, 2H, CH2); ^13^C-NMR (101 MHz, DMSO-*d*6) δ 155.69, 152.32, 148.53, 146.80, 139.88, 135.88, 124.46, 122.91, 122.61,120.71 (q, *J* = 255.4 Hz), 114.35, 113.24, 37.17; HRMS: (m/z)[M+H]^+^ calcd for C_15_H_11_F_3_N_4_O_3_: 353.0856. Found: 353.0874.

*7.7. Characterization data of 3-((4-(4-fluorophenyl)piperazin-1-yl)methyl)-4- (pyridin-3-yl)-1,2,5-oxadiazole 2-oxide* ***9bc****.*

Yield 77%; white solid; m.p :103-109℃; ^1^H-NMR (400 MHz, Chloroform-*d*) δ 9.22 (s, 1H, pyridine-H), 8.77 (d, *J* = 4.8 Hz, 1H, pyridine-H), 8.32 (d, *J* = 8.0 Hz, 1H, pyridine-H), 7.46 (dd, *J* = 8.1, 4.8 Hz, 1H, pyridine-H), 6.93 (t, *J* = 8.5 Hz, 2H, benzene-H), 6.88~6.79 (m, 2H, benzene-H), 3.61 (s, 2H, CH2), 3.10 (t, *J* = 4.8 Hz, 4H, CH2), 2.70 (t, *J* = 4.8 Hz, 4H, CH2); ^13^C-NMR (101 MHz, Chloroform-*d*) δ 158.46, 156.08, 155.20, 152.01, 149.01, 147.57, 135.34, 123.83, 123.33, 118.01 (d, *J* = 7.6 Hz), 115.53 (d, *J* = 22.1 Hz), 112.18, 52.86, 50.06, 50.00; HRMS:(m/z)[M+H]^+^ calcd for C_18_H_18_FN_5_O_2_: 356.1517. Found: 356.1544.

*7.8. Characterization data of 4-(pyridin-3-yl)-3-((4-(3-(trifluoromethyl)phenyl) piperazin-1-yl)methyl)-1,2,5-oxadiazole 2-oxide* ***9bd****.*

Yield 73%; white solid; m.p : 112-117℃; ^1^H-NMR (400 MHz, Chloroform-*d*) δ 9.23 (s, 1H, pyridine-H), 8.78 (d, *J* = 4.0 Hz, 1H, pyridine-H), 8.33 (dt, *J* = 7.9, 1.9 Hz, 1H, pyridine-H), 7.48 (dd, *J* = 8.0, 4.7 Hz, 1H, pyridine-H), 7.34 (t, *J* = 8.1 Hz, 1H, benzene-H), 7.11~7.01 (m, 3H, benzene-H), 3.63 (s, 2H, CH2), 3.23 (t, *J* = 4.9 Hz, 4H, CH2), 2.72 (t, *J* = 4.9 Hz 4H, CH2); ^13^C-NMR (101 MHz, Chloroform-*d*) δ 155.17, 152.05, 151.02, 148.98, 135.32, 131.40 (q, *J* = 31.8 Hz), 129.59, 124.23 (q, *J* = 273.6 Hz), 123.85, 123.30, 119.01, 116.22 (q, *J* = 3.5 Hz), 112.33 (q, *J* = 3.6 Hz), 112.11, 52.65, 50.05, 48.57; HRMS: (m/z)[M+H]^+^ calcd for C_19_H_18_F_3_N_5_O_2_: 406.1485. Found: 406.1509.

7.9. *Characterization data of* 3-(((N-((tert-butyldimethylsilyl)oxy)-4-methylphenyl)sulfonamido)methyl)-4-(pyridin-3-yl)-1,2,5-oxadiazole 2-oxide ***9be***

Yield 46%; white solid; m.p : 132-137℃; ^1^H-NMR (400 MHz, Chloroform-*d*) δ 9.17 (s, 1H, pyridine-H), 8.82 (d, *J* = 4.9 Hz, 1H, pyridine-H), 8.31 (d, *J* = 7.9 Hz, 1H, pyridine-H), 7.72 (d, *J* = 7.9 Hz, 2H, benzene-H), 7.54 (dd, *J* = 8.0, 4.8 Hz, 1H, pyridine-H), 7.39 (d, *J* = 7.9 Hz, 2H, benzene-H), 4.13 (s, 2H, CH_2_), 2.48 (s, 3H, CH_3_), 0.86 (s, 8H, CH_3_), 0.00 (s, 6H, CH_3_); ^13^C-NMR (101 MHz, Chloroform-*d*) δ 159.51, 156.99, 153.23, 150.86, 140.31, 134.86, 134.66, 132.72, 128.80, 127.26, 114.97, 53.29, 30.56, 26.59, 22.56, 0.00; HRMS: (m/z)[M+H]^+^ calcd for C_21_H_28_N_4_O_5_SSi: 477.1622. Found: 477.1617.

*7.10. Characterization data of 4-(6-methoxypyridin-3-yl)-3-((phenylamino)methyl)- 1,2,5-oxadiazole 2-oxide* ***9ca****.*

Yield 77%; yellowish solid; m.p : 86-91℃; ^1^H-NMR (400 MHz, Chloroform-*d*) δ 8.50 (d, *J* = 2.5 Hz, 1H, pyridine-H), 7.89 (dd, *J* = 8.7, 2.5 Hz, 1H, pyridine-H), 7.15 (t, *J* = 7.8 Hz, 2H, benzene-H), 6.87 (d, *J* = 8.7 Hz, 1H, pyridine-H), 6.78 (t, *J* = 7.4 Hz, 1H, benzene-H), 6.56 (d, *J* = 8.0 Hz, 2H, benzene-H), 4.39 (s, 2H, CH2), 4.00 (s, 3H, CH3); ^13^C-NMR (101 MHz, Chloroform-*d*) δ 165.79, 154.57, 146.48, 145.80, 137.61, 129.48, 119.53, 116.0, 113.67, 113.40, 111.88, 54.02, 37.44; HRMS: (m/z)[M+H]^+^ calcd for C_15_H_14_N_4_O_3_: 299.1139. Found: 299.1145.

*7.11. Characterization data of 3-((phenylamino)methyl)-4-(6-(trifluoromethyl)pyridin-3-yl)-1,2,5-oxadiazole 2-oxide* ***9da****.*

Yield 53%; yellowish solid; m.p : 111-116℃; ^1^H-NMR (400 MHz, Chloroform-*d*) δ 9.10 (s, 1H, pyridine-H), 8.24 (d, *J* = 7.8 Hz, 1H, pyridine-H), 7.83 (d, *J* = 8.1 Hz, 1H, pyridine-H), 7.18 (t, *J* = 7.7 Hz, 2H, benzene-H), 6.83 (t, *J* = 7.4 Hz, 1H, benzeneH), 6.56 (d, *J* = 7.9 Hz, 2H, benzene-H), 4.46 (d, *J* = 6.2 Hz, 2H, CH2), 4.12 (t, *J* = 6.1 Hz, 1H, NH); ^13^C-NMR (101 MHz, Chloroform-*d*) δ 153.49, 150.11 (q, *J* = 35.5 Hz), 148.64, 145.29, 137.00, 129.61, 125.70, 120.99 (q, *J* = 275.8 Hz), 120.93-120.73 (m), 120.02, 113.72, 113.11, 37.72; HRMS: (m/z)[M+H]^+^ calcd for C_15_H_11_F_3_N_4_O_2_: 337.0907. Found: 337.0907.

**Spectra of Oxadiazole-2-oxide derivatives**

**1. ^1^H-NMR spectra of compounds 4a-d and 5a-d.**


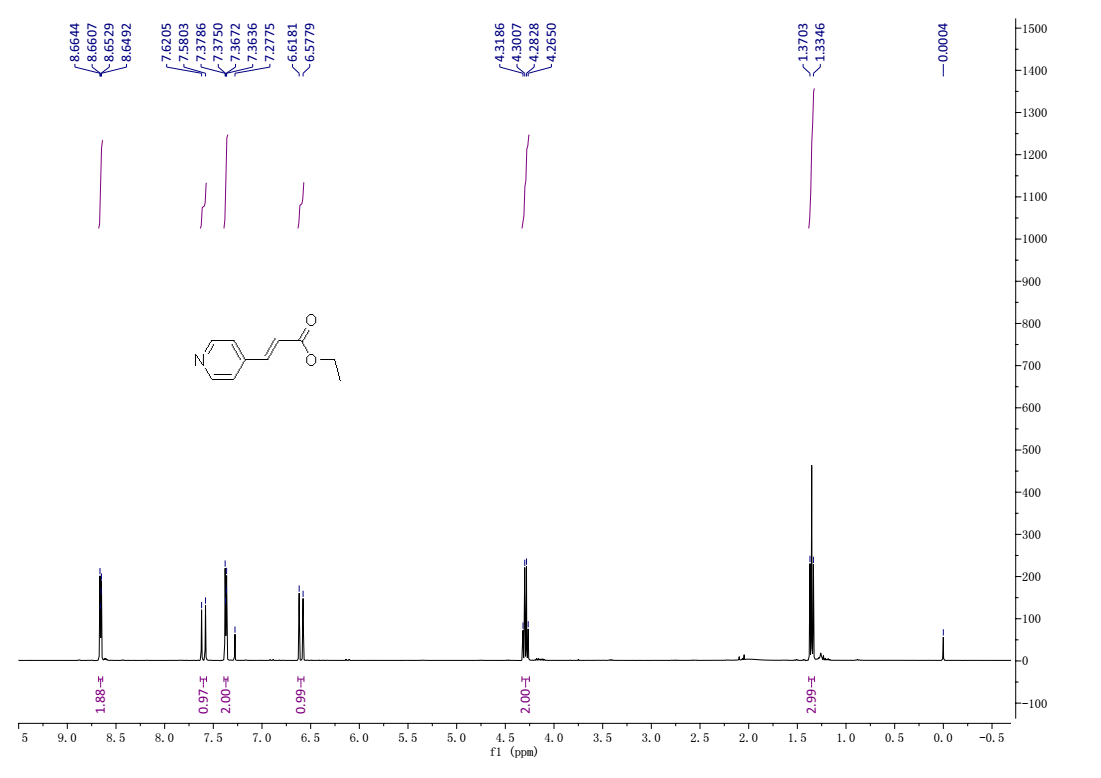


^1^H-NMR (400 MHz, Chloroform-*d*) spectrum of **4a**.


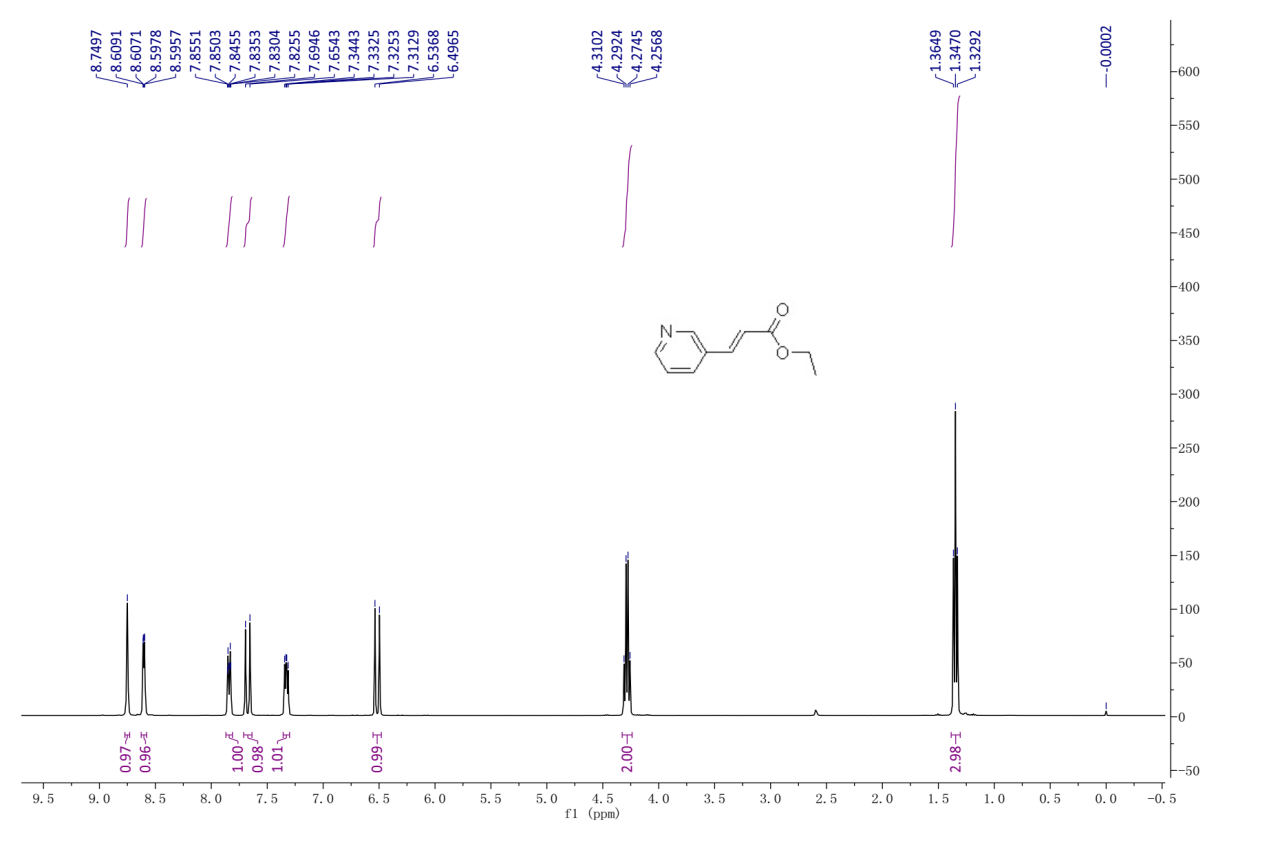


^1^H-NMR (400 MHz, Chloroform-*d*) spectrum of **4b**.


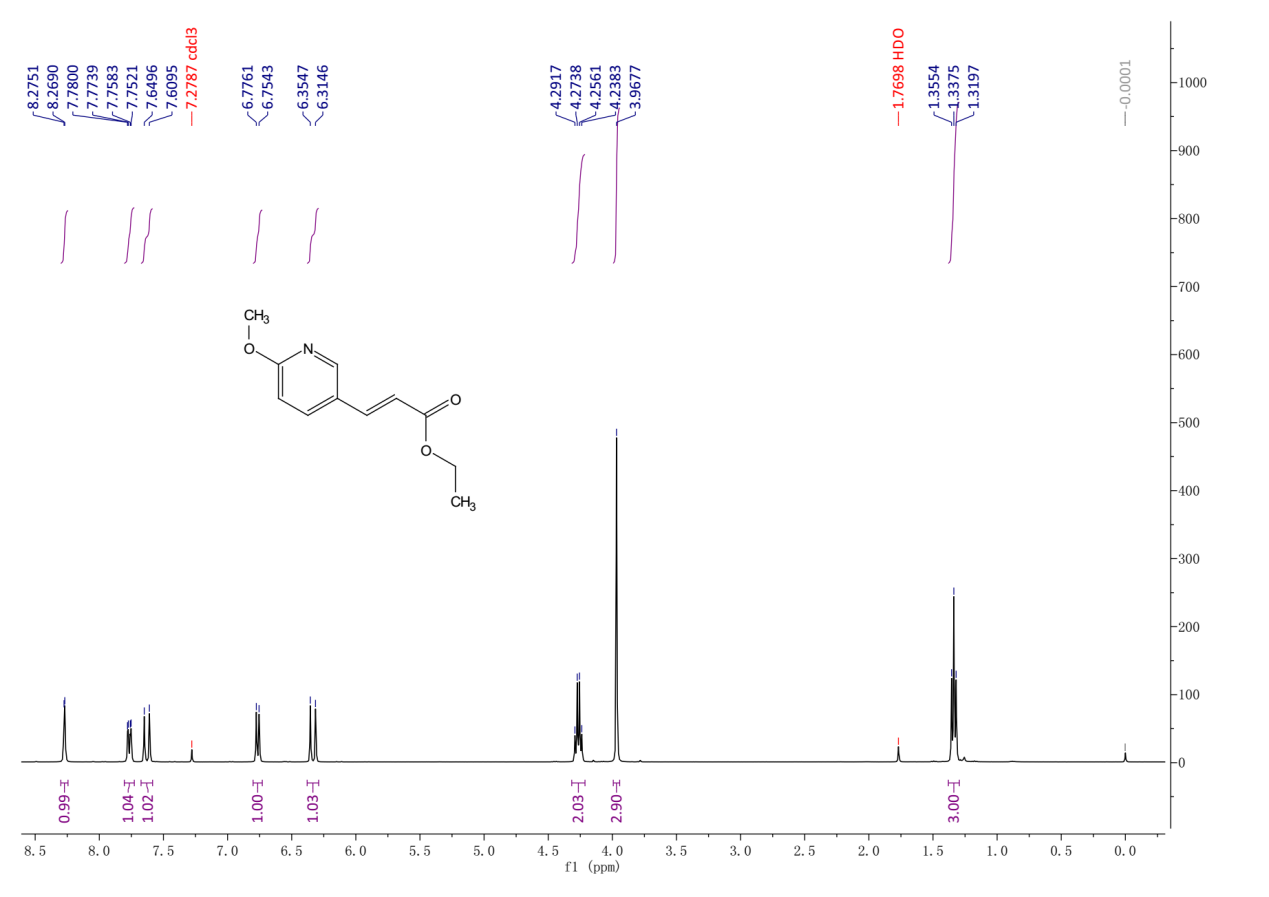


^1^H-NMR (400 MHz, Chloroform-*d*) spectrum of **4c**.


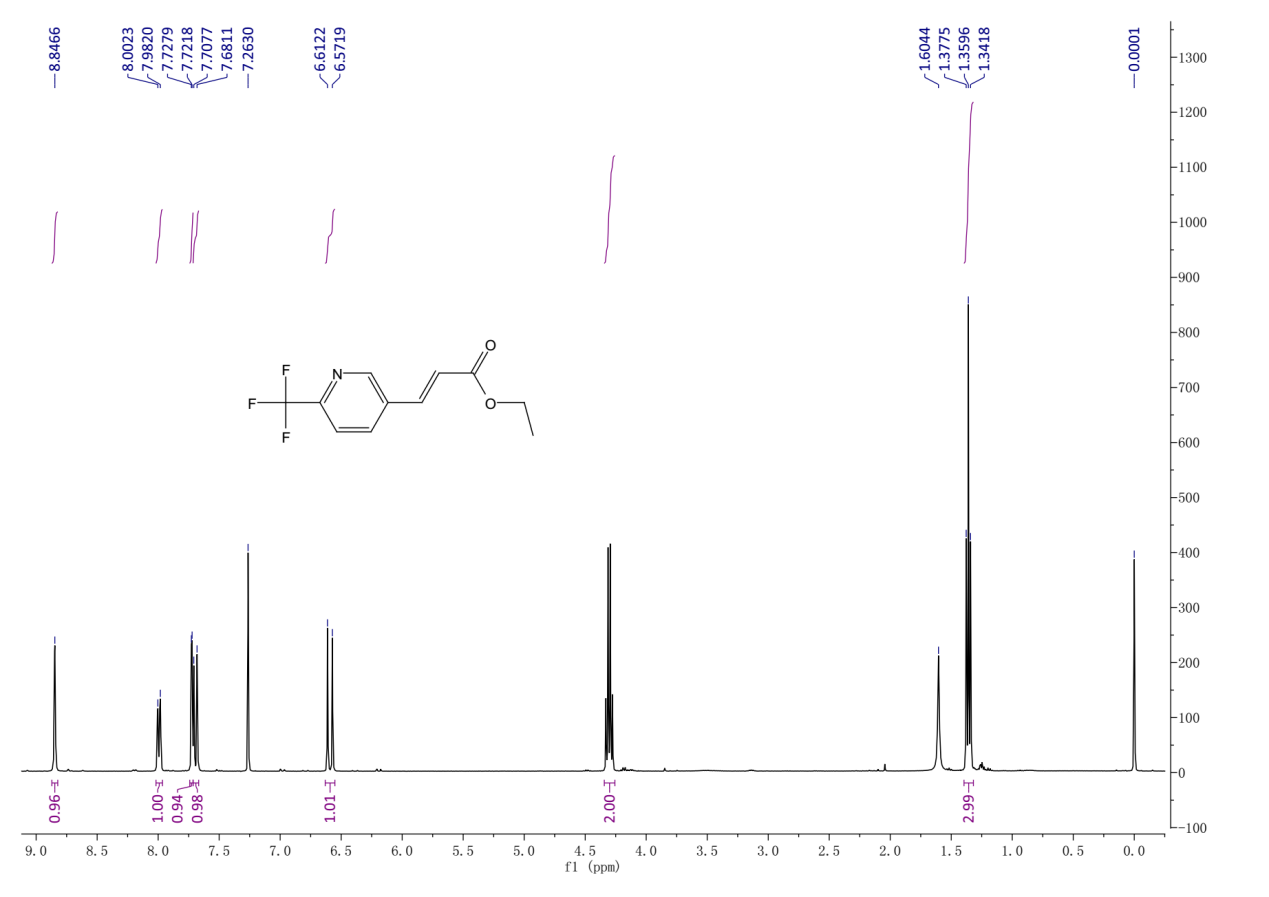


^1^H-NMR (400 MHz, Chloroform-*d*) spectrum of **4d**.


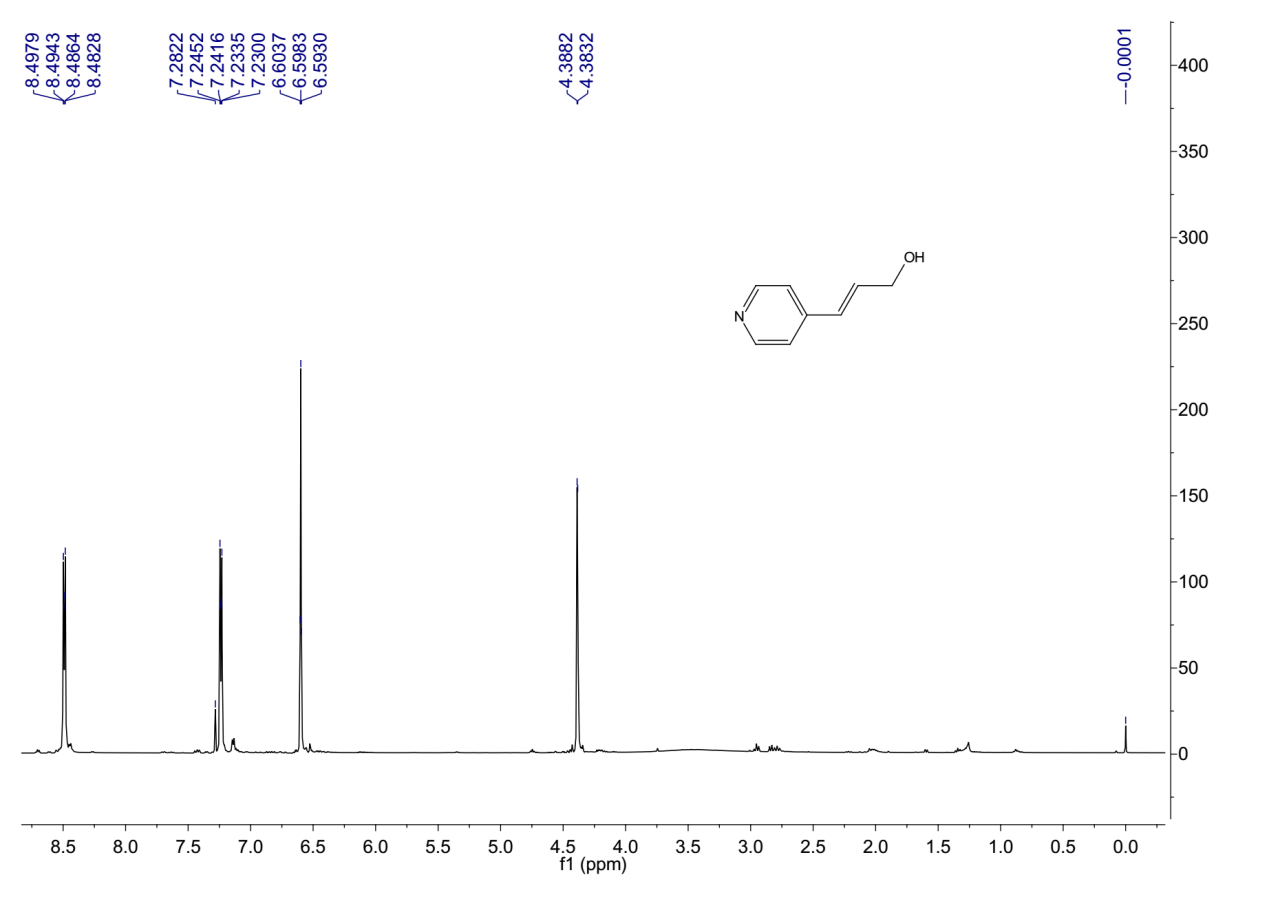


^1^H-NMR (400 MHz, Chloroform-*d*) spectrum of **5a**.


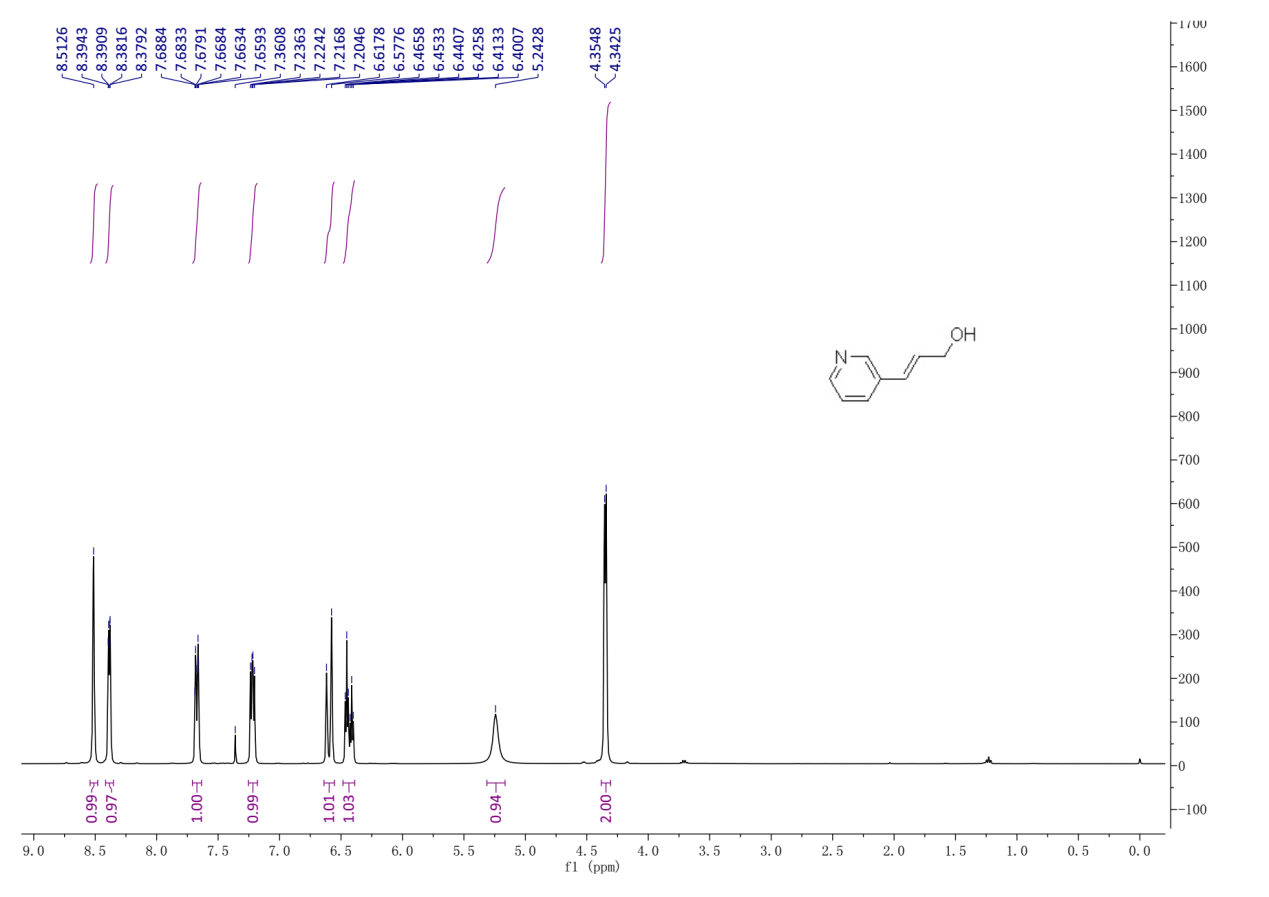


^1^H-NMR (400 MHz, Chloroform-*d*) spectrum of **5b**.


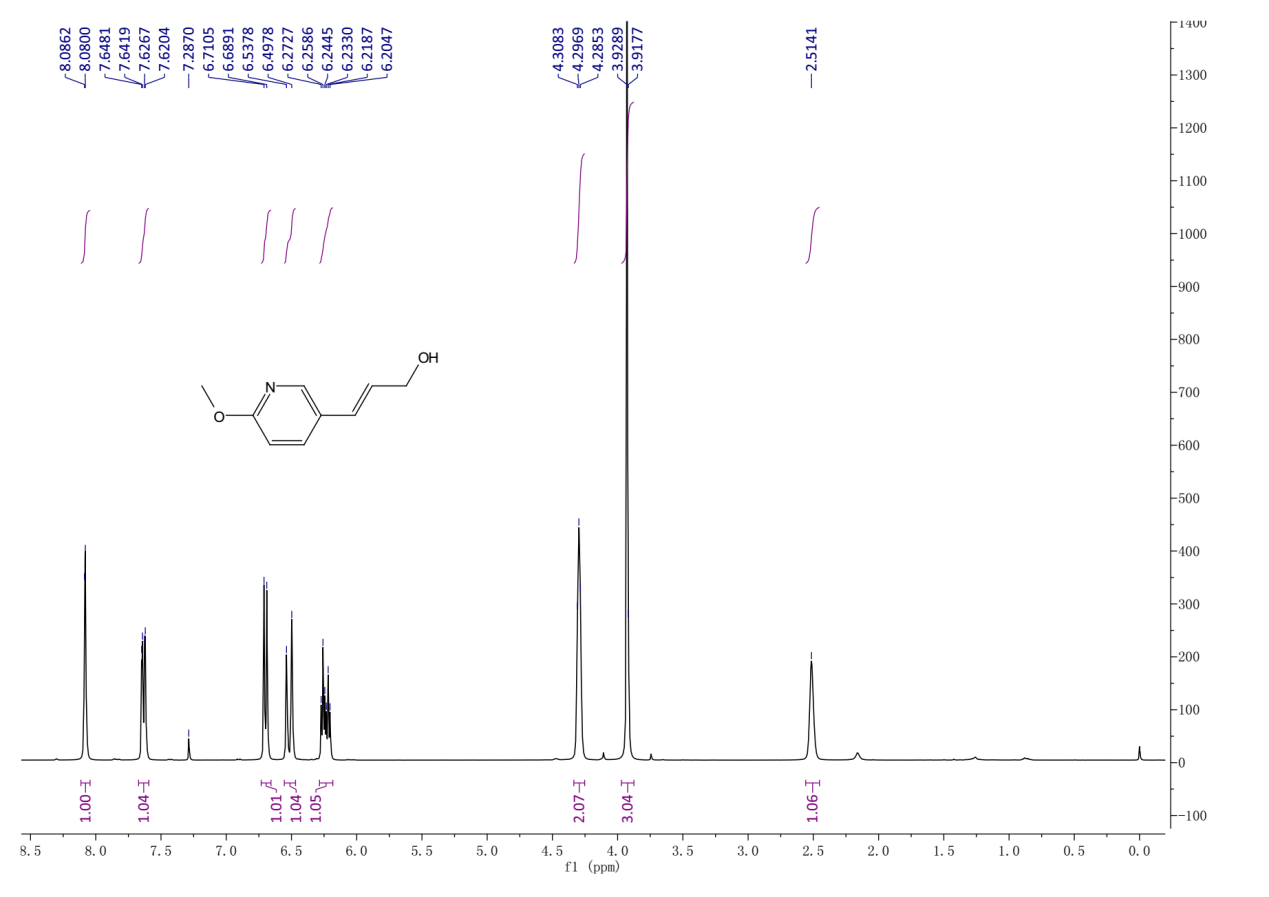


^1^H-NMR (400 MHz, Chloroform-*d*) spectrum of **5c**.


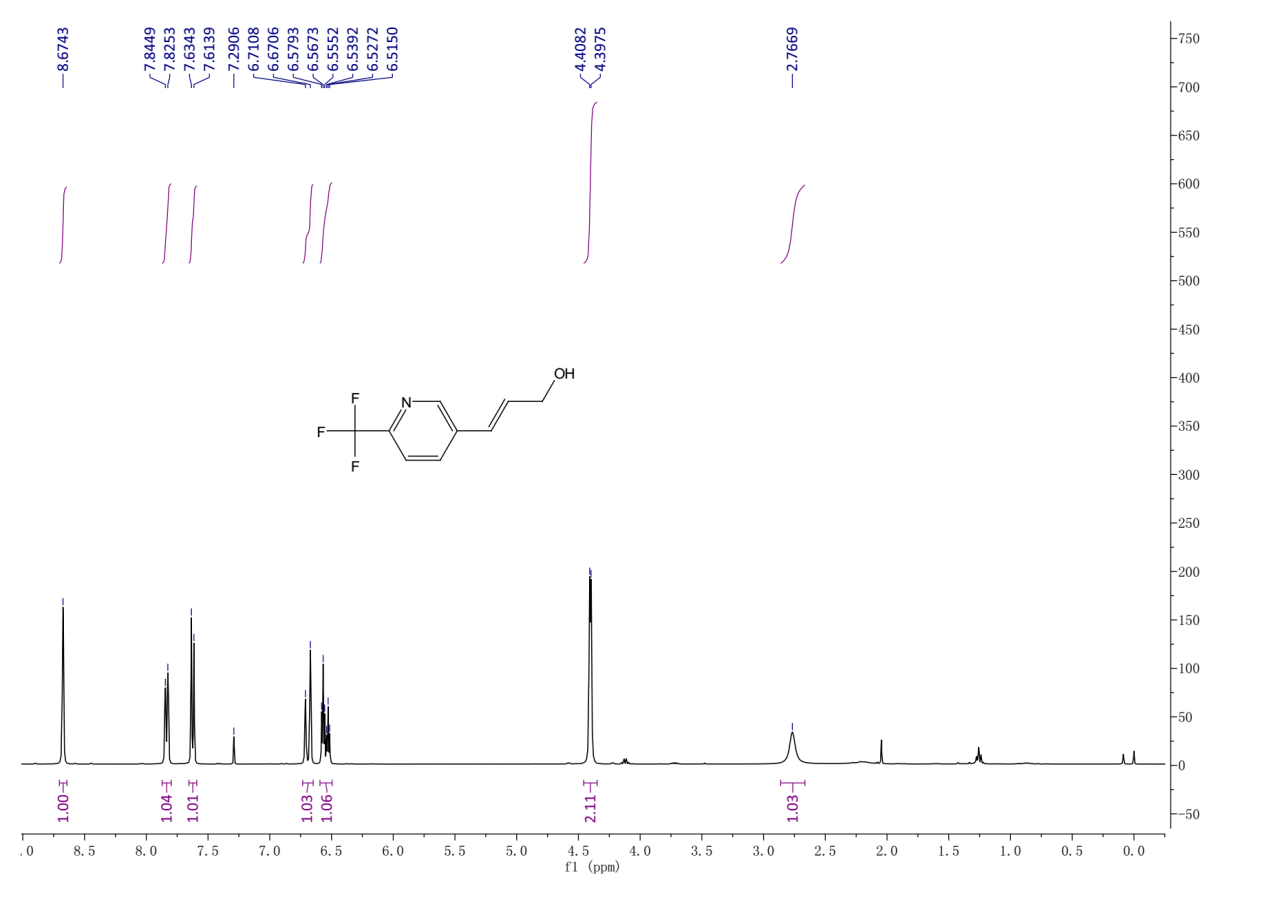


^1^H-NMR (400 MHz, Chloroform-*d*) spectrum of **5d**.

**2. ^1^H-NMR , ^13^C-NMR and HRMS spectra of compounds 6a-d, 7 and 9.**


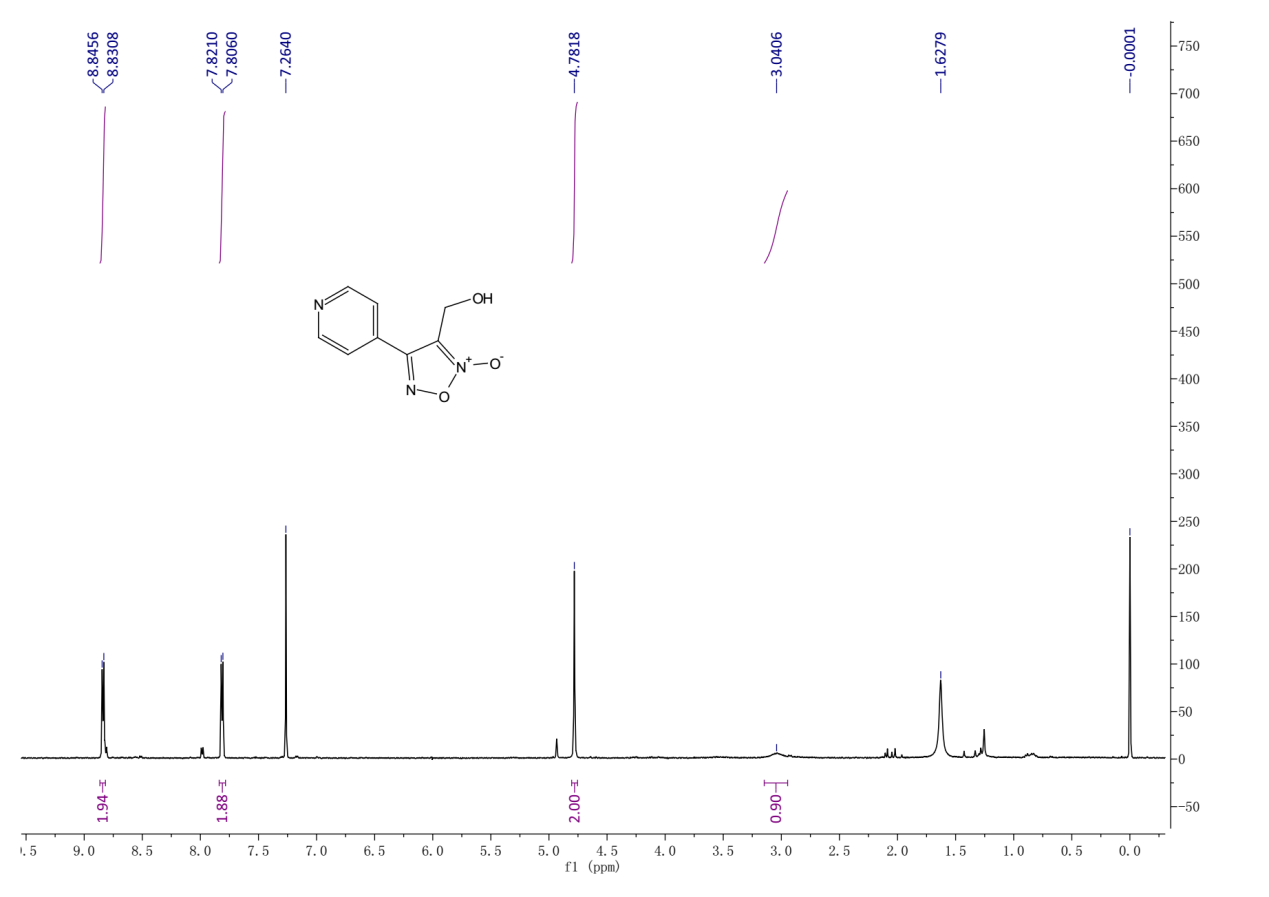


^1^H-NMR (400 MHz, Chloroform-*d*) spectrum of **6a**.


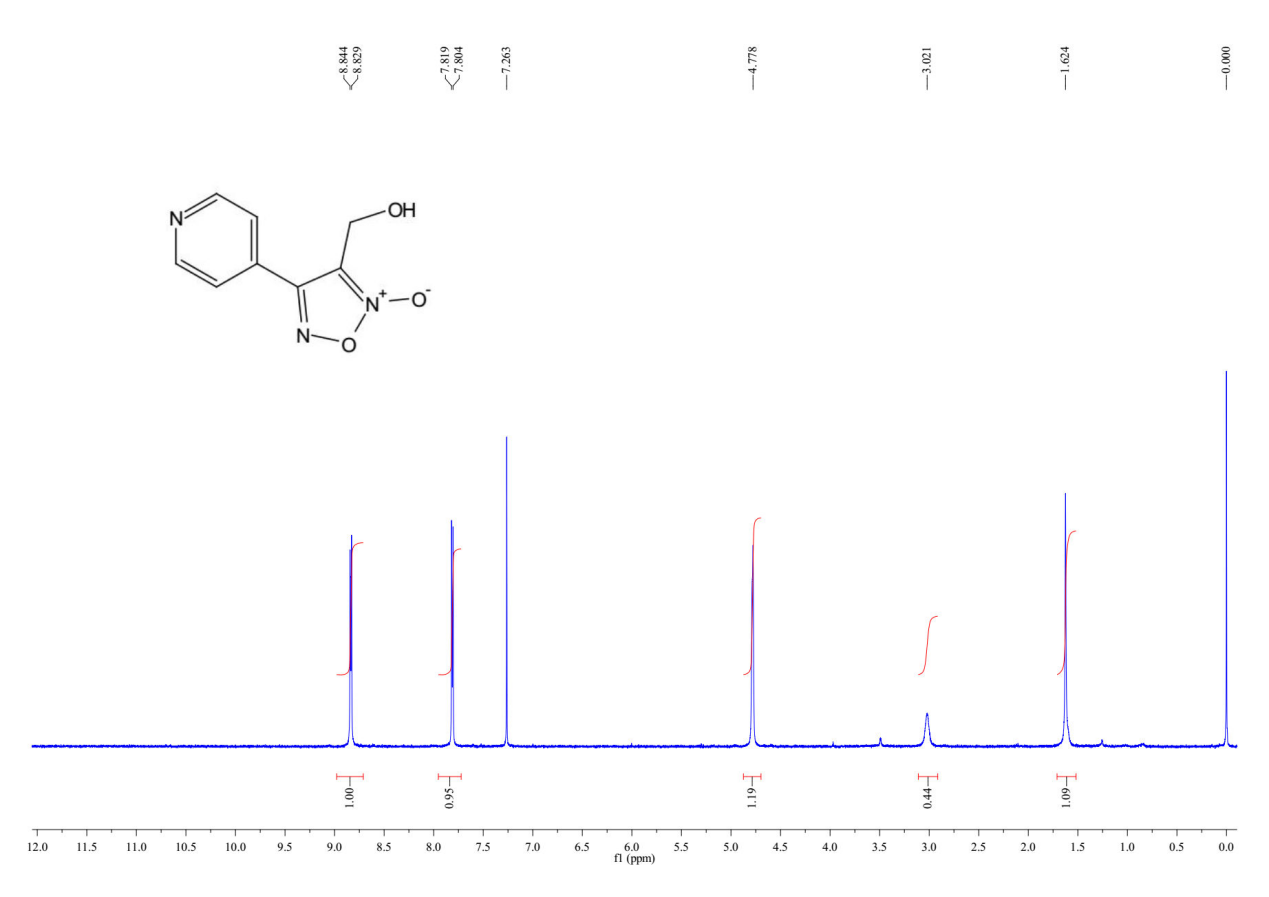


^13^C-NMR (101 MHz, Chloroform-*d*) spectrum of **6a**.


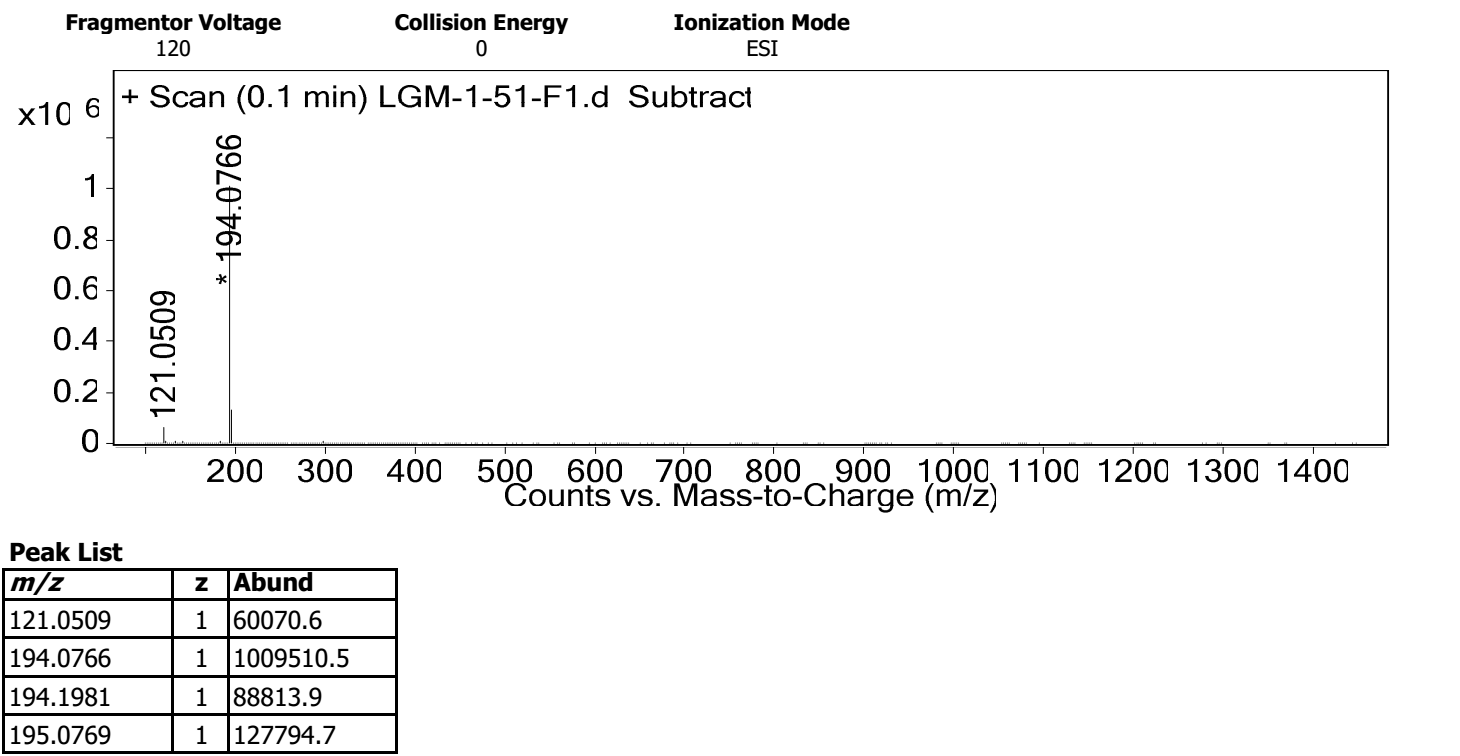


HRMS spectrum of **6a**.


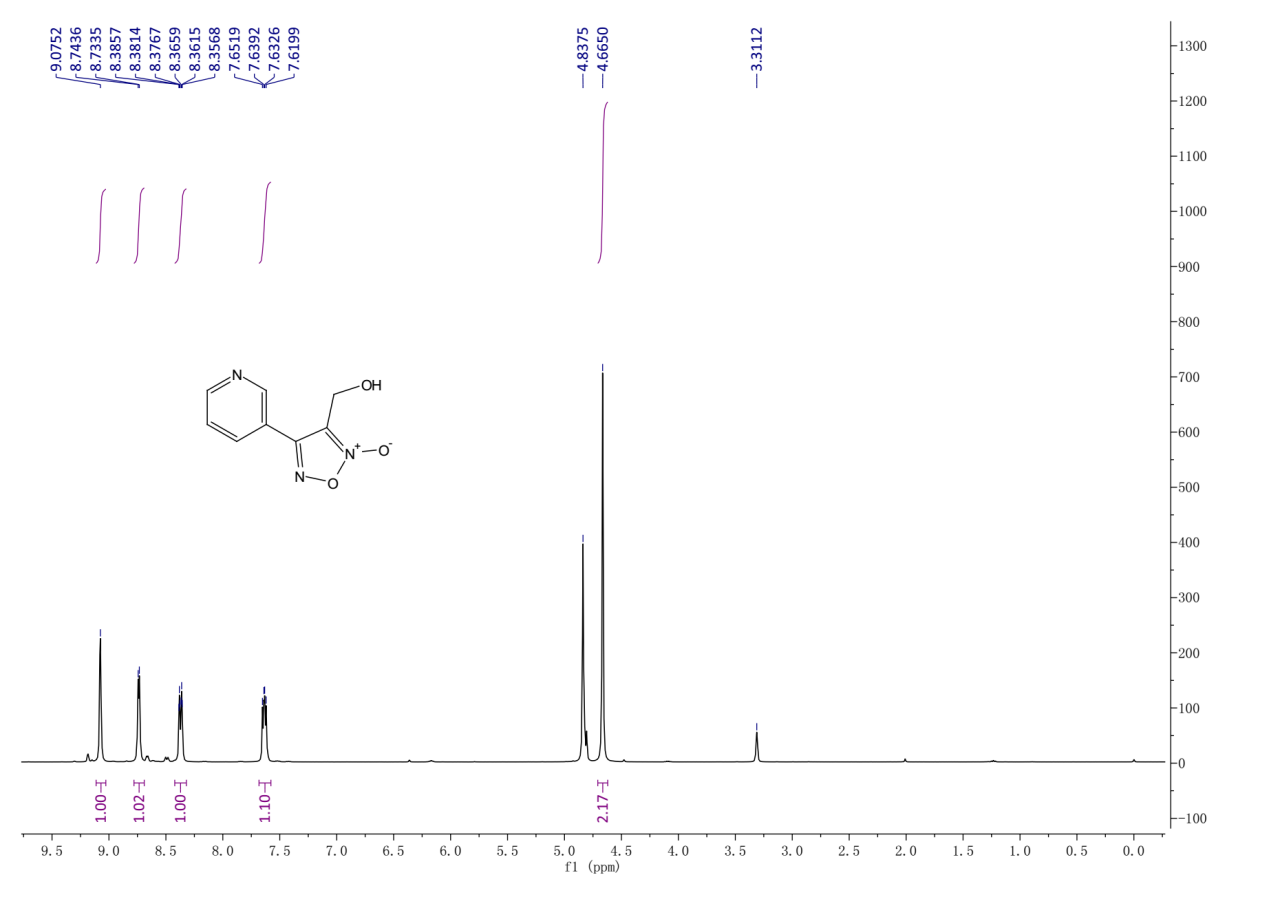


^1^H-NMR (400 MHz, Methanol-*d*4) spectrum of **6b**.


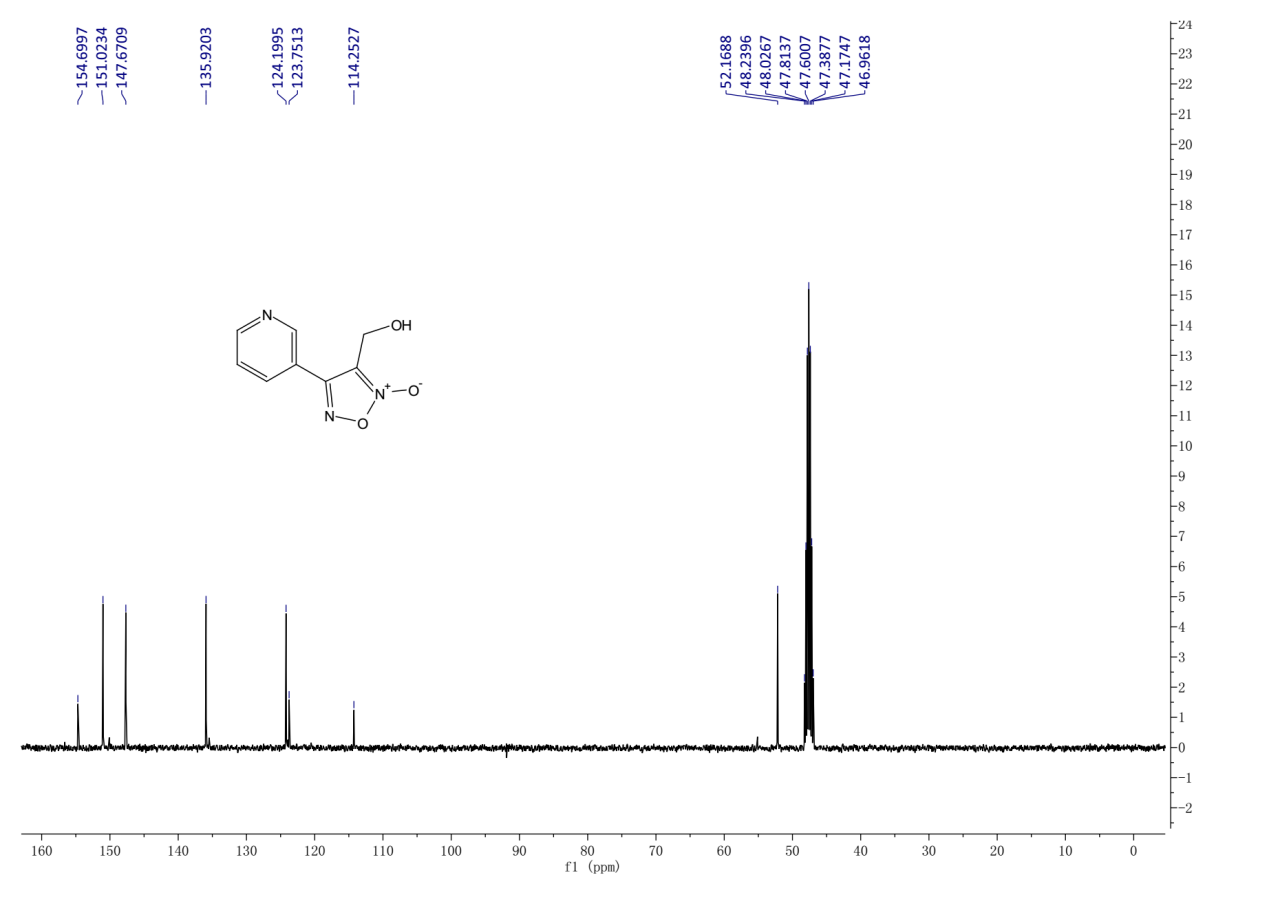


^13^C-NMR (101 MHz, Methanol-*d*4) spectrum of **6b**.


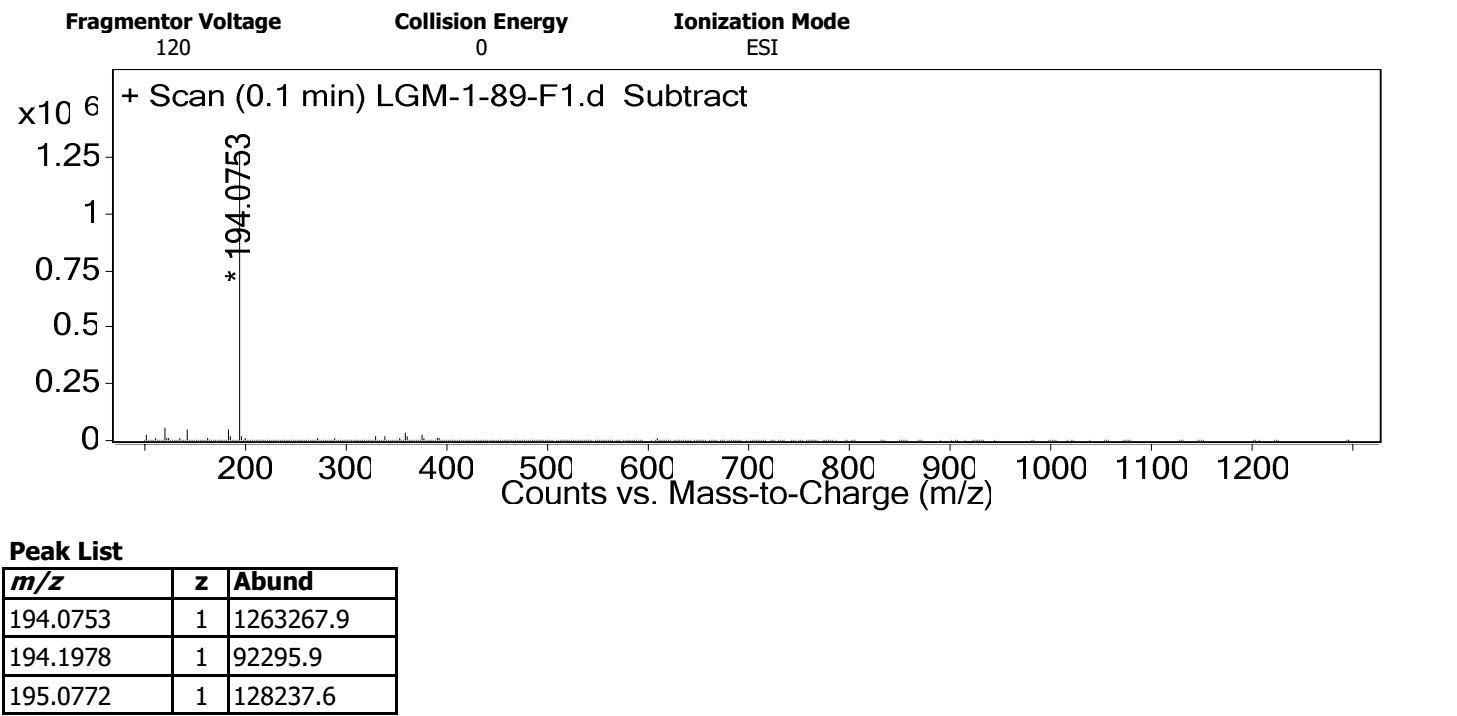


HRMS spectrum of **6b**.


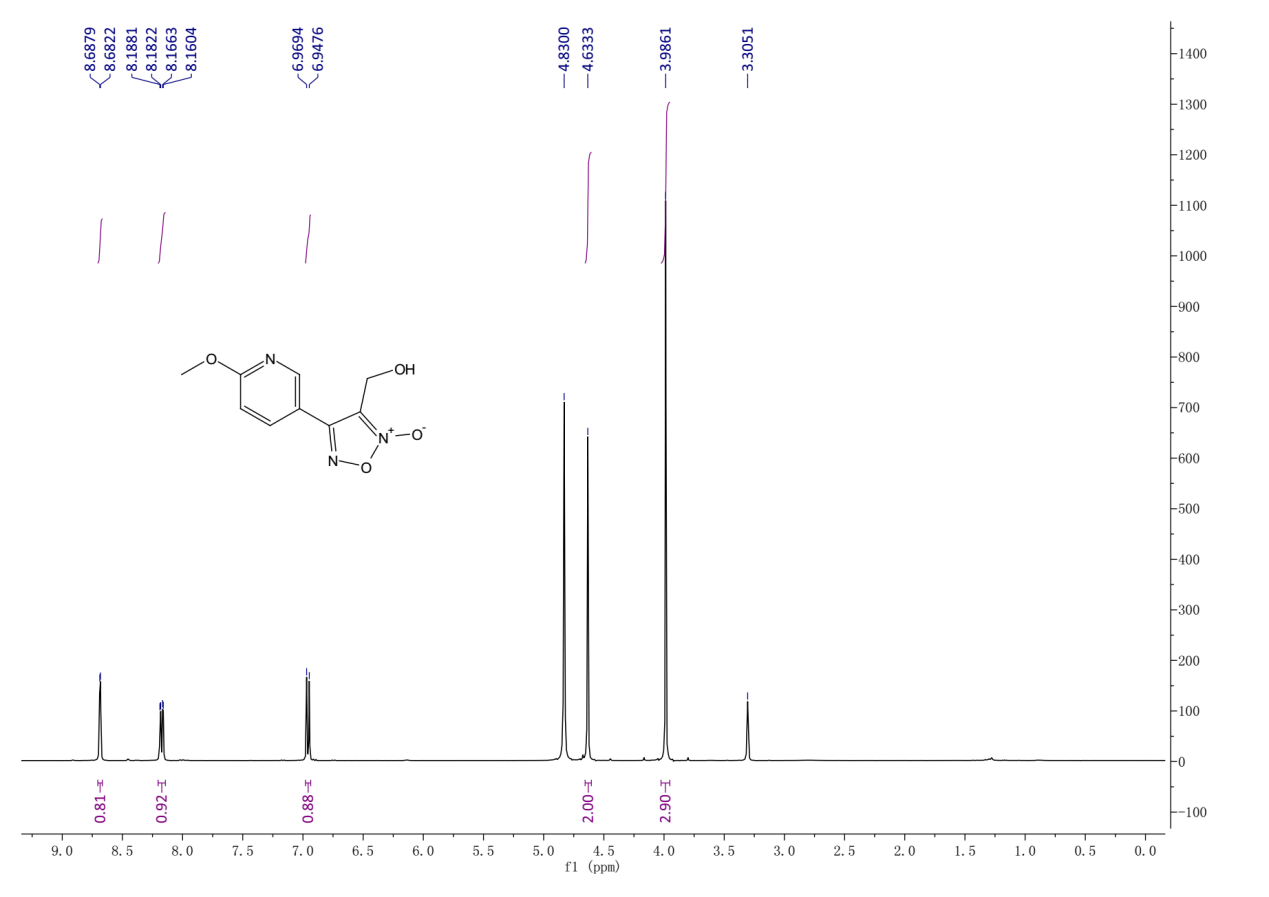


^1^H-NMR (400 MHz, Methanol-*d*4) spectrum of **6c**.


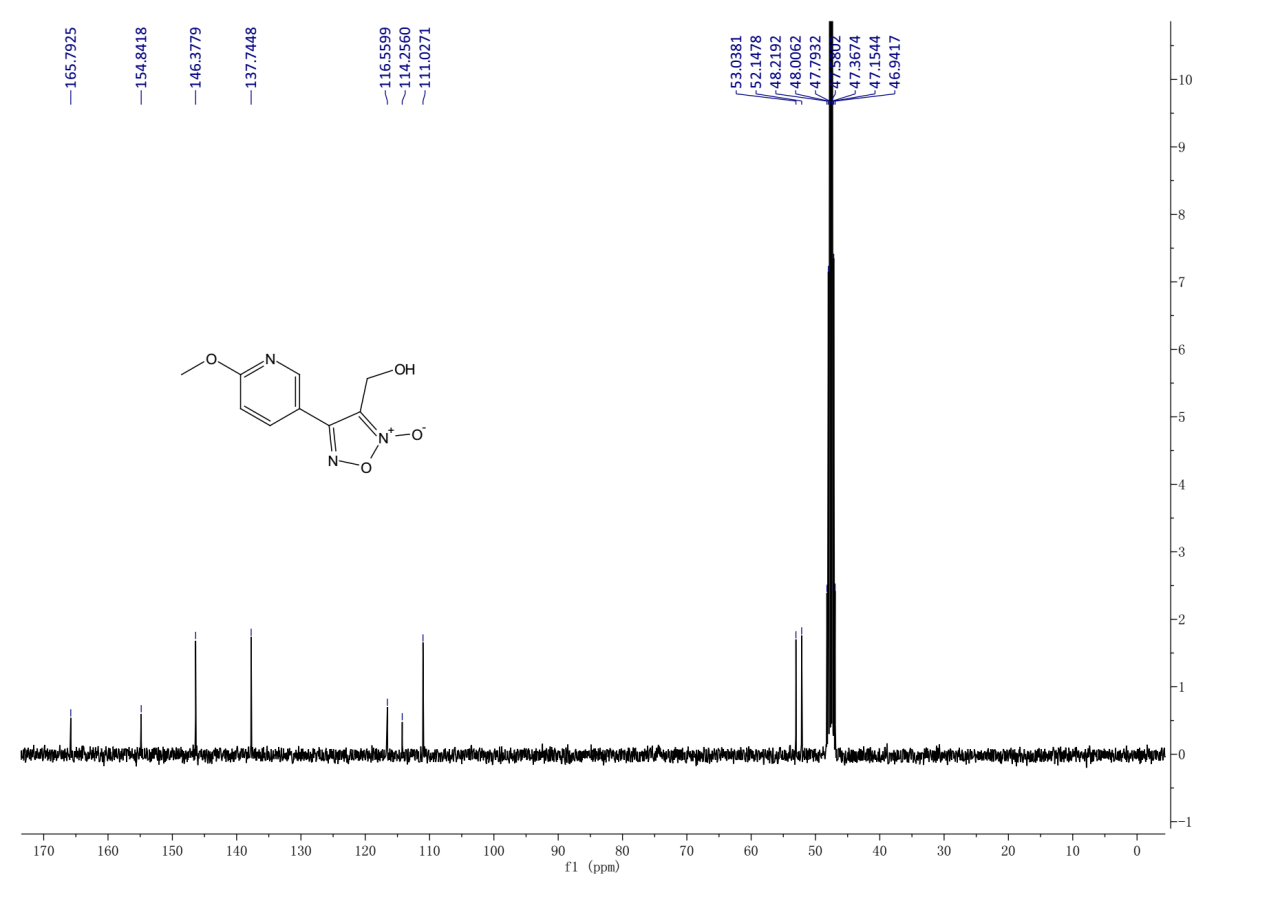


^13^C-NMR (101MHz, Methanol-*d*4) spectrum of **6c**.


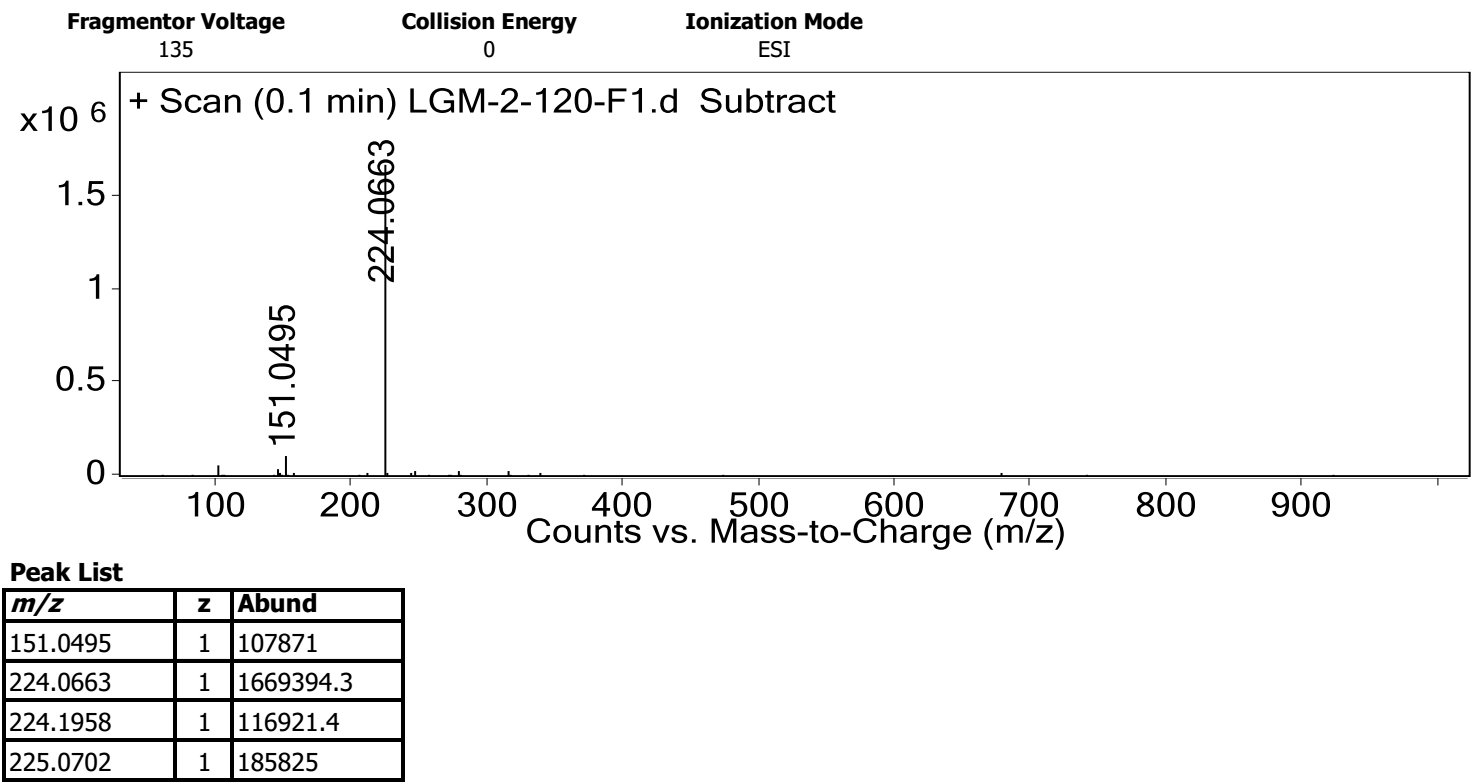


HRMS spectrum of **6c**.


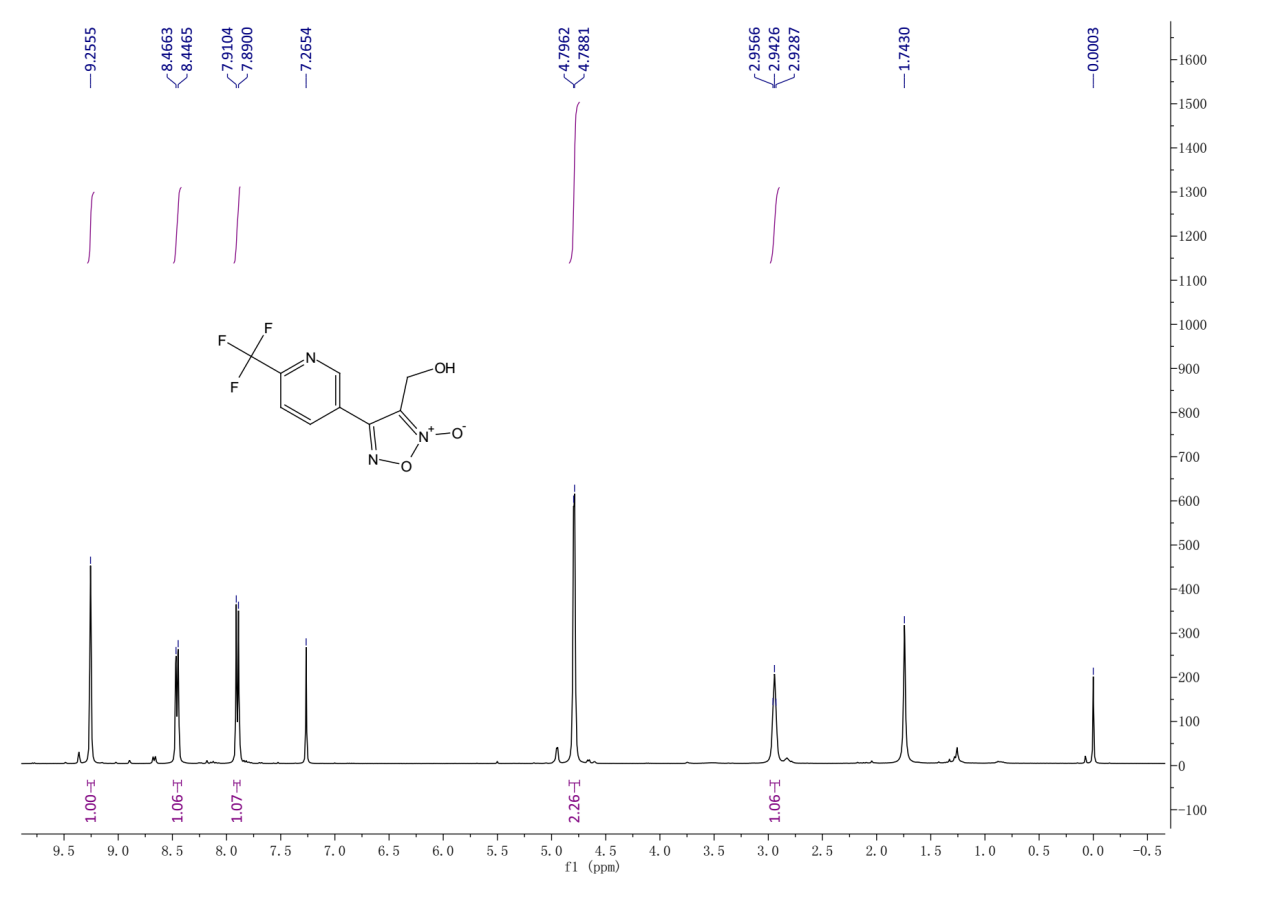


^1^H-NMR (400 MHz, Chloroform-*d*) spectrum of **6d**.


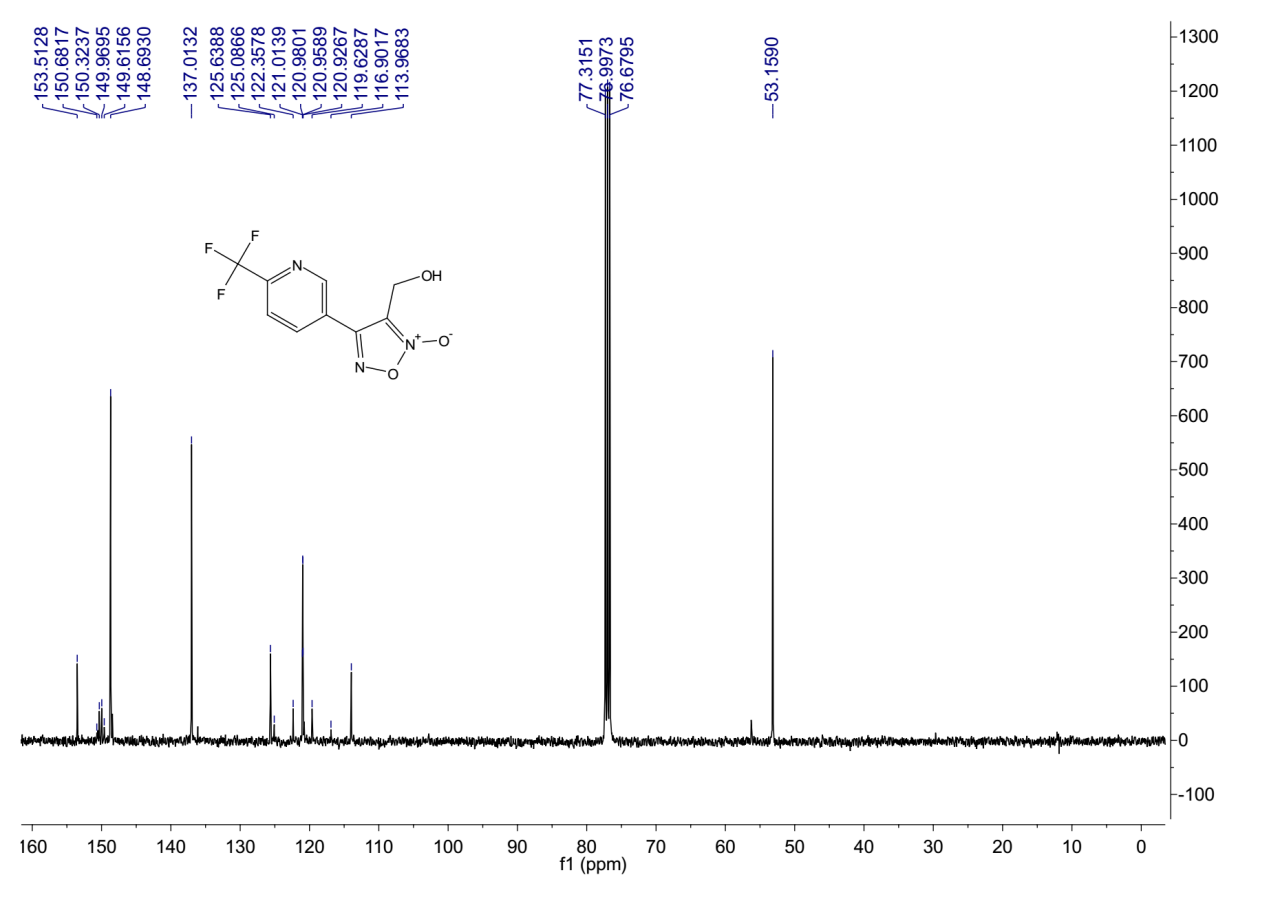


^13^C-NMR (101 MHz, Chloroform-*d*) spectrum of **6d**.


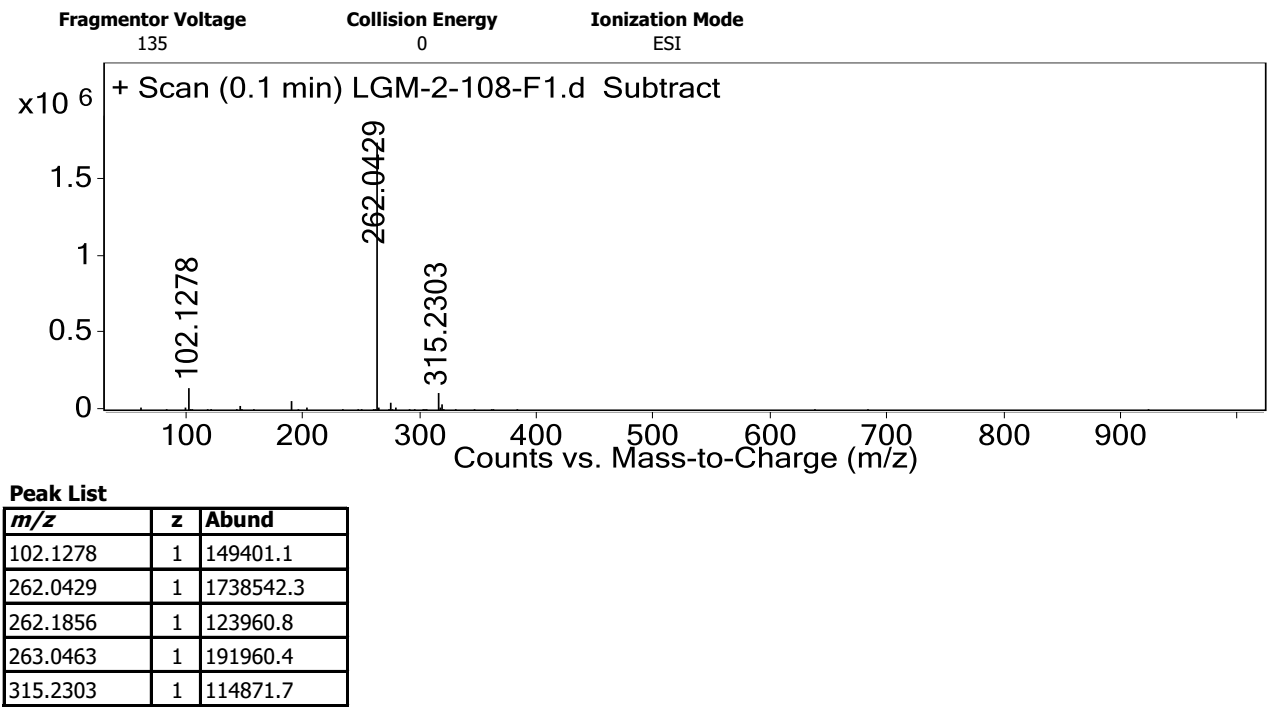


HRMS spectrum of **6d**.


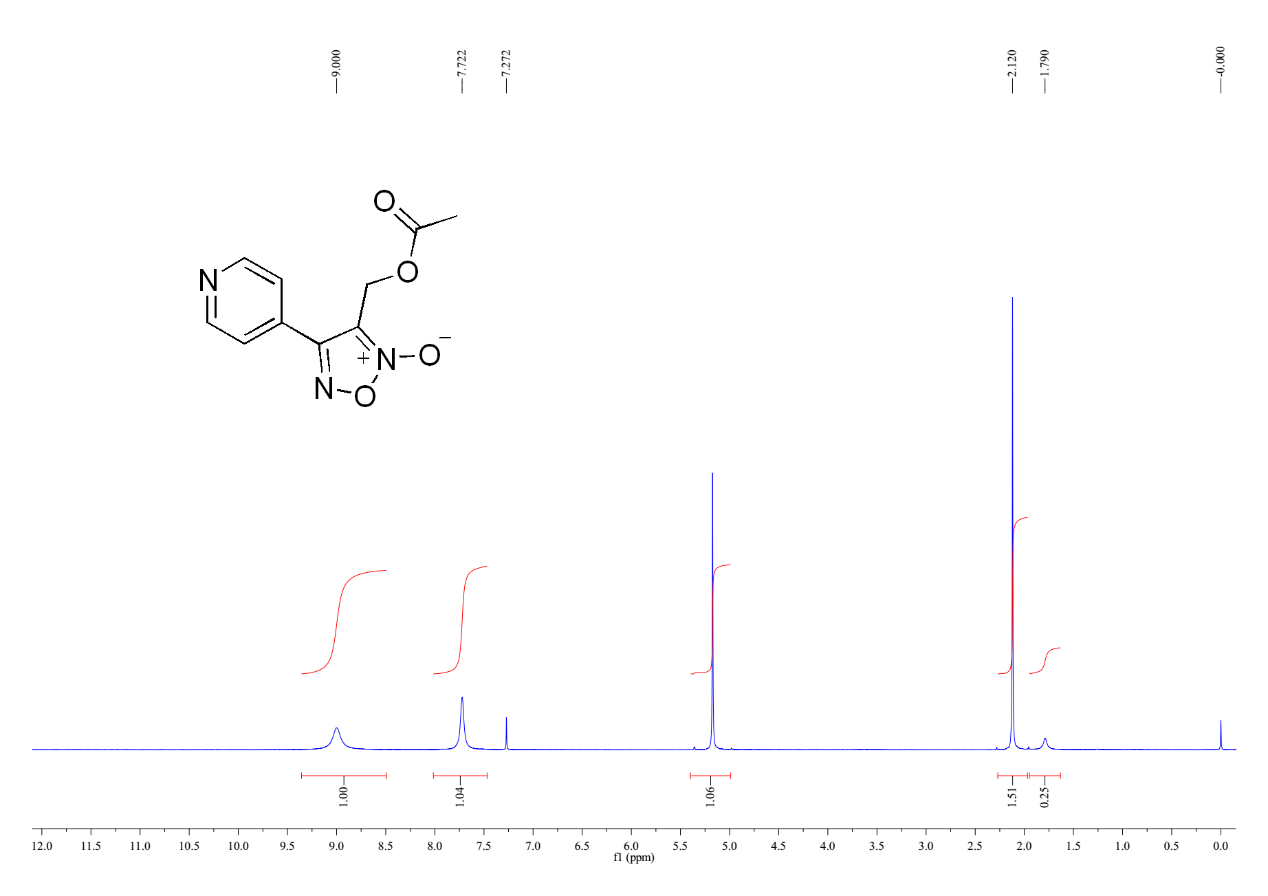


^1^H-NMR (400 MHz, Chloroform-*d*) spectrum of **7aa**.


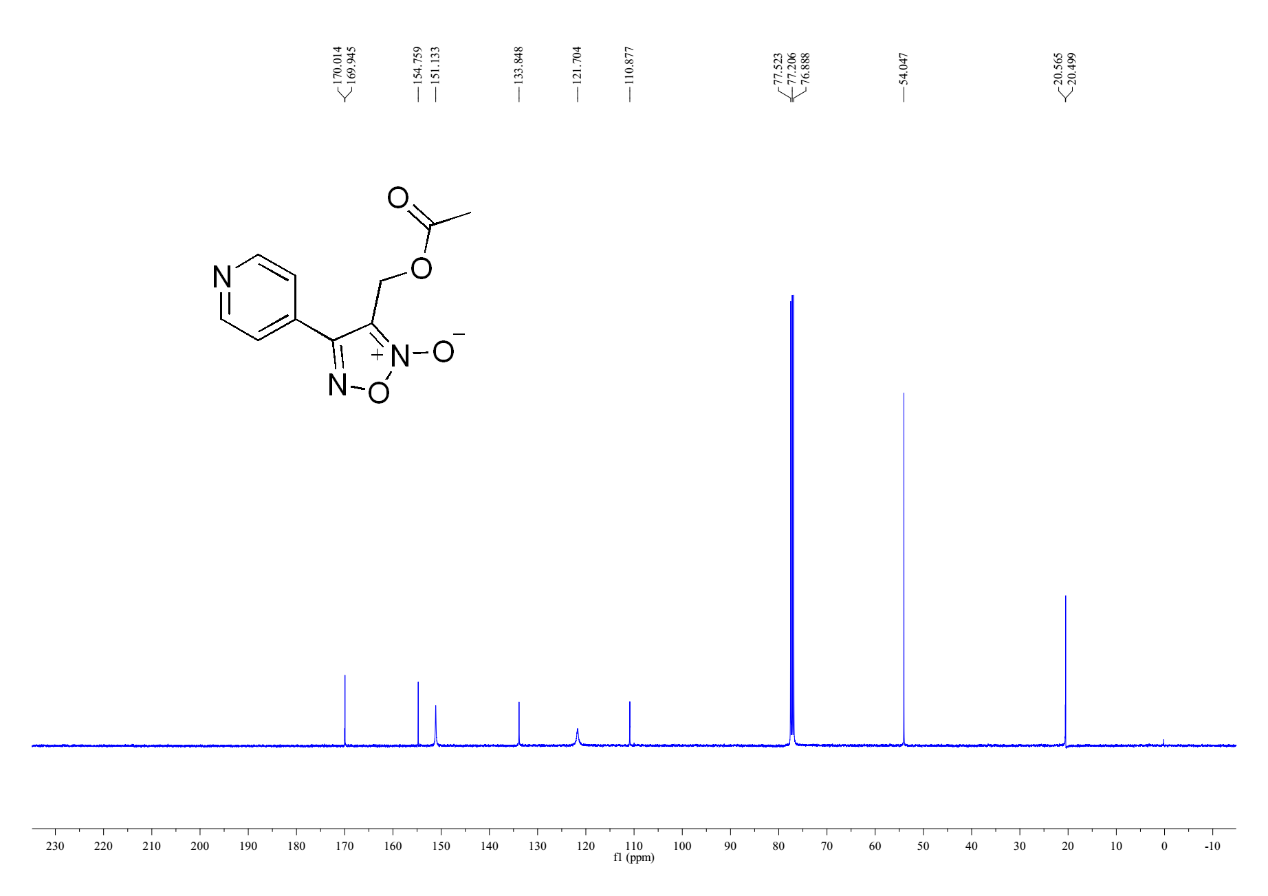


^13^C-NMR (101 MHz, Chloroform-*d*) spectrum of **7aa**.


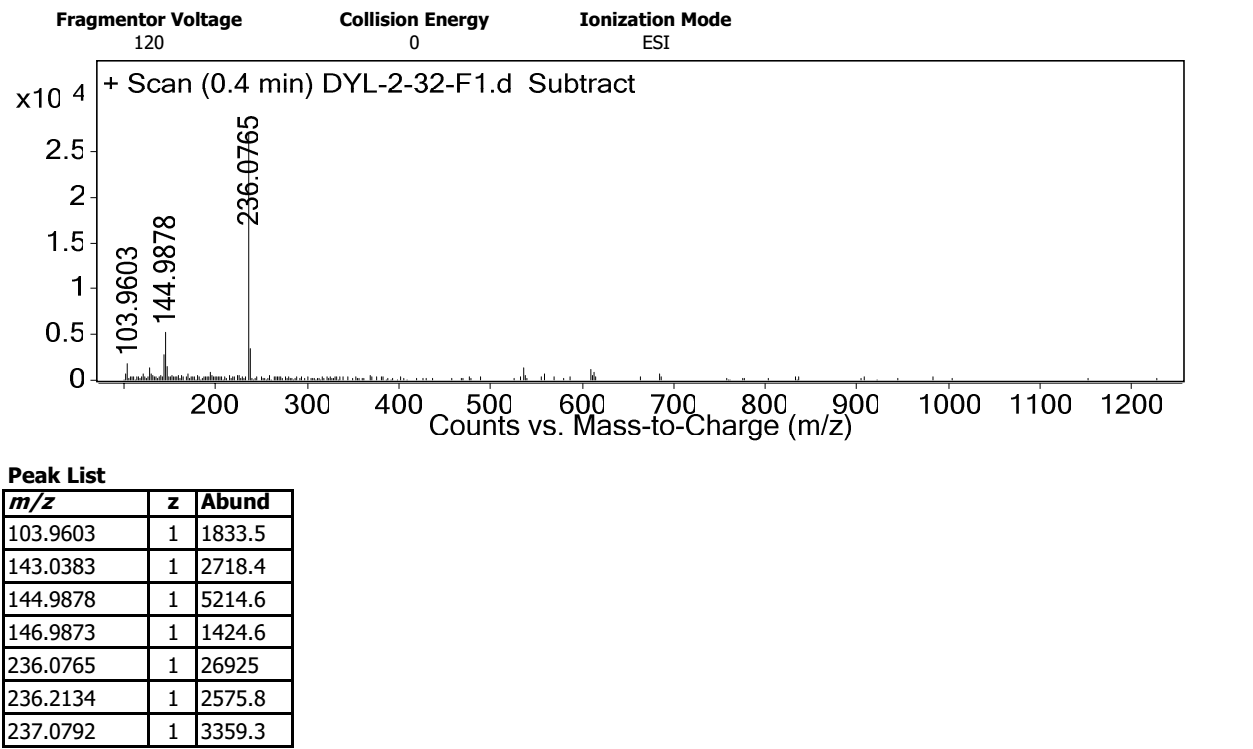


HRMS spectrum of **7aa**.


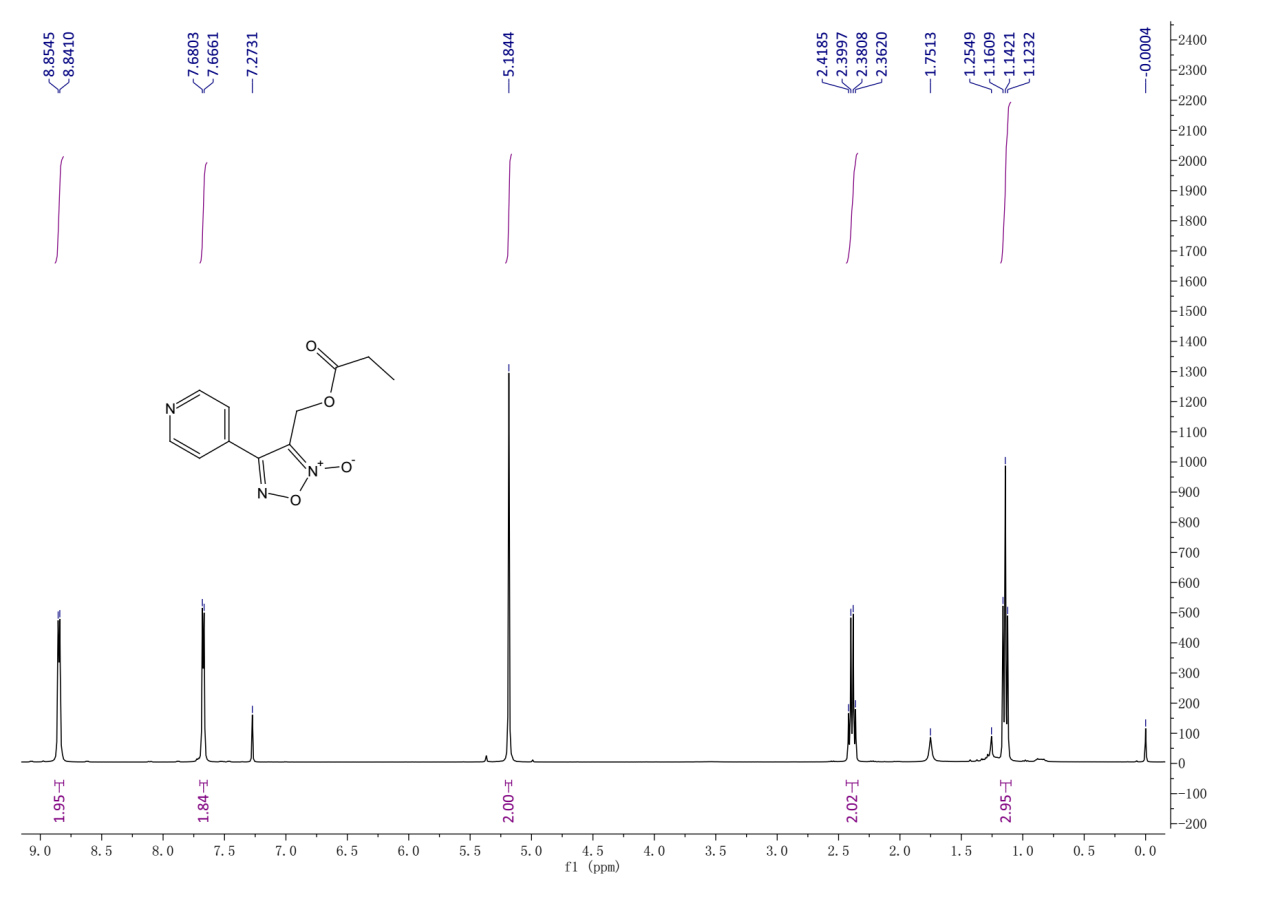


^1^H-NMR (400 MHz, Chloroform-*d*) spectrum of **7ab**.


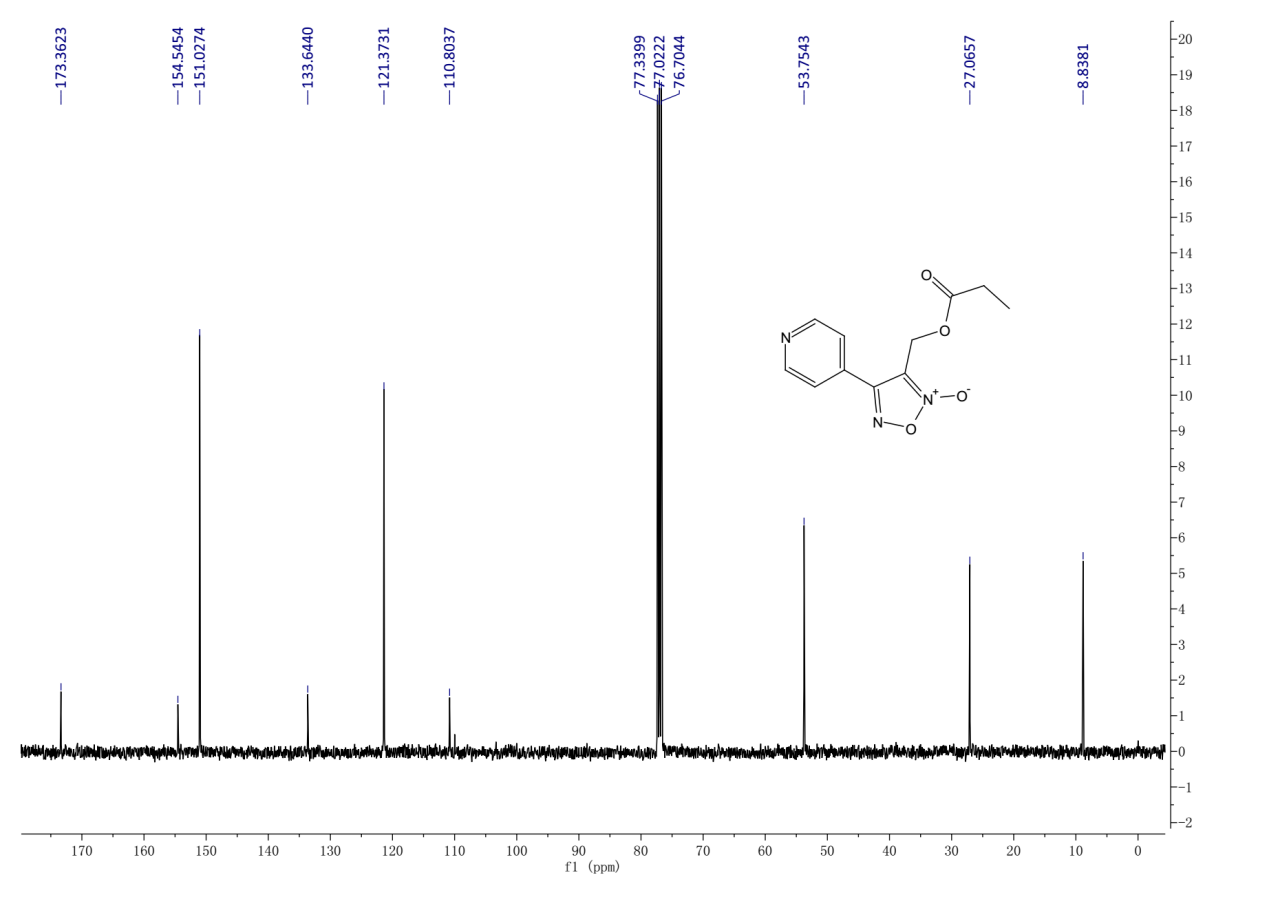


^13^C-NMR (101 MHz, Chloroform-*d*) spectrum of **7ab**.


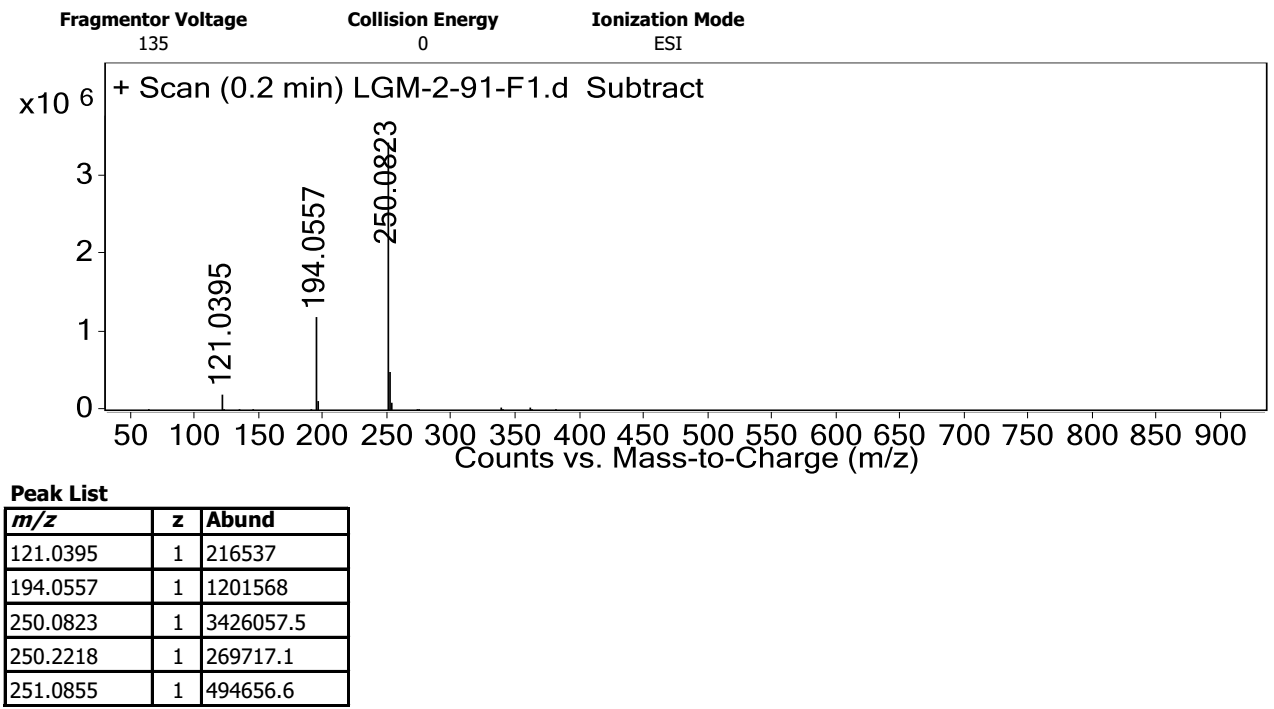


HRMS spectrum of **7ab**.


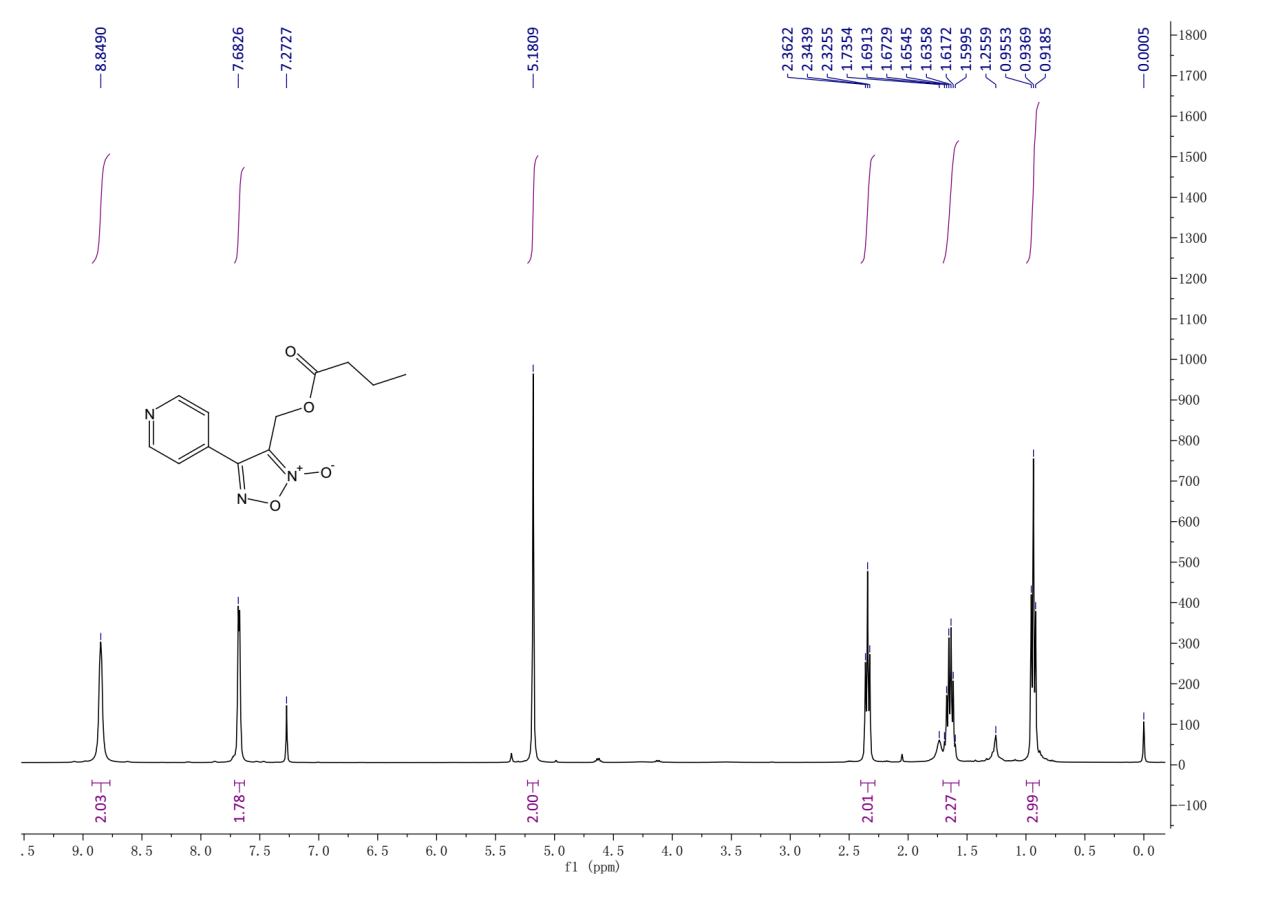


^1^H-NMR (400 MHz, Chloroform-*d*) spectrum of **7ac**.


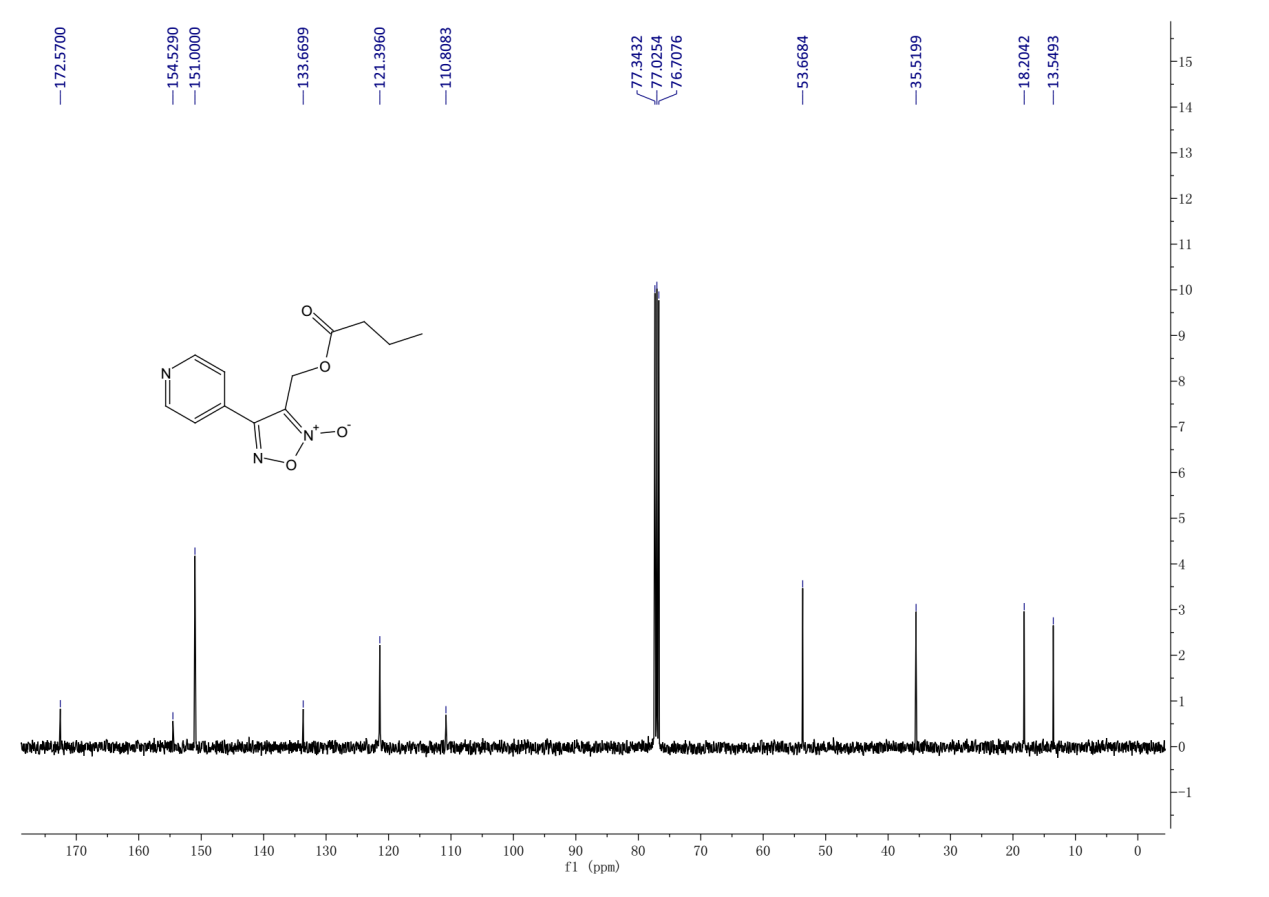


^13^C-NMR (101 MHz, Chloroform-*d*) spectrum of **7ac**.


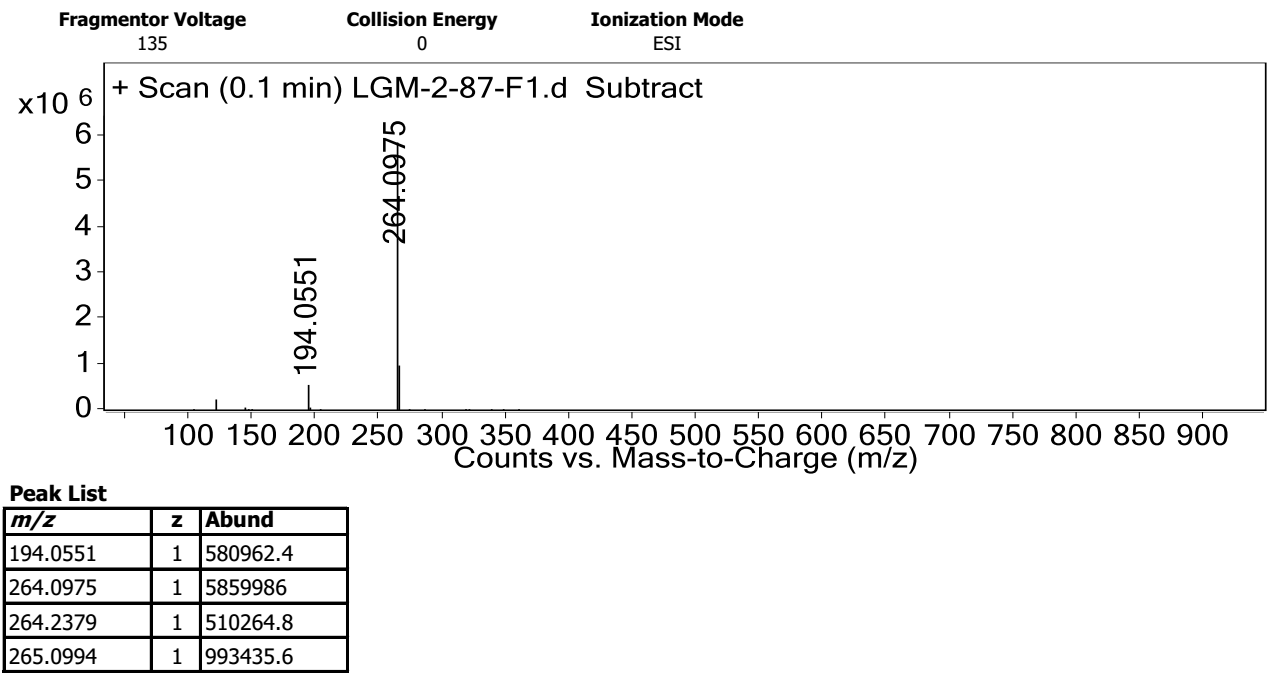


HRMS spectrum of **7ac**.


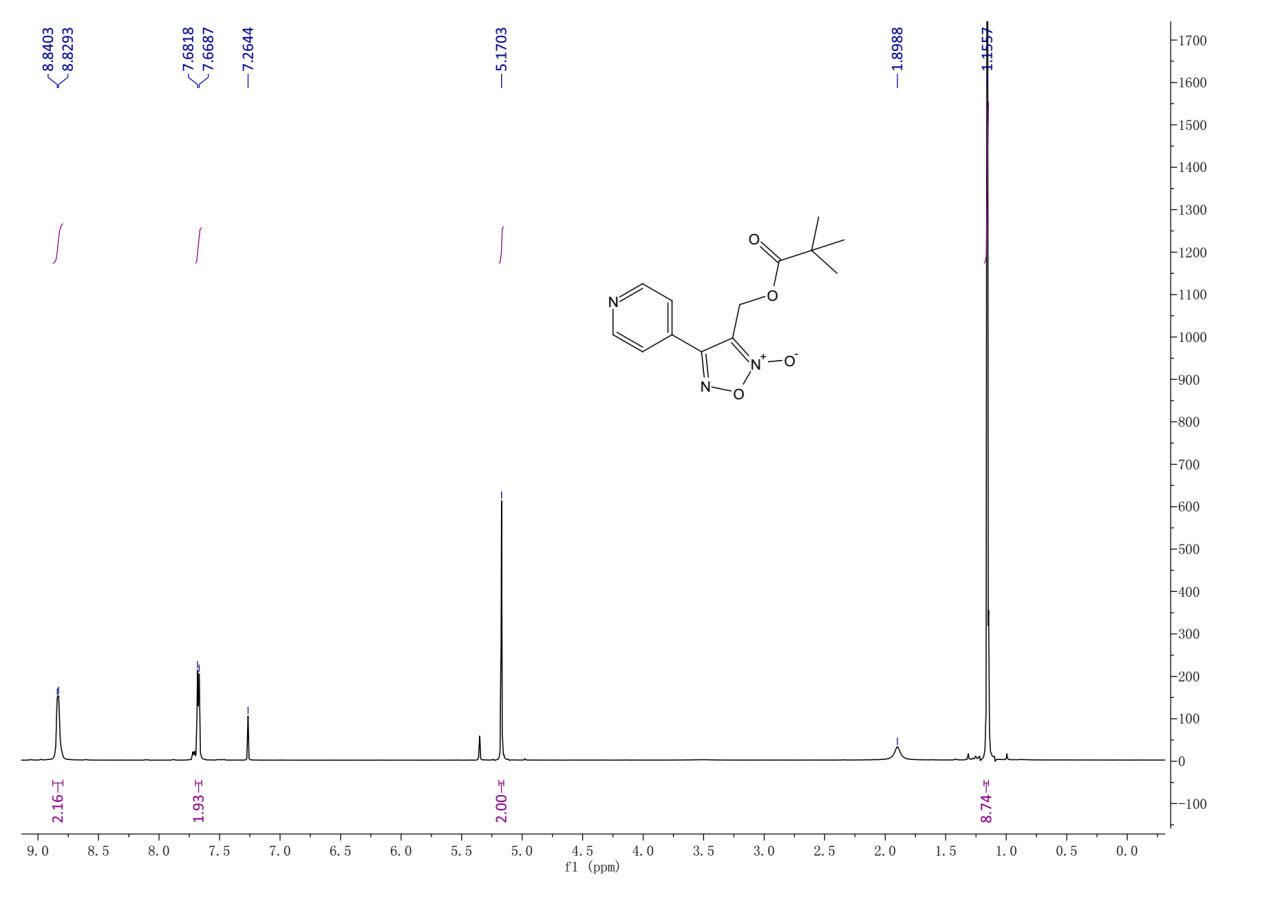
^1^H-NMR (400 MHz, Chloroform-*d*) spectrum of **7ad**..


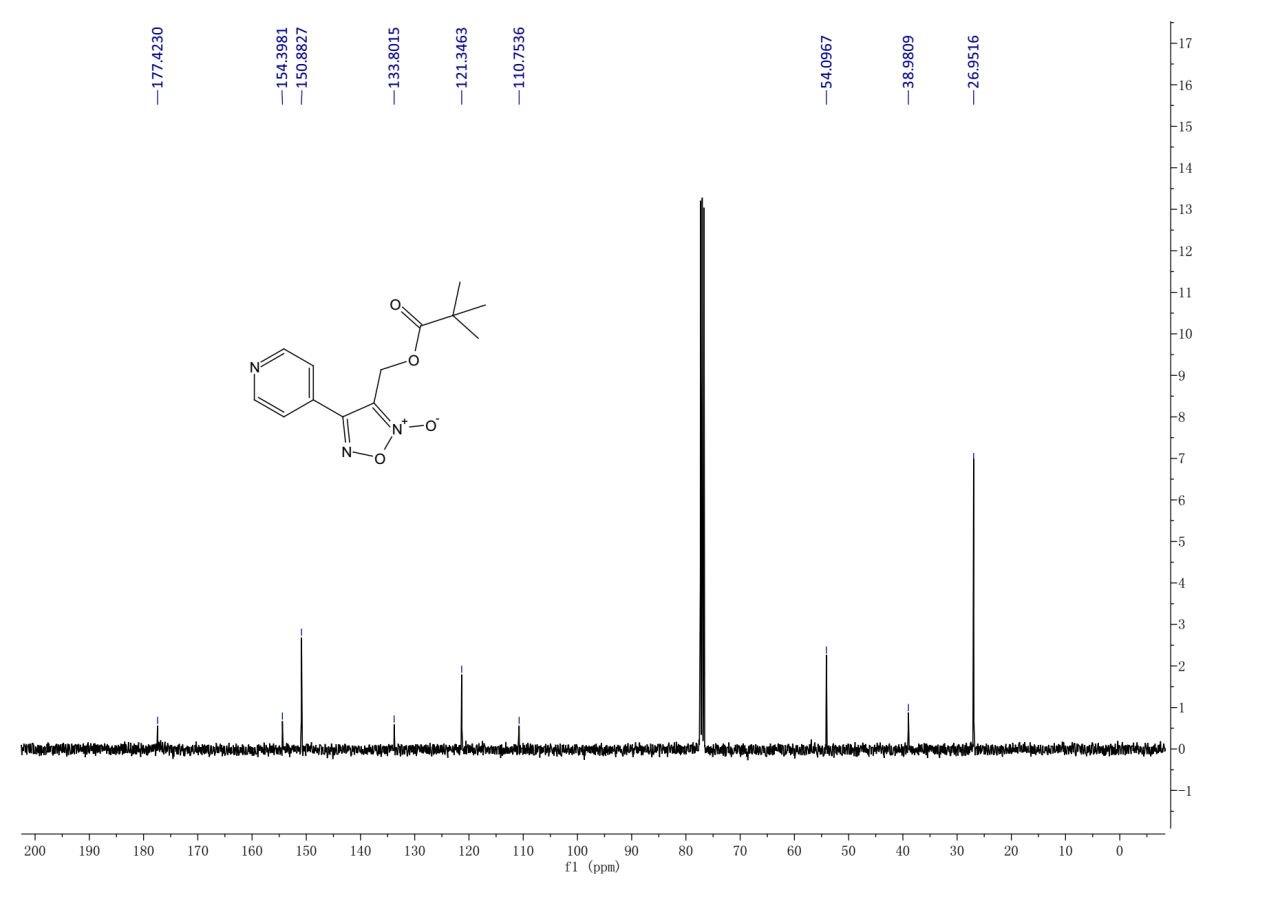


^13^C-NMR (101 MHz, Chloroform-*d*) spectrum of **7ad**.


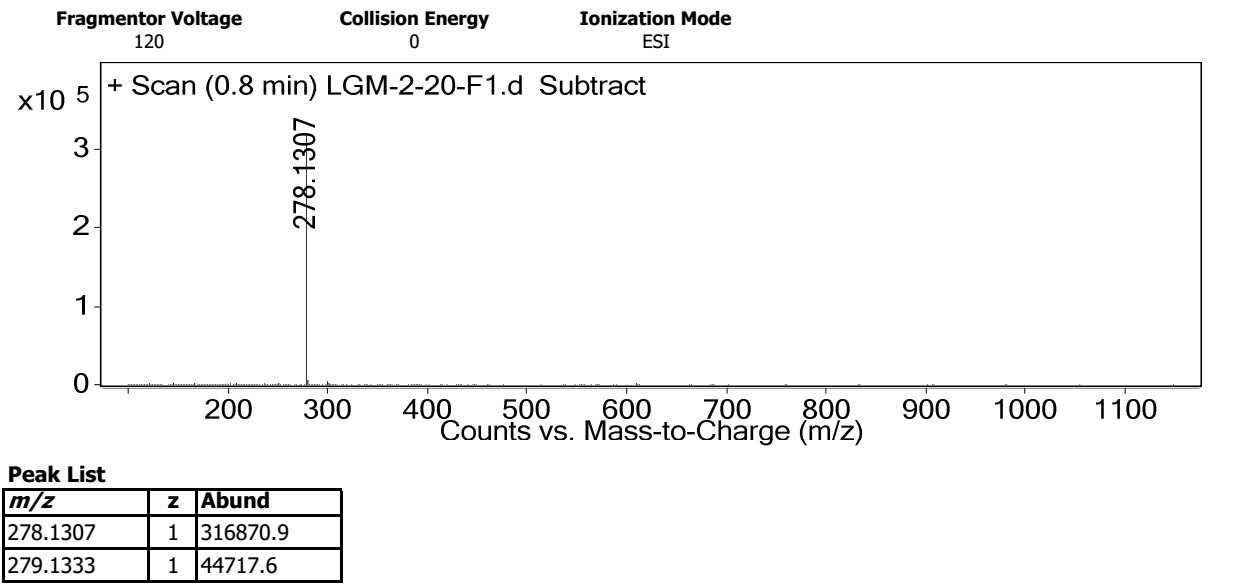


HRMS spectrum of **7ad**.


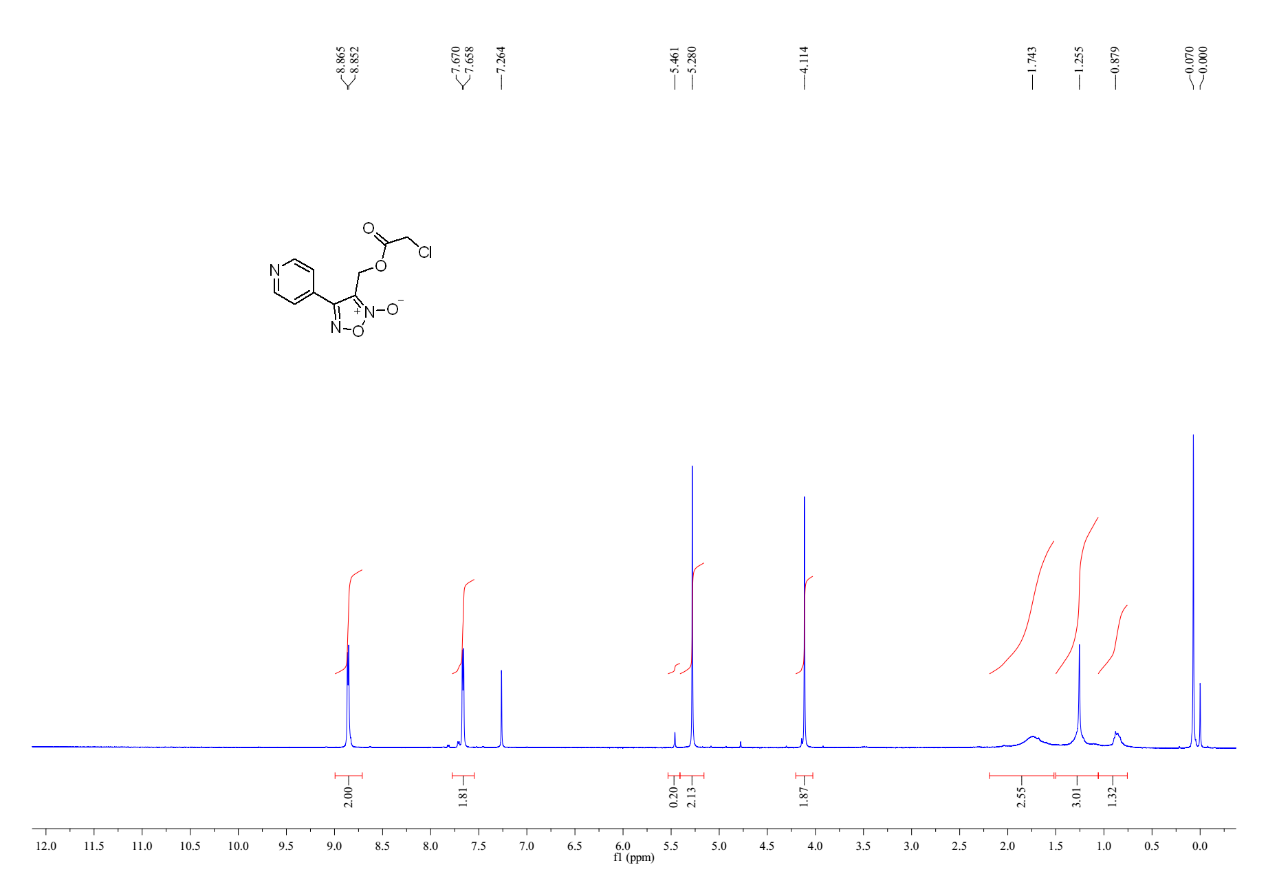


^1^H-NMR (400 MHz, Chloroform-*d*) spectrum of **7ae**.


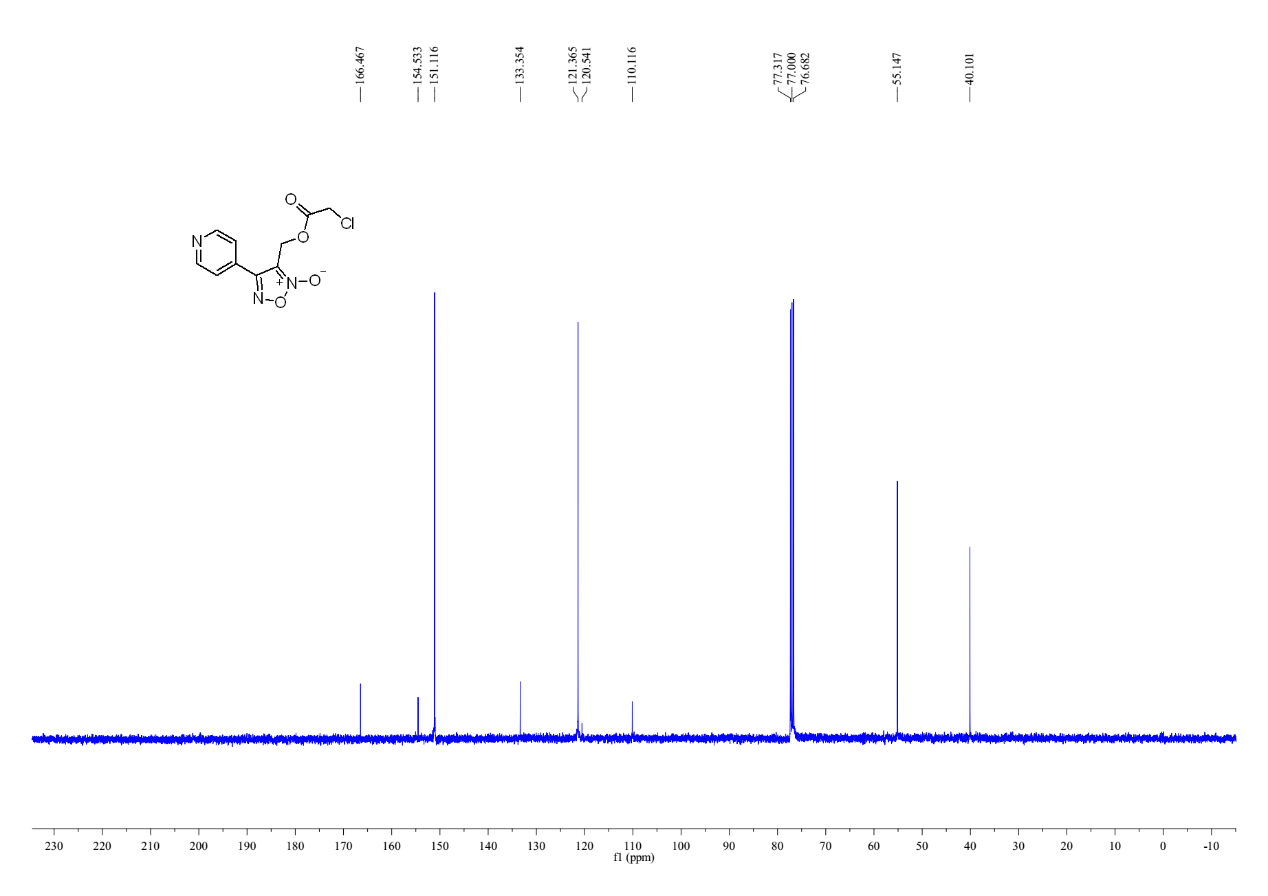


^13^C-NMR (400 MHz, Chloroform-*d*) spectrum of **7ae**.


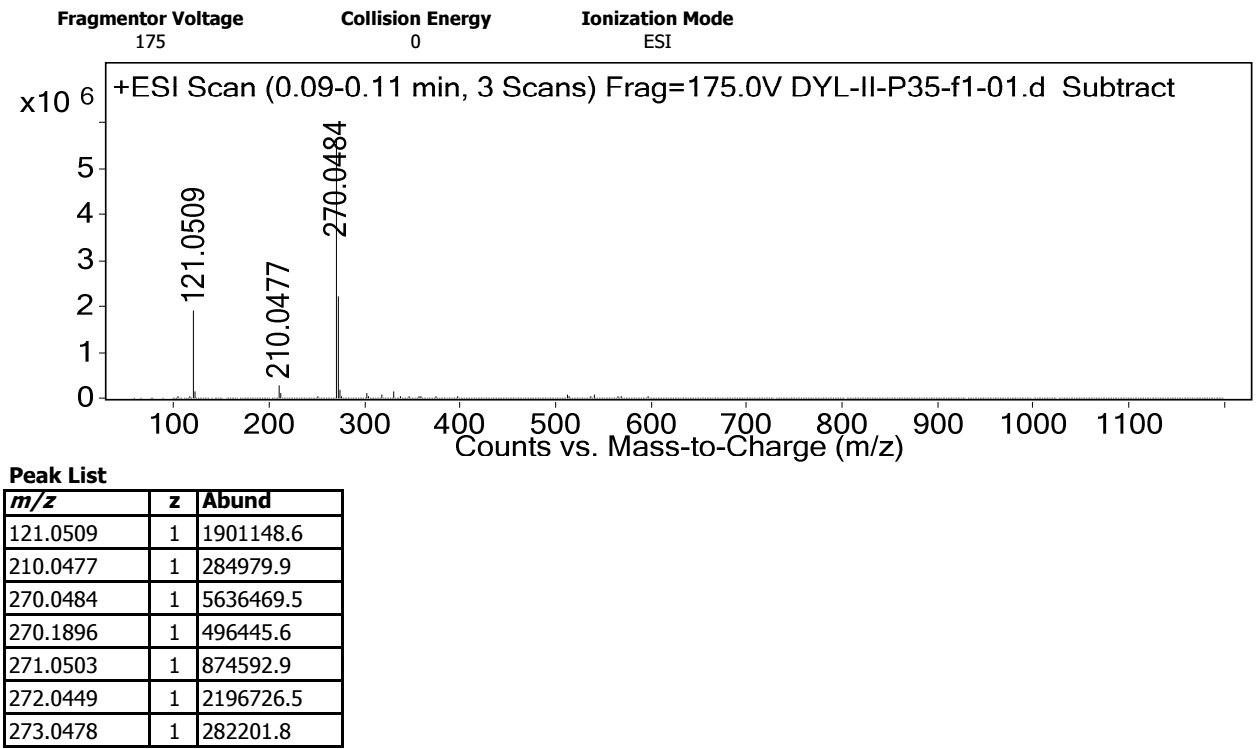


HRMS spectrum of **7ae**.


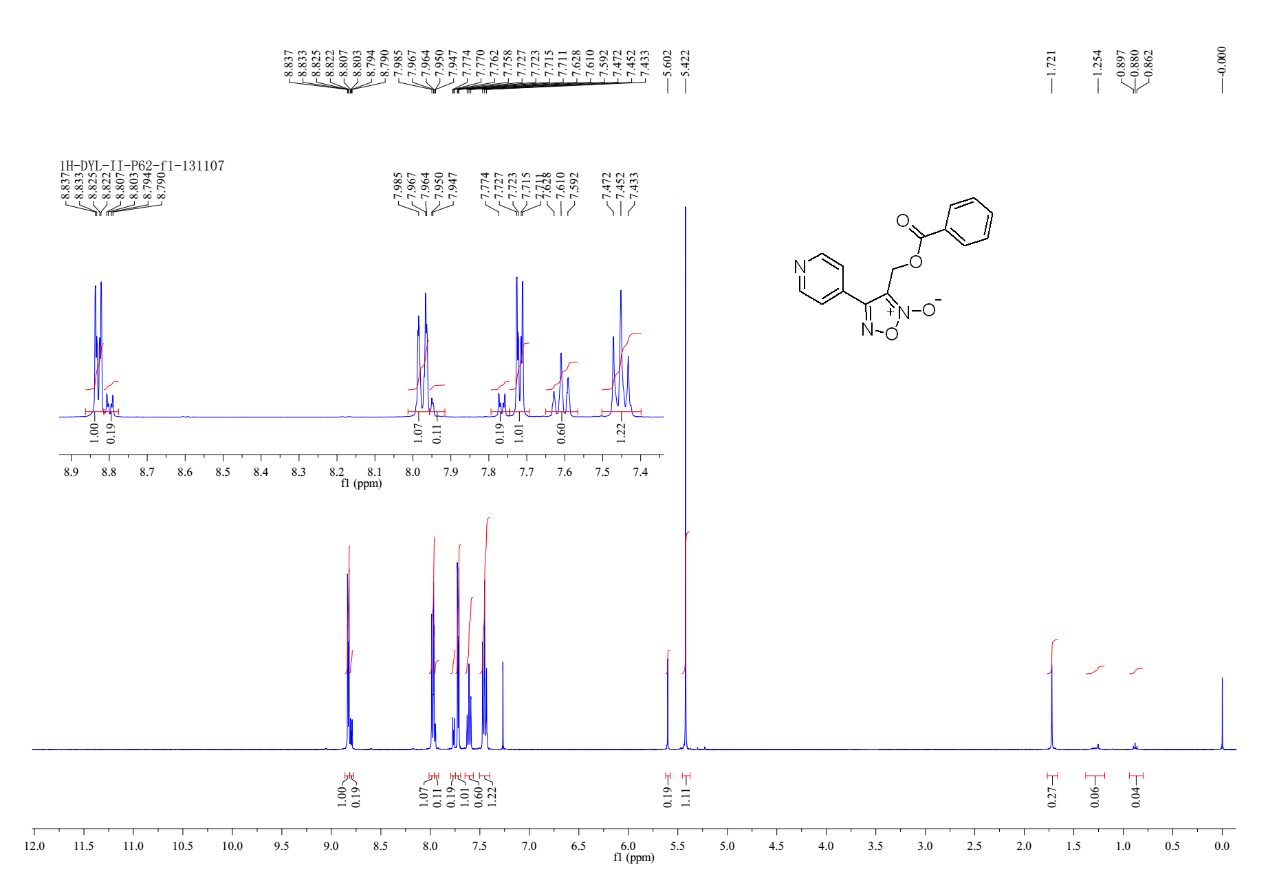


^1^H-NMR (400 MHz, Chloroform-*d*) spectrum of **7af**.


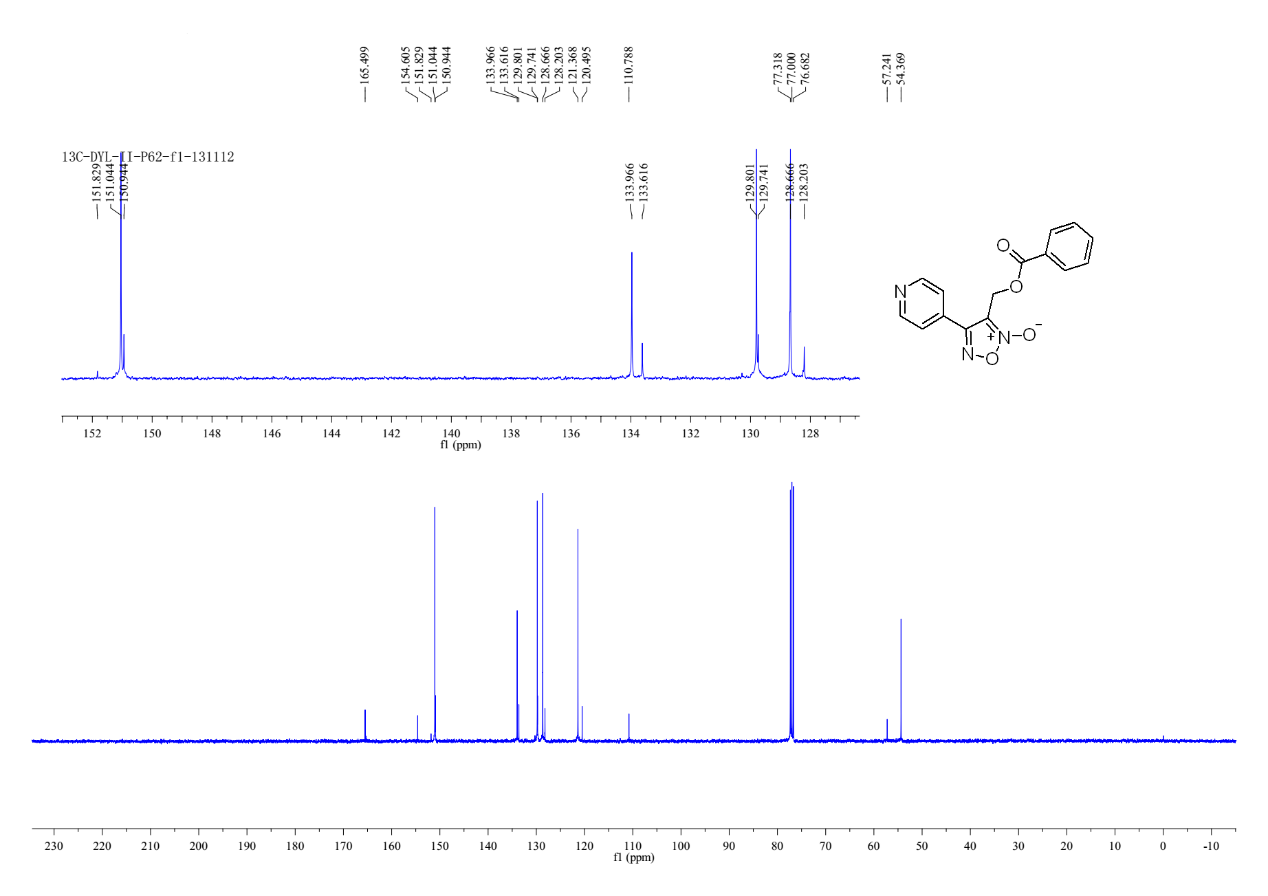


^13^C-NMR(400MHz, Chloroform-*d*) spectrum of **7af**.


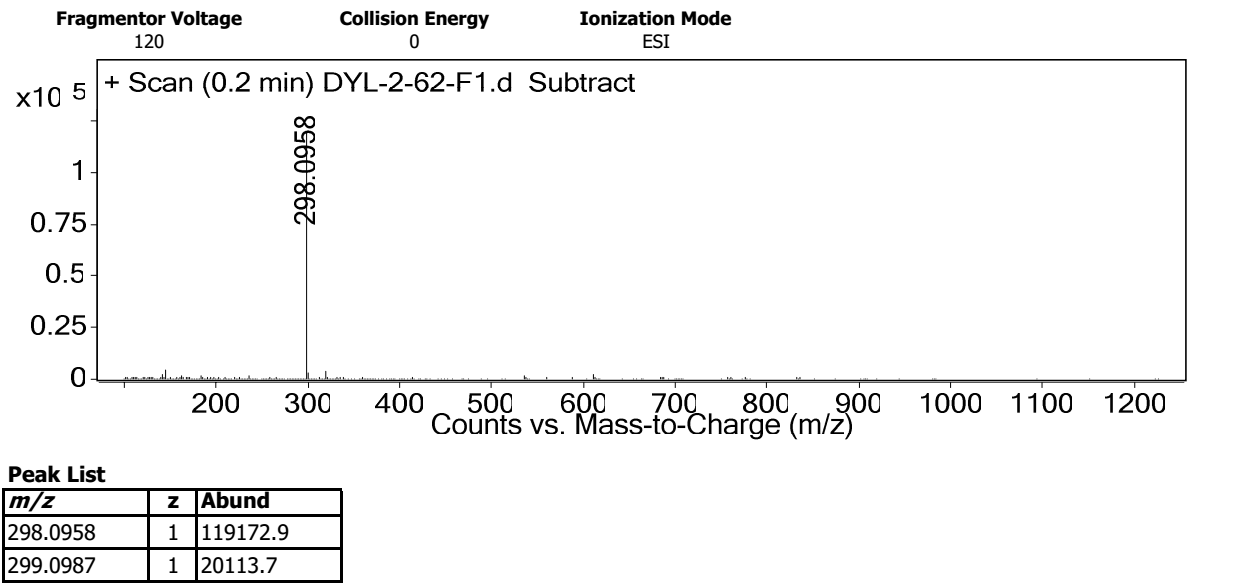


HRMS spectrum of **7af**.


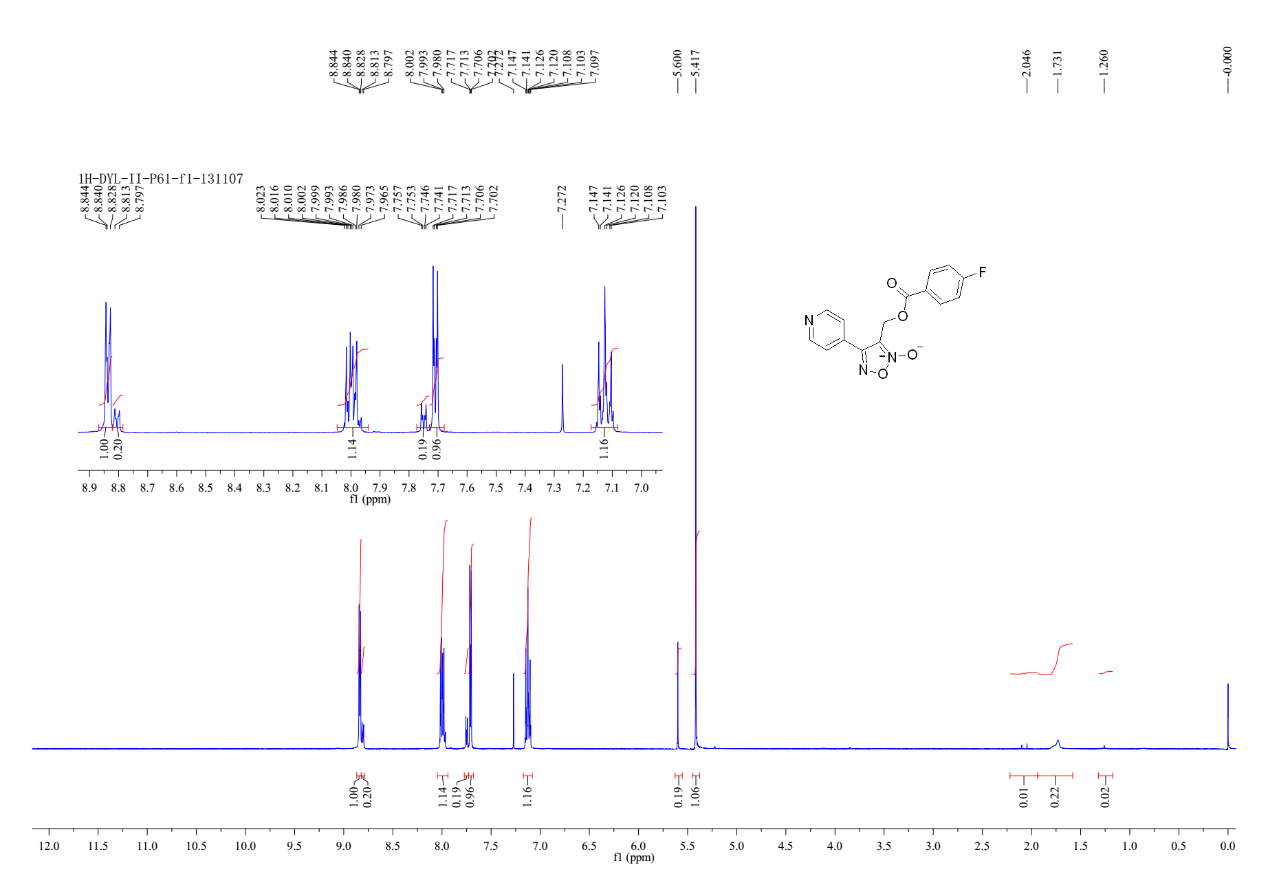


^1^H-NMR (400 MHz, Chloroform-*d*) spectrum of **7ag**.


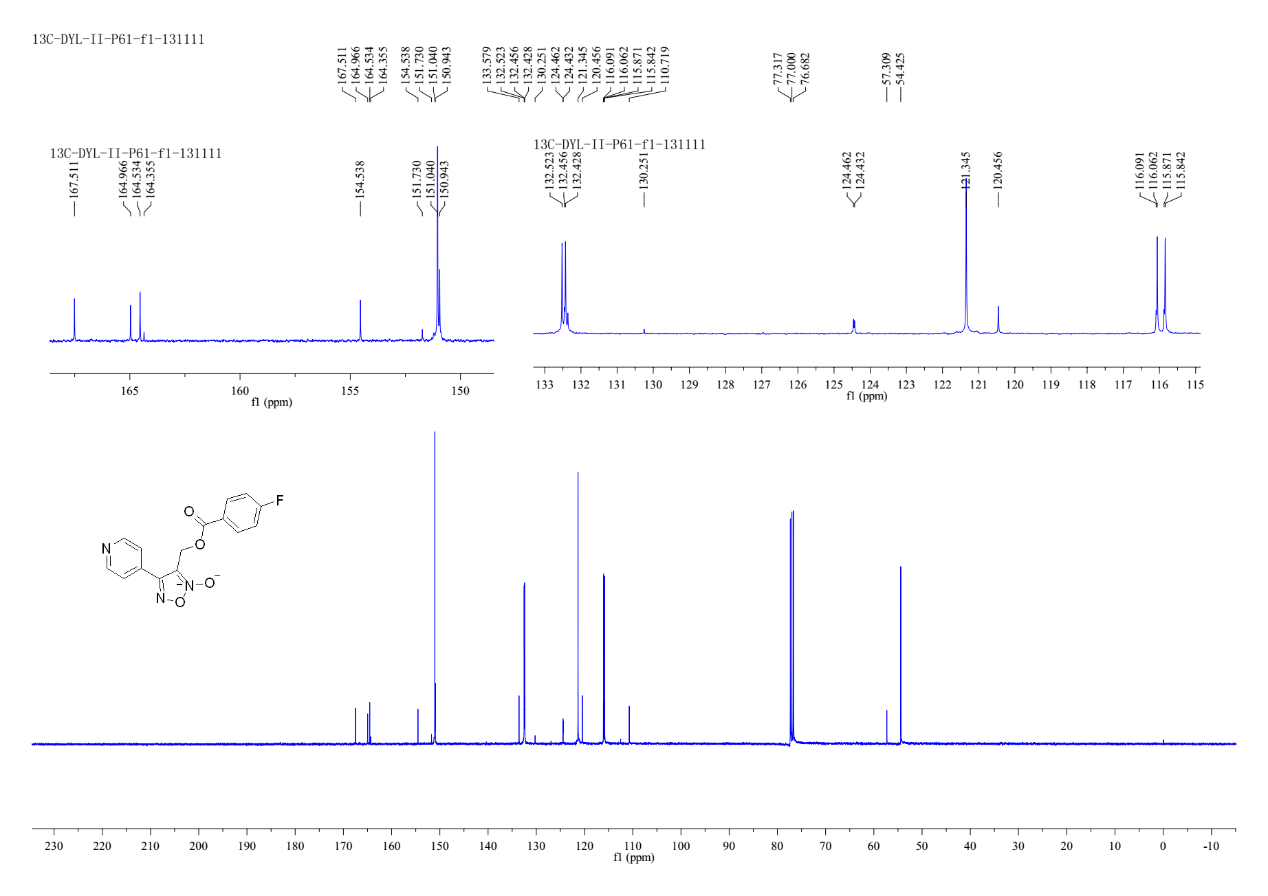


^13^C-NMR(400MHz, Chloroform-*d*) spectrum of **7ag**.


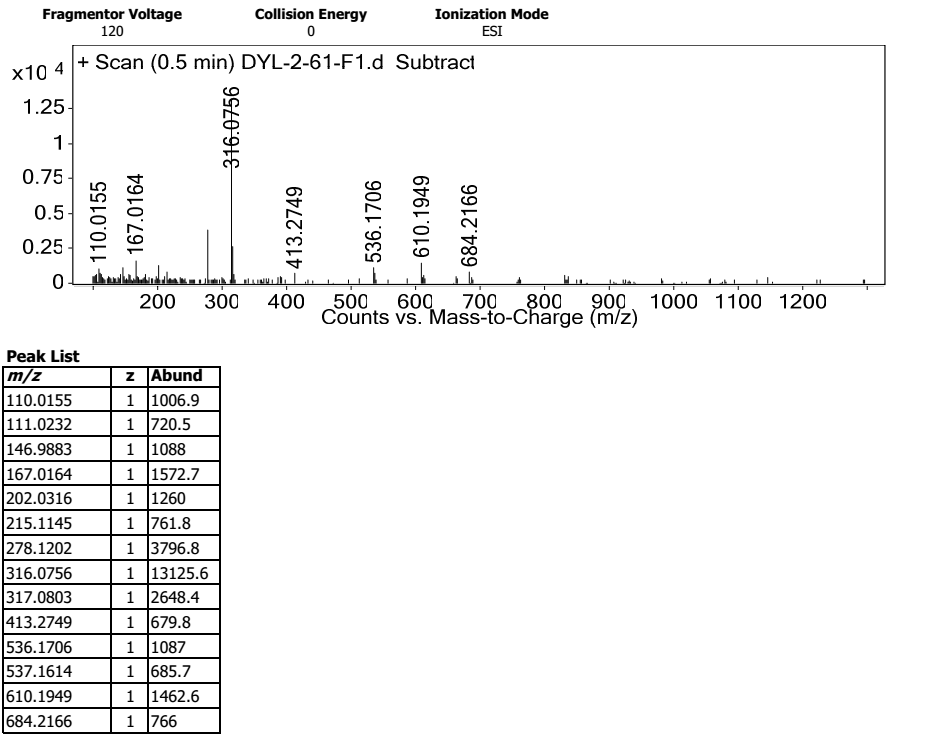


HRMS spectrum of **7ag**.


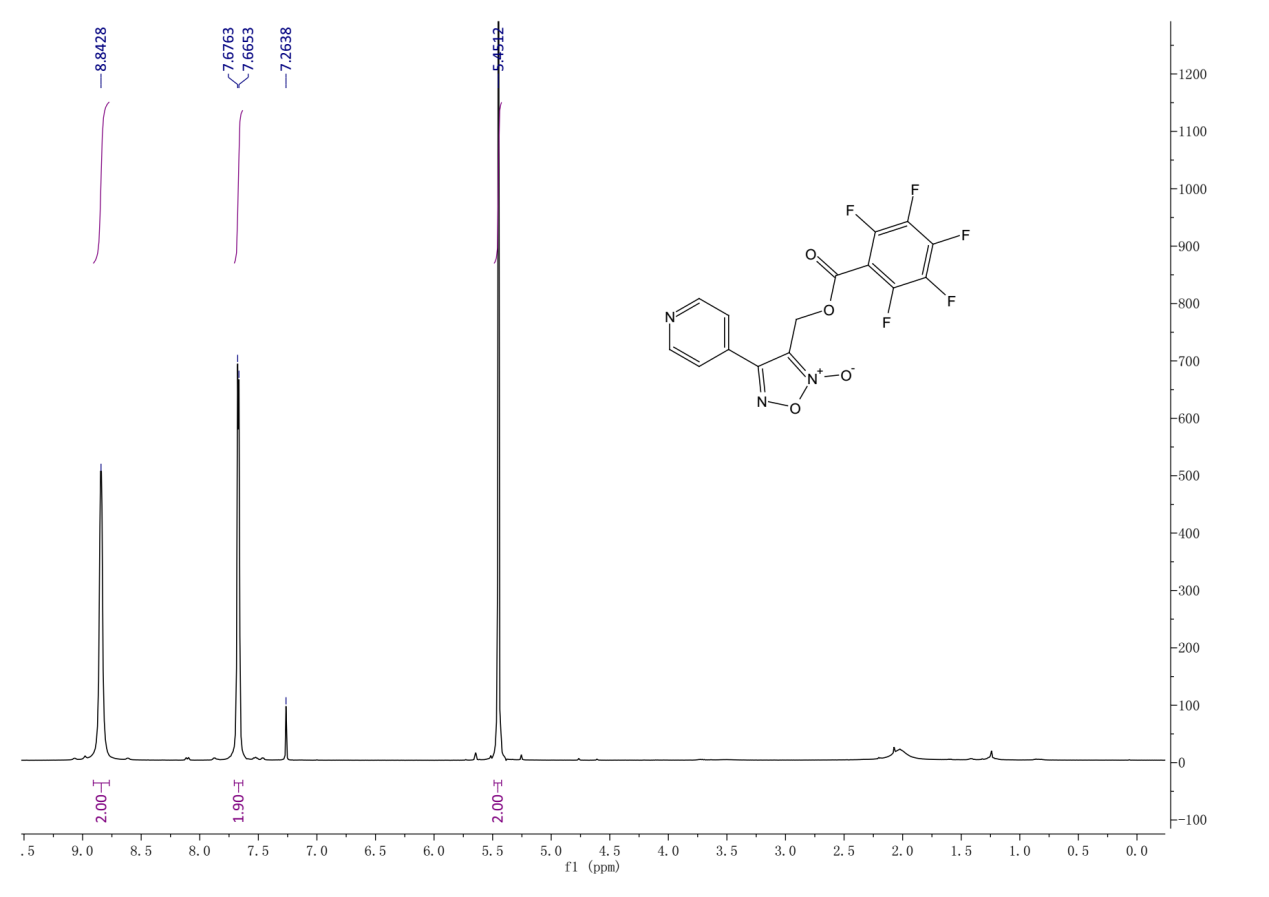


^1^H-NMR (400 MHz, Chloroform-*d*) spectrum of **7ah**.


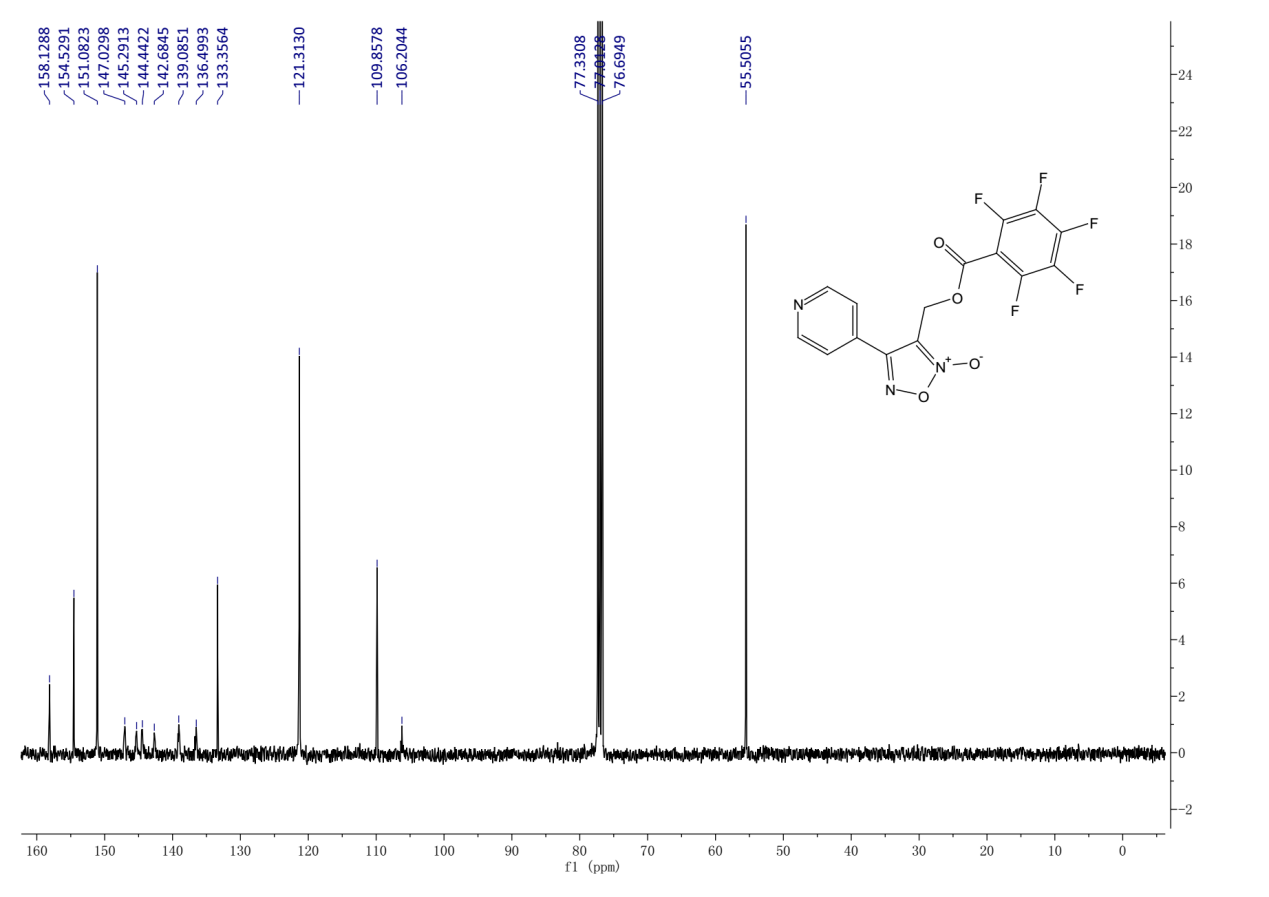
^13^CNMR (101 MHz, Chloroform-*d*) spectrum of **7ah**.


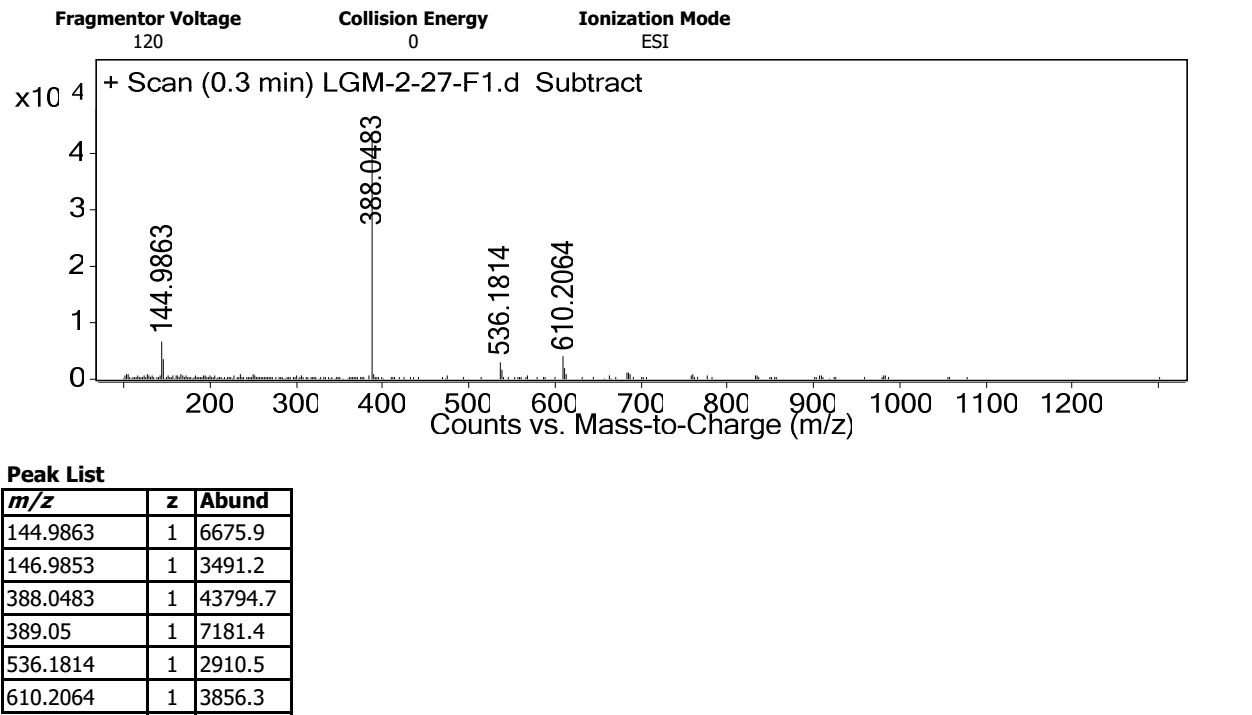


HRMS spectrum of **7ah**.


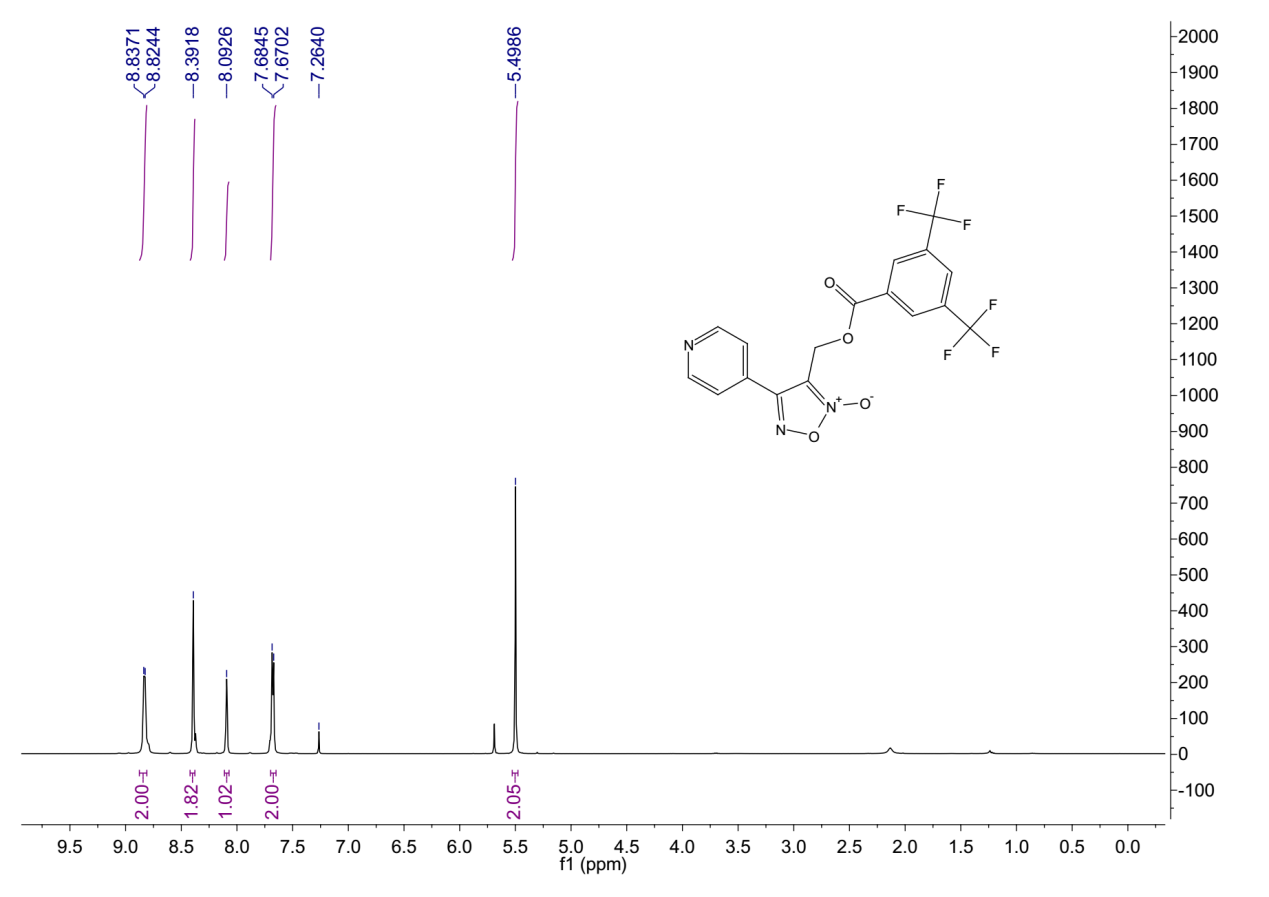


^1^H-NMR (400 MHz, Chloroform-*d*) spectrum of **7ai**.


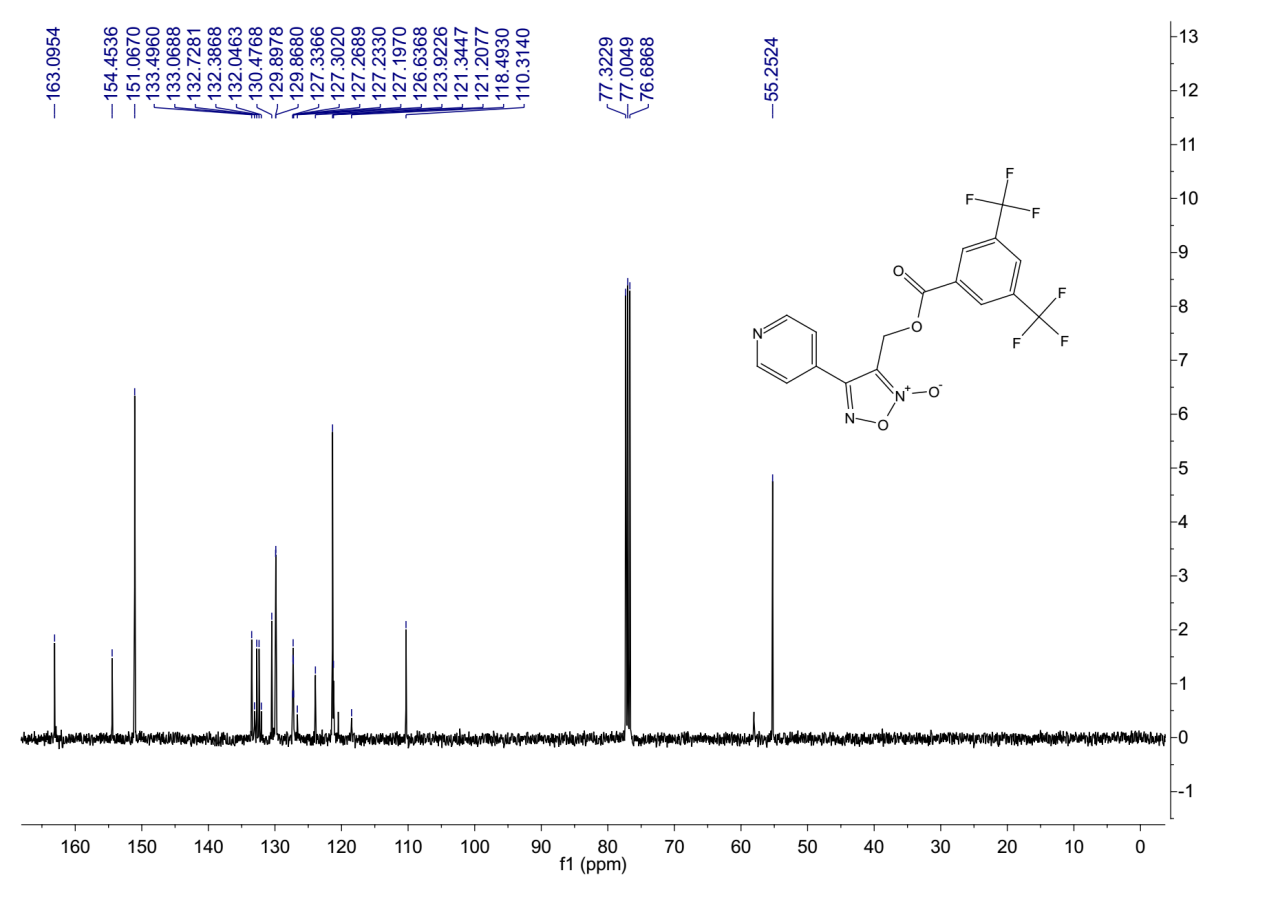


^13^C-NMR (101 MHz, Chloroform-*d*) spectrum of **7ai**.


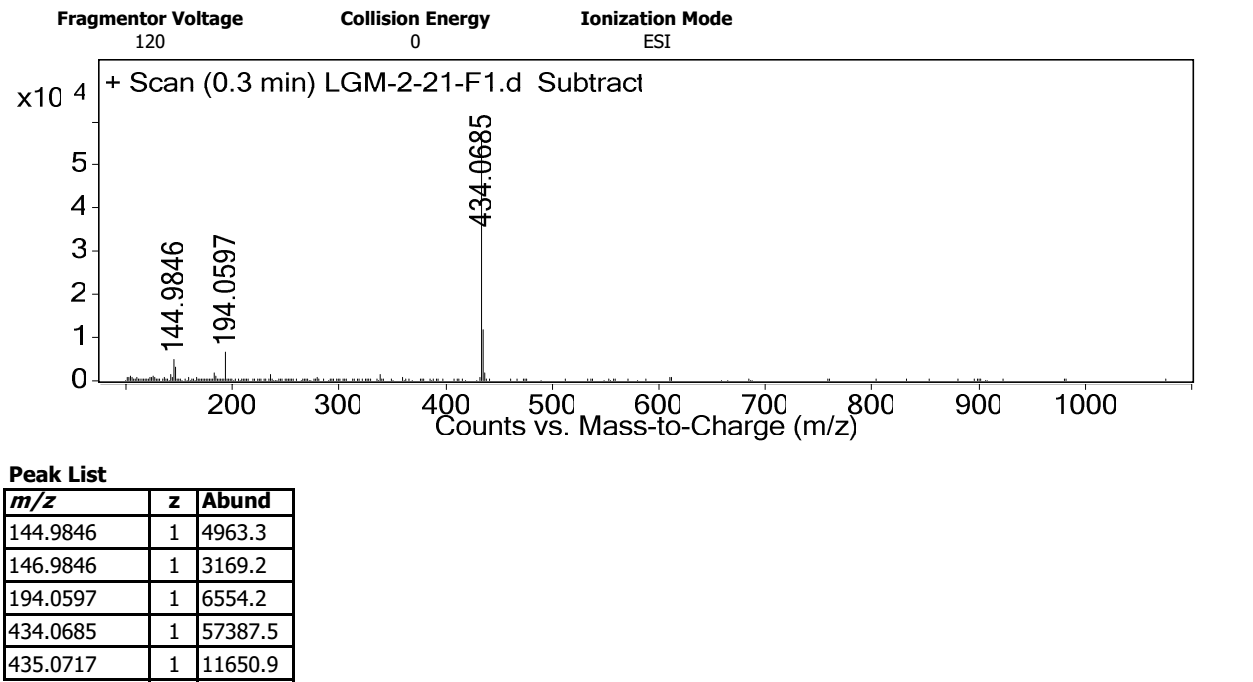


HRMS spectrum of **7ai**.


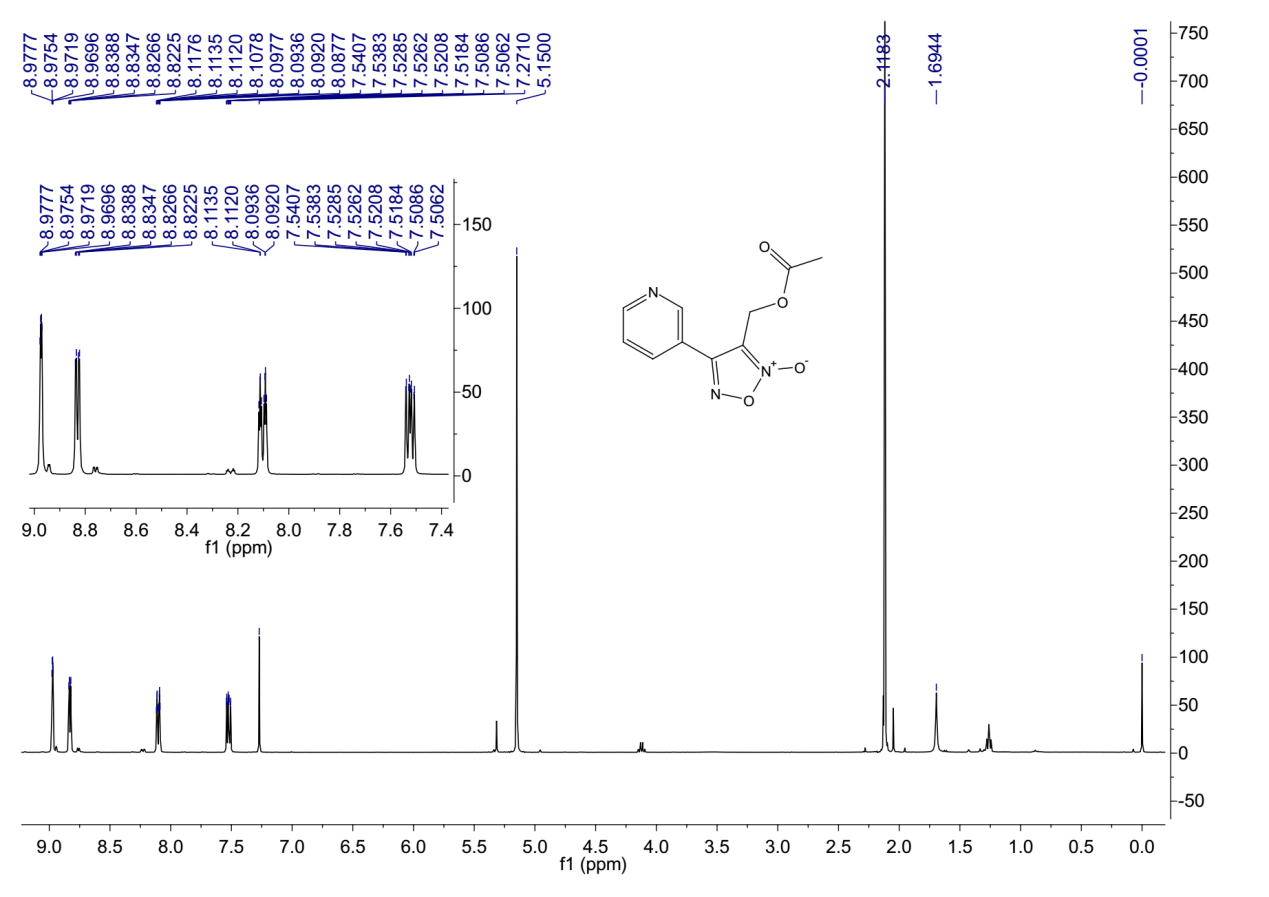


^1^H-NMR (400 MHz, Chloroform-*d*) spectrum of **7ba**.


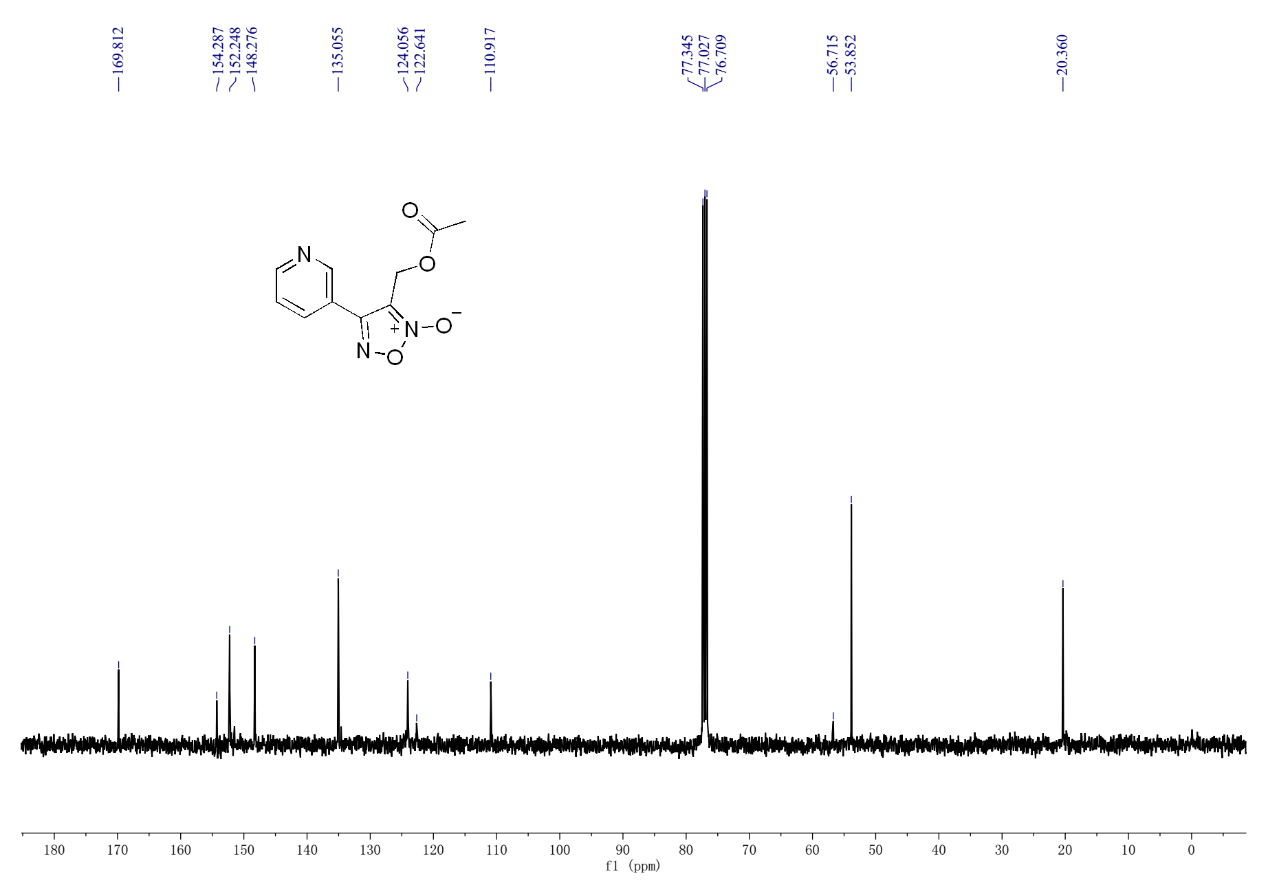


^13^C-NMR (101 MHz, Chloroform-*d*) spectrum of **7ba**.


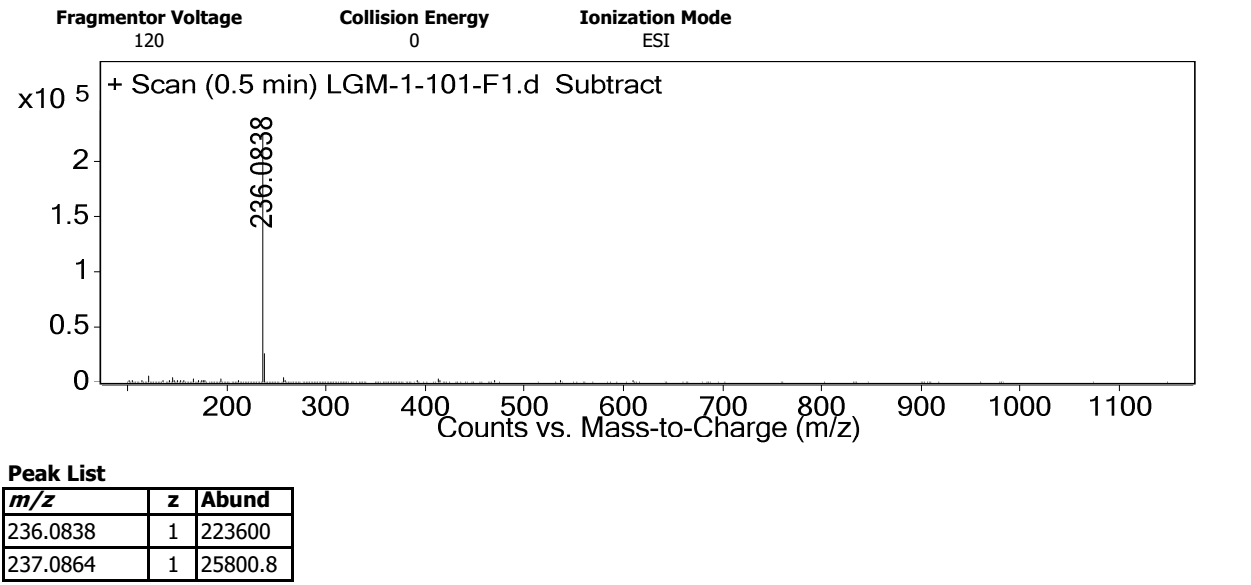


HRMS spectrum of **7ba**.


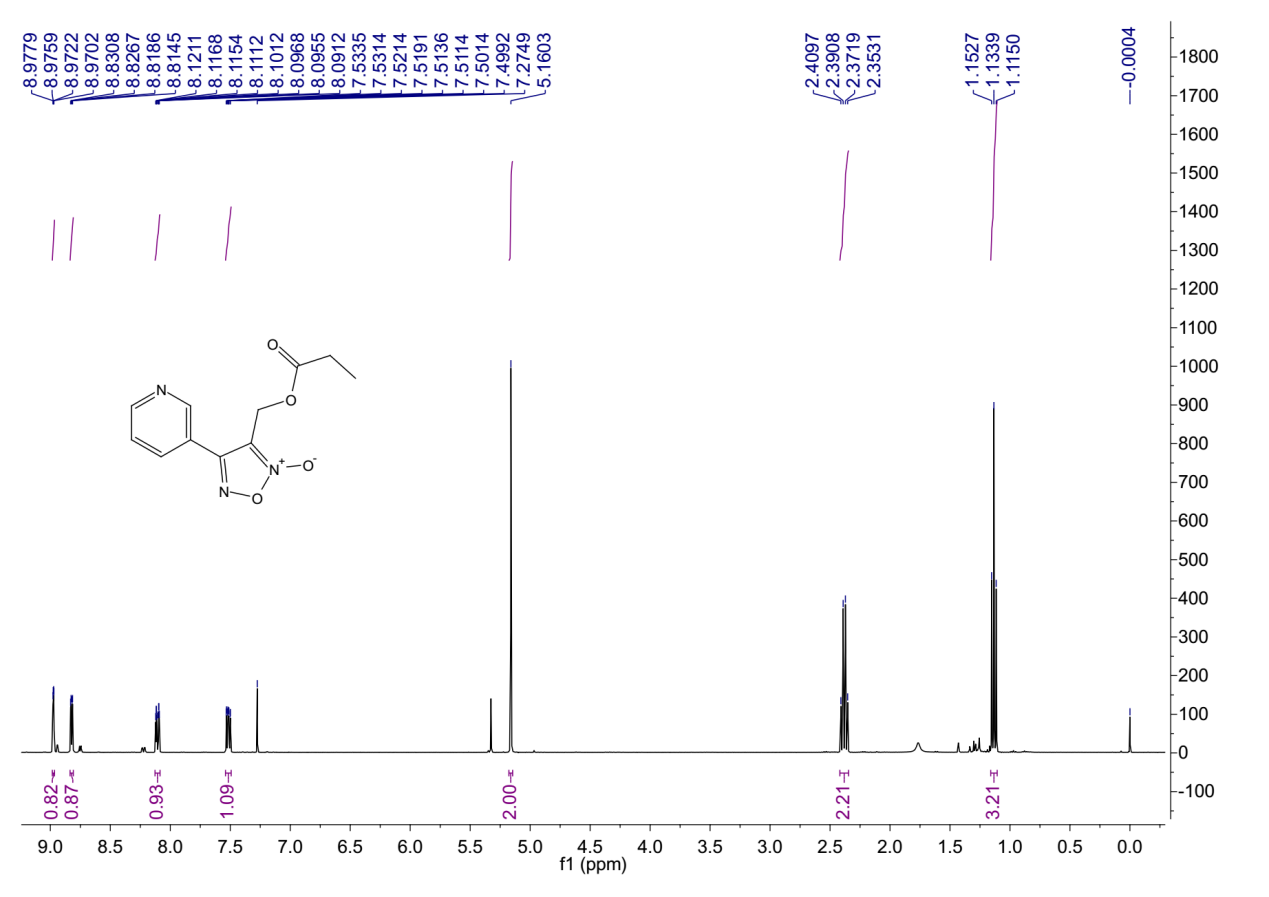


^1^H-NMR (400 MHz, Chloroform-*d*) spectrum of **7bb**.


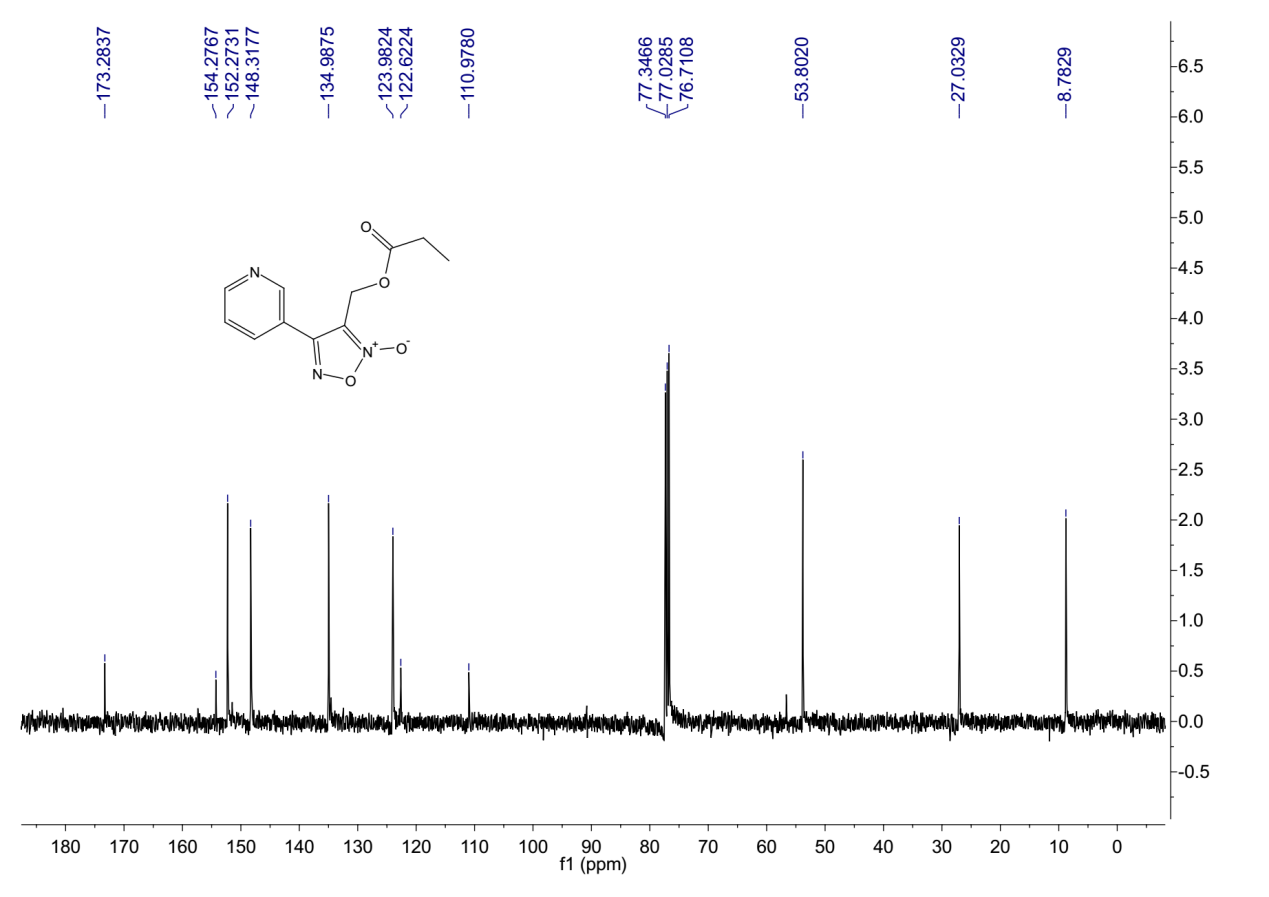


^13^C-NMR (101 MHz, Chloroform-*d*) spectrum of **7bb**.


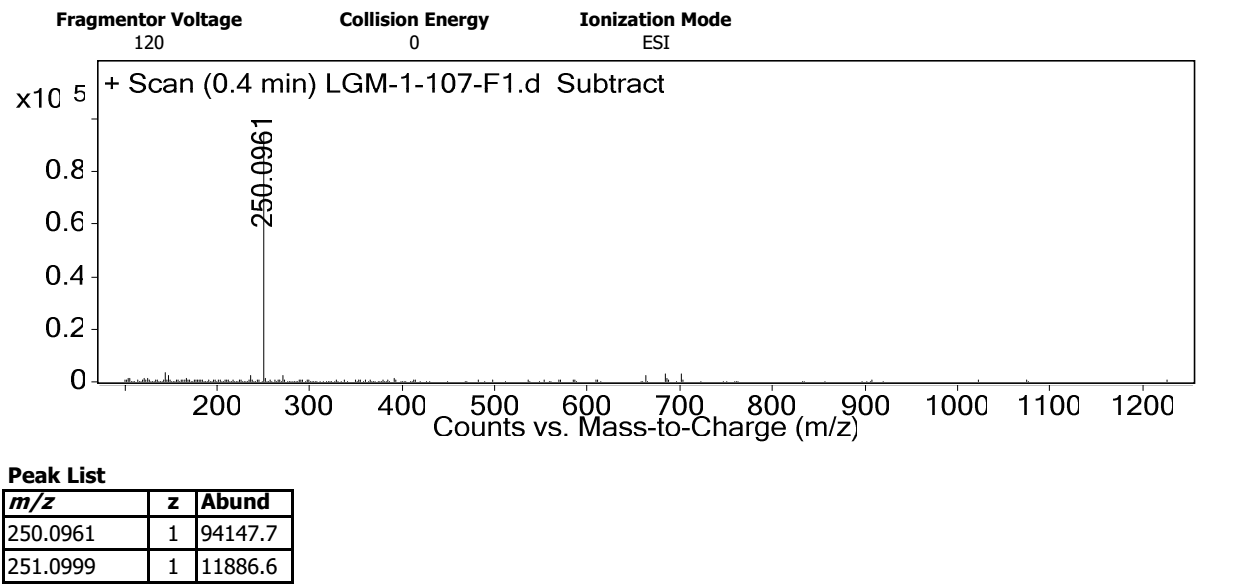


HRMS spectrum of **7bb**.


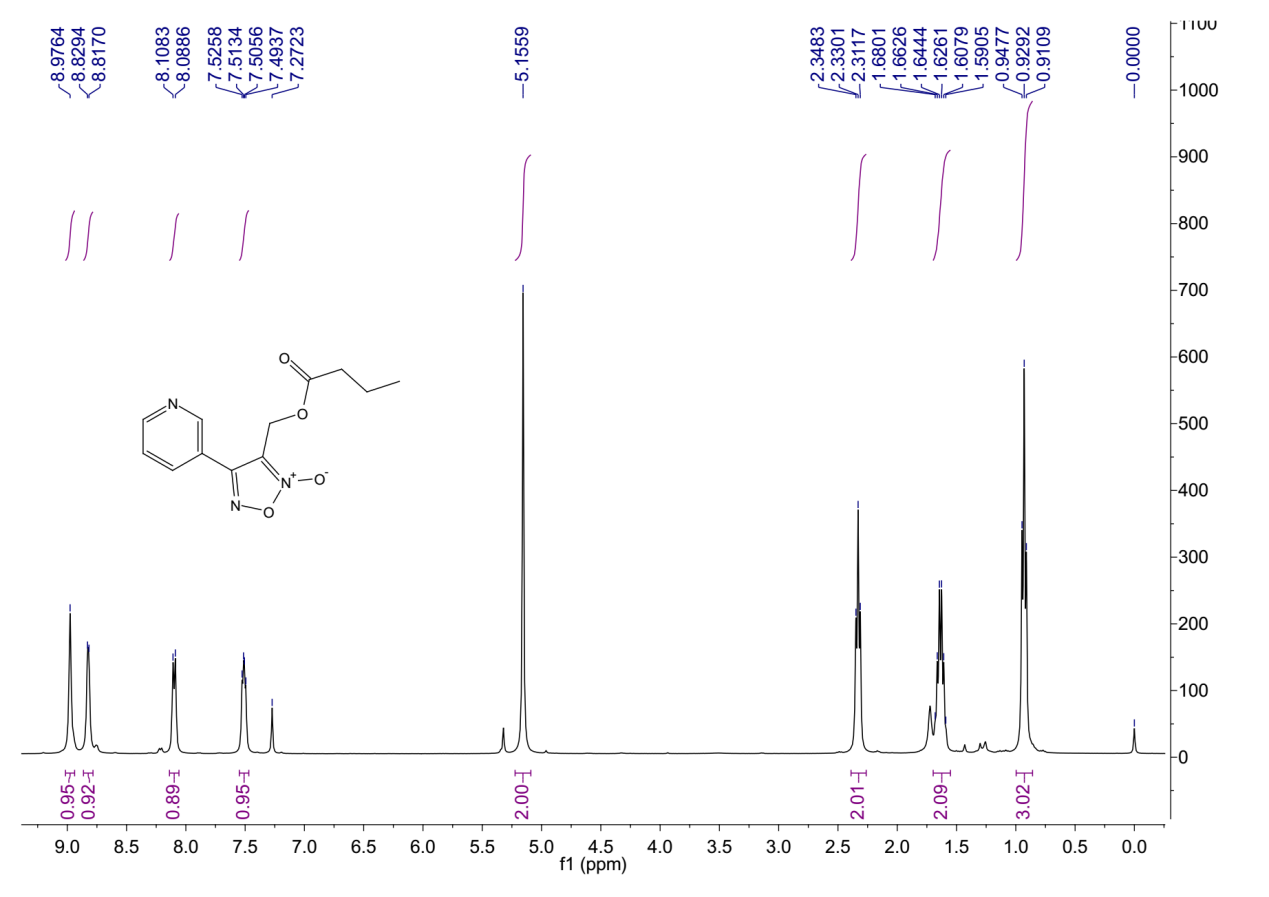


^1^H-NMR (400 MHz, Chloroform-*d*) spectrum of **7bc**.


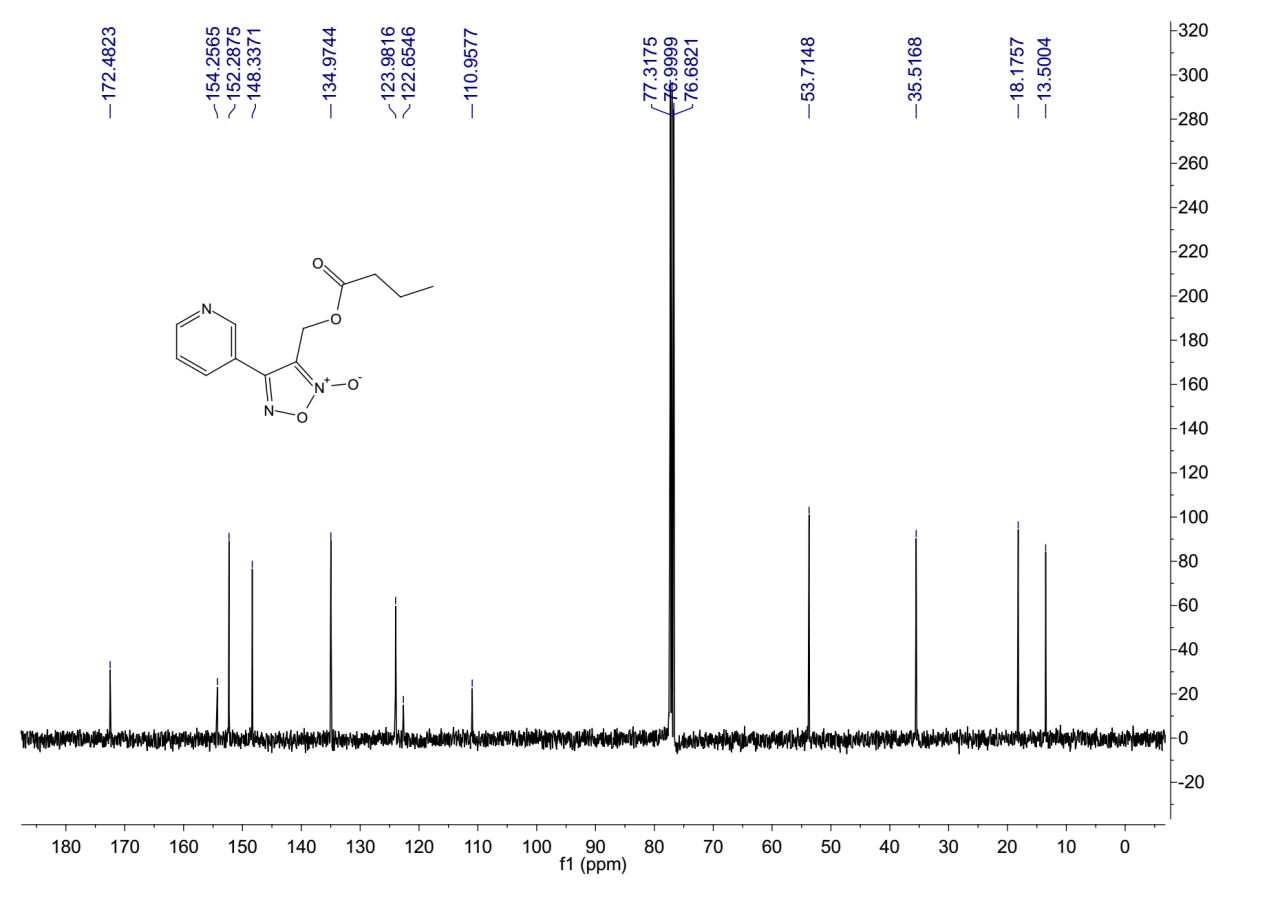


^13^C-NMR (101 MHz, Chloroform-*d*) spectrum of **7bc**.


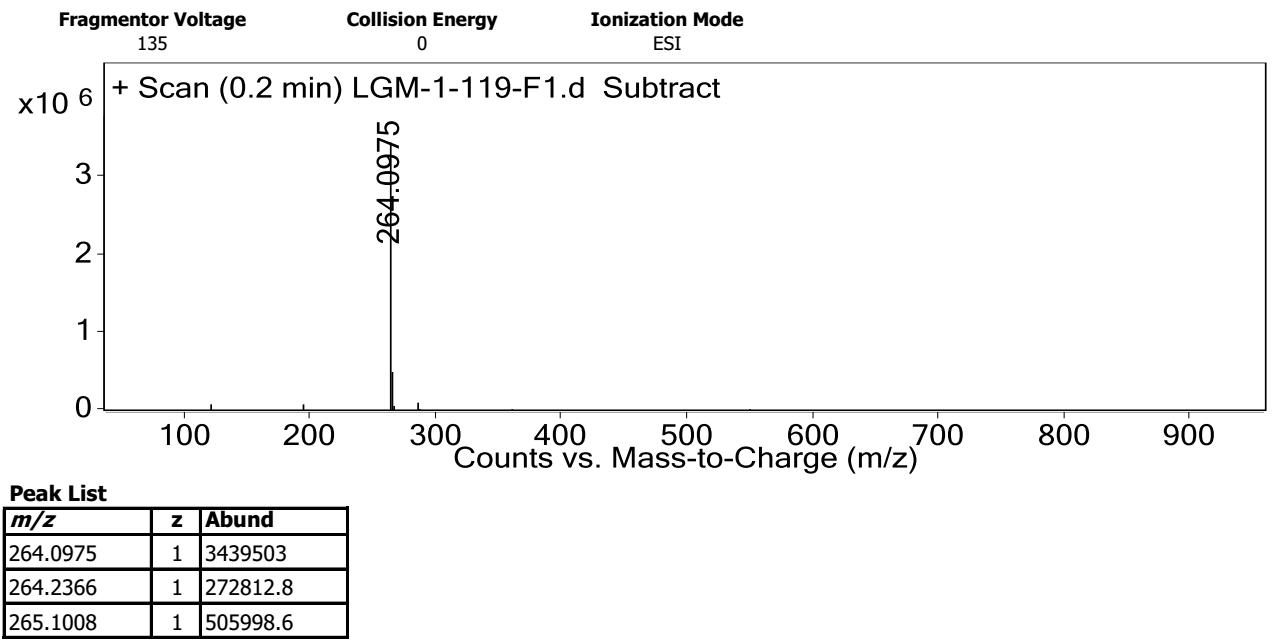


HRMS spectrum of **7bc**.


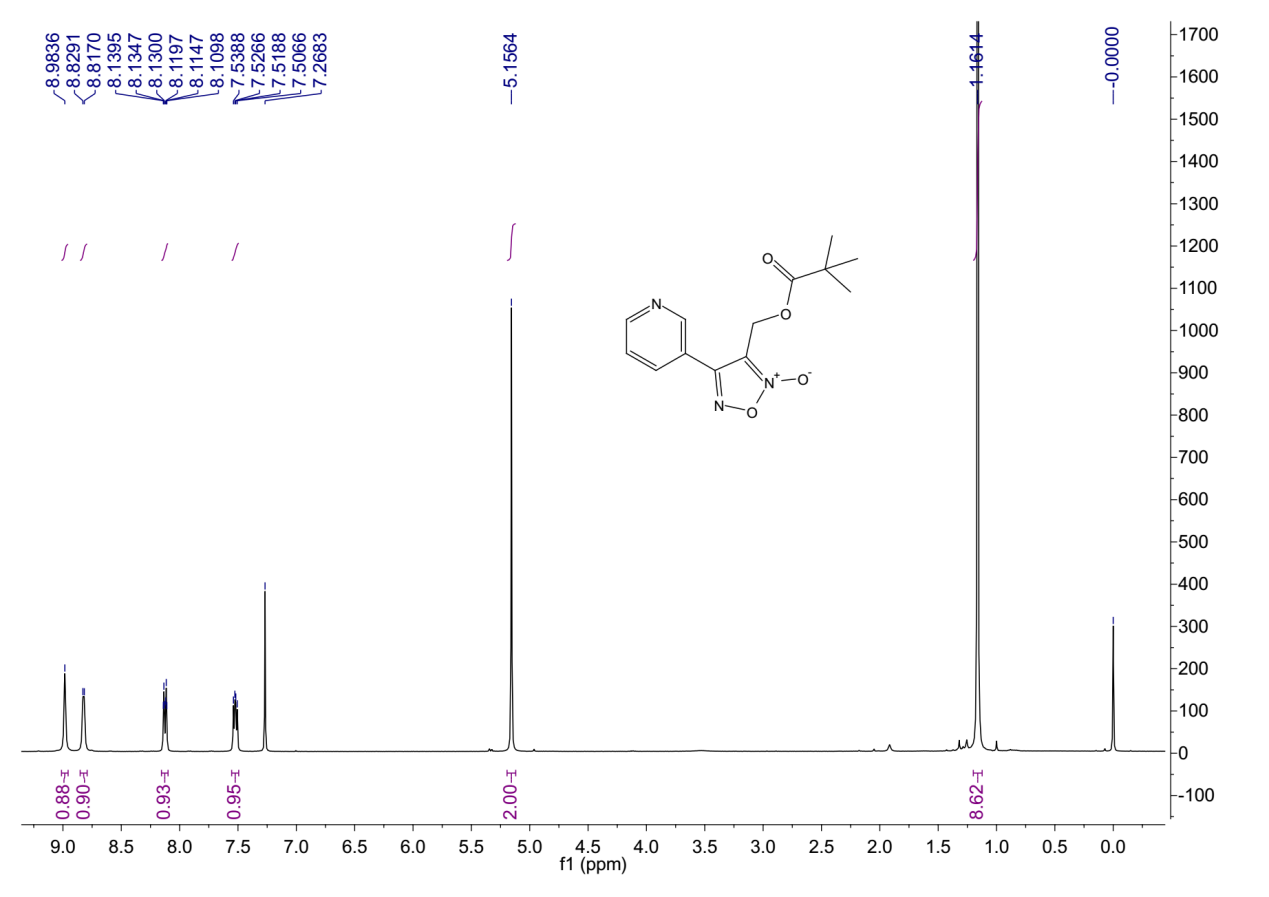


^1^H-NMR (400 MHz, Chloroform-*d*) spectrum of **7bd**.


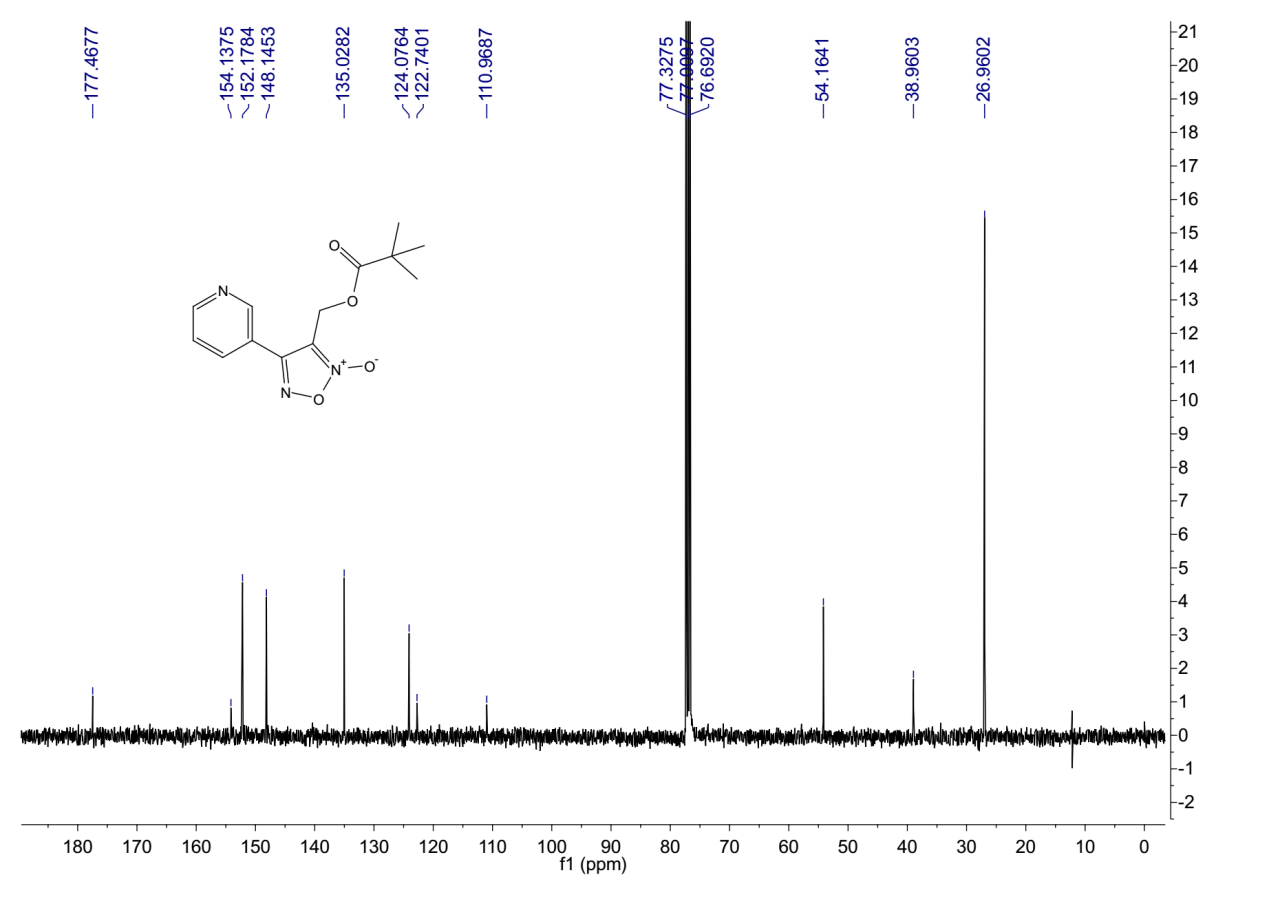


^13^C-NMR (101 MHz, Chloroform-*d*) spectrum of **7bd**.


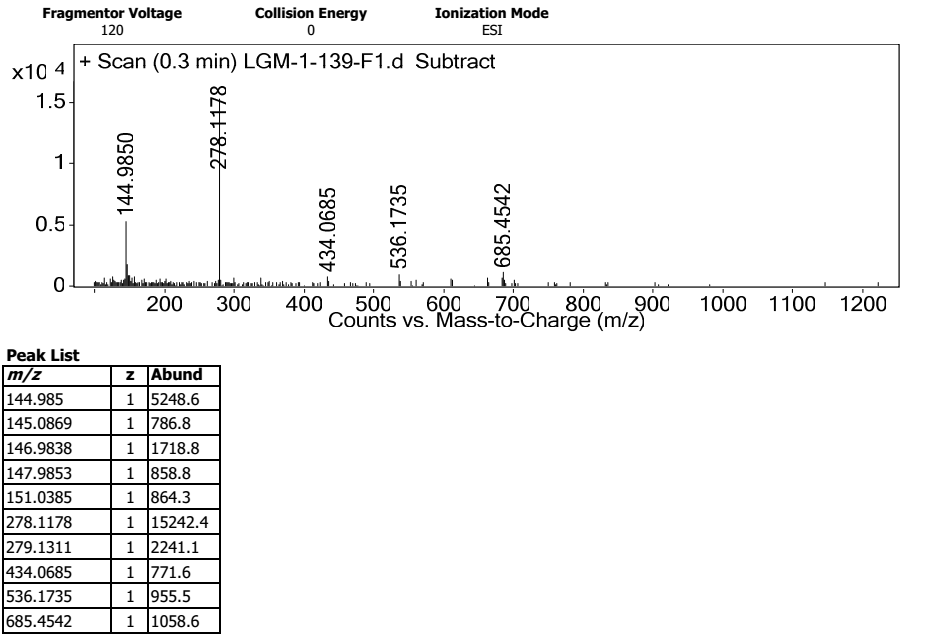


HRMS spectrum of **7bd**.


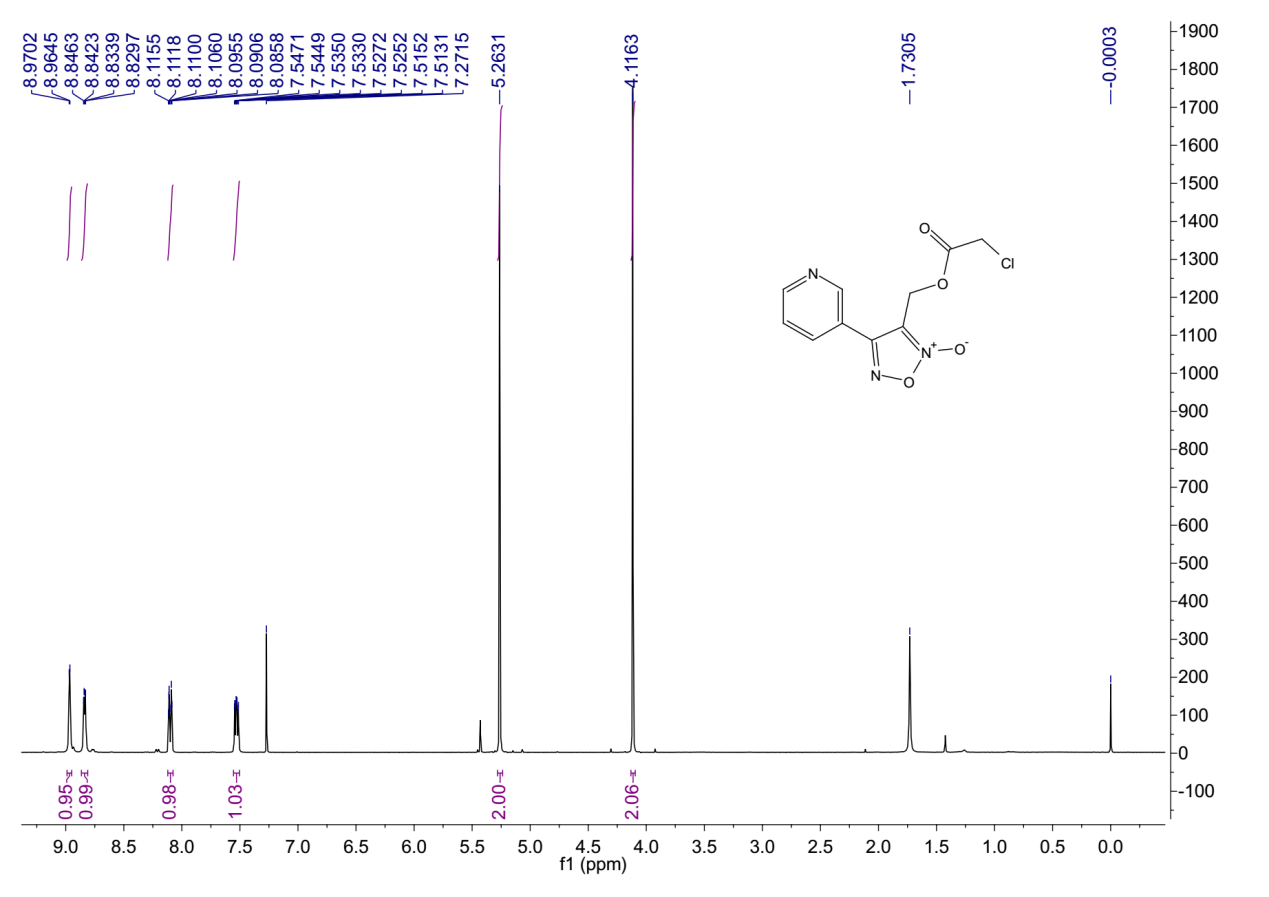


^1^H-NMR (400 MHz, Chloroform-*d*) spectrum of **7be**.


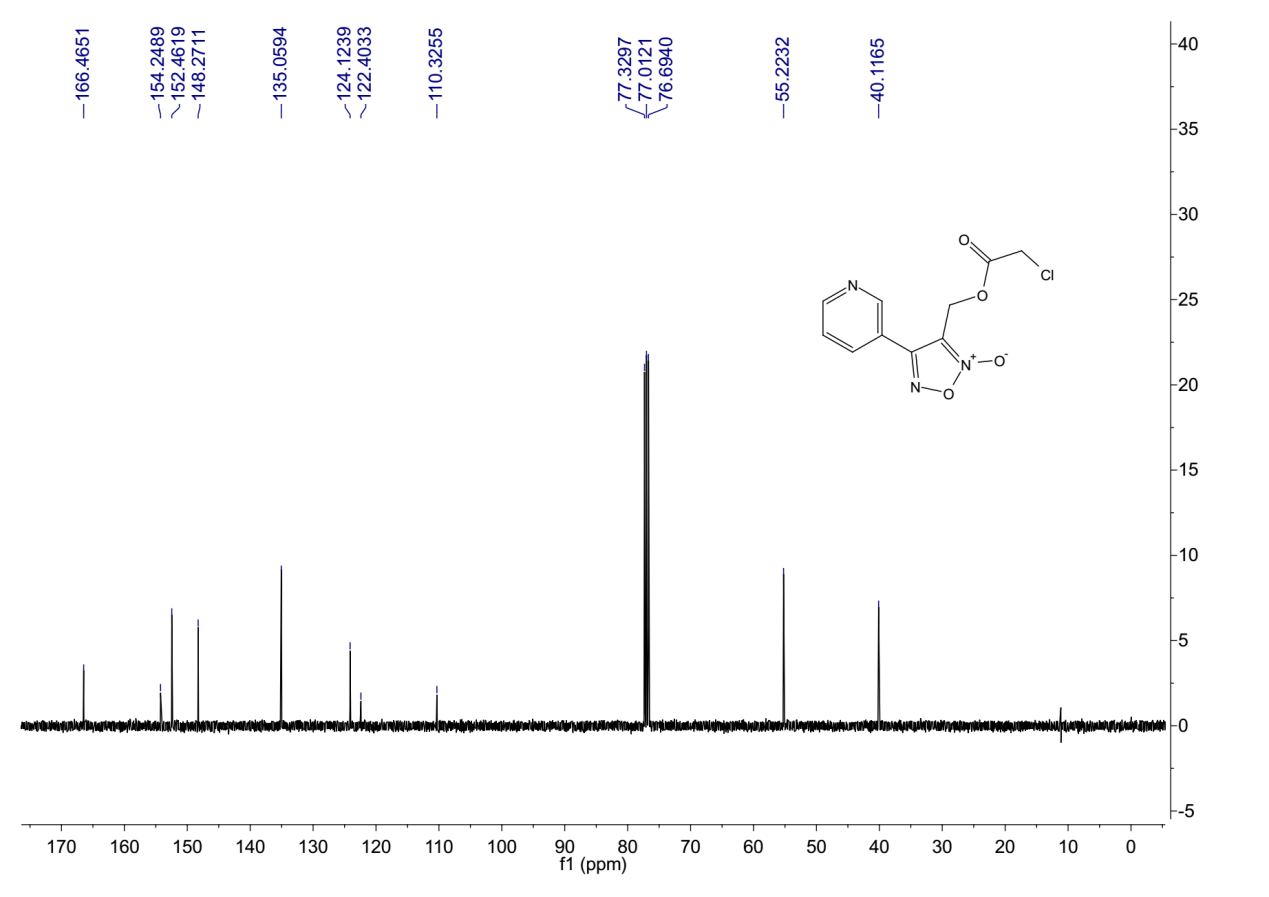


^13^C-NMR (101 MHz, Chloroform-*d*) spectrum of **7be**.


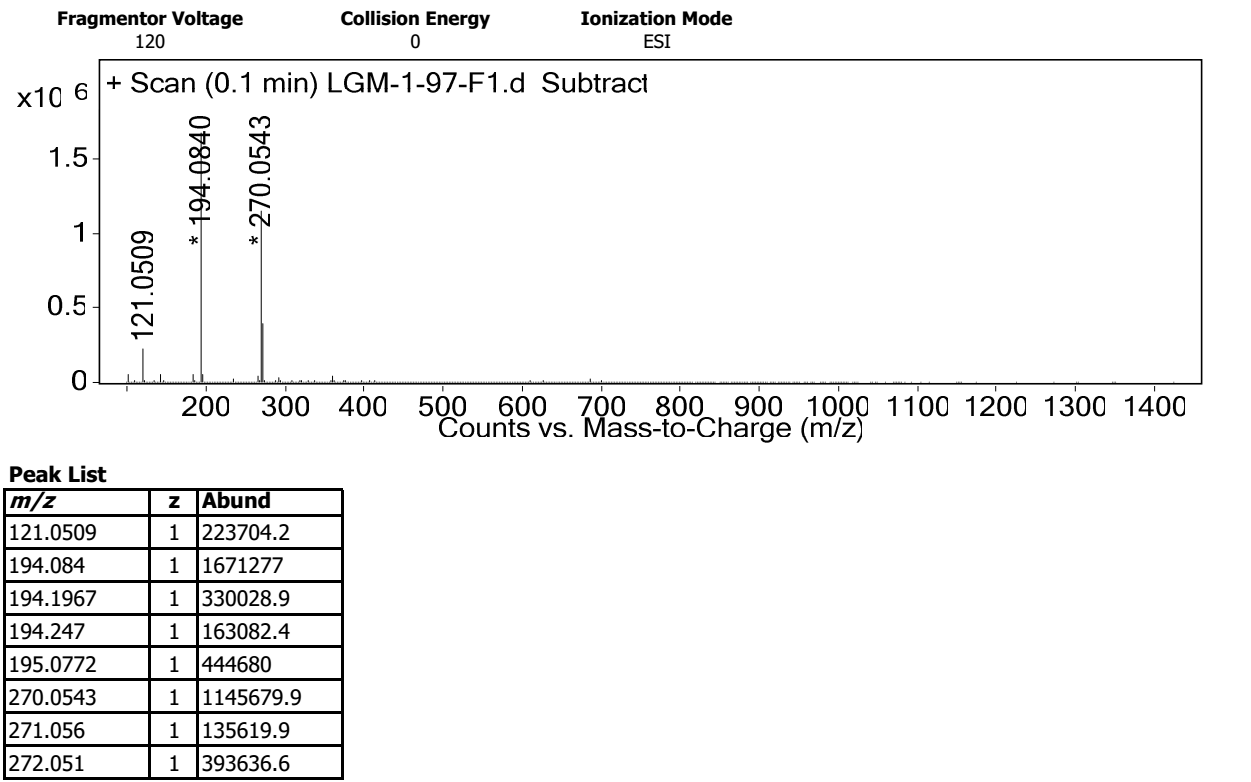


HRMS spectrum of **7be**.


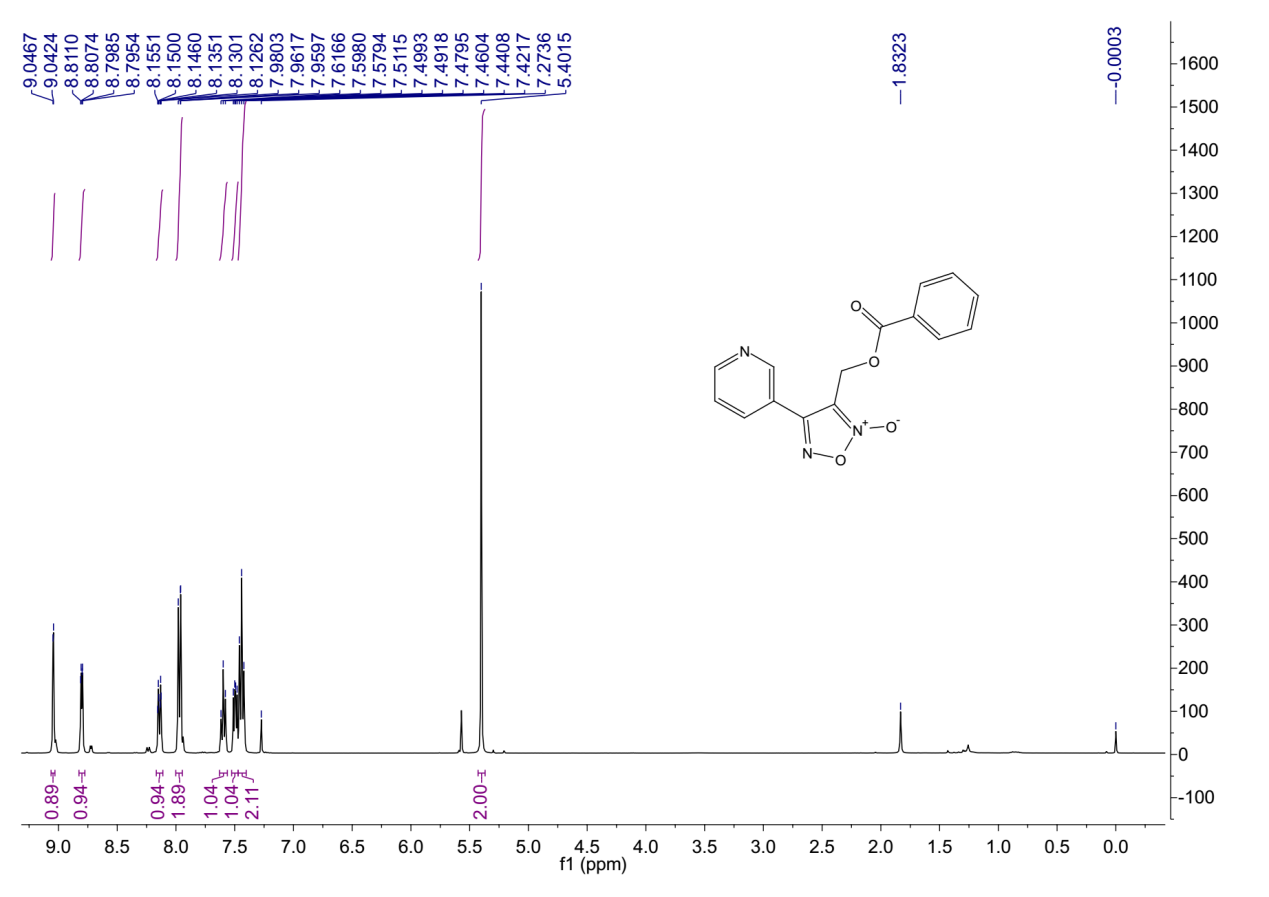


^1^H-NMR (400 MHz, Chloroform-*d*) spectrum of **7bf**.


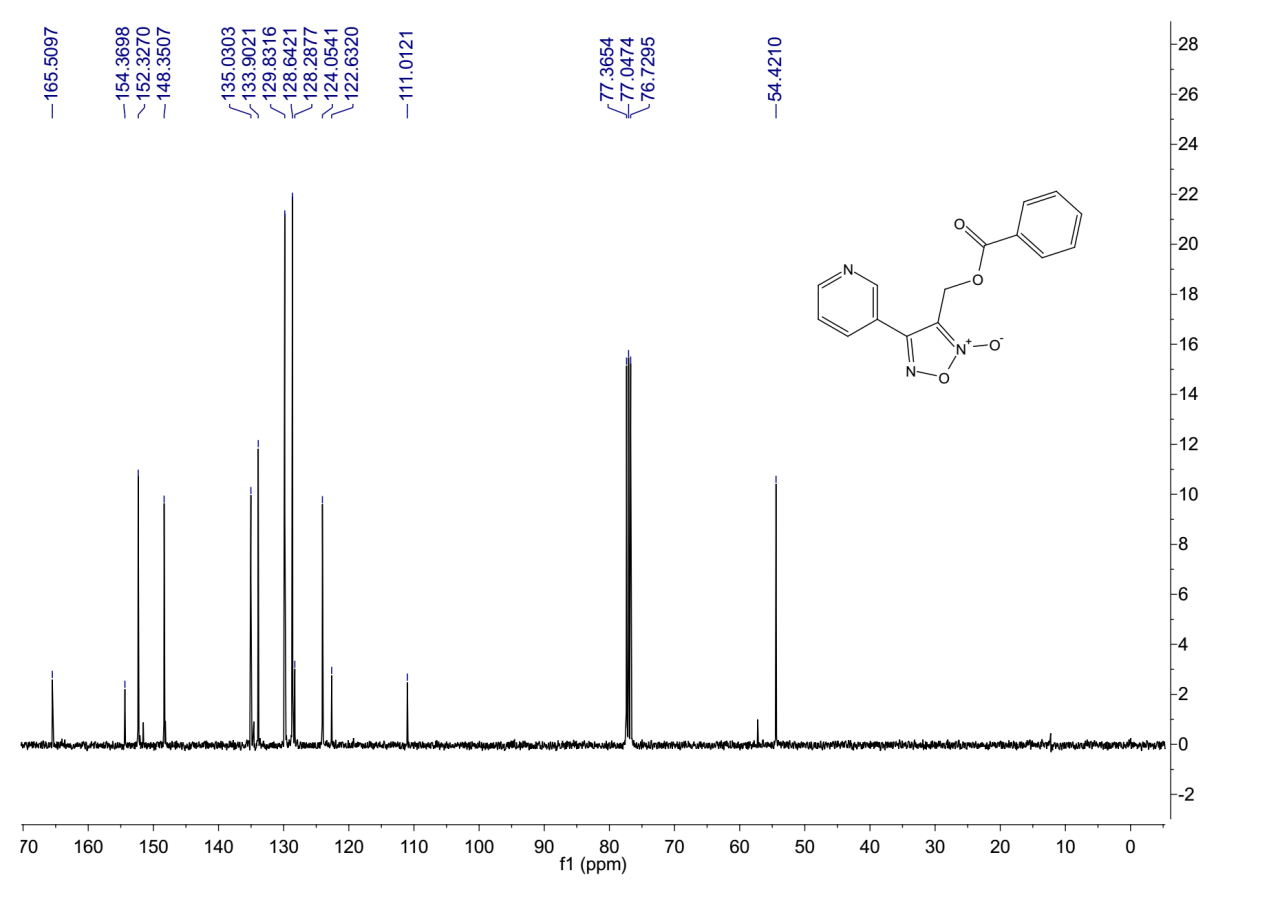


^13^C-NMR (101 MHz, Chloroform-*d*) spectrum of **7bf**.


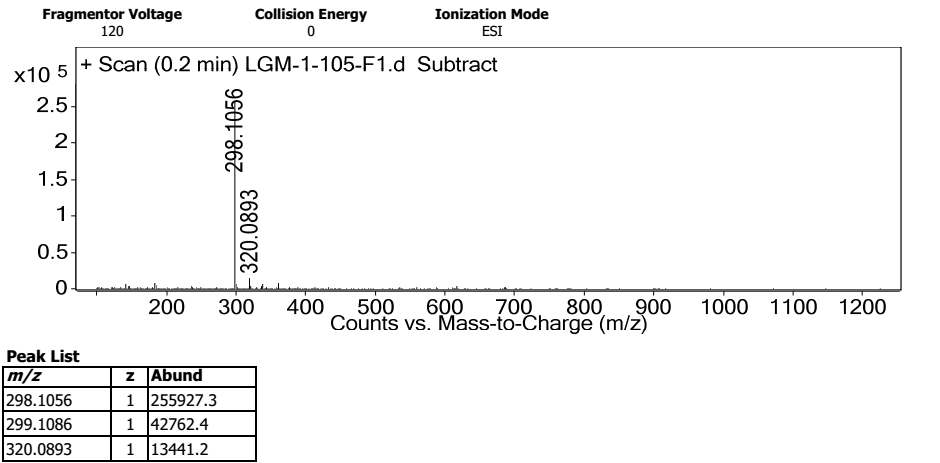


HRMS spectrum of **7bf**.


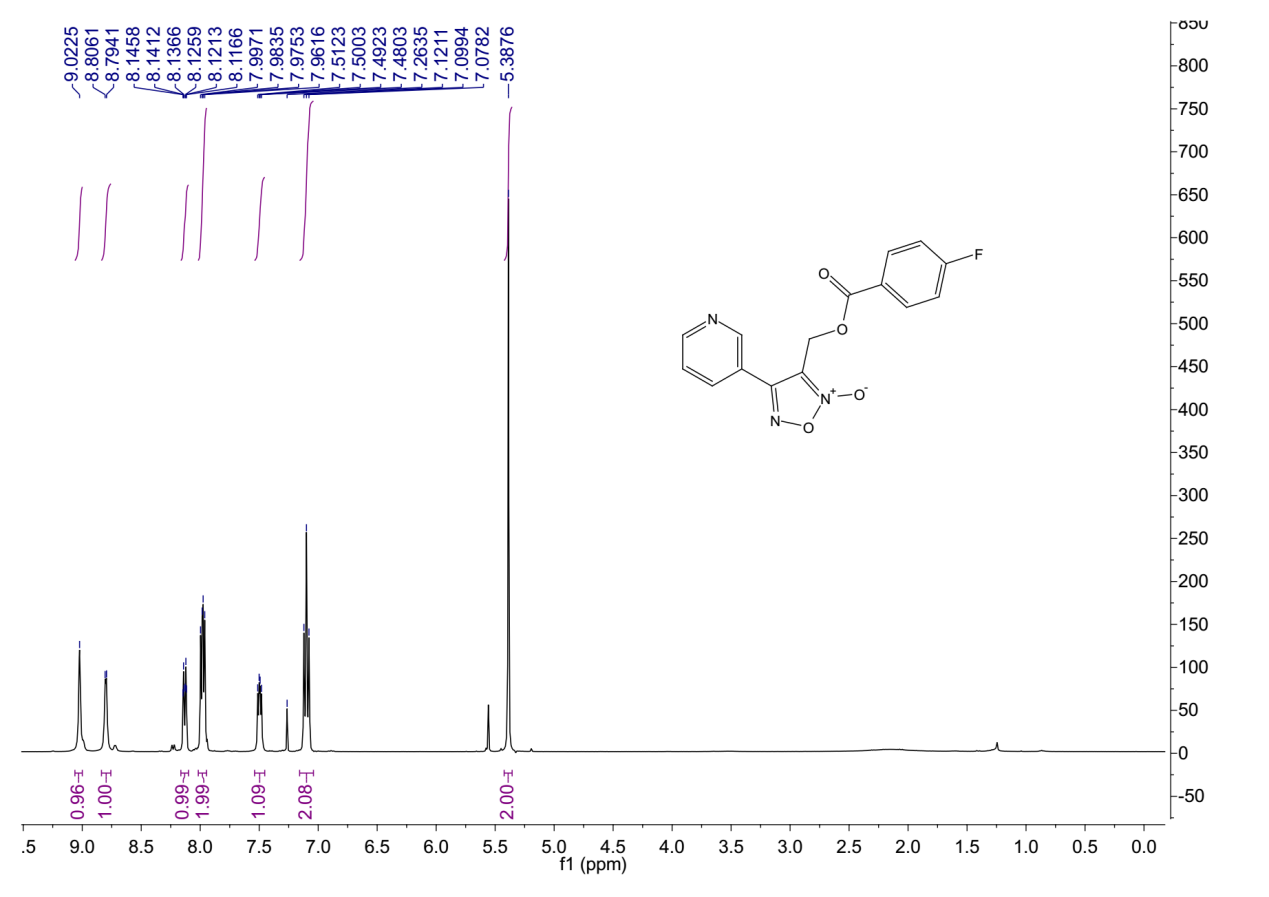


^1^H-NMR (400 MHz, Chloroform-*d*) spectrum of **7bg**.


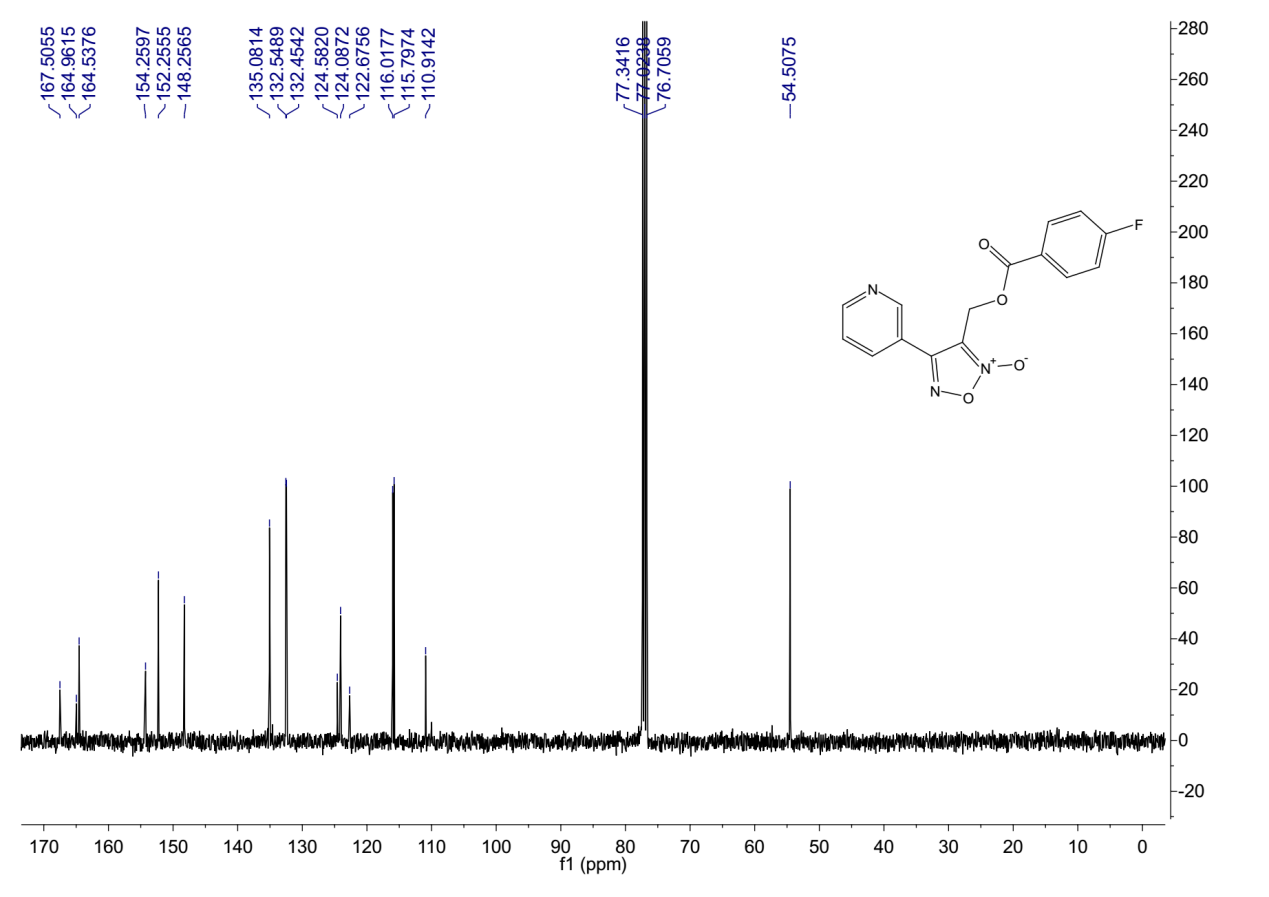


^13^C-NMR (101 MHz, Chloroform-d) spectrum of **7bg**.


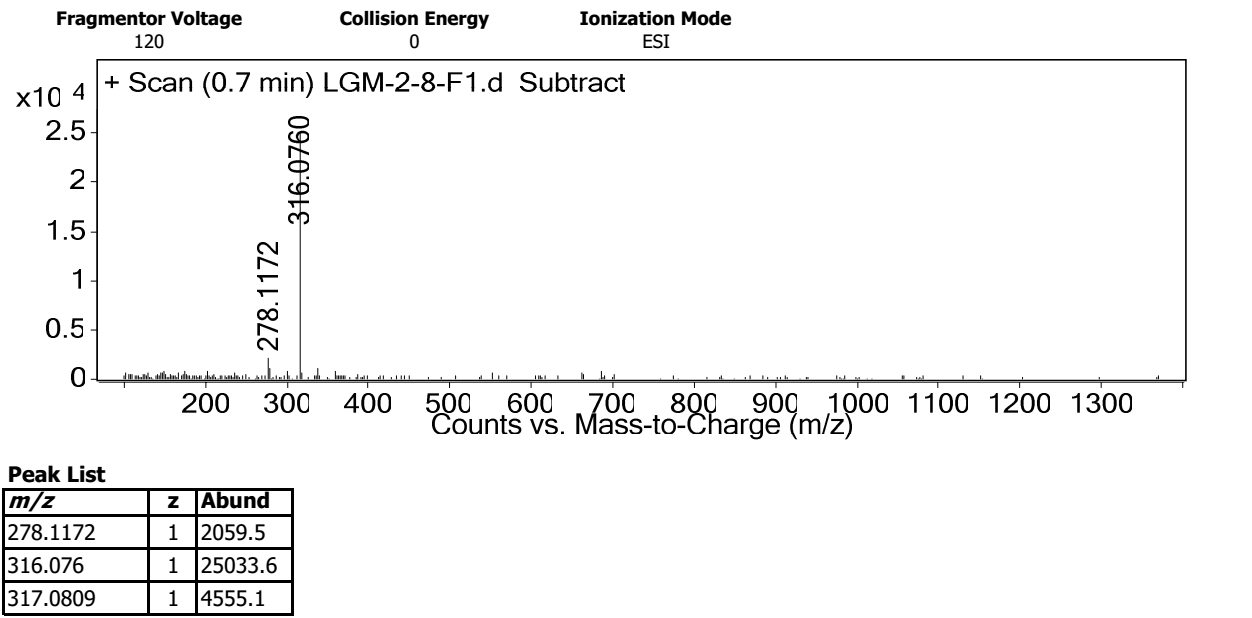


HRMS spectrum of **7bg**.


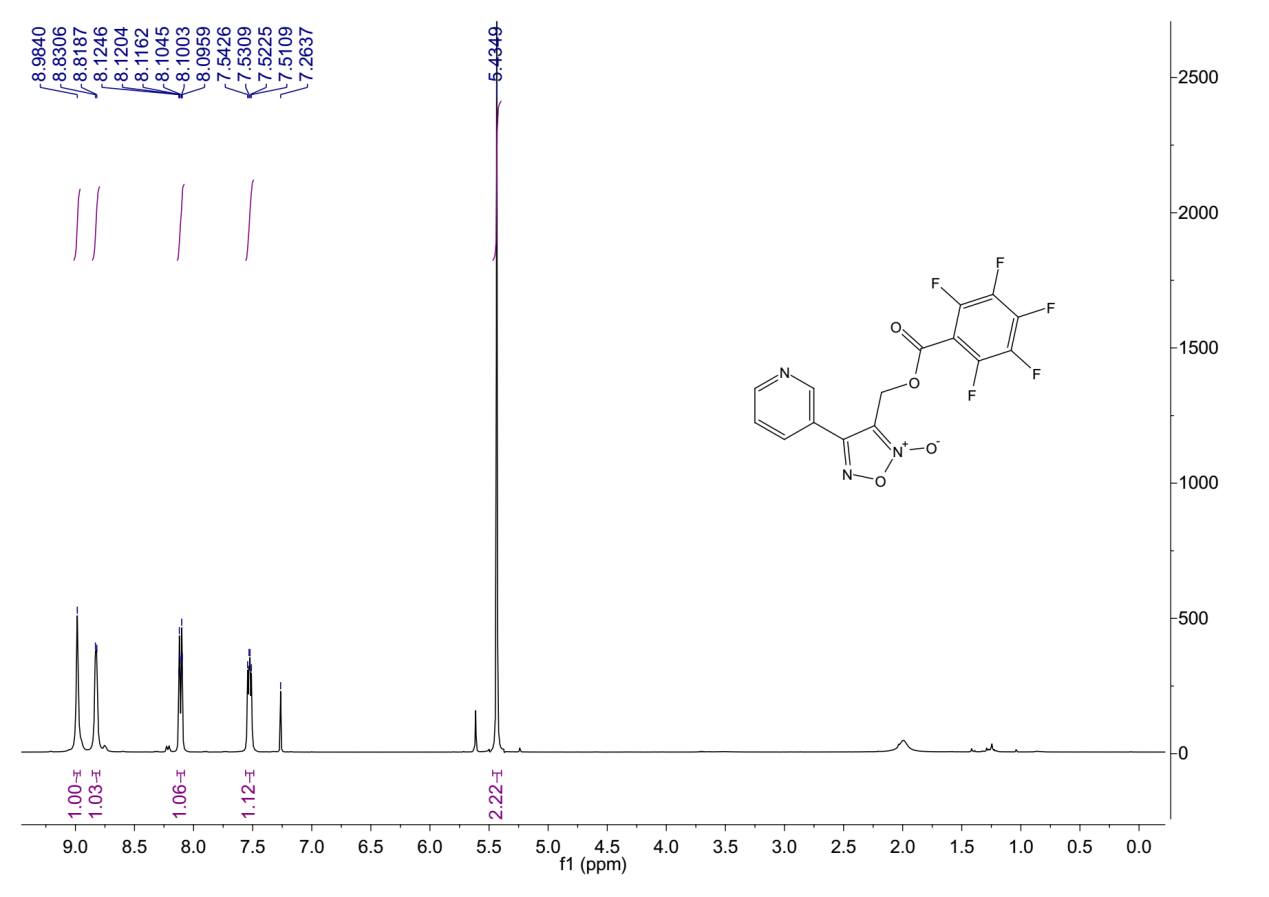


^1^H-NMR (400 MHz, Chloroform-*d*) spectrum of **7bh**.


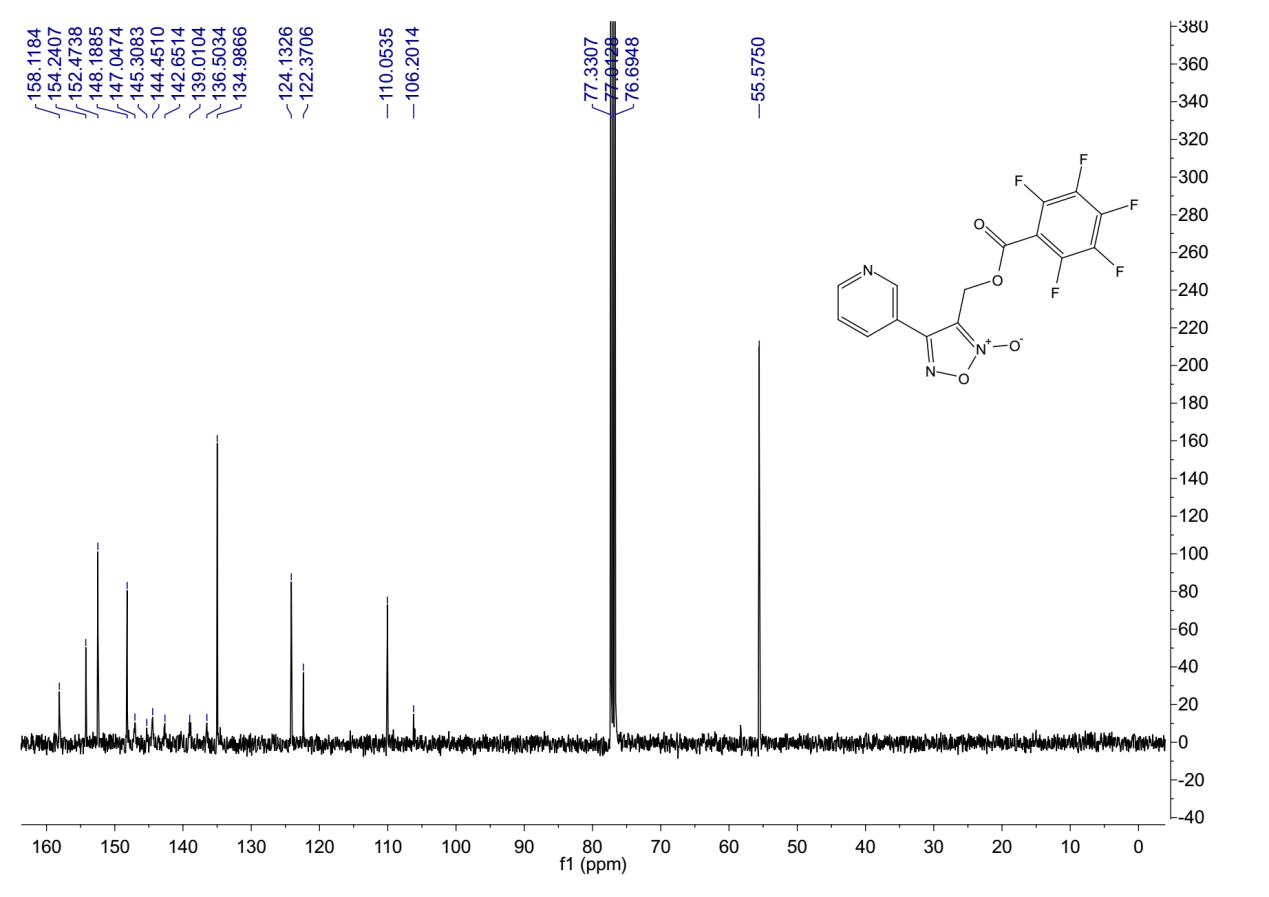


^13^C-NMR (101 MHz, Chloroform-*d*) spectrum of **7bh**.


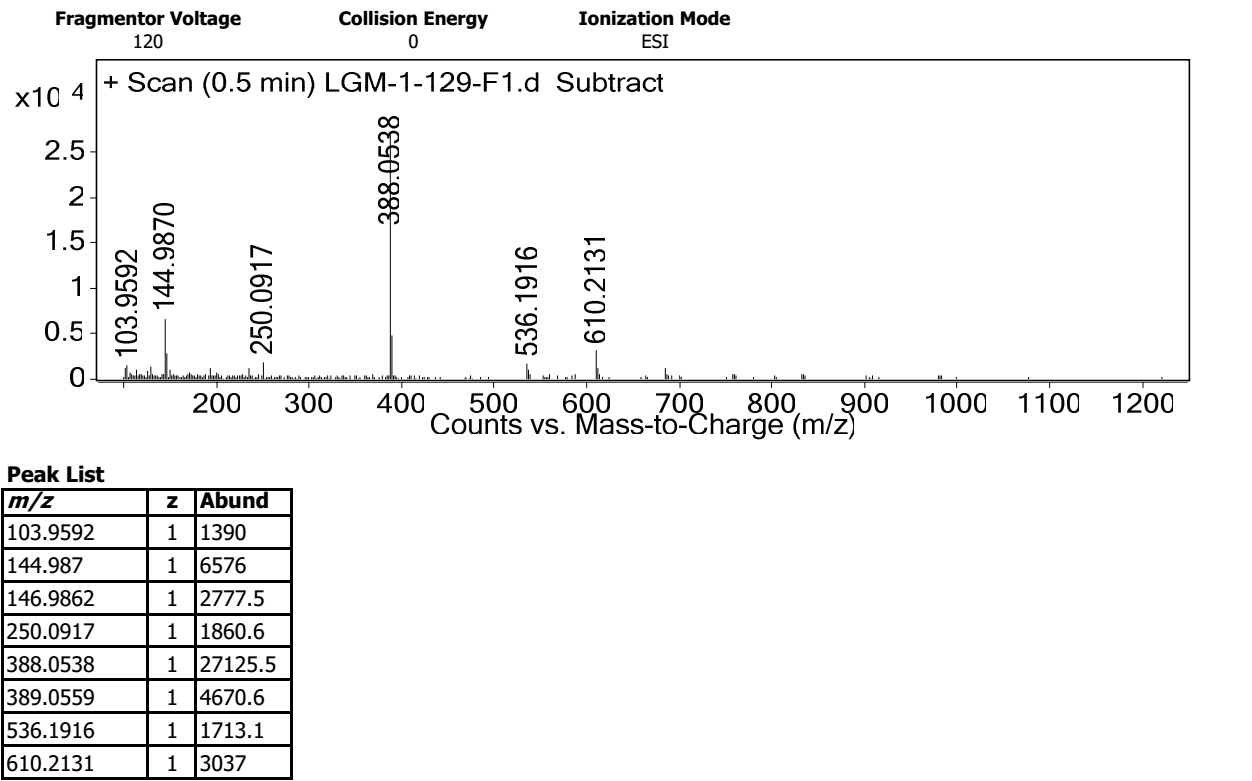


HRMS spectrum of **7bh**.


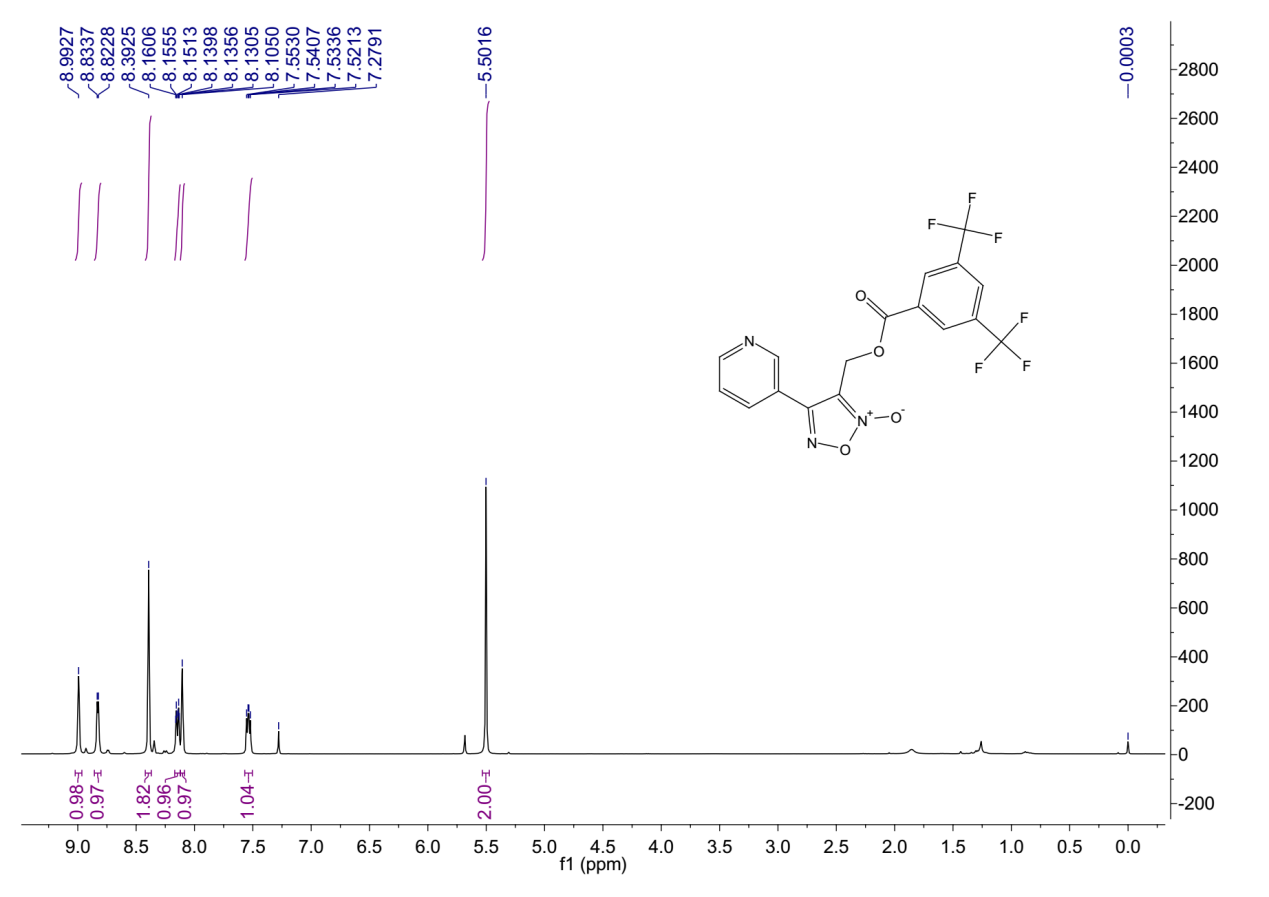


^1^H-NMR (400 MHz, Chloroform-*d*) spectrum of **7bi**.


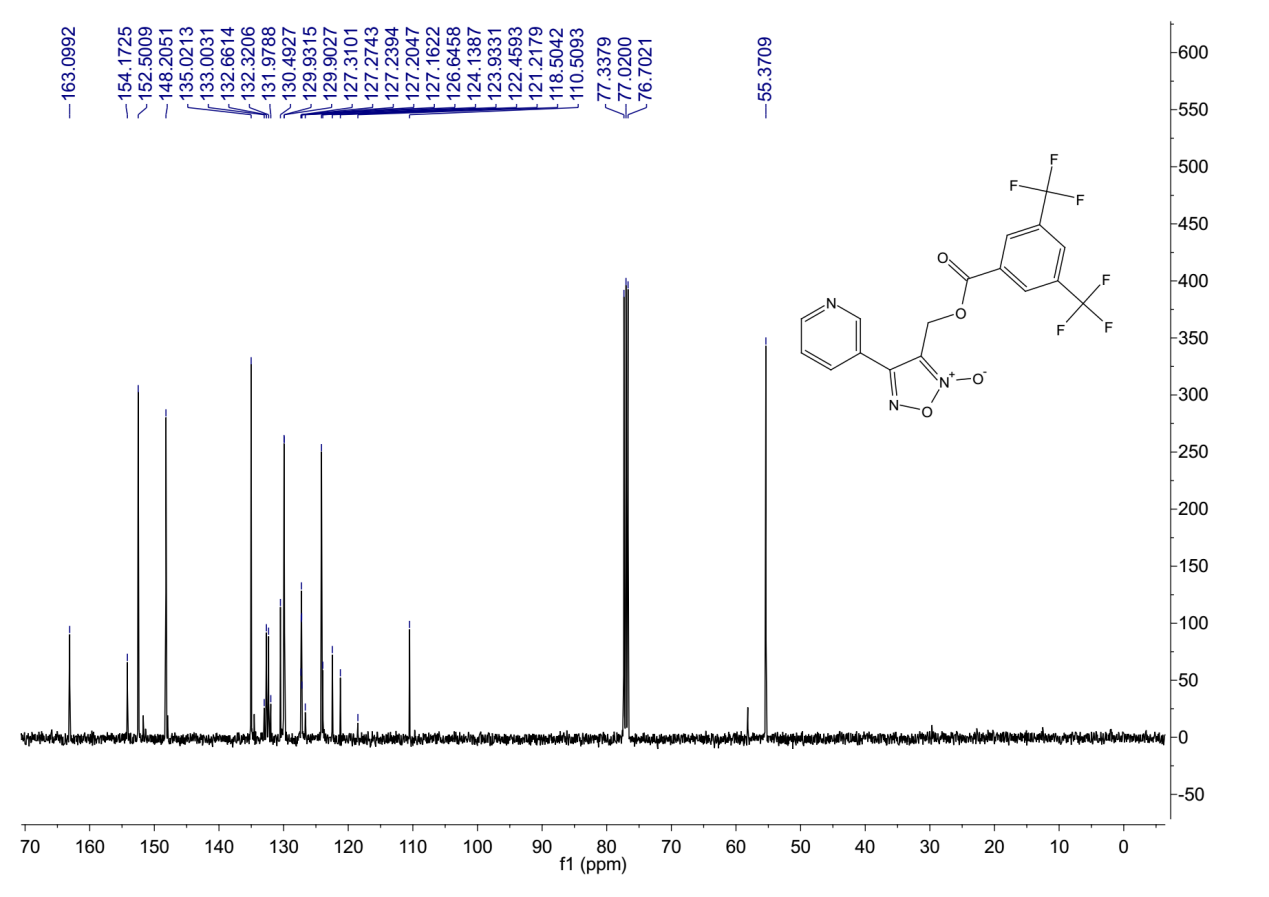


^13^C-NMR (101 MHz, Chloroform-*d*) spectrum of **7bi**.


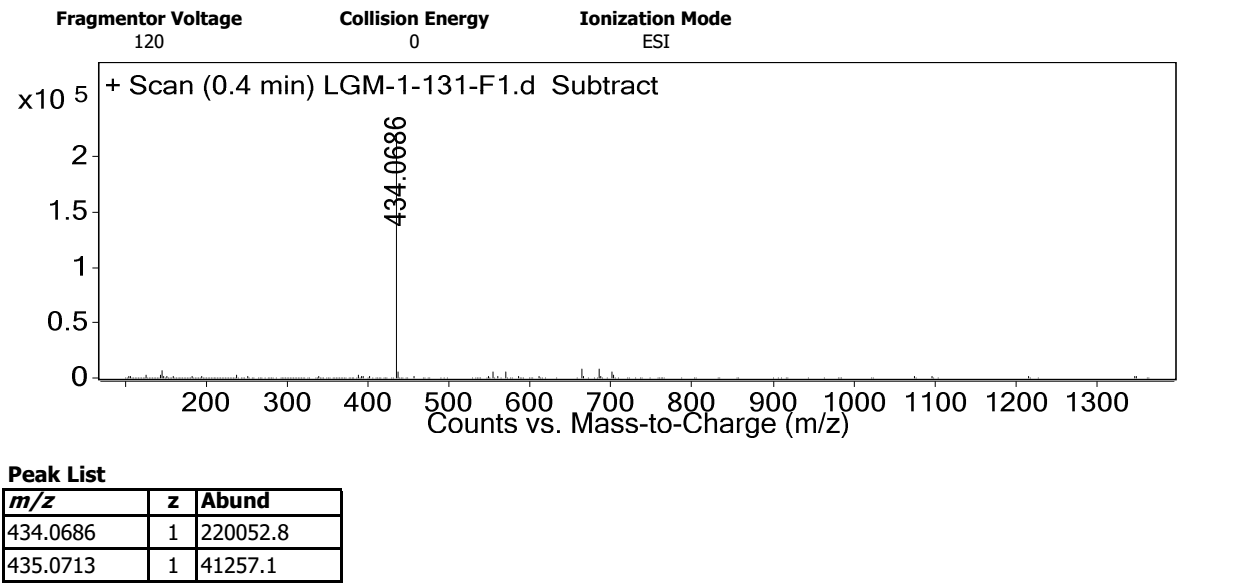


HRMS spectrum of **7bi**.


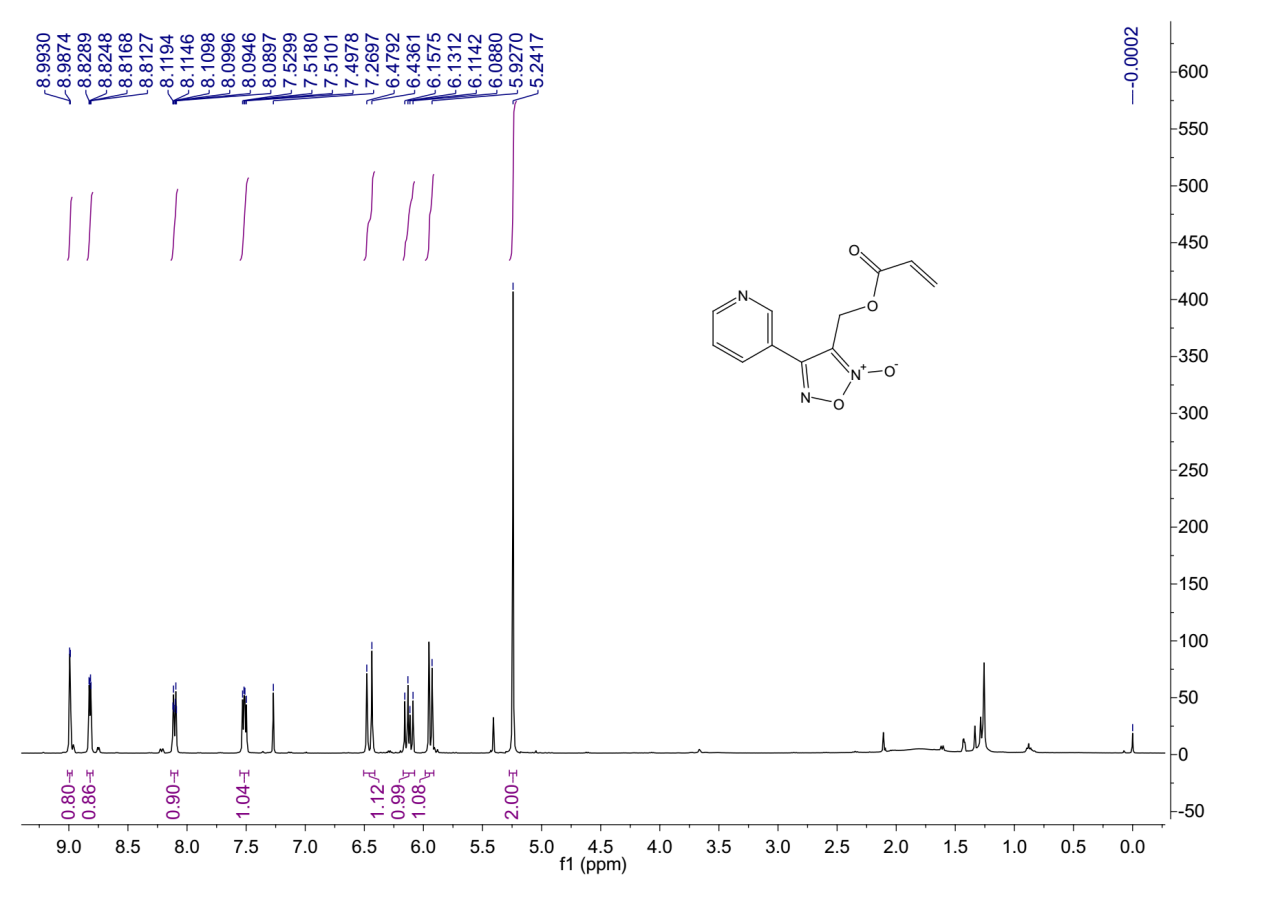


^1^H-NMR (400 MHz, Chloroform-*d*) spectrum of **7bj**.


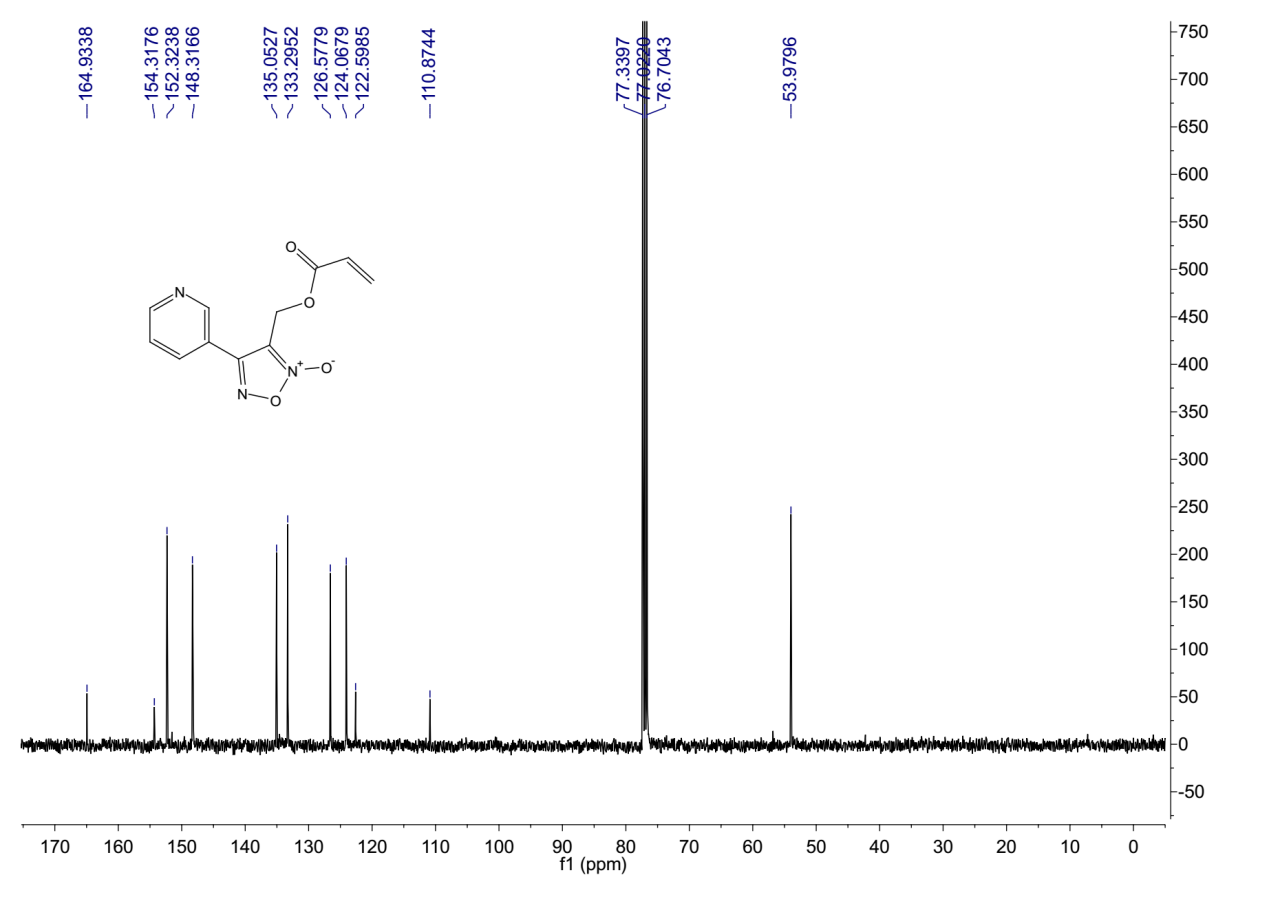


^13^C-NMR (101 MHz, Chloroform-*d*) spectrum of **7bj**.


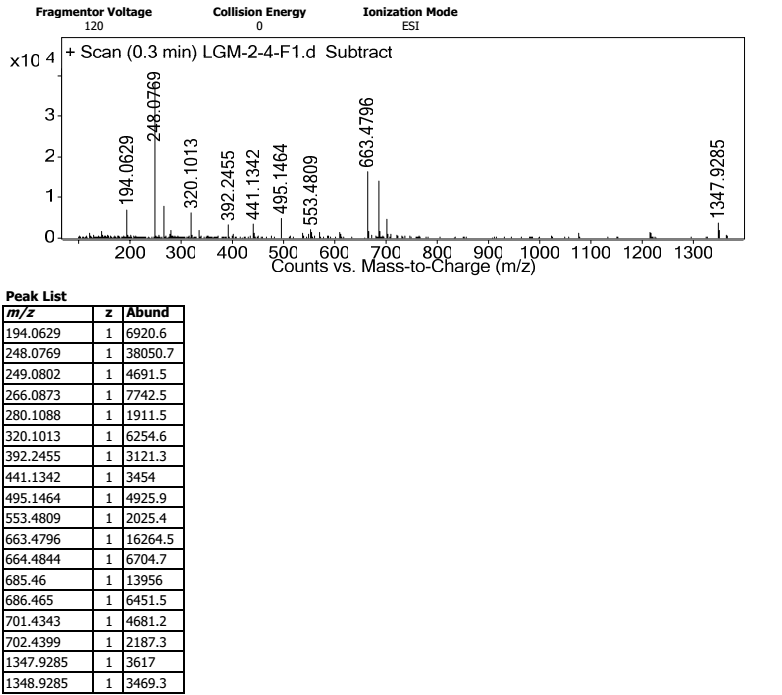


HRMS spectrum of **7bj**.


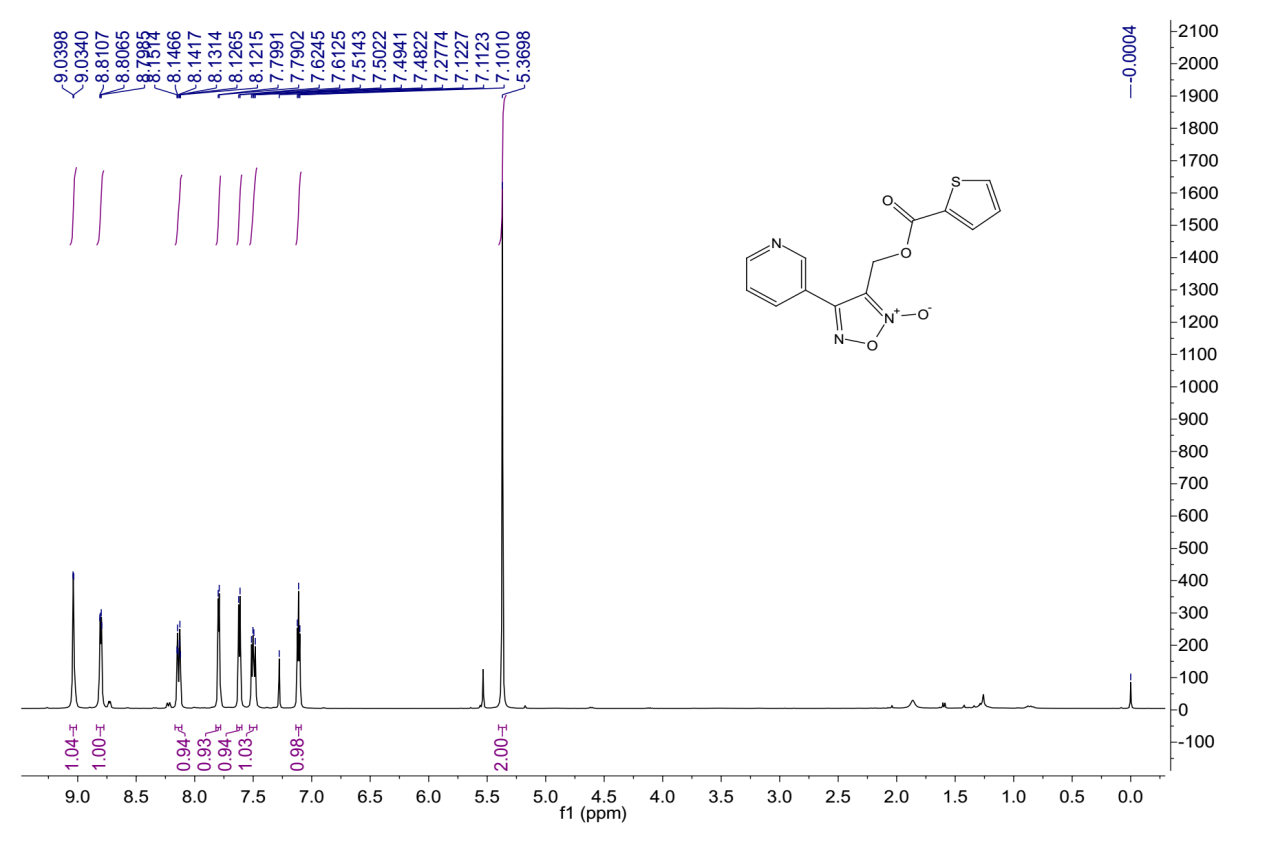


^1^H-NMR (400 MHz, Chloroform-*d*) spectrum of **7bk**.


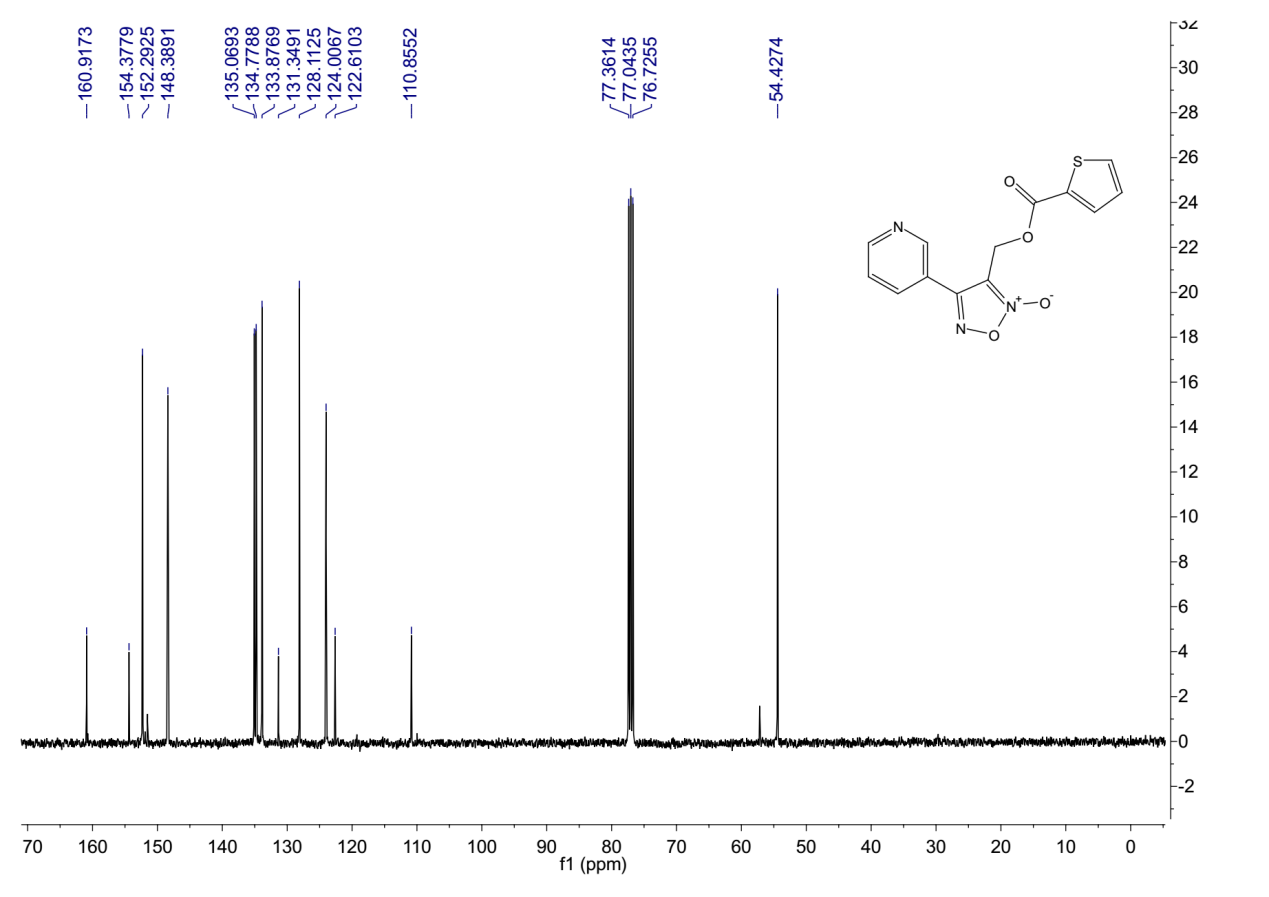


^13^C-NMR (101 MHz, Chloroform-d) spectrum of **7bk**.


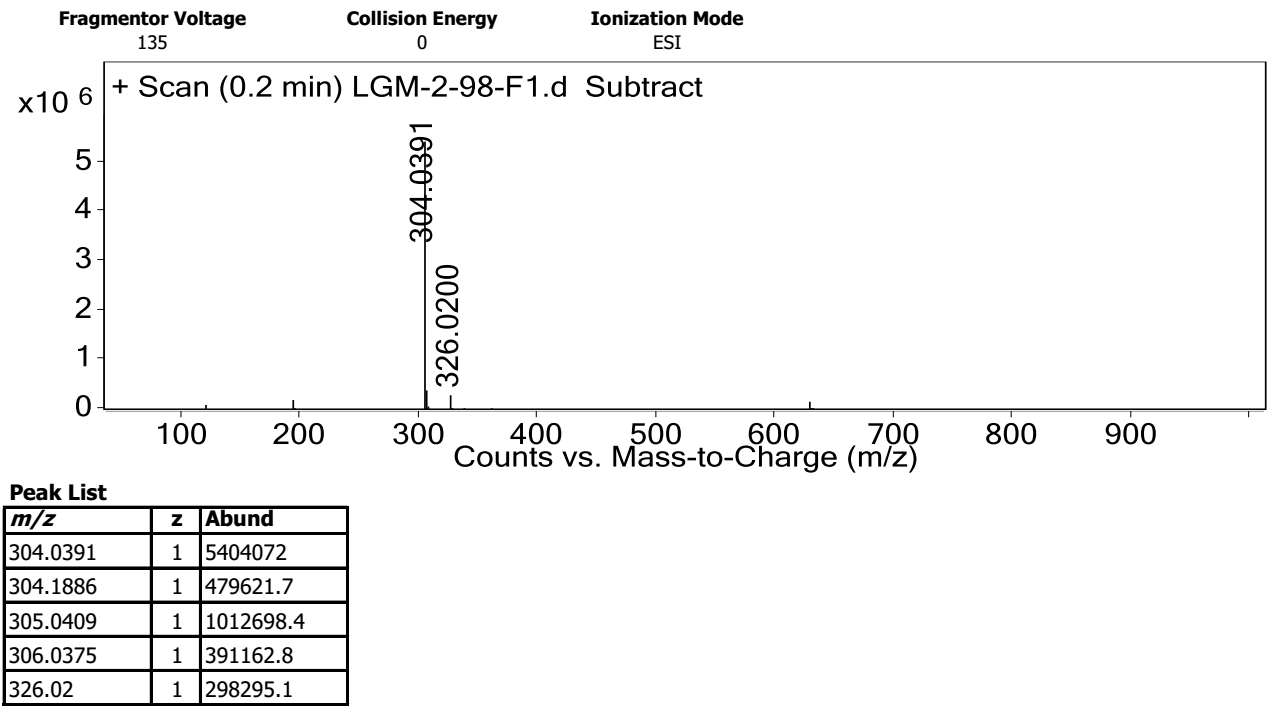


HRMS spectrum of **7bk**.


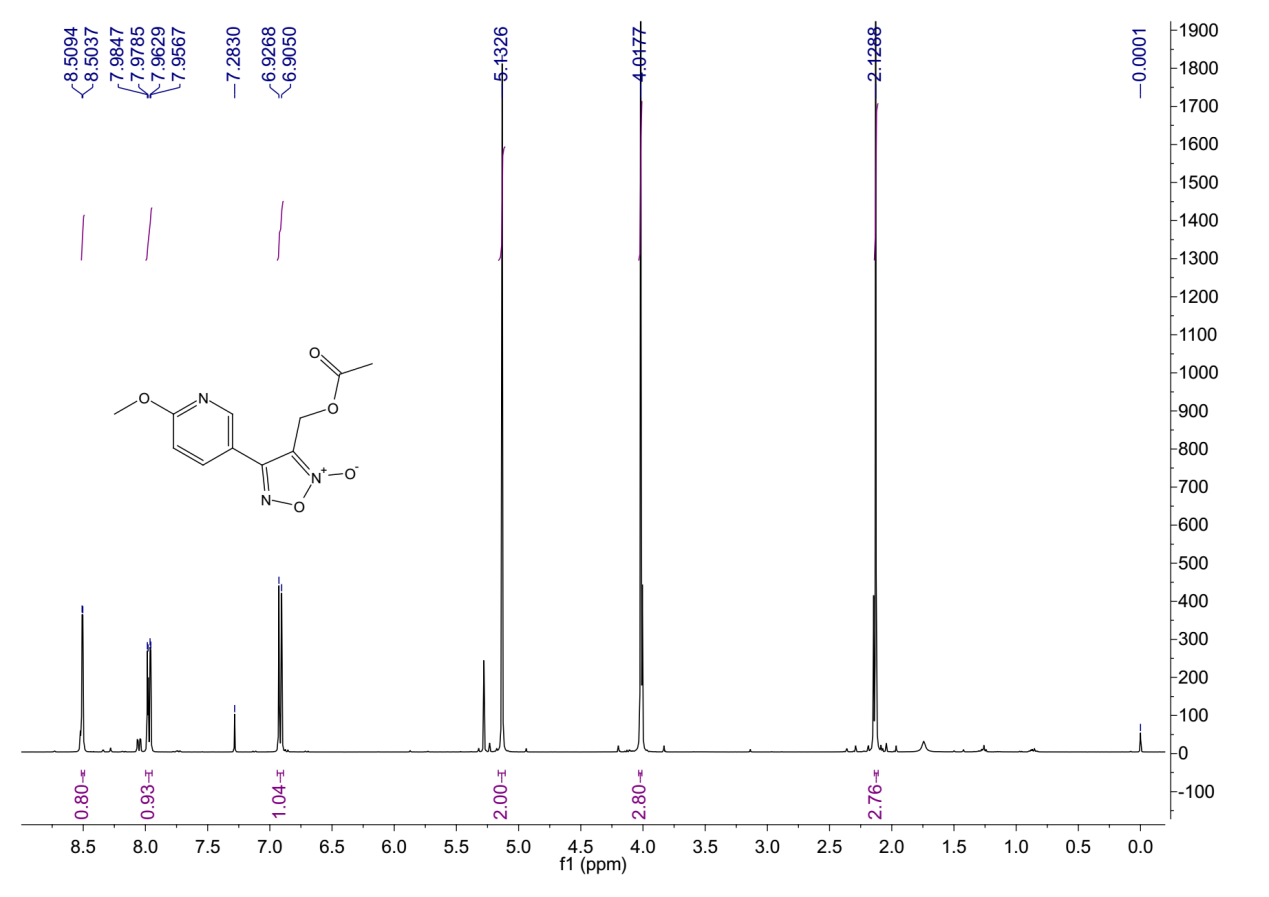


^1^H-NMR (400 MHz, Chloroform-*d*) spectrum of **7ca**.


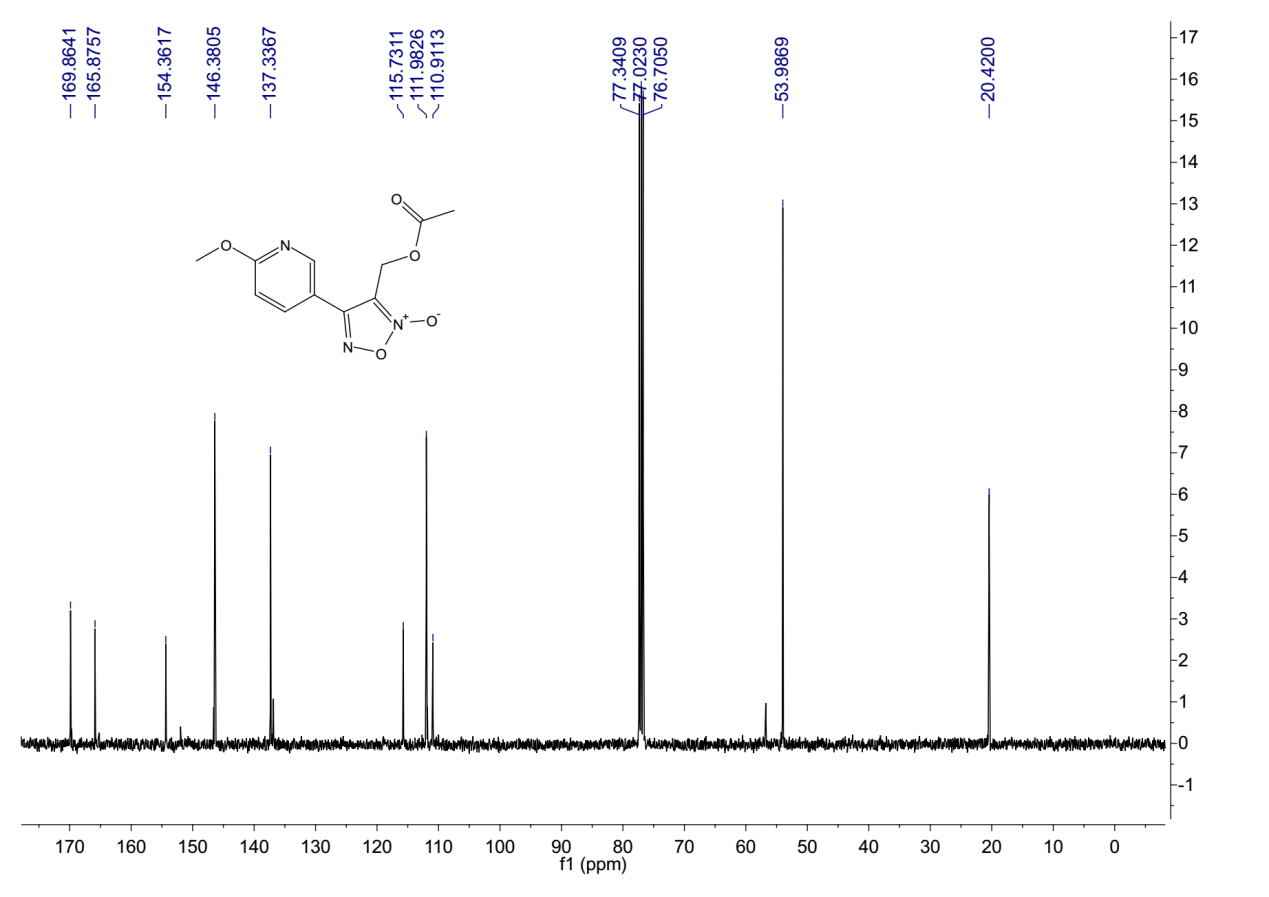


^13^C-NMR (101 MHz, Chloroform-d) spectrum of **7ca**.


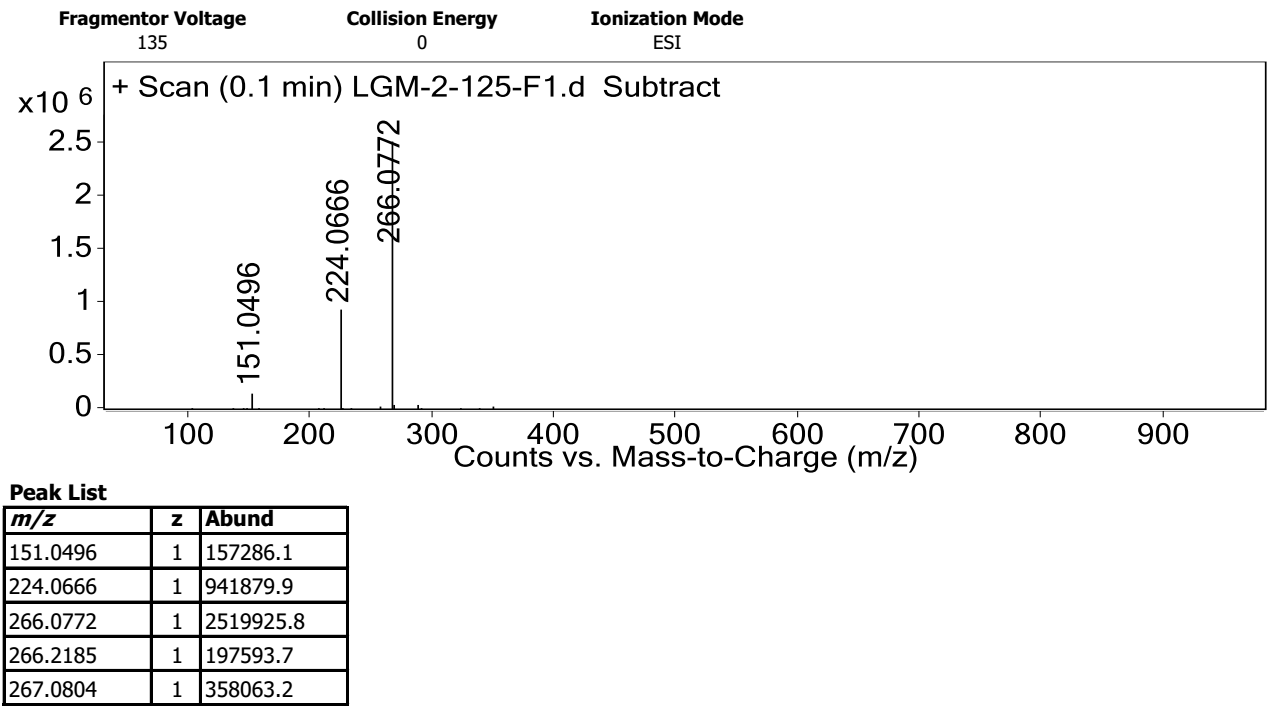


HRMS spectrum of **7ca**.


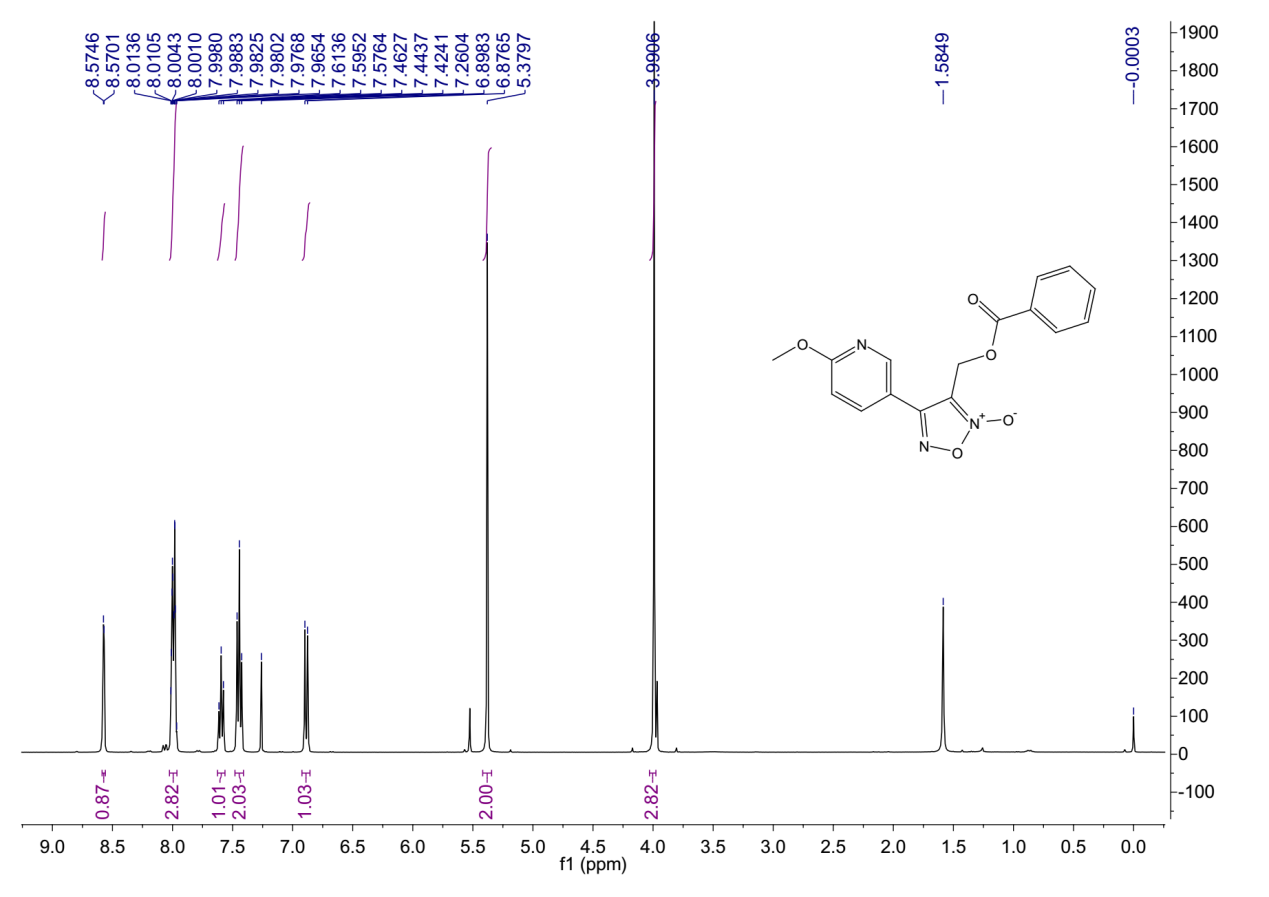


^1^H-NMR (400 MHz, Chloroform-*d*) spectrum of **7cf**.


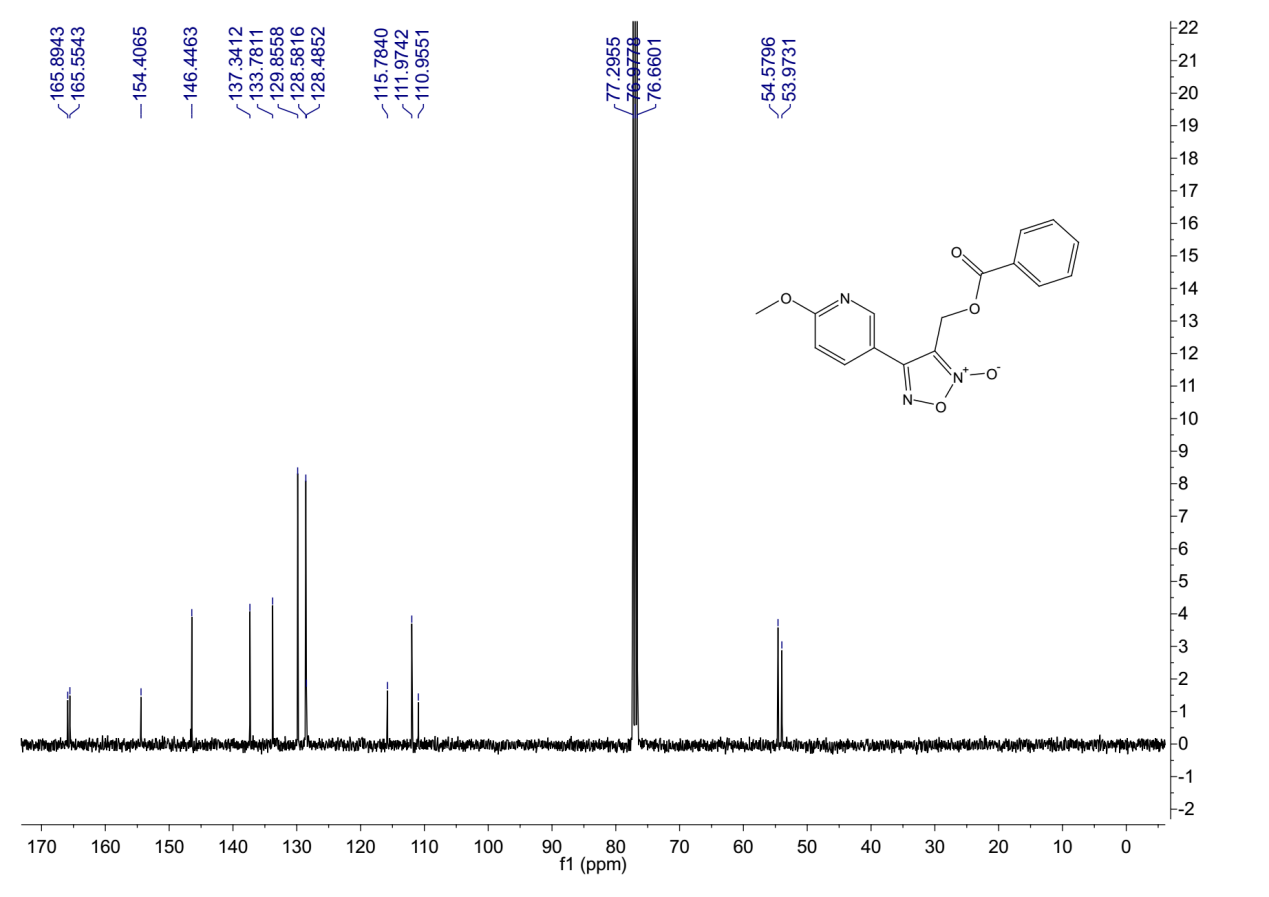


^13^C-NMR (101 MHz, Chloroform-*d*) spectrum of **7cf**.


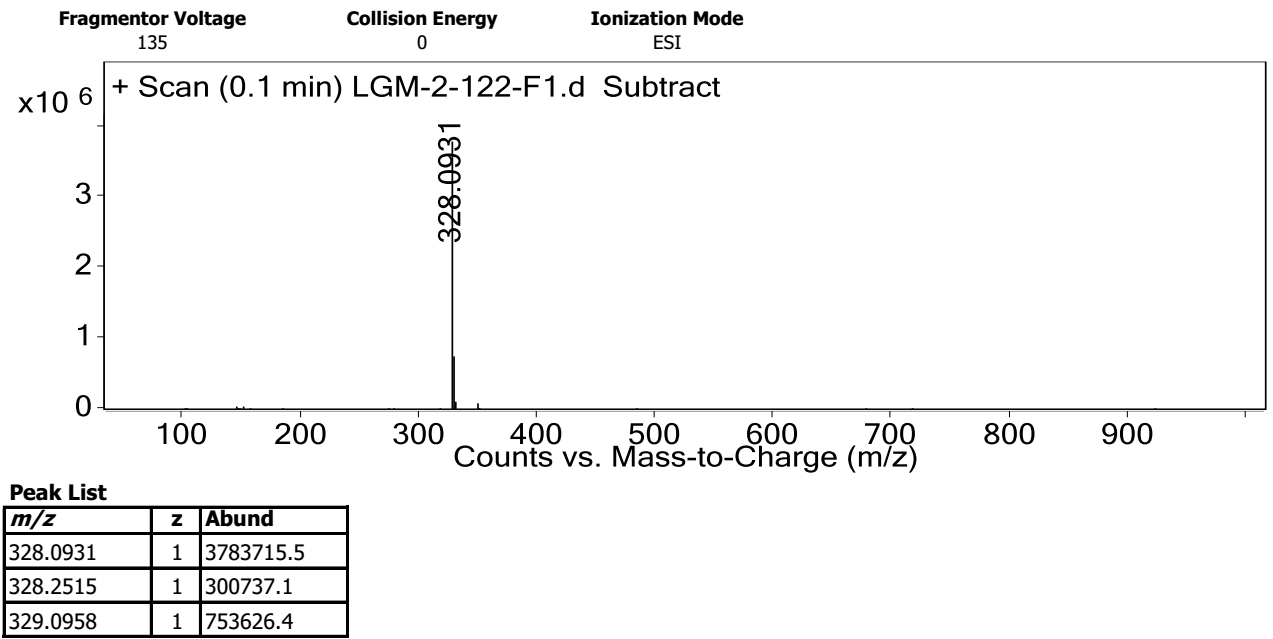


HRMS spectrum of **7cf**.


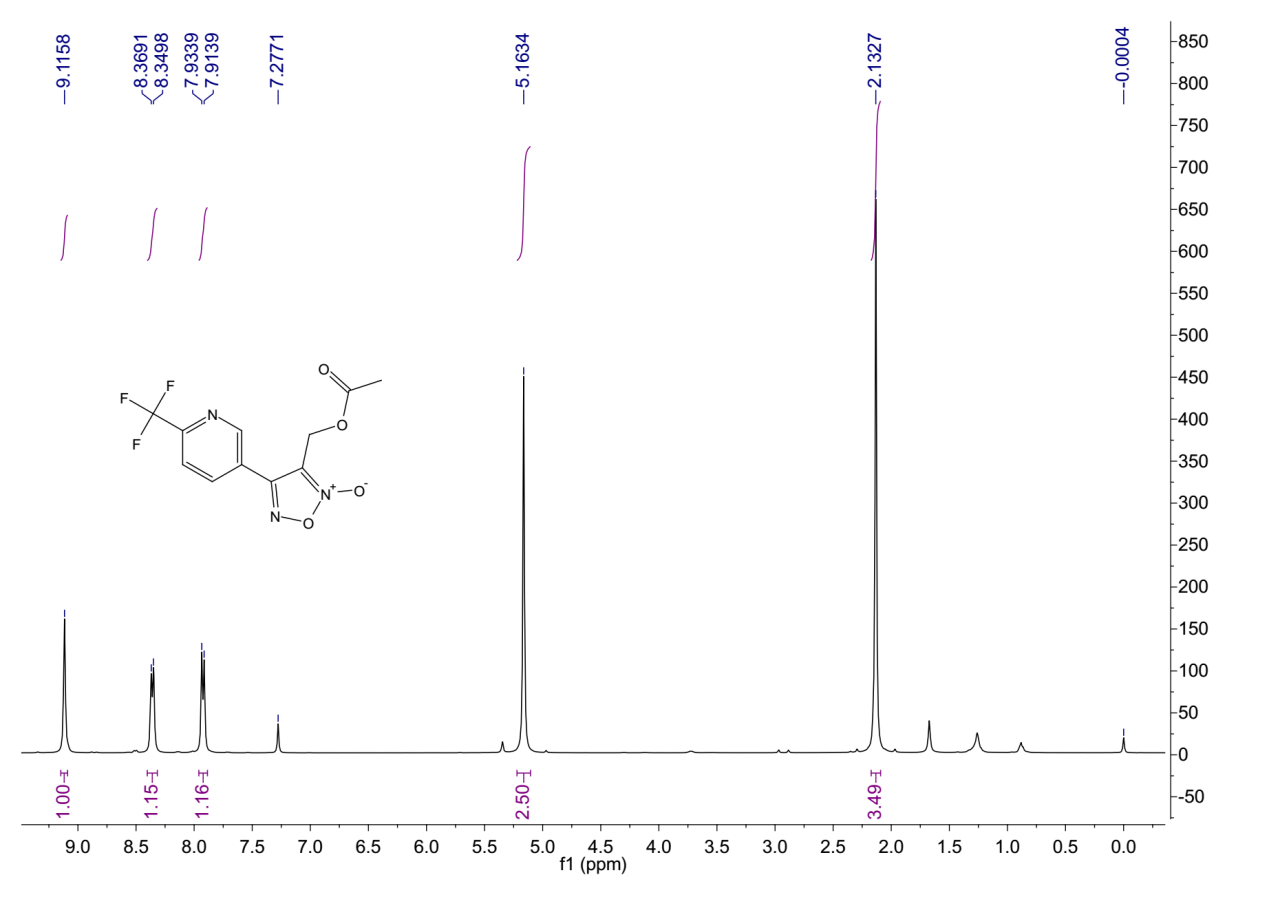


^1^H-NMR (400 MHz, Chloroform-*d*) spectrum of **7da**.


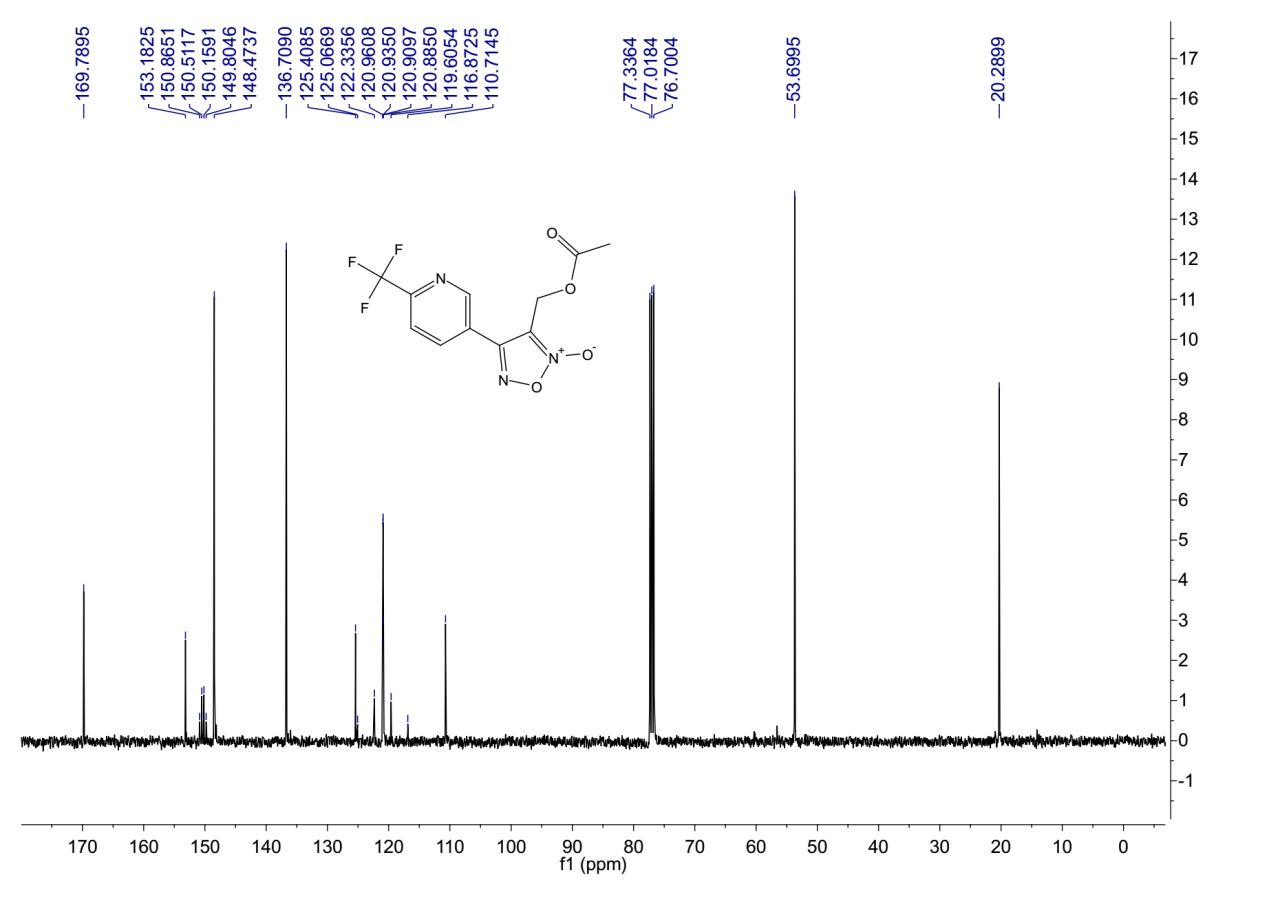


^13^C-NMR (101 MHz, Chloroform-d) spectrum of **7da**.


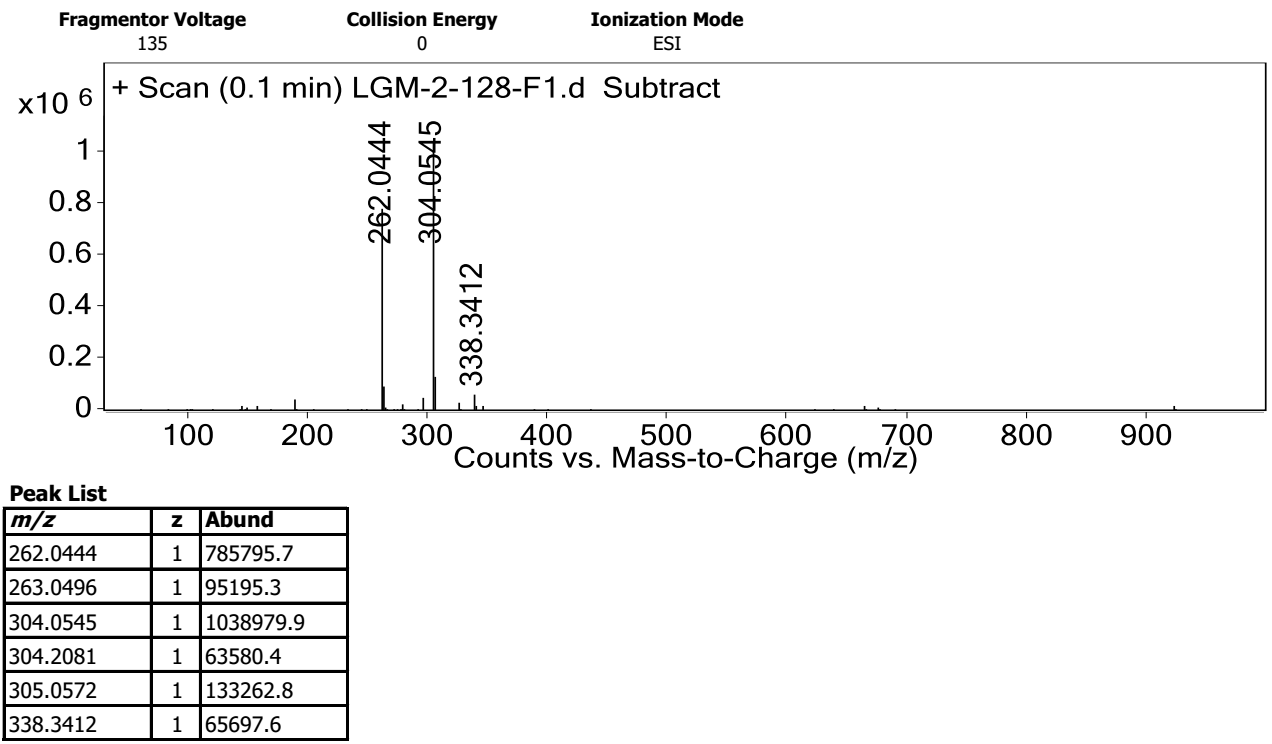


HRMS spectrum of **7da**.


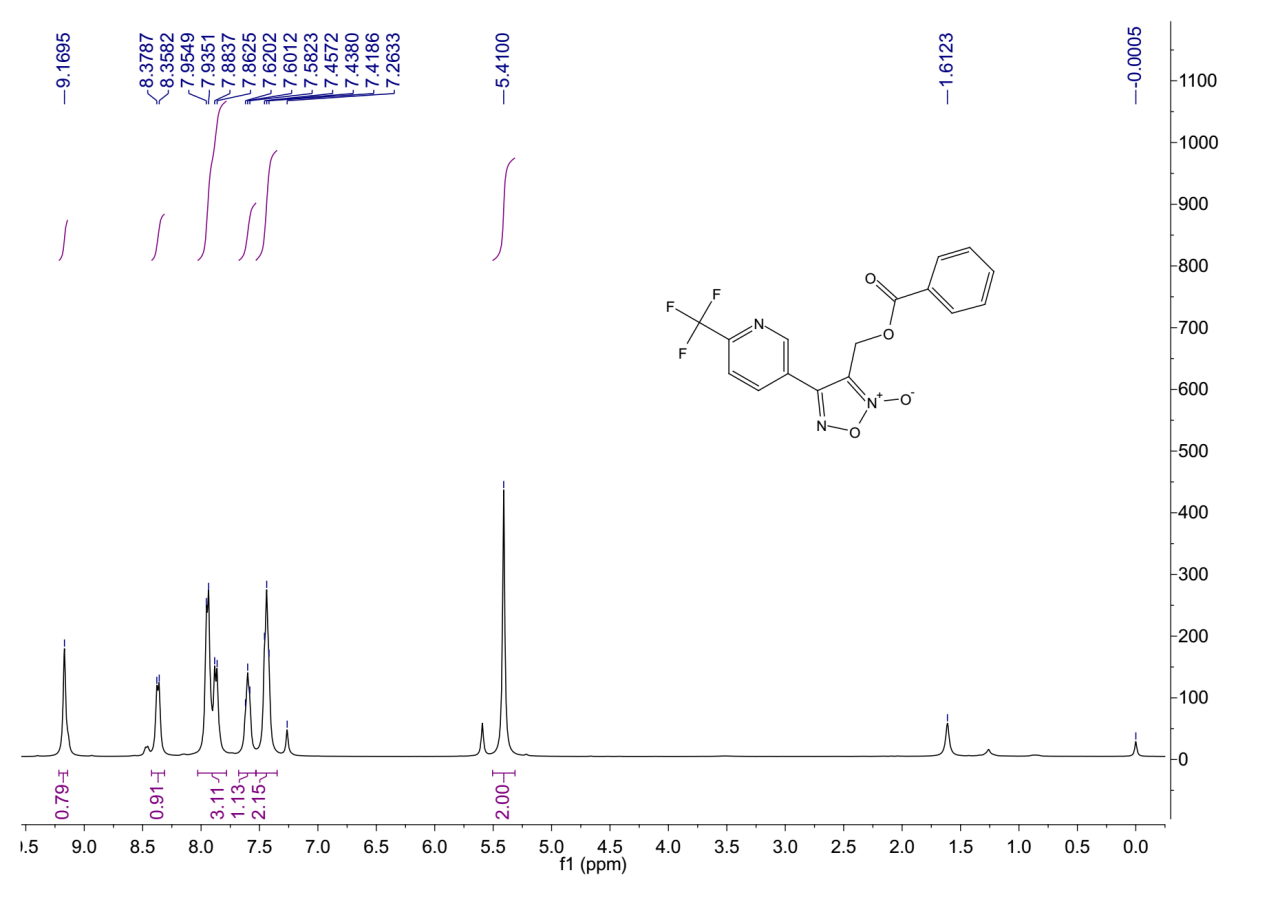


^1^H-NMR (400 MHz, Chloroform-*d*) spectrum of **7df**.


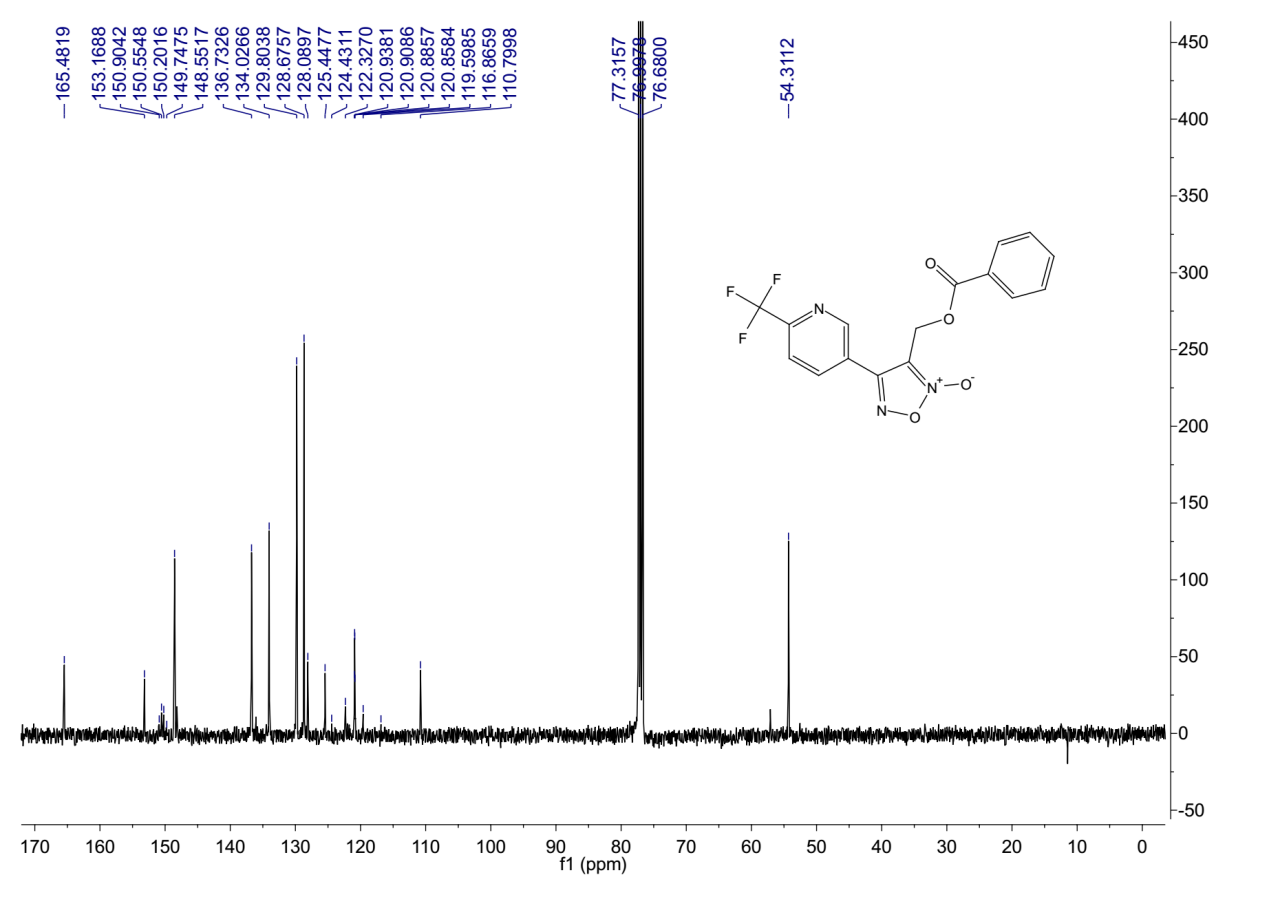


^13^C-NMR (101 MHz, Chloroform-*d*) spectrum of **7df**.


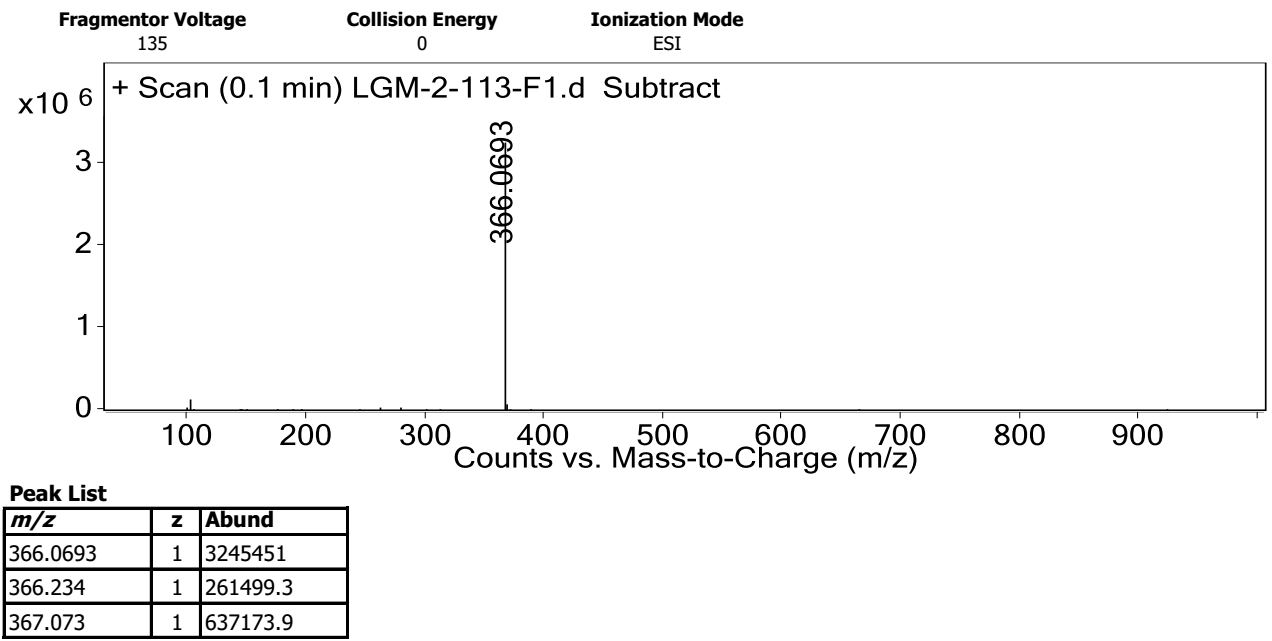


HRMS spectrum of **7df**.


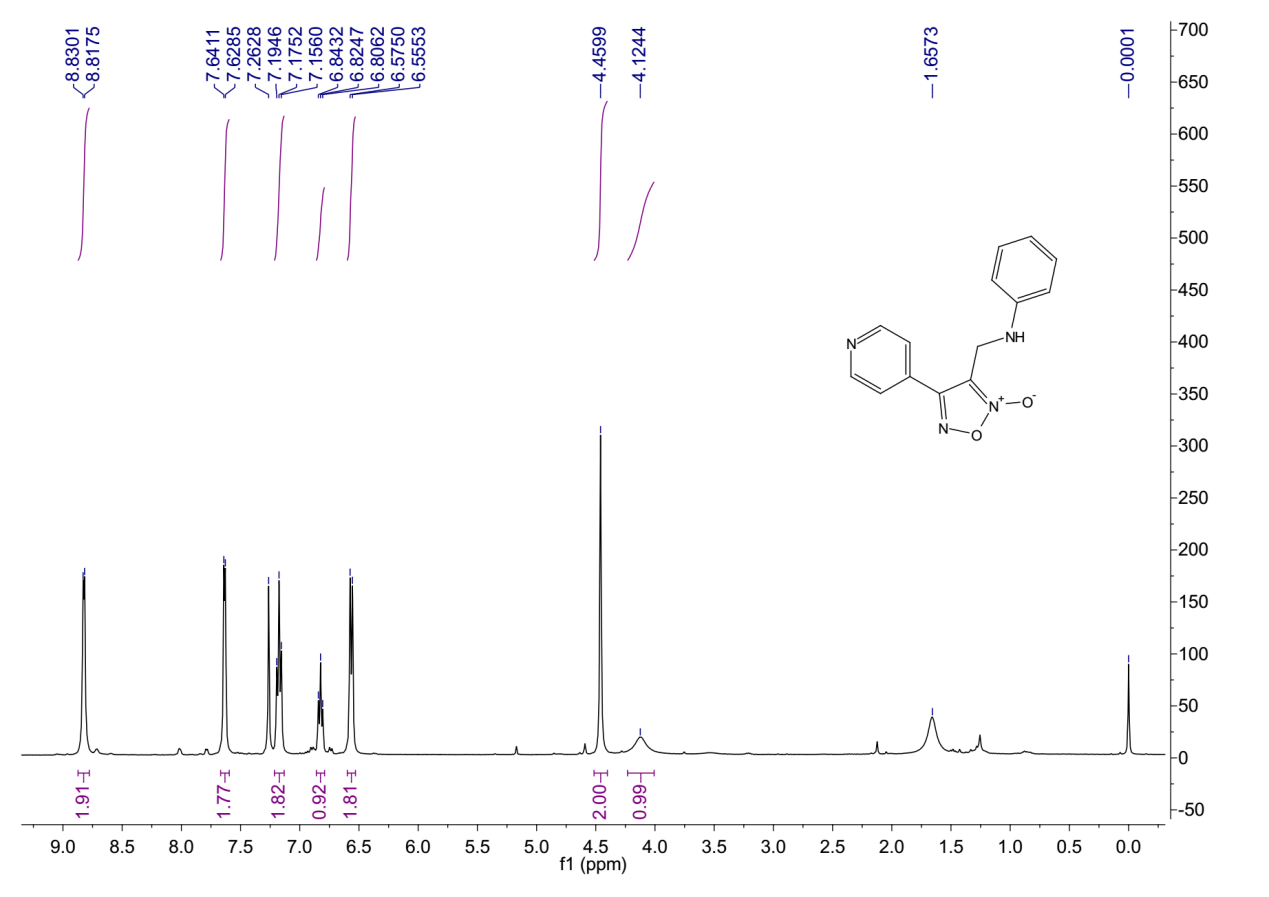


^1^H-NMR (400 MHz, Chloroform-*d*) spectrum of **9aa**.


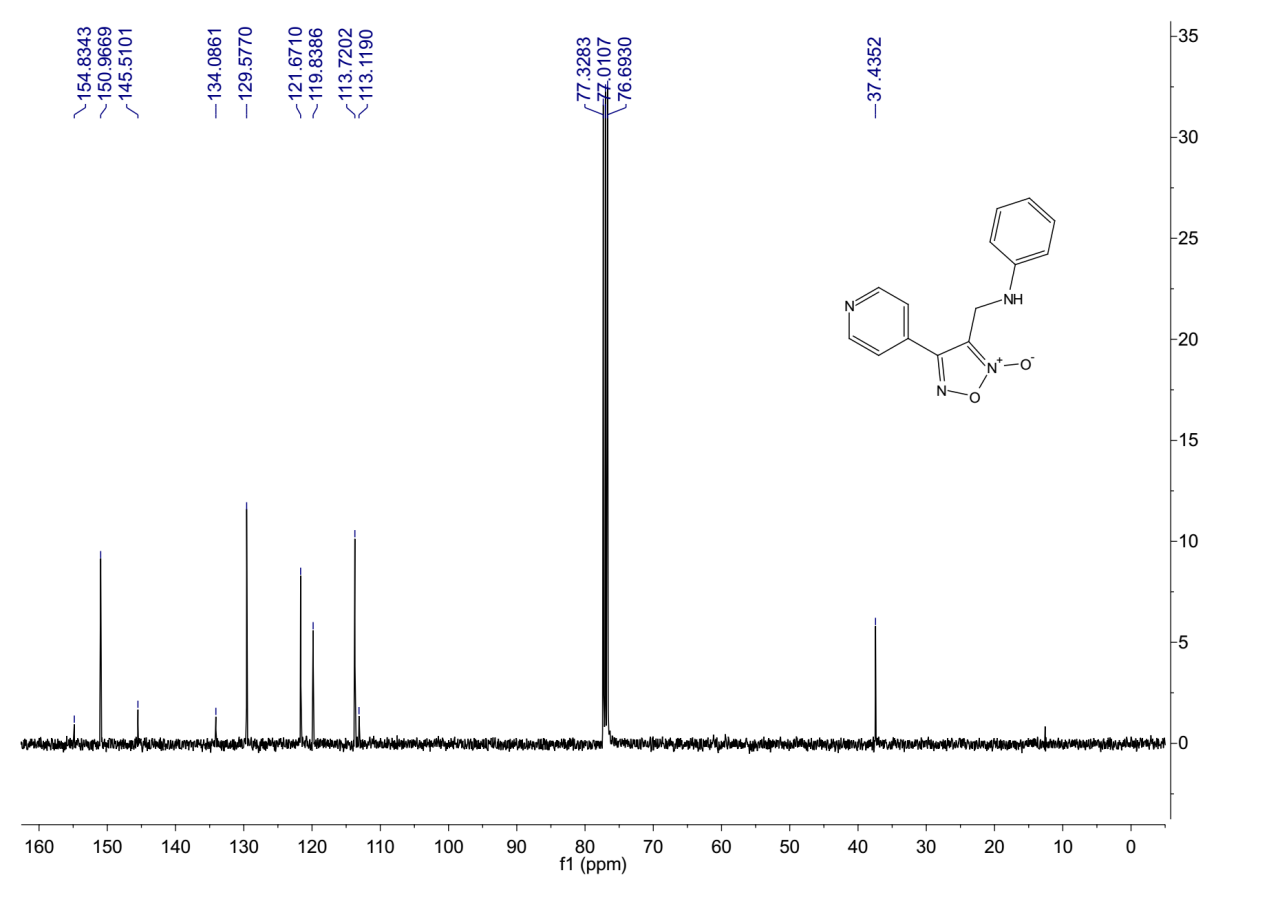


^13^C-NMR (101 MHz, Chloroform-d) spectrum of **9aa**.


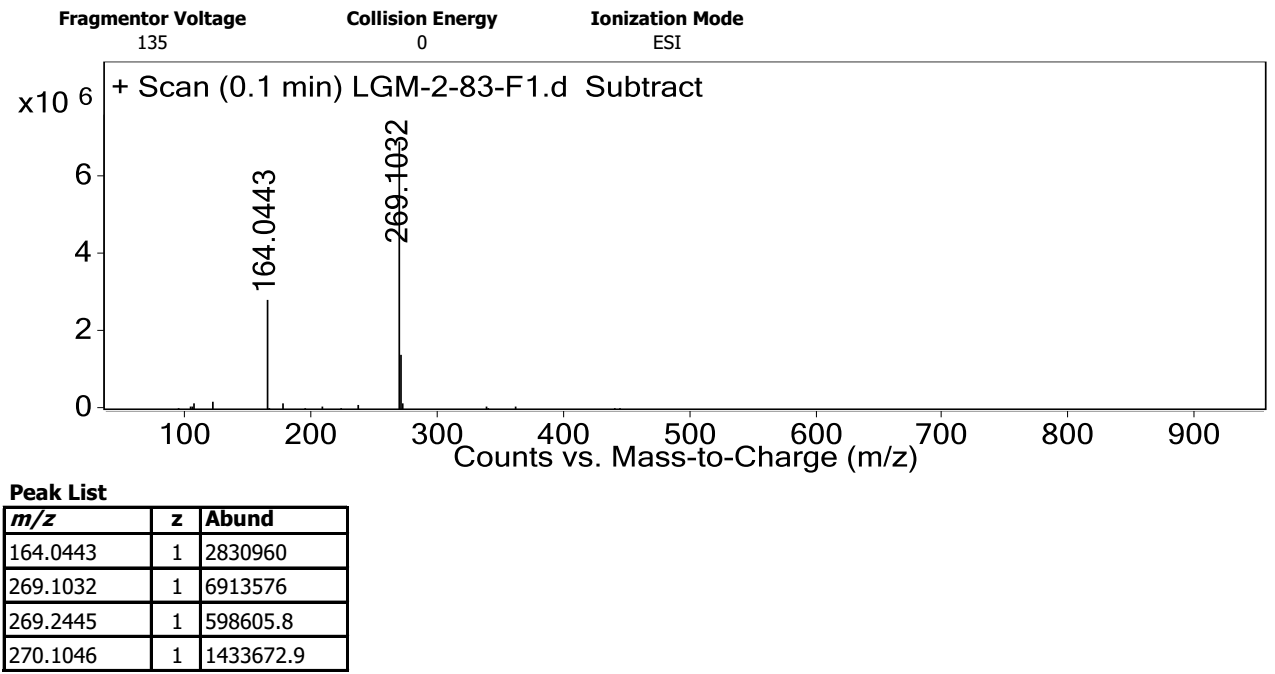


HRMS spectrum of **9aa**.


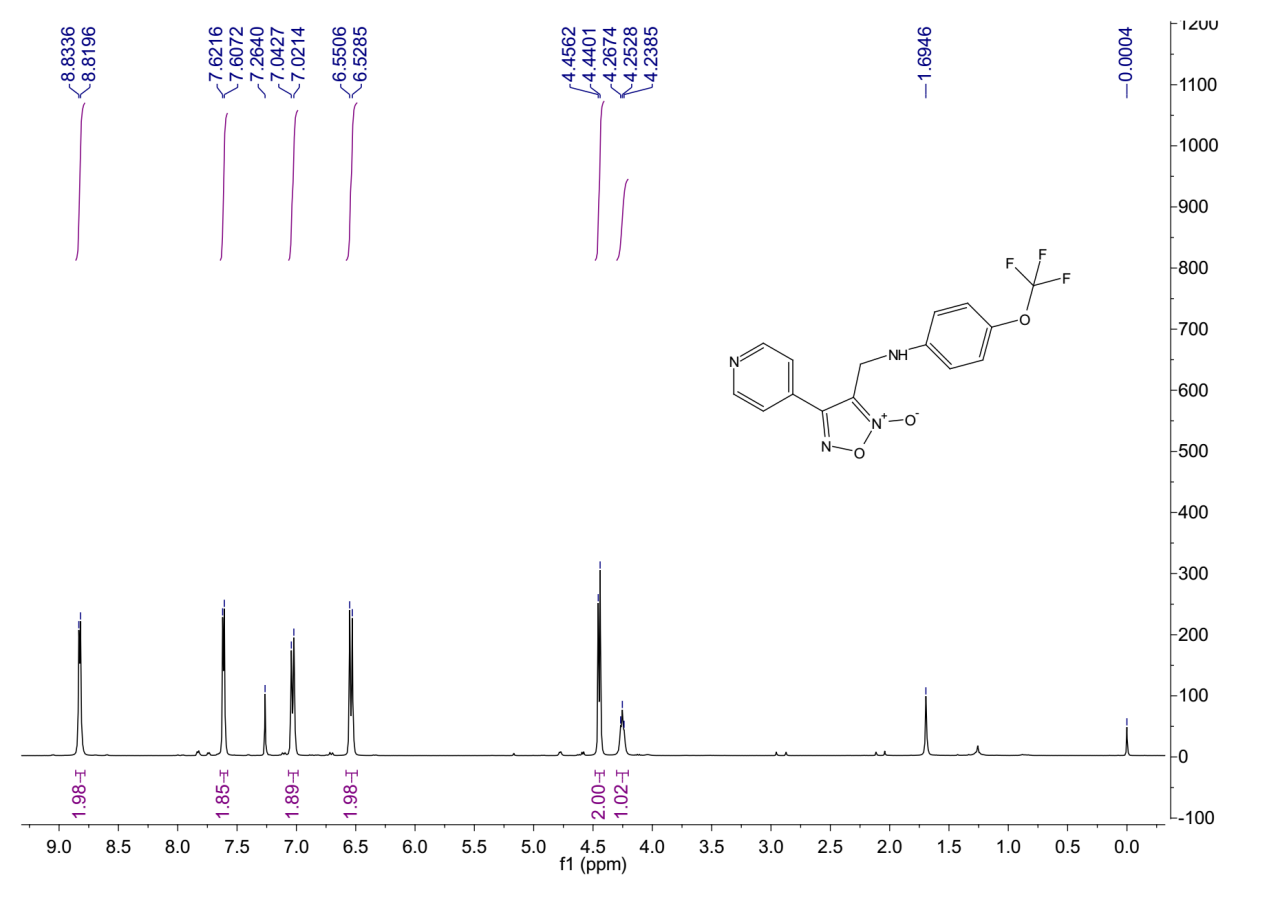


^1^H-NMR (400 MHz, Chloroform-*d*) spectrum of **9ab**.


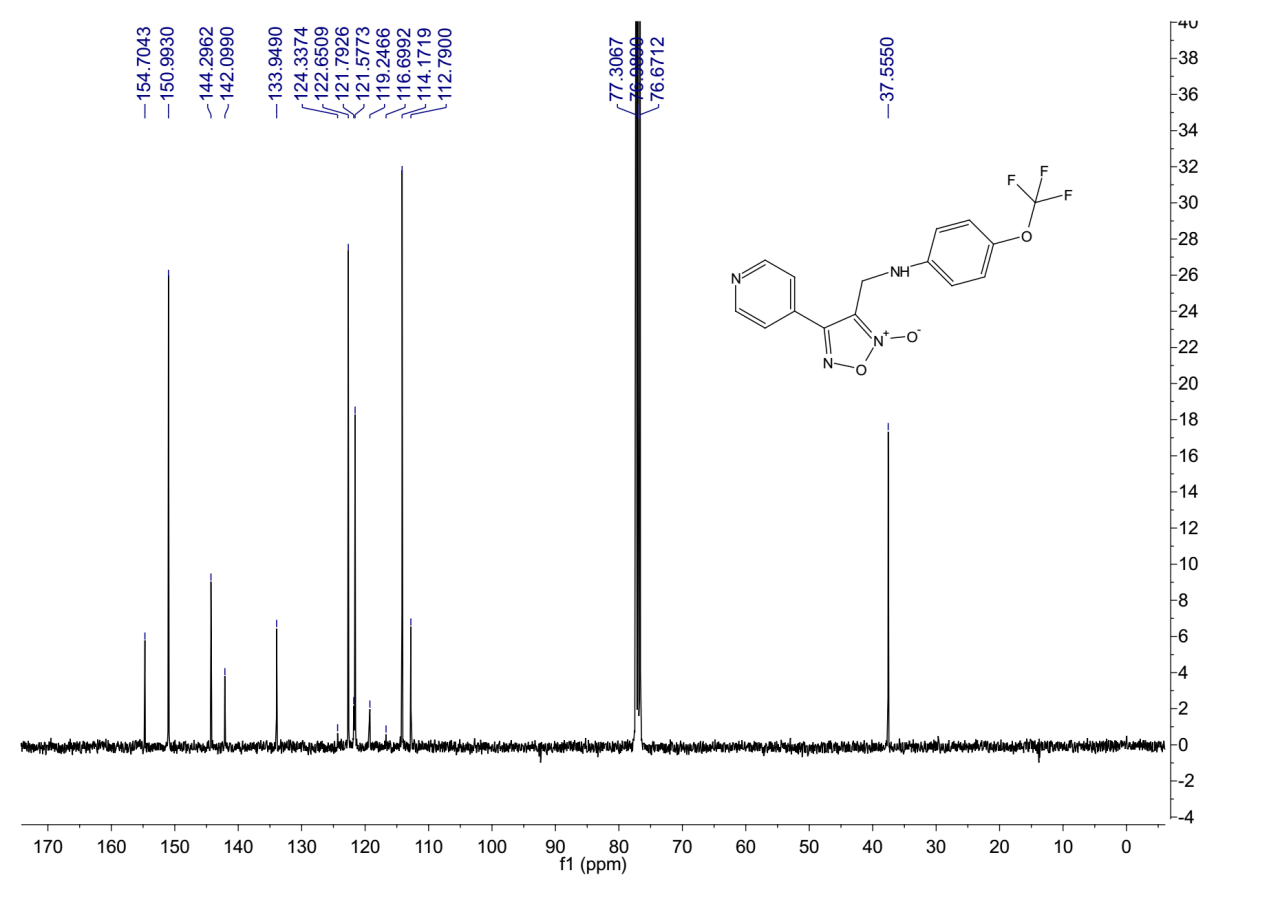


^13^C-NMR (101 MHz, Chloroform-*d*) spectrum of **9ab**.


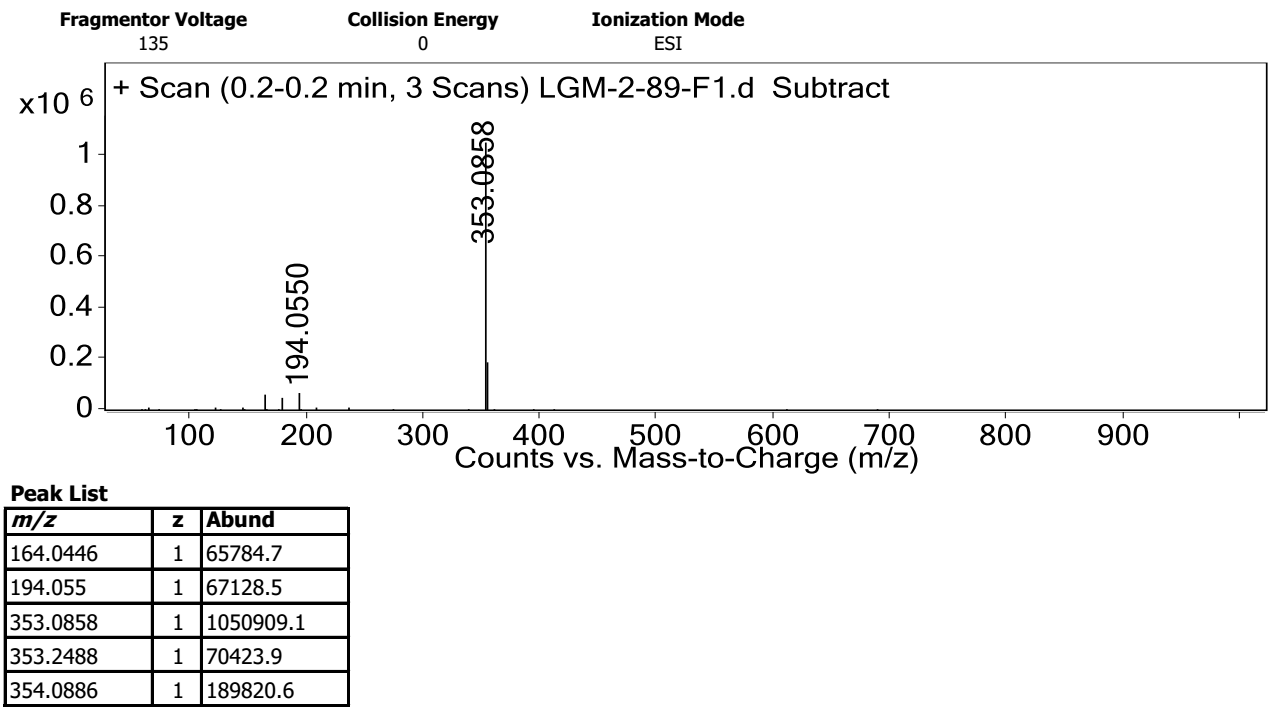


HRMS spectrum of **9ab**.


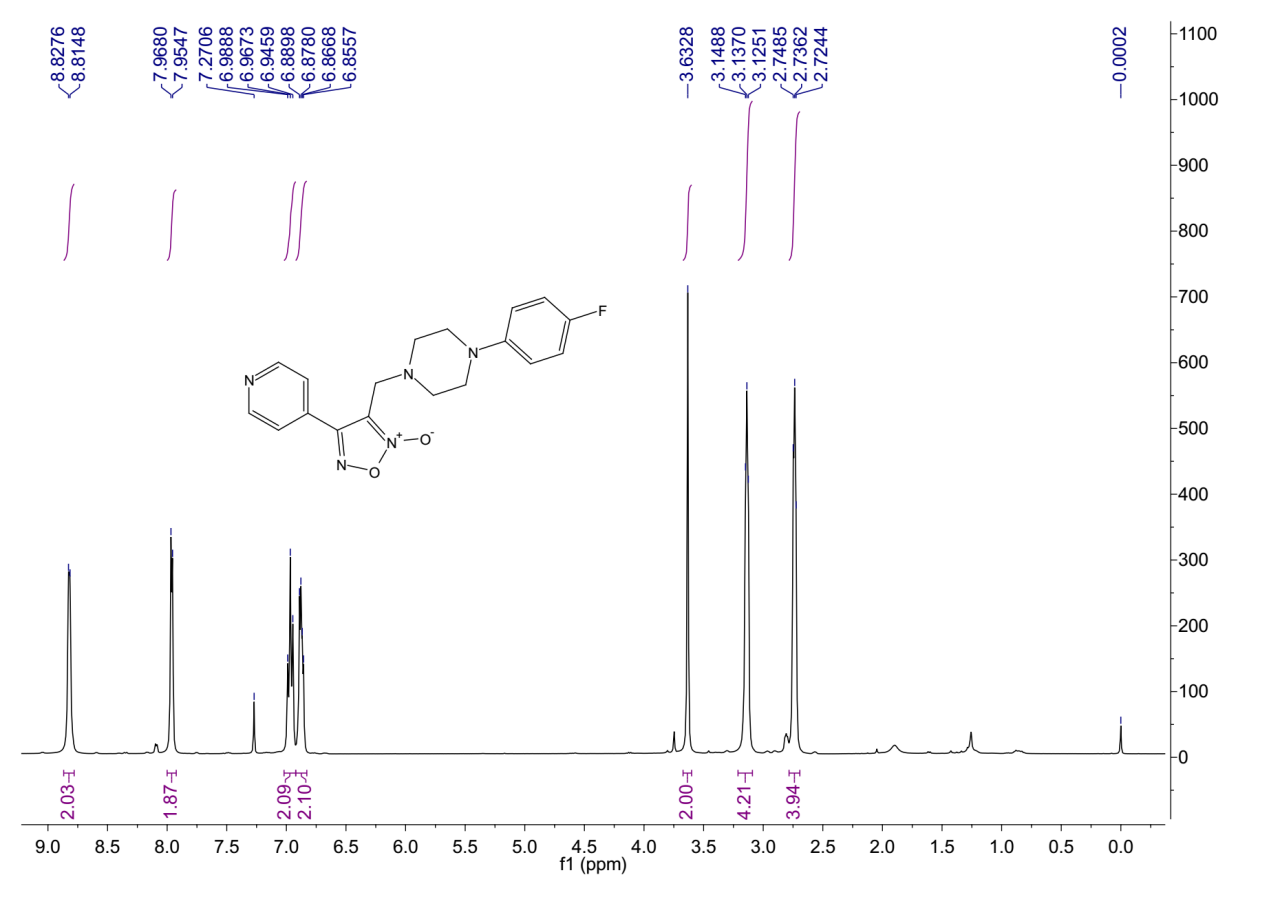


^1^H-NMR (400 MHz, Chloroform-*d*) spectrum of **9ac**.


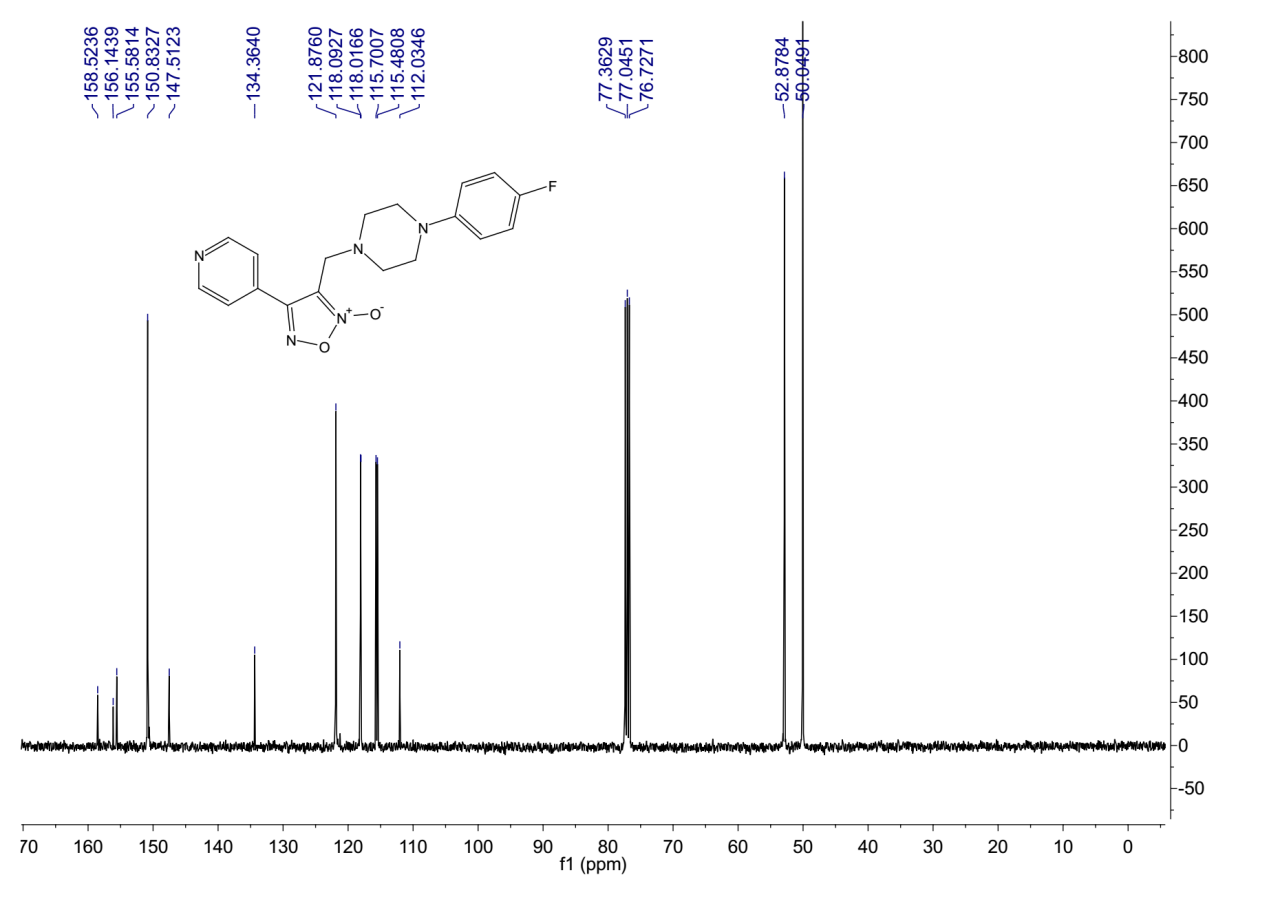


^13^C-NMR (101 MHz, Chloroform-*d*) spectrum of **9ac**.

HRMS spectrum of **9ac**.

^1^H-NMR (400 MHz, Chloroform-*d*) spectrum of **9ad**.

^13^C-NMR (101 MHz, Chloroform-*d*) spectrum of **9ad**.

HRMS spectrum of **9ad**.

^1^H-NMR (400 MHz, Chloroform-*d*) spectrum of **9ba**.

^13^C-NMR (101 MHz, Chloroform-*d*) spectrum of **9ba**.

HRMS spectrum of **9ba**.

^1^H-NMR (400 MHz, DMSO-*d*6) spectrum of **9bb**.

^13^C-NMR (101 MHz, DMSO-*d*6) spectrum of **9bb**.

HRMS spectrum of **9bb**.

^1^H-NMR (400 MHz, Chloroform-*d*) spectrum of **9bc**.

^13^C-NMR (101 MHz, Chloroform-*d*) spectrum of **9bc**.

HRMS spectrum of **9bc**.

^1^H-NMR (400 MHz, Chloroform-*d*) spectrum of **9bd**.

^13^C-NMR (101 MHz, Chloroform-*d*) spectrum of **9bd**.

HRMS spectrum of **9bd**.

^1^H-NMR (400 MHz, Chloroform-*d*) spectrum of **9be**.

^13^C-NMR (101 MHz, Chloroform-*d*) spectrum of **9be**.

HRMS spectrum of **9be**.

^1^H-NMR (400 MHz, Chloroform-*d*) spectrum of **9ca**.

^13^C-NMR (101 MHz, Chloroform-*d*) spectrum of **9ca**.

HRMS spectrum of **9ca**.

^1^H-NMR (400 MHz, Chloroform-*d*) spectrum of **9da**.

^13^C-NMR (101 MHz, Chloroform-*d*) spectrum of **9da**.

HRMS spectrum of **9da**.

**References**

1. Rai G, Thomas CJ, Leister W, Maloney DJ. Synthesis of oxadiazole-2-oxide analogues as potential antischistosomal agents. Tetrahedron Lett. 2009;50:1710-3.
2. Stambasky J, Malkov AV, Kocovsky P. Preparation of Boc-protected cinnamyl-type alcohols: A comparison of the Suzuki-Miyaura coupling, cross-metathesis, and Horner-Wadsworth-Emmons approaches and their merit in parallel synthesis. Collect. Czech Chem. C. 2008;73:705-32.
3. Jiang X, Hartwig JF. Iridium‐catalyzed enantioselective allylic substitution of aliphatic esters with silyl ketene acetals as the ester enolates. Angew. Chem. Int. Edit. 2017;56:8887-91.
4. Jung HJ, Park HJ, Shin YG, Kim YS, Yoon SH. Bioisoster of caffeic acid: syntheses of 1-hydroxy-2-pyridone analogues. Bull. Korean Chem. Soc. 2008;29:2277-80.
5. Thompson AM, Sutherland HS, Palmer BD, Kmentova I, Blaser A, Franzblau SG, et al. Synthesis and structure–activity relationships of varied ether linker analogues of the antitubercular drug (6S)-2-nitro-6-{[4-(trifluoromethoxy)benzyl]oxy}-6,7-dihydro-5H-imidazo[2,1-b][1,3]oxazine (PA-824). J. Med. Chem. 2011;54:6563-85.
6. Zartman AE, Duong LT, Fernandez-Metzler C, Hartman GD, Leu CT, Prueksaritanont T, et al. Nonpeptide α_v_β_3_ antagonists: identification of potent, chain-shortened 7-oxo RGD mimetics. Bioorg. Med. Chem. Lett. 2005;15:1647-50.
